# A database and checklist of geometrid moths (Lepidoptera) from Colombia

**DOI:** 10.3897/BDJ.9.e68693

**Published:** 2021-09-03

**Authors:** Leidys Murillo-Ramos, Pasi Sihvonen, Gunnar Brehm, Indiana C. Ríos-Malaver, Niklas Wahlberg

**Affiliations:** 1 Universidad de Sucre, Sincelejo, Colombia Universidad de Sucre Sincelejo Colombia; 2 University of Helsinki, Helsinki, Finland University of Helsinki Helsinki Finland; 3 Universität Jena, Jena, Germany Universität Jena Jena Germany; 4 Colecciones Biológicas, Instituto de Investigación de Recursos Biológicos Alexander von Humboldt, Boyacá, Colombia Colecciones Biológicas, Instituto de Investigación de Recursos Biológicos Alexander von Humboldt Boyacá Colombia; 5 McGuire Center for Lepidoptera and Biodiversity, University of Florida, Florida Museum of Natural History, Gainesville, United States of America McGuire Center for Lepidoptera and Biodiversity, University of Florida, Florida Museum of Natural History Gainesville United States of America; 6 Lund University, Lund, Sweden Lund University Lund Sweden

**Keywords:** occurrence records, DNA barcode, COI, moths, Colombia, Geometridae, Loopers.

## Abstract

**Background:**

Molecular DNA sequence data allow unprecedented advances in biodiversity assessments, monitoring schemes and taxonomic works, particularly in poorly-explored areas. They allow, for instance, the sorting of material rapidly into operational taxonomic units (such as BINs - Barcode Index Numbers), sequences can be subject to diverse analyses and, with linked metadata and physical vouchers, they can be examined further by experts. However, a prerequisite for their exploitation is the construction of reference libraries of DNA sequences that represent the existing biodiversity. To achieve these goals for Geometridae (Lepidoptera) moths in Colombia, expeditions were carried out to 26 localities in the northern part of the country in 2015–2019. The aim was to collect specimens and sequence their DNA barcodes and to record a fraction of the species richness and occurrences in one of the most biodiversity-rich countries. These data are the beginning of an identification guide to Colombian geometrid moths, whose identities are currently often provisional only, being morpho species or operational taxonomic units (OTUs). Prior to the current dataset, 99 Geometridae sequences forming 44 BINs from Colombia were publicly available on the Barcode of Life Data System (BOLD), covering 20 species only.

**New information:**

We enrich the Colombian Geometridae database significantly by including DNA barcodes, two nuclear markers, photos of vouchers and georeferenced occurrences of 281 specimens of geometrid moths from different localities. These specimens are classified into 80 genera. Analytical tools on BOLD clustered 157 of the mentioned sequences to existing BINs identified to species level, identified earlier by experts. Another 115 were assigned to BINs that were identified to genus or tribe level only. Eleven specimens did not match any existing BIN on BOLD and are, therefore, new additions to the database. It is likely that many BINs represent undescribed species. Nine short sequences (< 500bp) were not assigned to BINs, but identified to the lowest taxonomic category by expert taxonomists and with comparisons of type material photos. The released new genetic information will help to further progress the systematics of Geometridae. An illustrated catalogue of all new records allows validation of our identifications; it is also the first document of this kind for Colombian Geometridae. All specimens are deposited at the Museo de Zoología of Universidad de Sucre (MZUS), North Colombia. DNA BINs are reported in this study through dx.doi.org/10.5883/DS-GEOCO, the species occurrences are available on SIB Colombia https://sibcolombia.net/ and the Global Biodiversity Information Facility (GBIF) https://www.gbif.org/ through https://doi.org/10.15472/ucfmkh.

## Introduction

The moth family Geometridae is one of the most diverse lineages of Lepidoptera, with approximately 24,000 described species (Mitter et al. 2017). The Neotropical Region is more species-rich than any other biogeographical region with about 6,500 described species (Scoble 1999, Brehm et al. 2005), with the wet tropical Andes being the global diversity hotspot of the family (Brehm et al. 2016, Brehm et al. 2019). As with other species-rich insect groups, the species identification of geometrids is often hampered by the lack of taxonomic expertise, scattered, outdated and superficial literature or online sources. Even nowadays, interim taxonomy is often the only way to work on tropical insects and it is widely used to assign individuals to operational taxonomic units (OTUs), which are a proxy for species (Brito et al. 2016, Zenker et al. 2016, Rosero-garcIa et al. 2017, Strutzenberger et al. 2017).

However, interim taxonomy prevents comparisons between different studies and data cannot be combined, which precludes further research that needs reliable species identification. Amongst these are studies on the species distribution patterns, changes of abundance and systematic biology. An additional complication is the complex morphology of many insect taxa (homologies may be difficult to interpret due to quantitative variation) and the presence of cryptic species (Brehm et al. 2016). Recently, broad-level molecular phylogenetic studies in Geometridae have increased our understanding of their systematics and the studies have revealed that the Neotropics host a vast diversity of lineages that are in urgent need of taxonomic attention (Murillo-Ramos et al. 2019, Brehm et al. 2019, Sihvonen et al. 2020). To provide some examples, highly diverse genera like *Physocleora* Warren, 1897, *Idaea* Treitschke, 1825 and *Eupithecia* Curtis, 1825 contain mostly unidentified records on BOLD or on species occurrences platforms like GBIF, most likely hinting to a high percentage of undescribed species in these (and many other) taxa.

Over the last decade, the South American fauna of Geometridae has received increasing attention. Some species checklists, as well as taxonomic and ecological studies, have used a total evidence approach combining morphology, biology and molecular data to reveal the species diversity in, for example, Chile (Vargas and Hausmann 2008, Hausmann and Parra 2009, Ramos-Gonzalez et al. 2018), Peru (Brehm et al. 2011, Nino et al. 2019) and Ecuador (Brehm and Fiedler 2003, Hilt et al. 2006, Hilt et al. 2007, Bodner et al. 2010, Strutzenberger et al. 2011, Bodner et al. 2012, Strutzenberger et al. 2012, Brehm et al. 2013, Seifert et al. 2015, Brehm et al. 2016, Strutzenberger et al. 2017). However, comprehensive checklists are still lacking for any Latin Amerian country. Provisional checklists suggests that a small country like Costa Rica holds more than 1,100 geometrid species (http://www.tropicleps.ch/?page=1&fam=geo). Tropical Andean countries are expected to comprise the highest diversity; Brehm et al. (2016) reported more than 2,000 species alone in only a small part of south-eastern Ecuador.

**Figure 1.**
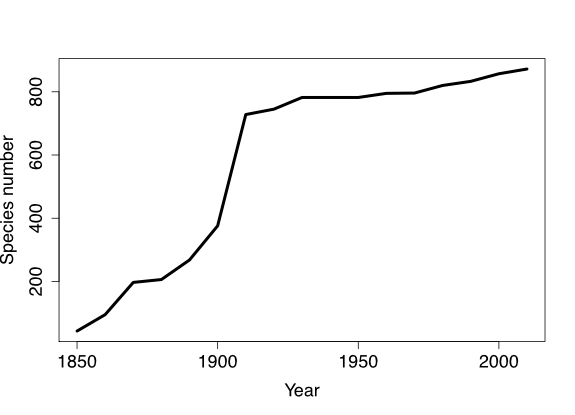
Cumulative numbers of name-bearing taxa, described in different decades with type locality in Colombia (data from Scoble 1999).

Unfortunately, knowledge of the geometrid fauna has only increased regionally and the fauna is still very poorly explored in most regions and countries. For instance, in Colombia, geometrids have received little attention, are still poorly collected and poorly known, limiting the accuracy and speed of biodiversity studies. Historically, the Colombian Geometridae fauna has been the target of descriptive taxonomy, most of the new species being described from Bogota, surroundings and centre of Colombia. Altogether, 1026 species-level taxa have been recorded from different localities from Colombia. Currently, about 872 of those are considered valid species (data derived from Scoble 1999, updated by authors with recent published literature and through the Zoological Record database) (Fig. 1).

Considering the high diversity of ecosystems in Colombia, which include dry and wet tropical forest in three Andean cordilleras, as well as the cloud forest and Paramos at high elevations, it can be expected that species richness of Geometridae in Colombia could comprise thousands of species, like in the neighbouring countries. However, the gaps in the taxonomic knowledge of Colombian geometrids have limited the sorting of material and species identification. Indeed, there are no taxonomic checklists, species inventories or catalogues available for Colombian Geometridae. Thus, a baseline of species records, based on modern sampling and DNA sequences, is urgently needed to document and estimate the real number of species.

With the integration of the DNA barcodes into species inventories, the task to uncover species richness has been made easier and DNA barcodes are helping to speed up species identifications, with a large amount of data currently available on platforms, such as BOLD. As a starting point, prior to this study, barcodes of 20 species of Colombian Geometridae have been publicly available (https://www.boldsystems.org/index.php/Public_BINSearch). To contribute to the knowledge of South American moths, this project has aimed to collect geometrid moths using light-traps from 26 localities from Colombia. It takes advantage of the efficiency of DNA barcodes for species sorting and identifications to increase the DNA dataset already available on BOLD and it strengthens the species inventories and taxonomic knowledge of Neotropical geometrids.

## General description

### Purpose

The primary purpose of the database is to provide a DNA barcode library and associated metadata of Geometridae. Geometrids are a globally-distributed family of moths (Lepidoptera) with high species richness in the Neotropics, but poorly studied in Colombia. This project is the result of four years of expeditions to 26 localities in the northeast of Colombia, aiming to collect geometrids and sequence their DNA barcodes, to record the number of species of geometrids and to make the taxonomic information, as well as distribution records, accessible. The barcode sequences will strengthen the data already available on BOLD and the new data will complement the morphology-based taxonomy and help to uncover the species richness of moths in the Neotropical Region. The availability of morphological and molecular data is a valuable resource for biodiversity studies and for understanding the distributions of Colombian geometrids. Released DNA barcodes provide a reference library for future sequences collected either in Colombia or elsewhere.

## Sampling methods

### Study extent

The sampling sites are located in: I) the Departments of Antioquia and Caldas in the central branch of the Andes, II) Boyacá and Santander in the centre and the eastern branch of the Andes, respectively, III) Serranía de Perijá Guajira, which is the furthest extension of the eastern branch of the Andes up north and IV) the Departments of Sucre and Córdoba, which belong to the Caribbean Region of Colombia (Table 1). Fieldwork was carried out from 2016 to 2019, mostly during the rainy season. A total of 26 sites were visited, which are located at different elevations.

### Sampling description

Depending on the environmental conditions at the sampling localities, light-traps were installed on forest edges or on walking trails. The traps included a mixed light bulb, powered by a portable gas generator and a LepiLED UV lamp (Brehm 2017) installed on a white sheet. Specimens were selectively collected from the sheets and euthanised with killing jars of Ethyl Acetate. The moon phase was considered an important factor for night sampling and full moons were avoided. After collecting, one to three legs were removed from each specimen for DNA barcoding. The vouchers were subsequently pinned, labelled and left to dry.

Specimens were provisionally assigned to putative species by using morphological characters, for example, wing patterns, abdominal tympanic organs, then followed by identifications through DNA barcodes using the tools available on BOLD, such as BIN (Ratnasingham and Hebert 2013). The BIN approach was chosen because BINs have been shown to have a high concordance with traditional taxonomic species concepts and can be used as a reliable proxy for species (Ortiz et al. 2017). The specimens are deposited at the Museo de Zoología, Universidad de Sucre, Colombia. DNA barcodes from vouchers and metadata are publicly available on BOLD. Records of species distributions were submitted and are publicly available on GBIF (https://www.gbif.org) and SIB Colombia (https://sibcolombia.net, https://doi.org/10.15472/ucfmkh) (Murillo-Ramos 2021).

*Molecular data*: The DNA extraction process was carried out using a NucleoSpin Tissue Kit (MACHEREY-NAGEL), following the manufacturer’s protocol. The DNA barcode region of the mitochondrial gene *cytochrome oxidase subunit I* (COI) was sequenced for all the samples. In addition, two protein-coding nuclear gene regions, *wingless* (Wnt) and *elongation factor 1 alpha* (EF-1alpha), were sequenced for the specimens that did not match with any record on BOLD, based on the DNA barcode. Specimens with three sequences were subject to Maximum Likelihood (ML) analyses as explained below.

DNA amplification and sequencing were carried out following protocols proposed by Wahlberg and Wheat (2008) and Wahlberg et al. (2016). PCR products were visualised on agarose gels. Successful PCR products were cleaned enzymatically with Exonuclease I and FastAP Thermosensitive Alkaline Phosphatase (ThermoFisher Scientific) and sent to Macrogen Europe (Amsterdam, The Netherlands) for Sanger sequencing. Additionally, some samples were sent to the Canadian Center for DNA Barcoding, University of Guelph, Canada, where DNA extraction, PCR amplification and sequencing were performed, following standard high-throughput protocols (deWaard et al. 2008).

### Quality control

Multiple sequence alignments were carried out in MAFFT as implemented in Geneious v.11.0.2 (Biomatters, http://www.geneious.com/). To check for possible errors in alignments and potential contamination, we constructed Neighbour Joining (NJ) and Maximum Likelihood (ML) trees. Successful DNA barcode sequences were uploaded and compared to those on BOLD (Ratnasingham and Hebert 2007), where sequences of more than 21,000 geometrid specimen BINs are available. In total, 386 Colombian Geometridae specimens were processed, but only 284 were successfully sequenced. We assesed species identification with sequences > 500 bp by the Barcode Index Number (BIN) system as implemented on BOLD (Ratnasingham and Hebert 2013).

Those COI sequences without a match on BOLD were submitted to a follow-up analysis with two additional nuclear genes. We retrieved the dataset of Murillo-Ramos et al. (2019) stored in VoSeq (Peña and Malm 2012). This dataset includes more than 1,000 taxa of Geometridae with good sampling of Neotropical species. We tried to obtain further identifications by merging our dataset and the sequences generated by Murillo-Ramos et al. (2019). We ran Maximum Likelihood analyses with partitions by gene using RAxML-HPC2 v.8.2.12 (Stamatakis 2014) on the web-server CIPRES Science Gateway (Miller et al. 2010). Support for nodes was evaluated with 1,000 rapid bootstraps in RAxML.

## Geographic coverage

### Description

**Figure 2.**
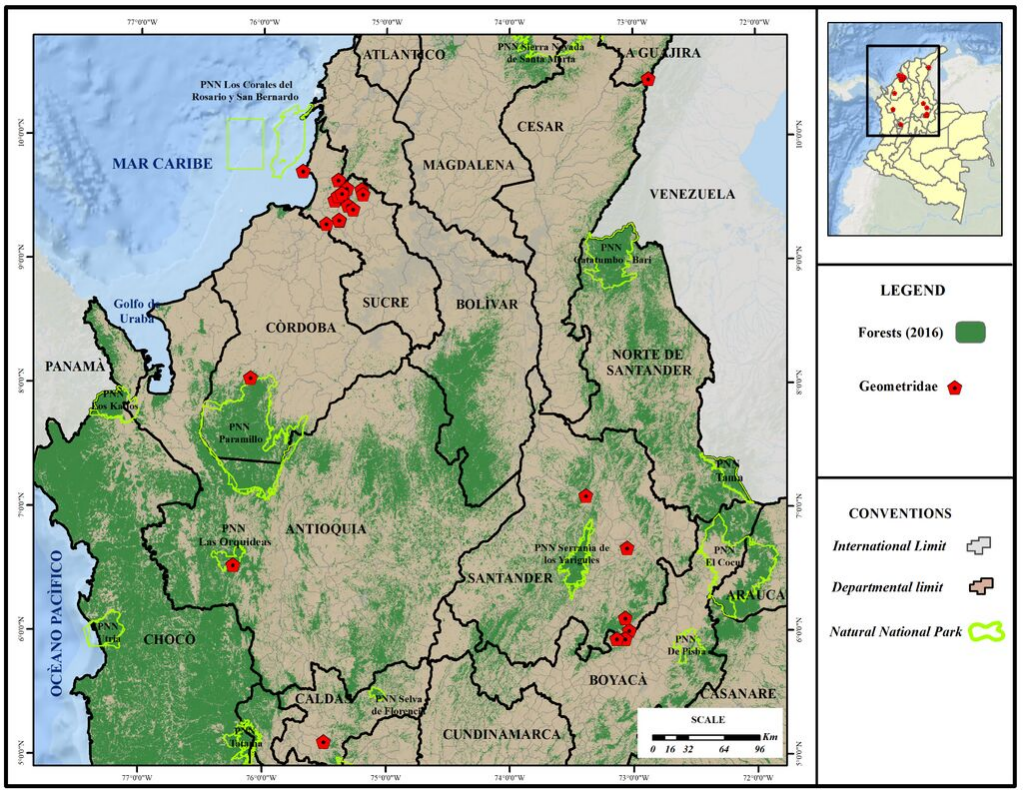
Distribution map of the localities sampled in this study.

The study sites are situated in two areas of Colombia: 1) the Caribbean Region and 2) the Andean Region (Fig. 2). Typical habitats in the Caribbean Region are dry and tropical rainforests and the sampling localities have elevations ranging from 0 to 600 m a.s.l. Typical habitats of localities sampled in Eastern and Central Andes are cloud forests and Paramo and the sampling sites have elevations ranging from 400 to 3800 m a.s.l. The Cerro Pintao site in the State of Guajira is covered by pre-montane forests, montane forests and paramo and the elevations of the sampling sites range from 2800 to 3000 m a.s.l.

## Taxonomic coverage

### Description

We report sequences of species belonging to the subfamilies Ennominae, Sterrhinae, Larentiinae and Geometrinae.

**Results**

We make available sequences of 281 specimens of Geometridae (dx.doi.org/10.5883/DS-GEOCO) of which 157 matched the BINs with previously-named species on BOLD (either from Colombia, but mostly from other countries, such as Ecuador; Suppl. material 1), while 115 sequences were assigned to BINs, which are identified only to genus or tribe level and more examinations are needed to reach species-level identifications. We checked (and modified accordingly) all existing identifications and provide further identifications, based on morphology and on the analysis of the two additional markers analysed using the Maximum Likelihood (ML) approach (Suppl. material 2). Identifications are provided in Suppl. materials 1, 3, 4. The current Colombian DNA barcode library contains sequences for species belonging to four out of eight subfamilies of Geometridae. Nearly 50% of the sequences are placed in the subfamily Ennominae, represented in our dataset by 159 BINs assigned to 55 genera (Suppl. material 3). Ennomines are very diverse in South America with more than 3,000 described Neotropical species (Pitkin 2002) and studies have suggested that Ennominae dominate particularly the lower elevational levels up to ca. 1,000 m (Brehm and Fiedler 2003). Although the estimation of elevational species richness gradients was beyond the scope of this study, one interesting pattern is that most of the Ennominae records correspond to the low elevation localities, which are mainly part of the Caribbean Region of Colombia, this Region being characterised by the presence of dry tropical forest.

Unsurprisingly, more than 50% of the barcodes which correspond to the subfamilies Sterrhinae and Larentiinae could not be identified at species level (Suppl. material 4), which reflects the lack of taxonomical revisions of those groups for South American species. Compared to available inventories in Ecuador and Costa Rica, the species list for Colombia is very far from being complete. Although the data release of this study is a significant contribution to the knowledge of Neotropical Geometridae, the current dataset stresses the gap in the knowledge of Colombian geometrids and it certainly represents only a small part of the fauna. Prior to the current study, only 99 public records were available on BOLD, which were assigned to 20 species. Unfortunately, there are no checklists of Colombian geometrids, although some species have been included in taxonomic works (Sullivan 2011, Brehm et al. 2011, Brehm 2018, Lindt et al. 2018). With the current dataset, we raise the number of sequences to 380 public records (including the previously-available data) of at least 177 species of Colombian geometrids (157 reported in this study).

**Discussion**

The occurrences, reported in this study, are based on specimens sampled in six Departments of Colombia, in which different habitat conditions prevail. Thus, the species composition of geometrids was very different in low elevation localities compared to the high-altitude sites and many species were narrowly distributed. Similar to the suggestion by Brehm and Fiedler (2003), the faunal composition of the geometrids found in this inventory seems to vary with regard to altitude, in which Sterrhinae, Geometrinae and Ennominae decrease in their proportions towards high elevations, while the opposite happens with Larentiinae. The species richness per locality is preliminary and it is clear that more studies are needed to understand the species distribution patterns and reveal the potential hotspot of geometrids in Colombia. Most probably the Andes host the highest diversity (Brehm et al. 2005, Brehm et al. 2016); however, far more sampling effort is required to consolidate the list. So far, the current species occurrences reported in databases from the GBIF portal contains 2,407 records of Geometridae in Colombia (GBIF Occurrence Download https://doi.org/10.15468/dl.b298x7). Those records have been provided from different collections of Colombian institutions and some observations from iNaturalist Research and the International Barcode of Life (iBOL) project. Of the 2,407 records for Geometridae in Colombia, only 453 records have been identified to species level (for a total of 131 species). Most records are identified only to the family (1,619 records), with some records to the genus level (335 records).

We provided further identifications of the specimens that were assigned to a BIN, but did not match with named species within Geometridae on the BOLD database. Based on the analysis of the two additional markers, using an ML approach and including the dataset retrieved from Murillo-Ramos et al. (2019), the phylogenetic tree confirmed the monophyly and taxonomic position of specimens in genera, such as *Synchlora*, *Iridopsis*, *Glena* and *Physocleora*. Even though it was not possible to reach species-level identification, the specimens clustered in their corresponding genera. In contrast, the results pointed out that genera like *Idaea*, *Scopula*, *Nephodia*, *Isochromodes* and *Macaria* require taxonomic revision, as they were recovered as being para- or polyphyletic. This could be explained by inadequate genetic information (three genes) or it could represent a true pattern phenomenon (poor taxonomy) or incomplete taxon sampling. We also found many independent lineages that, with more detailed studies, could possibly be assigned to undescribed genera within Boarmiini. Boarmiini is by far the most species-rich tribe-level clade of Ennominae, with ca. 200 genera and ca. 3,000 known species (Murillo-Ramos et al. 2021). However, the evolutionary relationships amongst boarmiines have been difficult to resolve, further complicated by numerous conflicting regional classifications. Boarmiini comprise many unidentified records in the Neotropical Region and this highlights the difficulties with working on species-rich groups that have not received much attention outside of Europe.

We could not assign more than 50% of the barcodes to species belonging to the subfamilies Sterrhinae and Larentiinae. Species in many genera, included in those subfamilies, are very difficult to identify, mostly due to the lack of taxonomic studies of Neotropical species. Most of the unassigned species correspond to the genera *Idaea* and *Eupithecia*, which are full of unidentified records, not only in this study, but also in public databases like BOLD. These two genera are amongst the species-rich radiations of Geometridae, widely distributed around the world and they are well-studied in the Palaearctic (Mironov 2003, Hausmann 2004), but poorly known in the Neotropics. *Idaea* comprises 669 species, while *Eupithecia* includes 1,360 described species (Hausmann 2004, Choi and Kim 2013, Mironov and Galsworthy 2014). The reasons behind their success have been attributed to the availability of suitable host plants, as well as to the ecological and morphological plasticity of the species (Brehm et al. 2005, Mironov and Galsworthy 2014). However, those hypotheses have not been tested, mainly because of insufficient biological information available for the vast majority of species in *Eupithecia* and *Idaea*. The latter prevents the analysis and interpretation of their diversification patterns. This is the same case for many lineages in Geometridae. Concerning the taxonomy and species number of those genera, our results underline the poor knowledge of Sterrhinae and Larentiinae in South America. There is no doubt that future taxonomic revisions will uncover many undescribed species and Neotropical taxa may, in fact, represent independent lineages from old-world genera.

Altogether, 157 geometrids identified in this study to the BIN-level are now barcoded from Colombia (dx.doi.org/10.5883/DS-GEOCO). This represents roughly 5% of the expected species richness of Colombia (1026 species have been reported from literature Scoble 1999, Scoble and Hausmann 2007 , while 2,407 records are reported in platforms like GBIF, https://doi.org/10.15468/dl.b298x7). This would neatly summarise the state of our knowledge and underlines the huge work that lies ahead before the fauna is well studied. The fact that we could only identify less than 50% of the specimens at species-level allows us to conclude that: I) a properly-curated DNA barcode reference library on BOLD is still a work in progress and it covers rather poorly the northern South American Geometridae fauna, II) there are not enough taxonomic experts on Colombian Geometridae, III) the available information for species identification is scarce and IV) more efforts are needed to develop our species list of Colombian Geometridae further towards a comprehensive checklist. Despite all the taxonomic uncertainties, we firmly believe that this barcode library will be a baseline reference for future research and will play an important role in monitoring and biodiversity studies. Although more inventories are needed to know more about the richness of Colombian geometrids, we also highlight that the type specimens described from Colombia should be barcoded in the future. This would make an important contribution to the database, by adding precision and giving Linnean names to barcodes already in the database and for new fresh material.

### Taxa included

**Table:** 

Rank	Scientific Name	Common Name
kingdom	Animalia	Animals
phylum	Arthropoda	
class	Insecta	Insects
order	Lepidoptera	
superfamily	Geometroidea	Moths
family	Geometridae	
subfamily	Sterrhinae	Waves moths
subfamily	Larentiinae	
subfamily	Geometrinae	Emerald moths
subfamily	Ennominae	

## Collection data

### Collection name

Museo de Zoología Universidad de Sucre, Colombia (MZUS)

### Collection identifier

Registro Nacional de Colecciones Biológicas: 231

### Parent collection identifier

Dried specimens, relaxed and mounted in entomological pins.

### Specimen preservation method

DNA voucher tubes (LMR-Geo001-386)

## Usage licence

### Usage licence

Creative Commons Public Domain Waiver (CC-Zero)

## Data resources

### Data package title

Colección de polillas (Lepidoptera) del Museo de Zoología de la universidad de Sucre.

### Number of data sets

1

### Data set 1.

#### Data set name

Collection of moths (Lepidoptera) of the Museum of Zoology of the University of Sucre

#### Number of columns

44

#### Download URL

https://www.gbif.org/dataset/9f462a42-8161-4759-9f0f-ffcc7857efec

#### Data format version

Darwin Core Archive, EML.

#### Description

A database of Colombian Geometridae occurrences.

**Data set 1.** 

Column label	Column description
Sample ID	Identifier for the sample being sequenced, often identical to the Field ID or Museum ID.
InstitutionID	Institution that has physical possession of the specimen
Collection ID	An identifier for the collection or dataset from which the record was derived
InstitutionCode	Code of the institution where samples are deposited
CollectionCode	The name, acronym, code or initialism identifying the collection or dataset from which the record was derived
BasisOfRecord	The specific nature of the data record
OcurrenceID	An identifier for the Occurrence
CatalogNumber	An identifier for the record within the dataset or collection
RecordedBy	The primary collector or observer, especially one who applies a personal identifier
IndividualCount	The number of individuals represented at the of the occurrence
Preparations	A list of preparations and preservation methods for a specimen
Sampling protocol	The name or reference of the method used during a event
EventDate	The date-time during an event occurred
Year	The four-digit year in which the event occurred
Month	The integer month in which the event occurred
Day	The integer day in which the event occurred
Habitat	A category or description of the habitat in which the event occurred
Continent	The name of the continent in which the location occurs
CountryCode	The standard code for the country in which the location occurs
StateProvince	The name of the next smaller administrative region than country in which the location occurs
County	The full, unabbreviated name of the next smaller administrative region than stateProvince in which the location occurs
Municipality	The specific description of the place
Locality	The specific description of the place
VerbatimLocality	The original textual description of the place
VerbatimElevation	The original description of the elevation of the location
VerbatimLatitude	The verbatim original latitude of the location
VerbatimLongitude	The verbatim original longitude of the location
VerbatimCoordinateSystem	The coordinate format for the verbatimLatitude and verbatimLongitude or the verbatimCoordinates of the Location
VerbarimSRS	The ellipsoid, geodetic datum or spatial reference system (SRS) upon which coordinates given in verbatimLatitude and verbatimLongitude or verbatimCoordinates are based
DecimalLatitude	The geographic latitude of the geographic centre of a Location
DecimalLongitude	The geographic longitude of the geographic centre of a Location
GeodeticDatum	The ellipsoid, geodetic datum or spatial reference system (SRS) upon which the geographic coordinates given in decimalLatitude and decimalLongitude are based
IdentifiedBy	A list of names of people, groups or organisation who assigned the taxon to the subject
DateIdentified	The date on which the subject was determined as representing the Taxon
IdentificationRemarks	Comments or notes about the identification
IdentificationQualifier	A brief phrase or a standard term to express the determiner's doubts about the identification
ScientificName	An identifier for the nomenclatural detail of a scientific name
Kingdom	The full name of the kingdom in which the taxon is classified
Phyllum	The full scientific name of the phylum or division in which the taxon is classified
Class	The full scientific name of the class in which the taxon is classified
Order	The full scientific name of the order in which the taxon is classified
Family	The full scientific name of the family in which the taxon is classified
Genus	The full scientific name of genus in which the taxon is classified
specificEpithet	The full scientific name of species in which the taxon is classified

## Supplementary Material

7A2DDE2C-788E-5B32-A980-51625C21908A 10.3897/BDJ.9.e68693.suppl1 Supplementary material 1 List of Geometridae species successfully barcoded and assigned to BINs Data type Occurrences and vouchers. Brief description List of Geometridae species successfully barcoded and assigned to BINs through the analytical tools of BOLD (Ratnasingham and Hebert 2007). File: oo_541268.txt https://binary.pensoft.net/file/541268 Murillo-Ramos L, Sihvonen P, Brehm G, Ríos Malaver I, Wahlberg N.

29654CED-C0E8-5015-B0F2-0453E6584F6F 10.3897/BDJ.9.e68693.suppl2 Supplementary material 2 Maximum Likelihood tree (ML) of Colombian geometrids including COI, Wingless and EF1a markers Data type Phylogenetic data. Brief description Maximum Likelihood tree (ML) of Colombian geometrids including COI, Wingless and EF1a markers analysed together with a dataset retrieved from Murillo-Ramos et al. (2019). Specimens in red correspond to the sequences generated in this study. File: oo_541144.pdf https://binary.pensoft.net/file/541144 Murillo-Ramos L, Sihvonen P, Brehm G, Ríos Malaver I, Wahlberg N.

9C394C45-5BBA-57E4-90DA-76D9B86BE109 10.3897/BDJ.9.e68693.suppl3 Supplementary material 3 Illustrated catalogue of all new records of Colombian Geometridae, part 1 Data type Photos of vouchers. Brief description Illustrated catalogue of all new records of Colombian Geometridae, in two parts: Ennominae File: oo_541151.pdf https://binary.pensoft.net/file/541151 Murillo-Ramos L, Sihvonen P, Brehm G, Ríos Malaver I, Wahlberg N.

627FD551-CD96-5A50-A7E9-05BD83B8F27E 10.3897/BDJ.9.e68693.suppl4 Supplementary material 4 Illustrated catalogue of all new records of Colombian Geometridae, part 2 Data type Photos of vouchers. Brief description Illustrated catalogue of all new records of Colombian Geometridae, in two parts: Other subfamilies. File: oo_541147.pdf https://binary.pensoft.net/file/541147 Murillo-Ramos L, Sihvonen P, Brehm G, Ríos Malaver I, Wahlberg N. Data type: Photos of vouchers.

## Figures and Tables

**Table 1.** Sampling localities with coordinates and elevation.

State	Municipality	Locality	Longitude / Latitude	Elevation (m a.s.l.)
Antioquia	Urrao	Cañón de río calles, en borde de bosque subandino, de camino de herradura	06°31'46.65"N, 76°14'65"W	1466
Boyacá	Duitama	Vereda el Carmen, Páramo de la Rusia	05°56'28.3"N, 73°04'35.2"W	3800
Boyacá	Duitama	Vereda el Carmen, finca Villa Kathy	05°57'04.6"N, 73°08'43.3"W	2982
Boyacá	Duitama	Vereda Avendaños II, quebrada el Papayo	06°00'30.7"N, 73°02'14.0"W	2757
Caldas	Manizales	Vereda el Águila, quebrada la Caracola	05°06'480"N, 75°30'566"W	1650
Córdoba	Tierralta	Vereda Tuis-Tuis, finca el Tuti fruti	08°02'23.7"N, 76°05'59.6"W	129
Guajira	Urumita	Cerro Pintao, Serranía de Perijá	10°27'.36.4"N, 72°52'11.1"W	2844
Santander	Encino	Vereda la Cabuya, sitio la Variante	06°06'44.1"N, 73°04' 29.6"W	1872
Santander	Aratoca	Vereda la laja, san Ignacio, finca La Esmeralda	06°40'21.2"N, 73°03'30.7"W	1731
Santander	Girón	Quebrada la Triguereña, vía Barranca/Bucaramanga	07°05'25.88"N, 73°23'02.01"W	473
Sucre	Colosó	Vereda Pajarito	09°31'58.2"N, 75°21'54.8"W	193
Sucre	Sincelejo (San Antonio)	Finca La Pastora	09°17'15.6"N, 75°29'19.3"W	68
Sucre	San Antonio de Palmito	Finca La Gloria	09°18'48.3"N, 75°23'03.8"W	78
Sucre	Sincelejo	Universidad de Sucre (Puerta roja)	09°19'03.87"N, 75°23'11.50"W	187
Sucre	Morroa	Finca El Socorro	09°24'23.4"N, 75°16'22.6"W	193
Sucre	Morroa	Finca El Oriente	09°26'06.2"N, 75°18'49.2"W	138
Sucre	Tolú Viejo	Finca La Gaviota	09°28'37.19"N, 75°25'21.53"W	161
Sucre	Colosó	Vereda Paraíso	09°29'27.1"N, 75°23'09.8"W	170
Sucre	Tolú Viejo	Roca Madre	09°30'44.90"N, 75°23'41.21"W	135
Sucre	Ovejas	Finca El Socorro	09°31'29.8"N, 75°11'32.7"W	215
Sucre	Colosó	Estación Primates	09°31'53.39"N, 75°20'55.52"W	226
Sucre	Ovejas	Finca El Principio	09°33'44.4"N, 75°12'07.5"W	216
Sucre	Chalán	Finca La División	09°34'26.71"N, 75°19'28.54"W	600
Sucre	Aguacate	Finca Catatumbo	09°38'28.8"N, 75°23'30.0"W	65
Sucre	San Onofre	Reserva San Guaré	09°42'44.2"N, 75°40'47.5"W	8
