# Supplementary material for: A database and checklist of geometrid moths (Lepidoptera) from Colombia
Source: Biodivers Data J. 2021 Sep 3;9:e68693. doi: 10.3897/BDJ.9.e68693 (PMC8433126; doi:10.3897/BDJ.9.e68693)

Colombia Geometridae ENNOMINAE identification catalogue

State 1 April 2021
Identified using the NHM collection and USNM images
alphabetical order

Legend

Species identification
nr = near
group = this species or very simillar

Type locality

Further specimens from Colombia

Specimen(s) barcoded

Isochromodes sabularia Dognin (TL: Ecuador, San-Francisco near Loja)

BC
BOLD:AAC2727
OTU-188

Barcoded specimens from other regions
with same BIN
or closely related
(indicated)

Additional compared specimen
= Ec-Geo-19097|Ecuador|Zamora Chinchipe

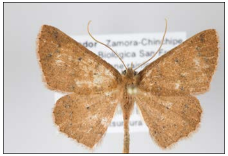

LMR-Geo-

Reference specimen
ideally type specimen
or no-type identified in NHM
(Natural History Museum
London)
or USNM
or ZSM (Herbulot)

Compared specimen:
USNM photo

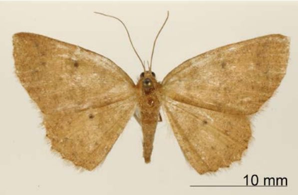

LMR-Geo-
0222

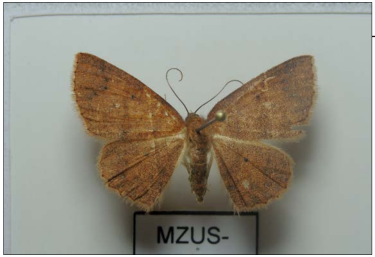

Barcoded specimen from Colombia

2

Acrosemia vulpecularia Herrich-Schäffer (TL: Brazil)

BC

BOLD:AAB0440

OTU-59

Additional compared specimen

LMR-Geo-

Compared specimen:

NHM type of synonym

LMR-Geo-

0377

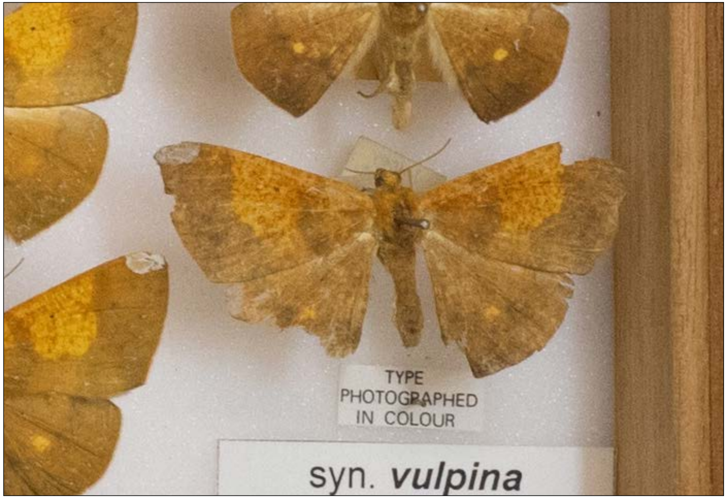

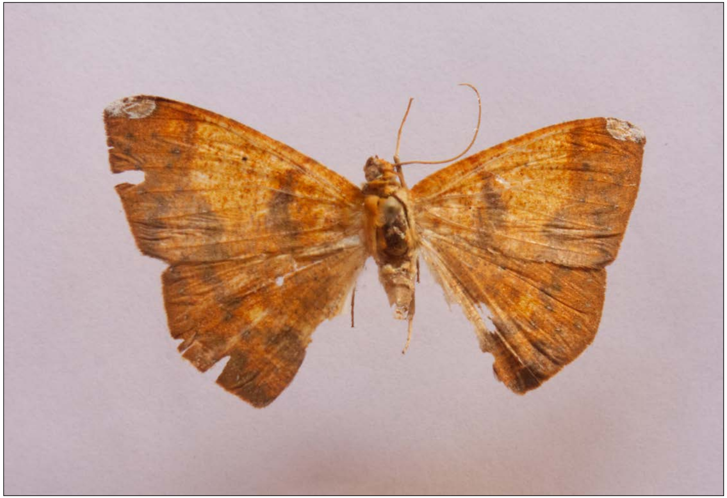

Acrotomia viminaria Herrich-Schäffer (TL: Mexico)

BC
BOLD:AAA6749
OTU-127

Additional compared specimen

LMR-Geo-
0101 (no photo)

Compared specimen:
Pitkin 2002 illustration

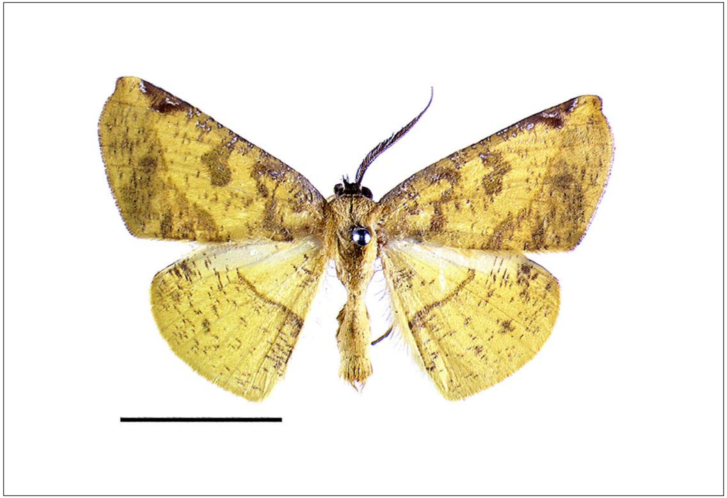

LMR-Geo-
0103

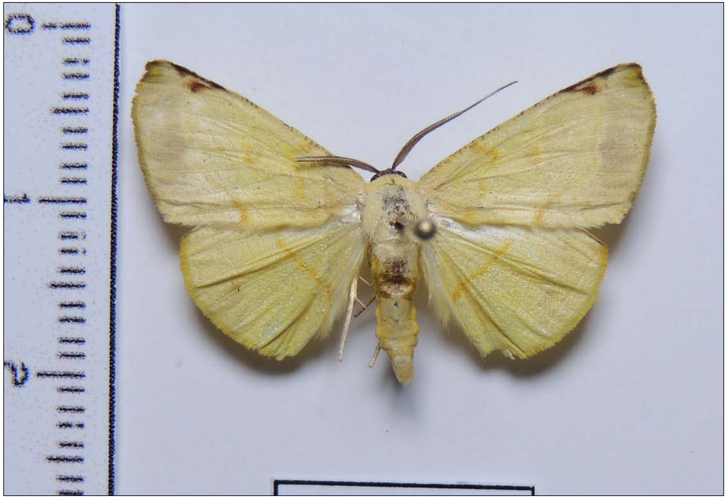

Anavitrinella sp (TL:)

BC

BOLD:AEE2274

OTU-121

Additional compared specimen

LMR-Geo-

Compared specimen:

LMR-Geo-

0108

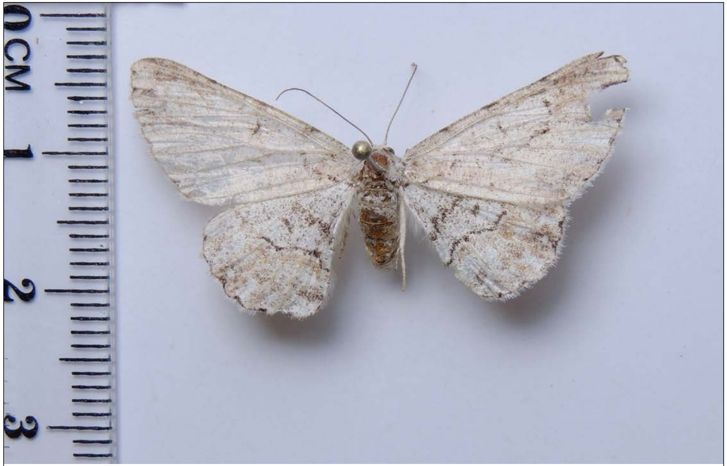

Aplogompha chotaria Schaus (TL: Mexico: Jalapa; Venezuela, Aroa)

BC

BOLD:AAA0607

OTU-32

Additional compared specimen

near Pe-Geo-0988|Peru|Huanuco|BOLD:ADF7110

LMR-Geo-

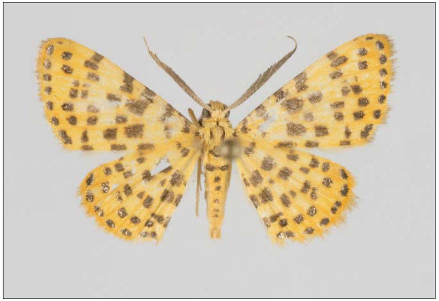

Compared specimen:

USNM type

LMR-Geo-

0343

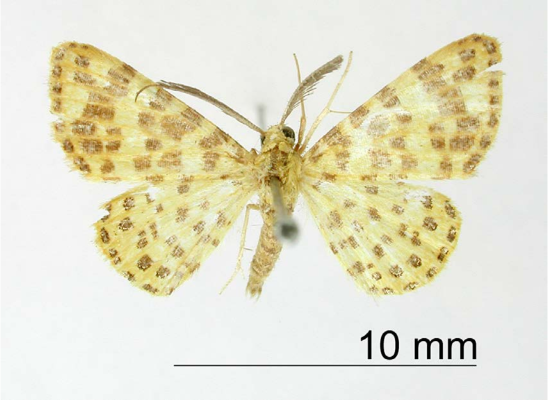

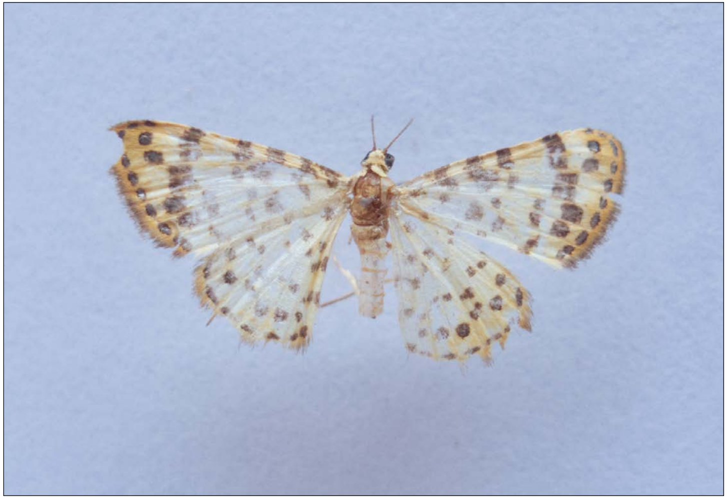

Aplogompha nr chotaria Schaus (TL: Mexico: Jalapa; Venezuela, Aroa)

BC
BOLD:AEE5389
OTU-124

Additional compared specimen
near Pe-Geo-0988|Peru|Huanuco|BOLD:ADF7110

LMR-Geo-

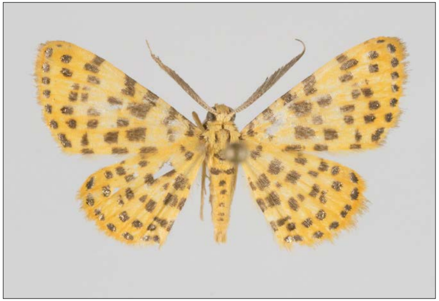

Compared specimen:
USNM type

LMR-Geo-
0105

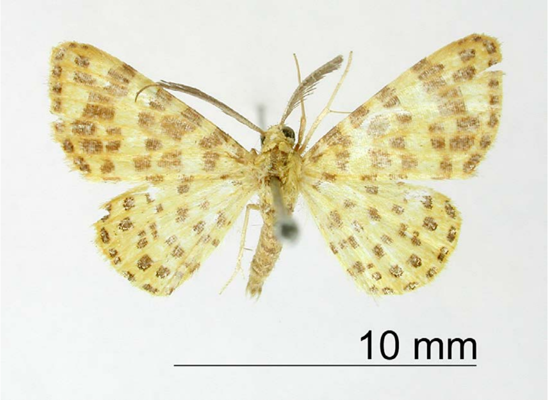

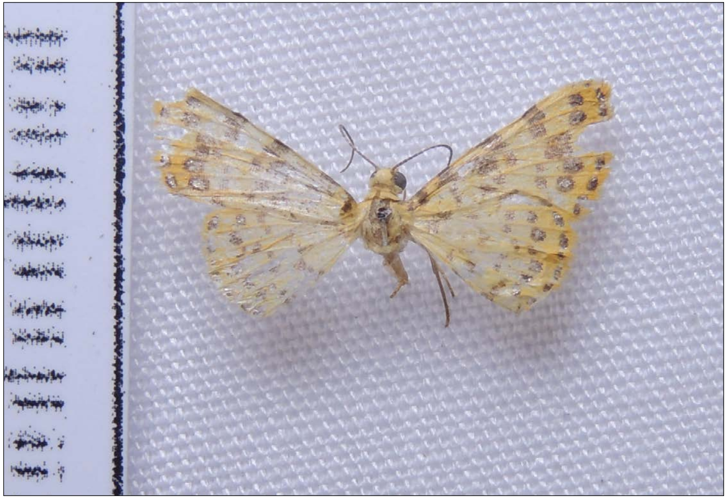

Argyrotome prospectata group Snellen (TL: [Colombia)

BC

BOLD:AAN2084

OTU-75

Additional compared specimen

LMR-Geo-
0028

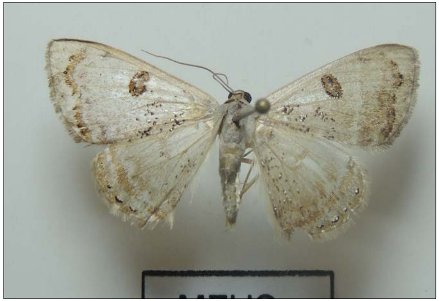

Compared specimen:
NHM no type

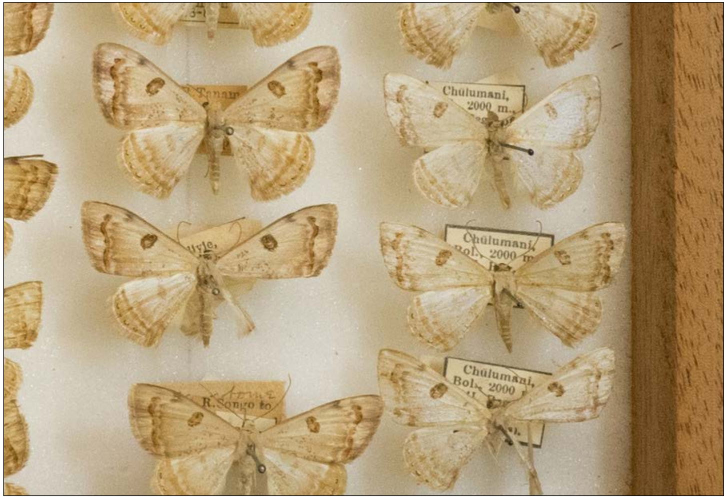

LMR-Geo-
0027

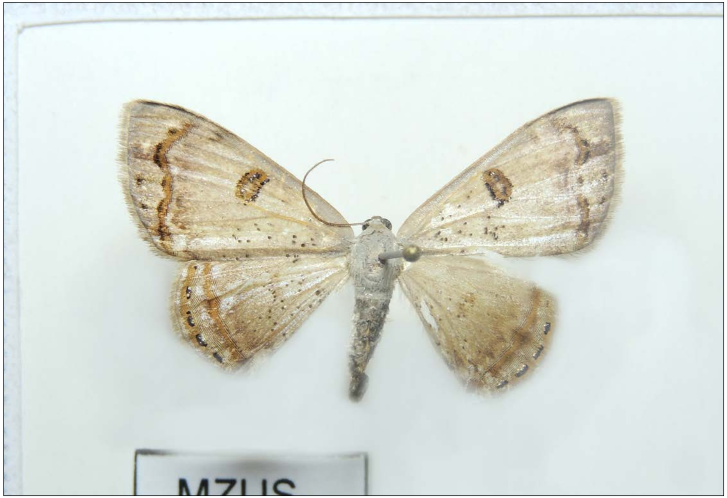

"Bassania" crocallinaria Oberthür (TL Peru: Tambillo)

BC

BOLD:AAD2301
OTU-79

Additional compared specimen
= Ec-Geo-22420|Ecuador|Zamora Chinchipe|BOLD:AAD2301

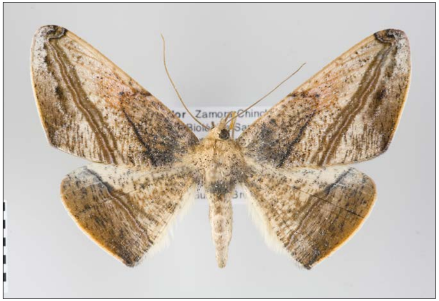

LMR-Geo-

Compared specimen:
NHM type

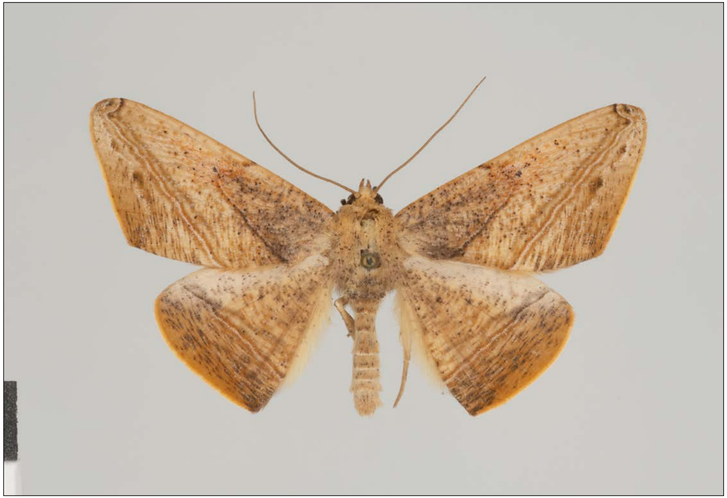

LMR-Geo-
0023

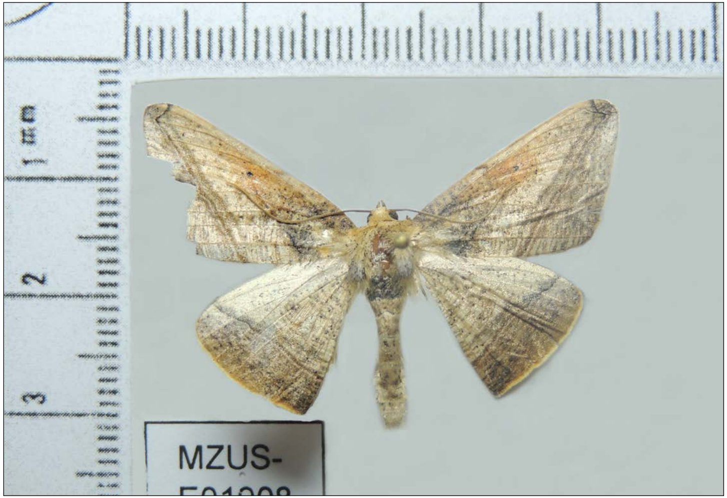

Argyrotome nr melae Druce (TL: Guatemala: Panama: Bugaba; Volcan de Chiriqui, 2000-3000 ft; Ecuador)

BC

BOLD:AAB2441	
OTU-115	

Additional compared specimen

LMR-Geo-

Compared specimen:

NHM type

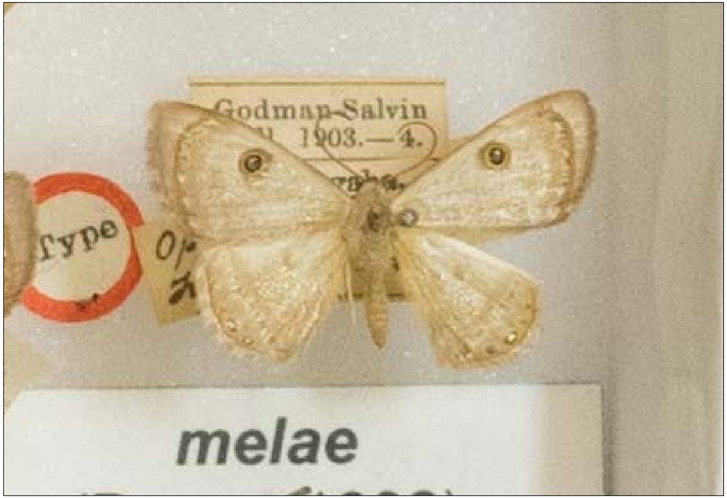

LMR-Geo-

0081

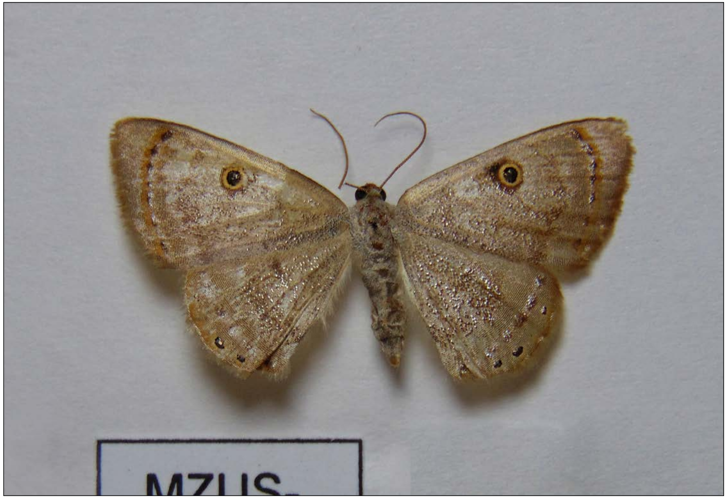

Brachyctenistis incongruata Warren (TL: Colombia]: Bogotá)

BC

BOLD:AEE3159

OTU-79

Additional compared specimen

near Ec-Geo-11082|Ecuador|Zamora Chinchipe|BOLD:AAP2005

LMR-Geo-

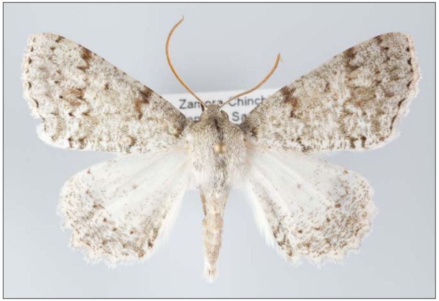

Compared specimen:

NHM type

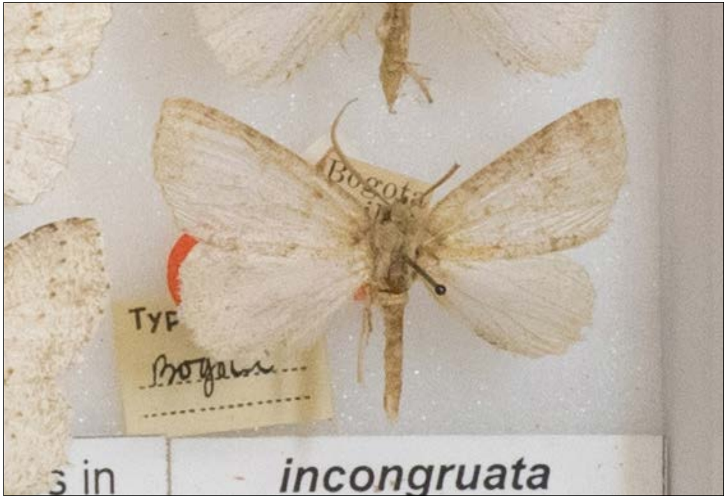

LMR-Geo-

0212

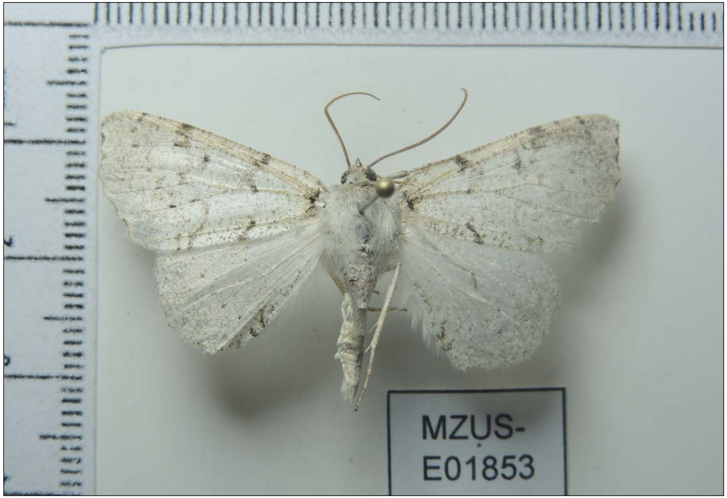

Bryoptera nr friaria Schaus (TL: Costa Rica: Poas; Turrialba)

BC

BOLD:AEE4000

OTU-77

Additional compared specimen

near Ec-Geo-19392|Ecuador|Zamora Chinchipe|BOLD:AAI3843

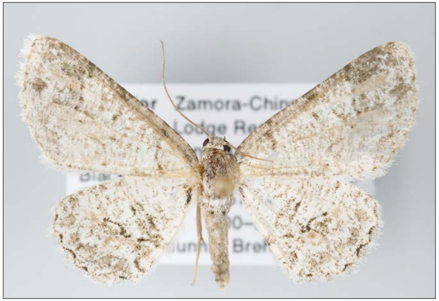

LMR-Geo-

Compared specimen:

USNM type

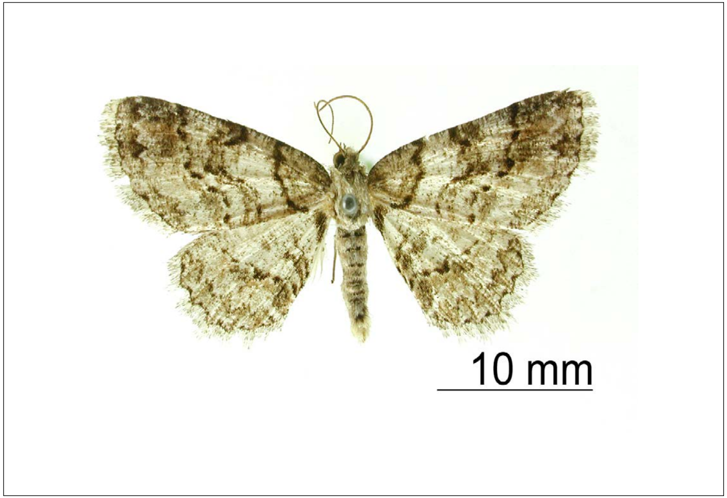

LMR-Geo-

0021

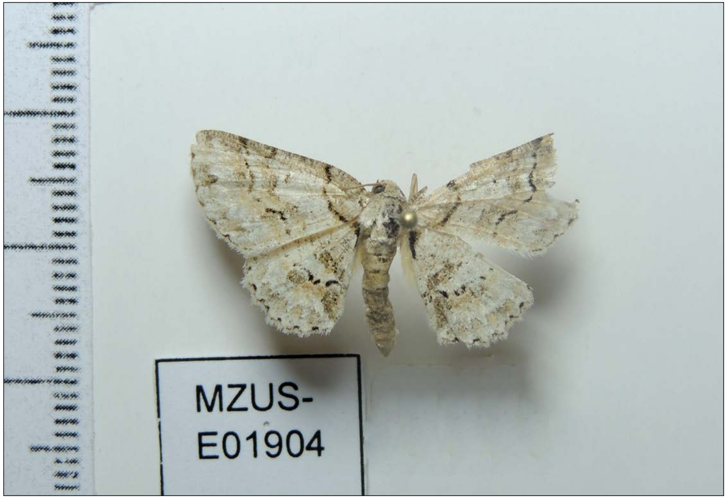

Cimicodes sp (TL:)

BC

BOLD:AEB9599
OTU-49

Additional compared specimen
near Pe-Geo-0596|Peru|Cuzco|BOLD:ADF1909

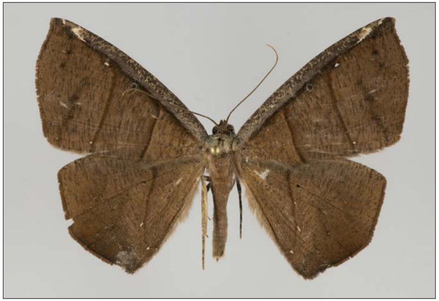

LMR-Geo-
0376, 0291

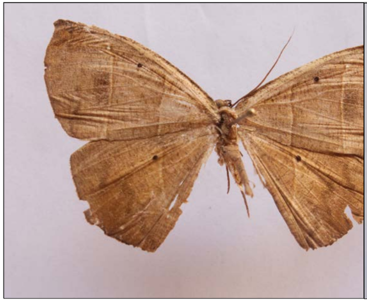

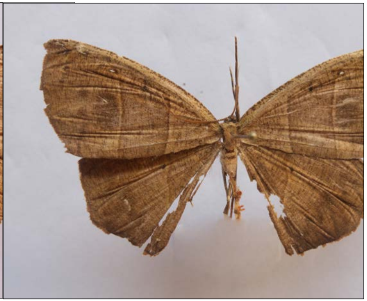

Compared specimen:

LMR-Geo-
0291

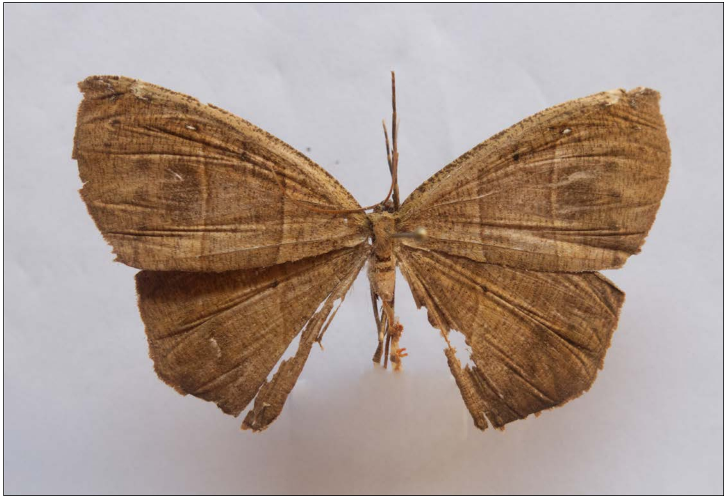

Cimicodes albicosta Dognin (TL: Panama: Lino, 800 m)

BC

BOLD:AAL6144	
OTU-37	

Additional compared specimen
= Pe-Geo-3868|Peru|CuzcoBOLD:AAL6144

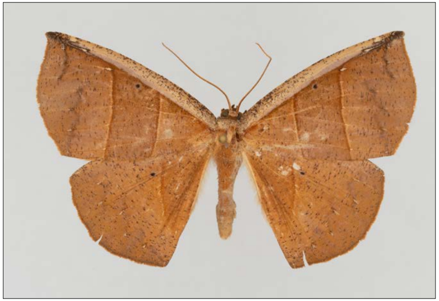

LMR-Geo-

Compared specimen:

USNM type

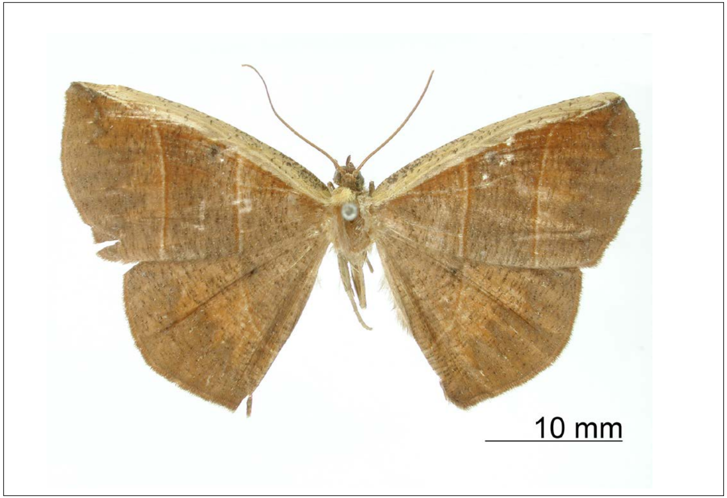

LMR-Geo-

0350

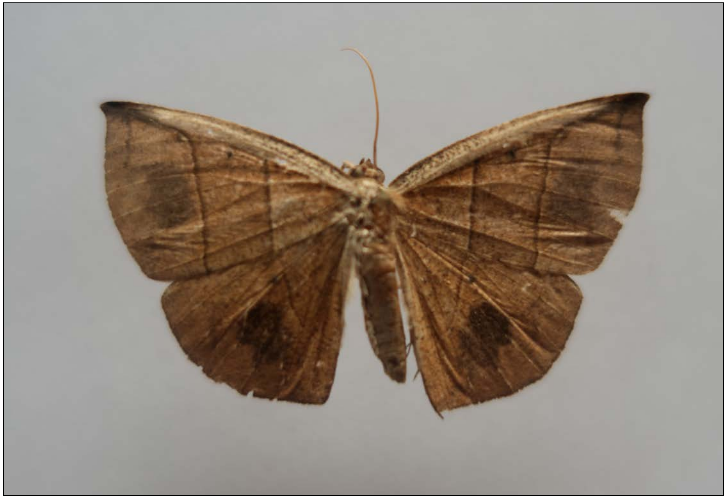

Cirsodes sp (TL:)

BC

BOLD:AEE6294
OTU-164

Additional compared specimen

LMR-Geo-

Compared specimen:

LMR-Geo-
0214

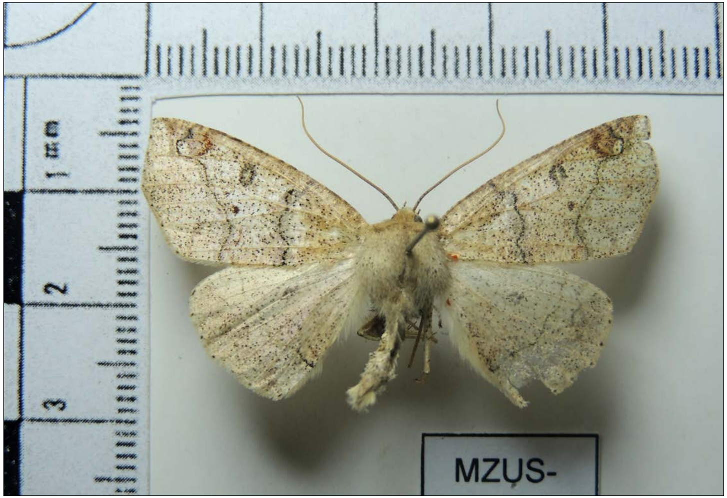

Cnephora nr cocapata cana Dognin (TL: Ecuador: Loja)

BC

BOLD:ADV3958

OTU-200

Additional compared specimen
near *cocapata cana* 22403|Ecuador|Loja|BOLD:AAW1355

LMR-Geo-

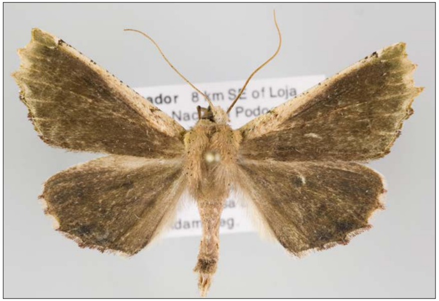

Compared specimen:
USNM type

LMR-Geo-
0247

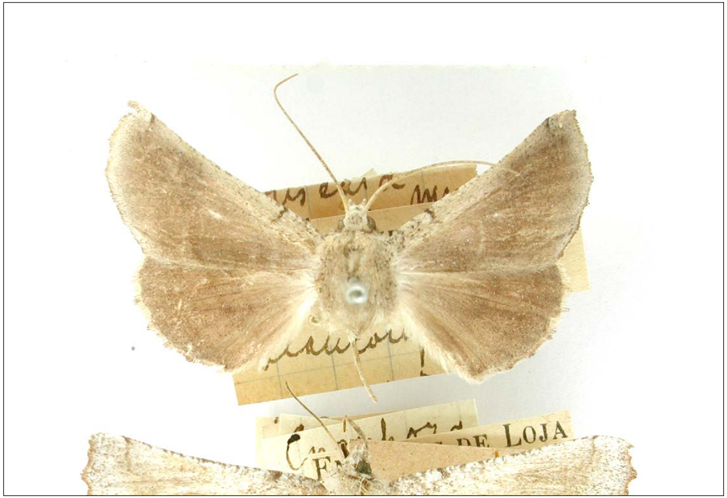

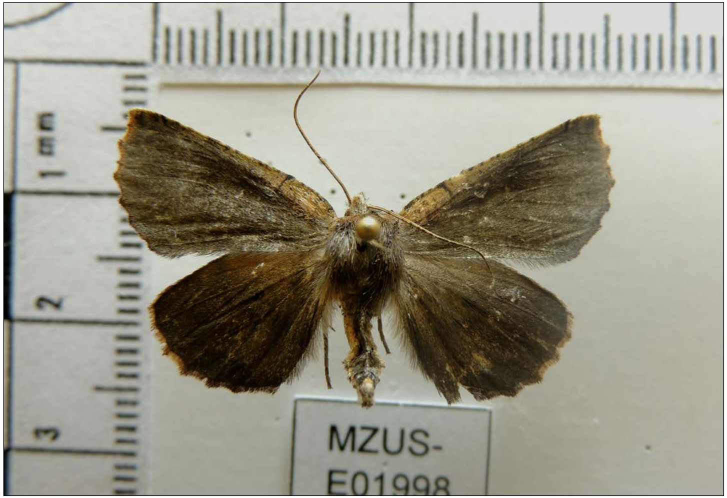

Digrammia sp (TL:)

BC

BOLD:AEE1708	
OTU-112	

Additional compared specimen

LMR-Geo-

Compared specimen:

LMR-Geo-

0100

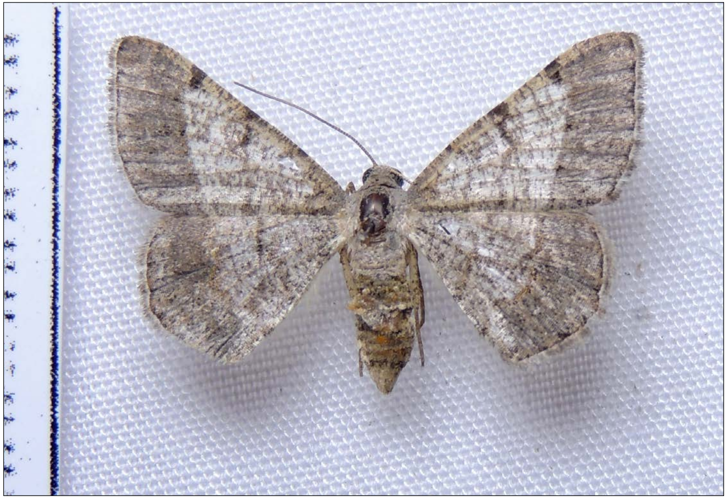

Epimecis nr plumbilinea Warren (TL: Peru, (south-east): Carabaya, Santo Domingo, 6000 ft)

BC

BOLD:AAA6719
OTU-222

Additional compared specimen
= Pe-Geo-1341|Peru|Huanuco|BOLD:AAA6719

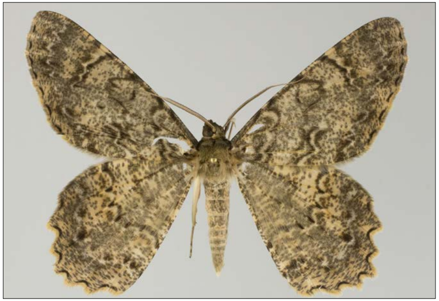

LMR-Geo-

Compared specimen:
NHM type

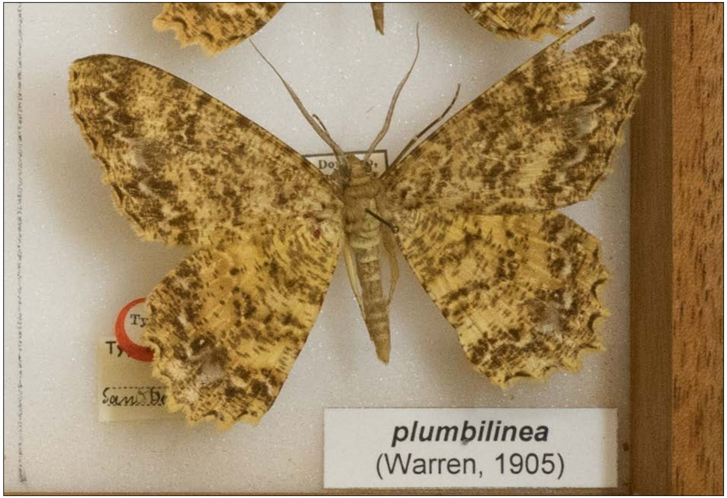

LMR-Geo-
0270

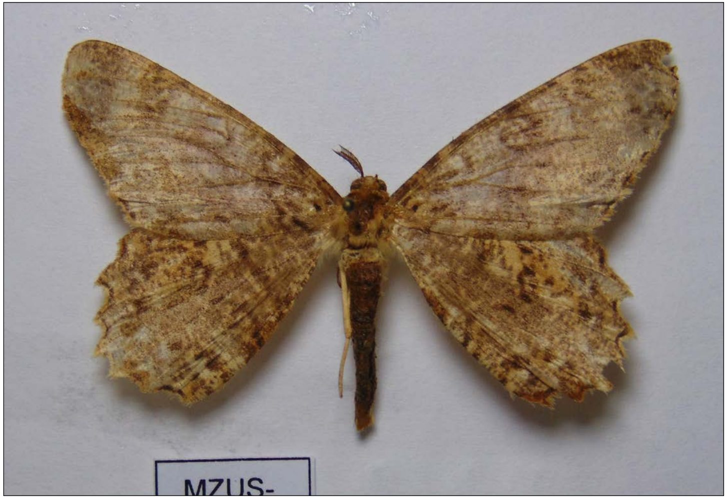

Erastria decrepitaria Hübner (TL: Brazil: Bahia)

BC

BOLD:AAB6014
OTU-125

Additional compared specimen

LMR-Geo-

Compared specimen:

NHM type of synonym

LMR-Geo-

0104

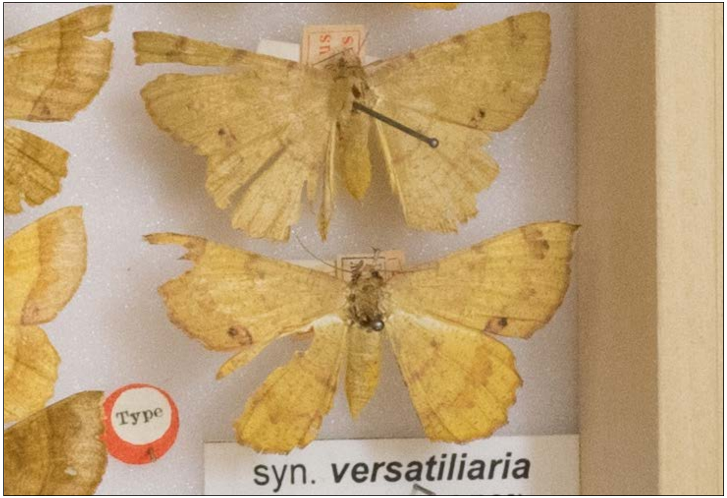

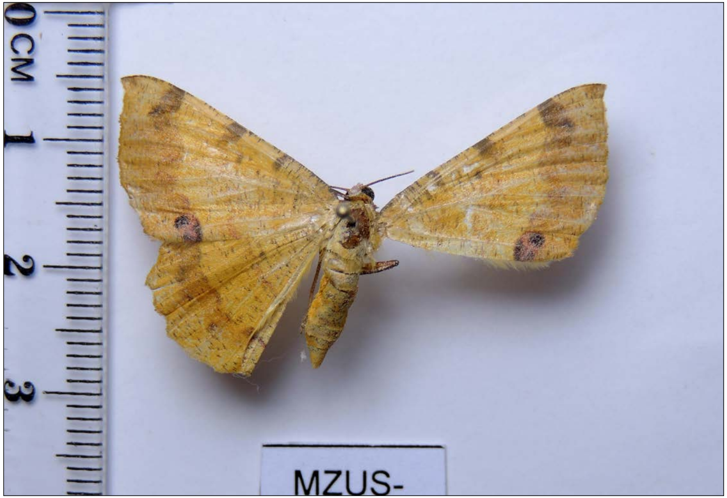

Eusarca sp (TL:)

BC

BOLD:AEE3815
OTU-123

Additional compared specimen
near Pe-Geo-1206|Peru|Huanuco|BOLD:ADG5013

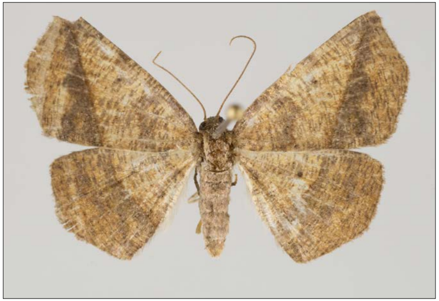

LMR-Geo-

Compared specimen:

LMR-Geo-
0106

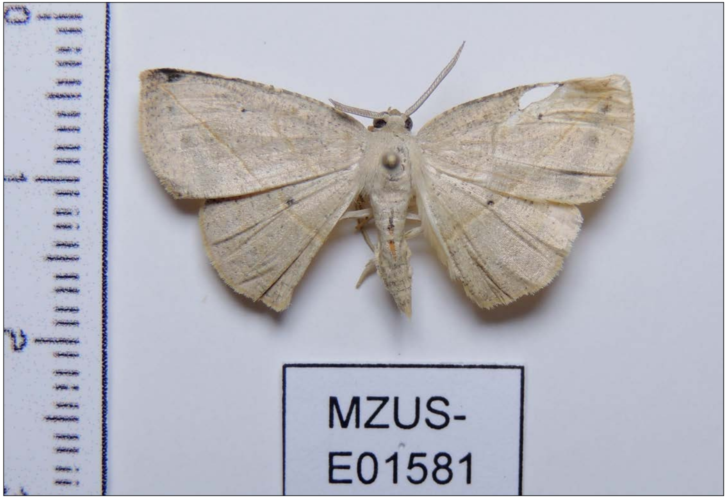

Eusarca nr cayennaria Guenée (TL: [French Guiana]: Cayenne)

BC

BOLD:ADG5013
OTU-143

Additional compared specimen

LMR-Geo-

Compared specimen:

NHM type

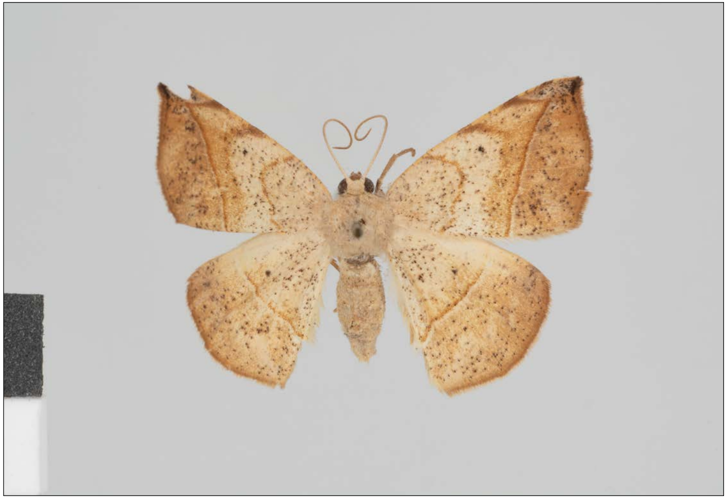

LMR-Geo-

0127

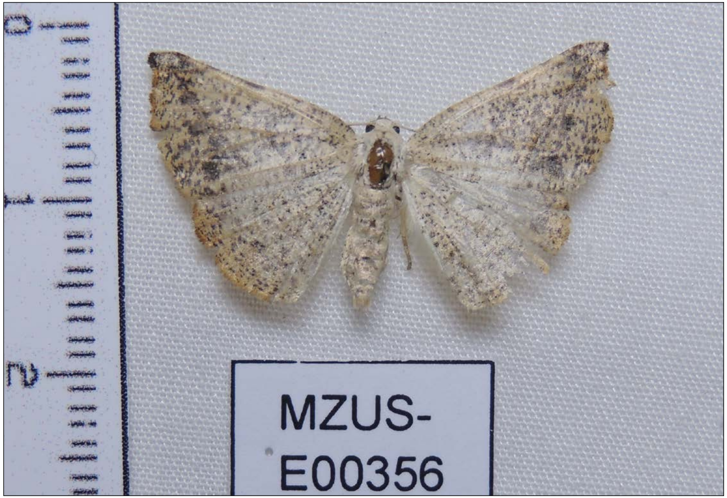

Eutomopepla nr rogenhoferi Oberthür (TL: Peru: ambillo; Huambo)

BC

BOLD:AEE0550
OTU-106

Additional compared specimen

LMR-Geo-

Compared specimen:

NHM type

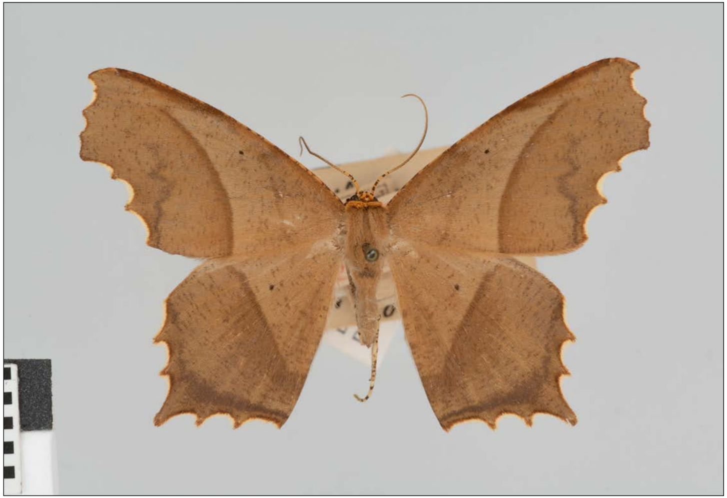

LMR-Geo-

0068

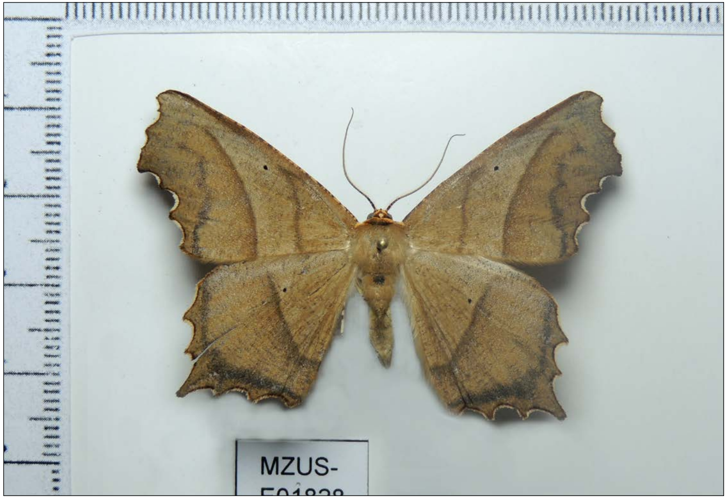

Eutomopepla nr rogenhoferi Oberthür (TL: Peru: ambillo; Huambo)

BC
BOLD:AEC0218
OTU-5

Additional compared specimen

LMR-Geo-

Compared specimen:

NHM type

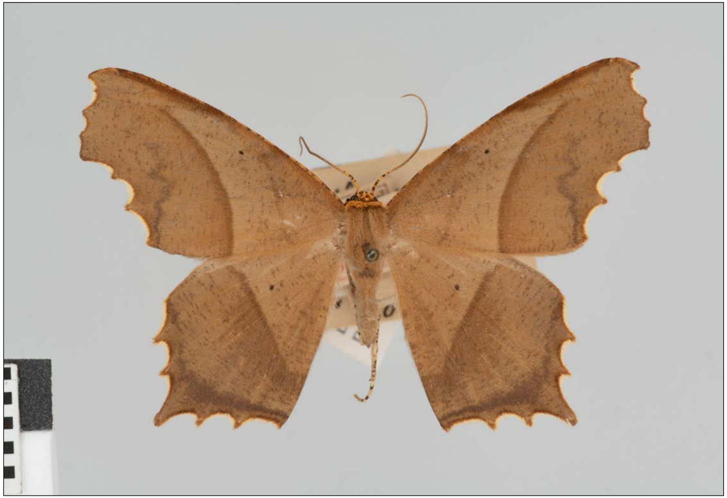

LMR-Geo-

0308

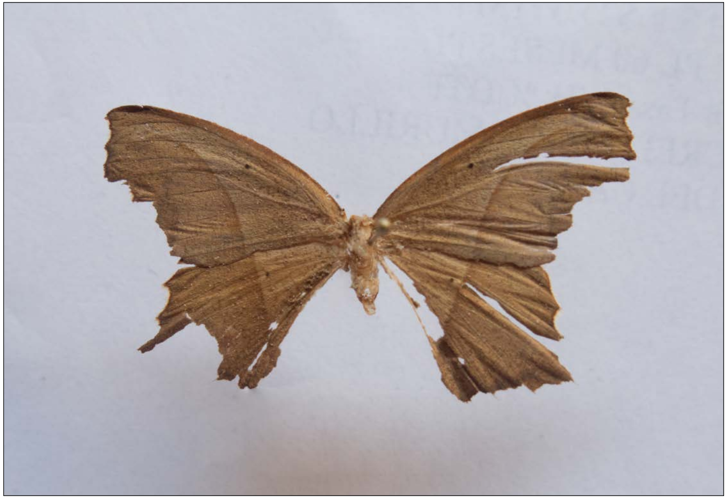

Glena sp (TL: Brazil)

BC

BOLD:ACH1247	
OTU-122	

Additional compared specimen

LMR-Geo-

Compared specimen:

LMR-Geo-

0107

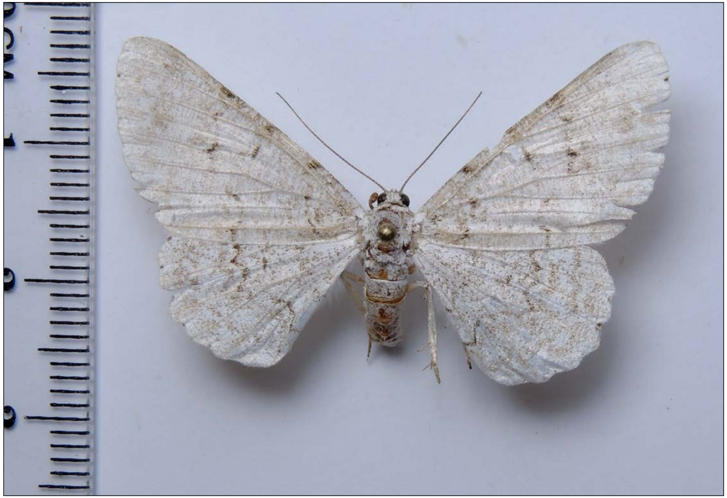

Glena nr bisulca Rindge (TL: Ecuador, [Napo-Pastaza], Rio Topo, Hacienda la Mascota, 4500 ft)

BC

BOLD:ACH1247
OTU-50

Additional compared specimen

LMR-Geo-

Compared specimen:

NHM paratype

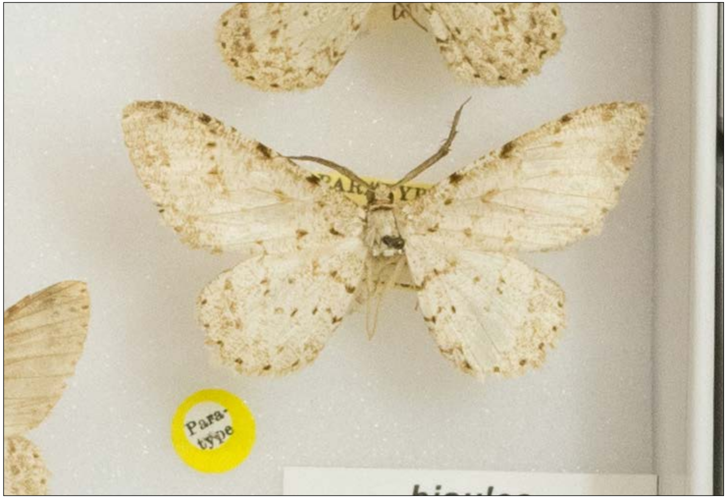

LMR-Geo-

0366

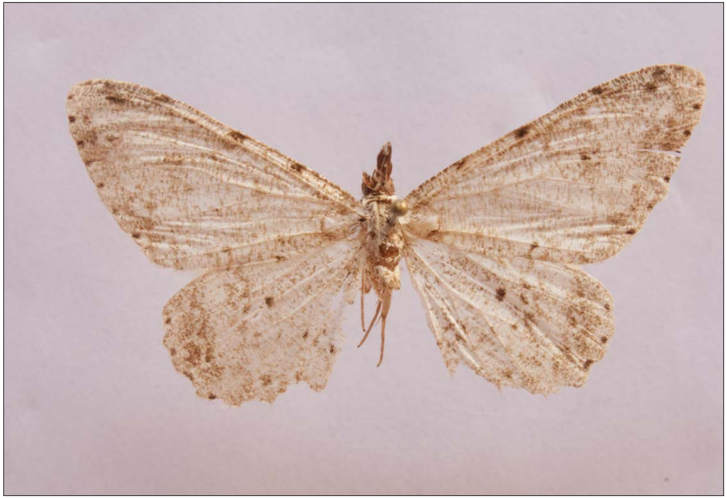

Gonora hyelosioides Walker (TL: [Colombia]: Bogota)

BC

BOLD:AAH0697	
OTU-3	

Additional compared specimen
= Ec-Geo-16916|Ecuador|Zamora Chinchipe|BOLD:AAH0697

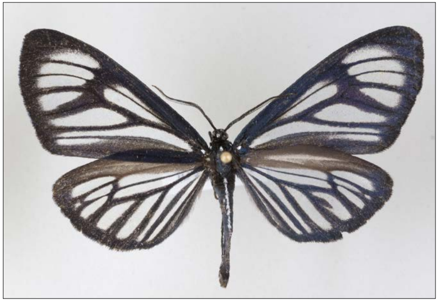

LMR-Geo-

Compared specimen:
NHM type of synonym

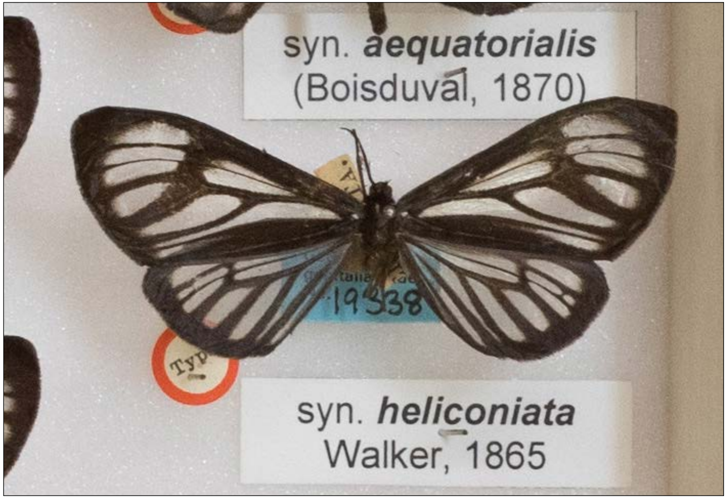

LMR-Geo-
0304

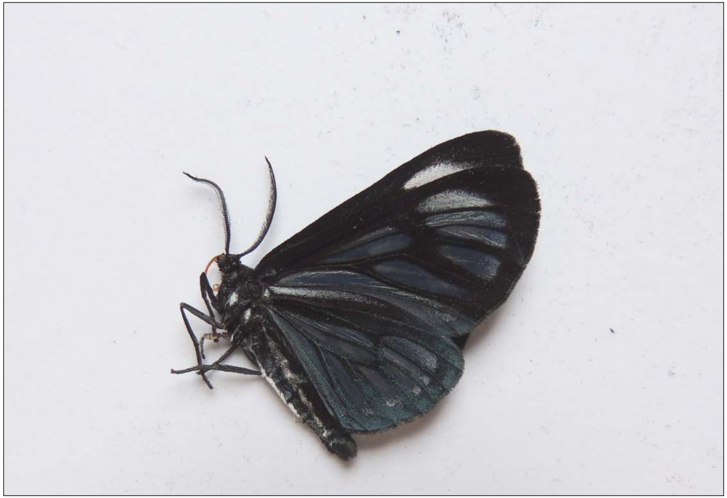

Hemixera nr orthosiodes Warren (TL: (Peru, south-east): Carabaya, Santo Domingo, 6000 ft)

BC

BOLD:AEE9120
OTU-102

Additional compared specimen
near Ec-Geo-22322|Ecuador|Loja|BOLD:AAM6908 #169

LMR-Geo-

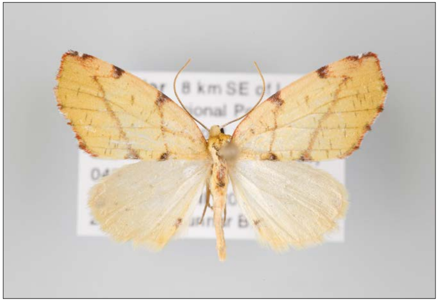

Compared specimen:

NHM type of synonym

LMR-Geo-

0050

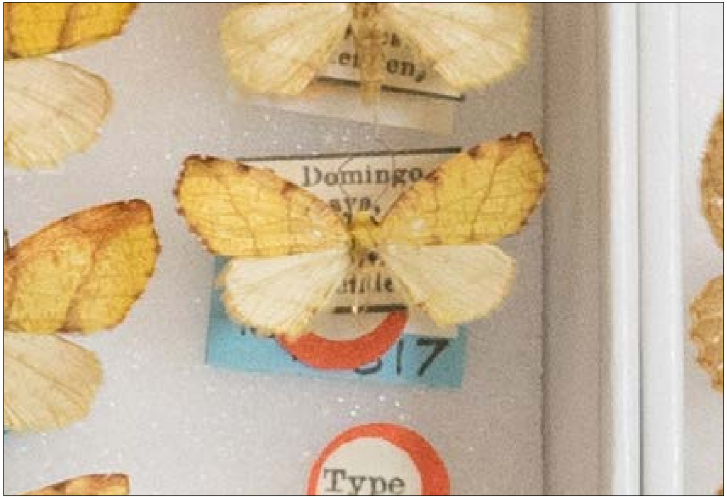

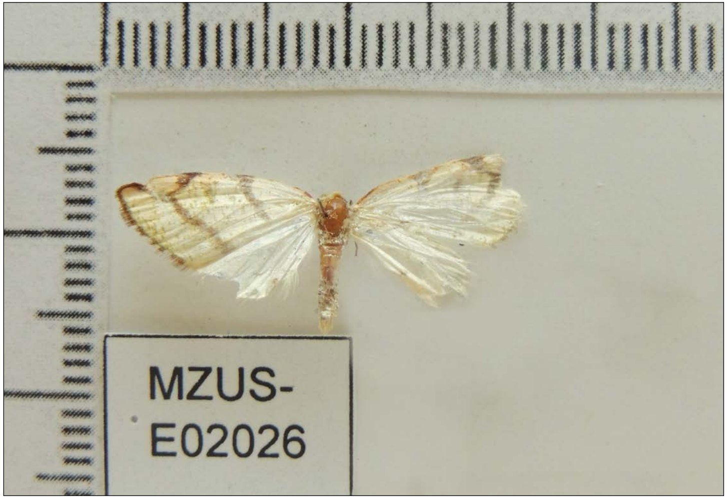

Herbita sp (TL:)

BC

BOLD:AEB9190

OTU-52

Additional compared specimen

distant *zarina* Ec-Geo-22438|Ecuador|Zamora Chinchipe|BOLD:AAB3113

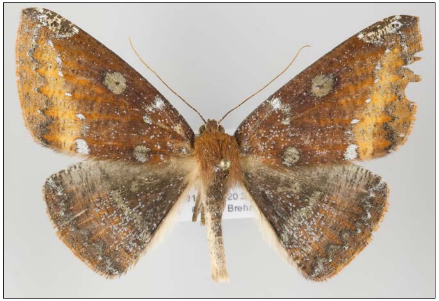

LMR-Geo-

Compared specimen:

LMR-Geo-

0375

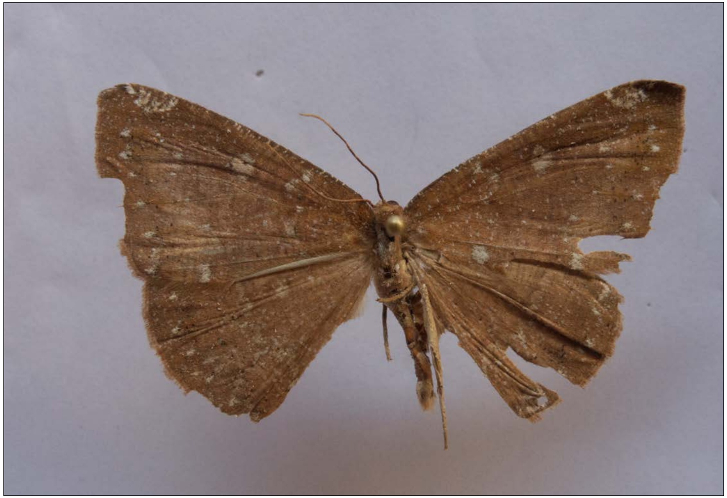

Hypochroma nr flavinigra Warren (TL: ?)

BC

BOLD:AEA7619

OTU-230

Additional compared specimen

LMR-Geo-

Compared specimen:

NHM type

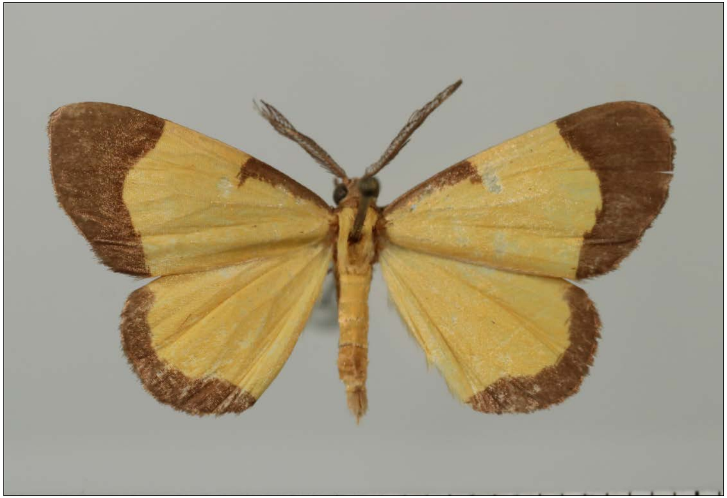

LMR-Geo-

0164

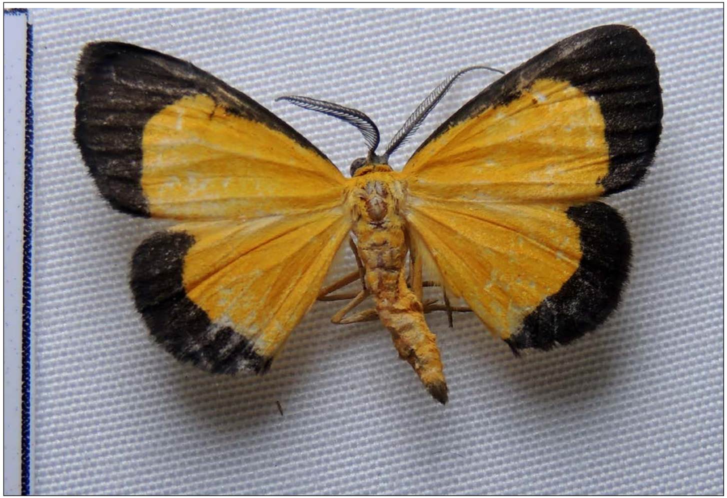

Iridopsis huambaria Oberthür (TL:)

BC

BOLD:AAF2680
OTU-74

Additional compared specimen
= Pe-Geo-1032|Peru|Cuzco| #2959

LMR-Geo-

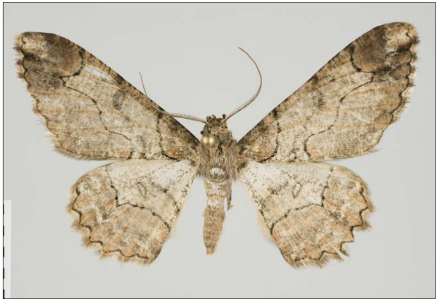

Compared specimen:

NHM type

LMR-Geo-

0026

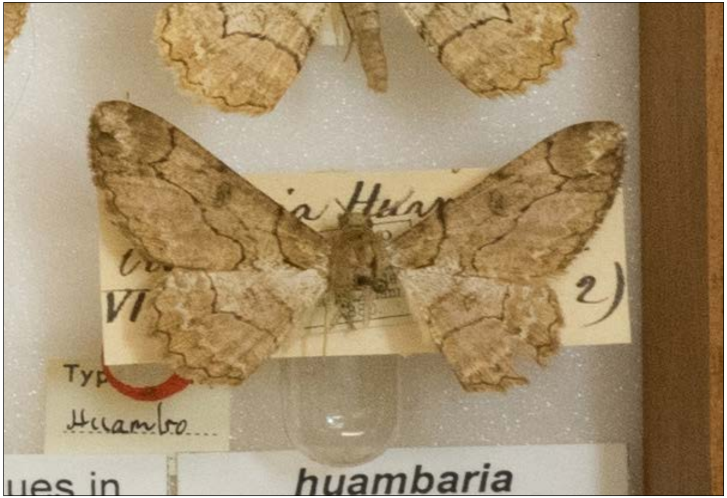

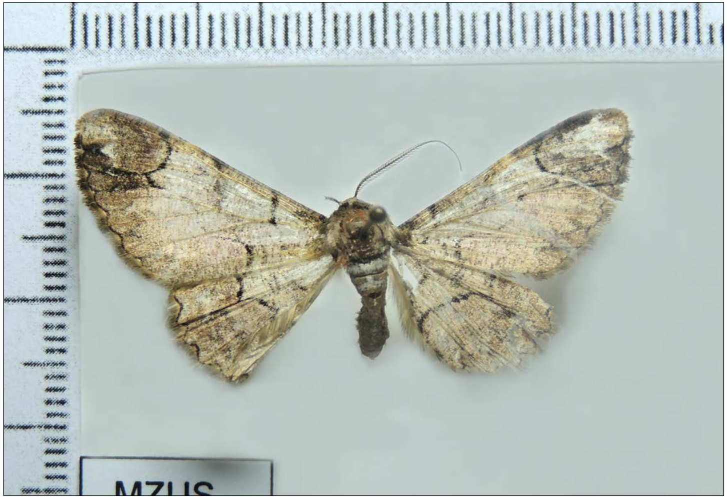

Iridopsis nr scolancala Prout (TL: Colombia: Papayan; Coreato, Cauca)

BC

no BIN / 408 bp	
OTU-113	

Additional compared specimen
near Ec-Geo-22701|Ecuador|Zamora Chinchipe|BOLD:AAN4371

LMR-Geo-

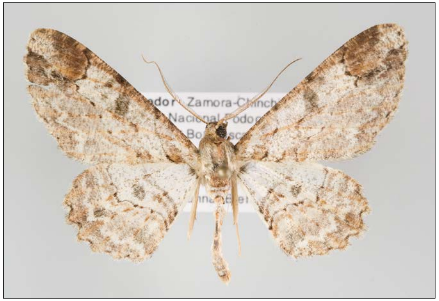

Compared specimen:

NHM type

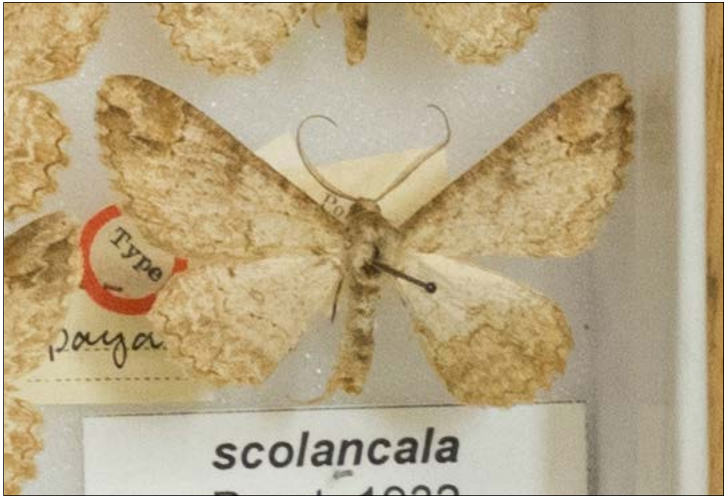

LMR-Geo-

0094

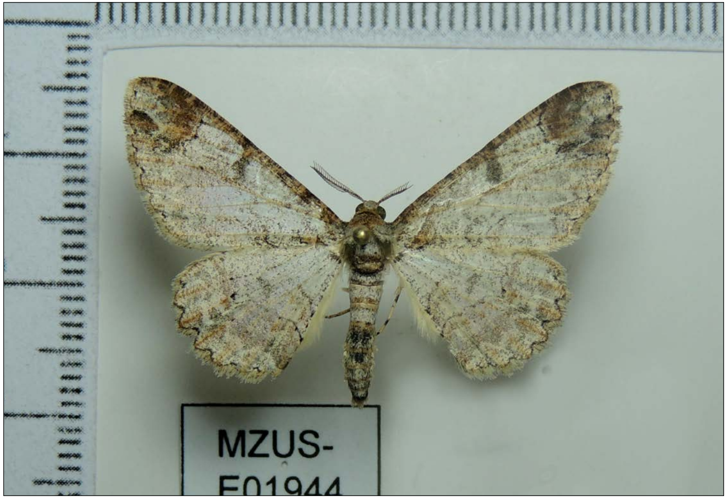

Iridopsis nr subnigrata Warren (TL: Peru (south-east): Carabaya, Santo Domingo, 6500 ft)

BC

BOLD:AEE5237
OTU-98

Additional compared specimen
near Ec-Geo-22177|Ecuador|Zamora Chinchipe|BOLD:AAF2674

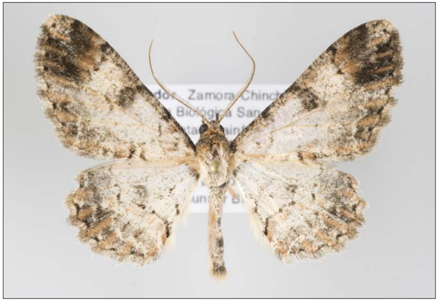

LMR-Geo-
0057

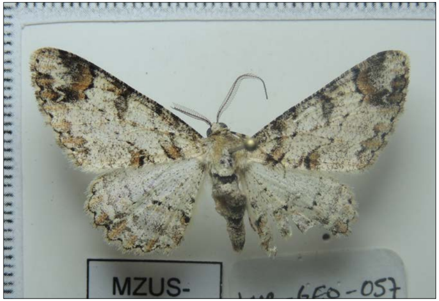

Compared specimen:
(no photo available)

LMR-Geo-
0063

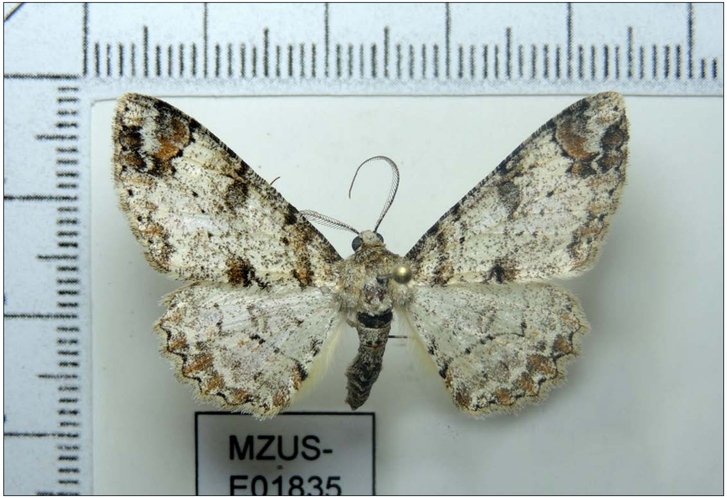

Iridopsis nr subnigrata Warren (TL: Peru (south-east): Carabaya, Santo Domingo, 6500 ft)

BC

BOLD:AEB8456
OTU-9

Additional compared specimen
near Ec-Geo-22177|Ecuador|Zamora Chinchipe|BOLD:AAF2674

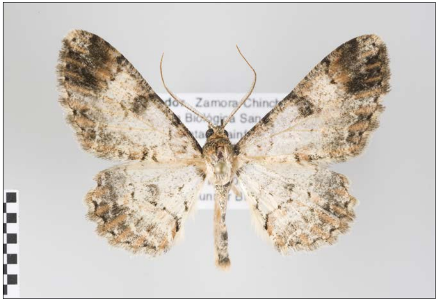

LMR-Geo-

Compared specimen:
(no photo available)

LMR-Geo-
0312

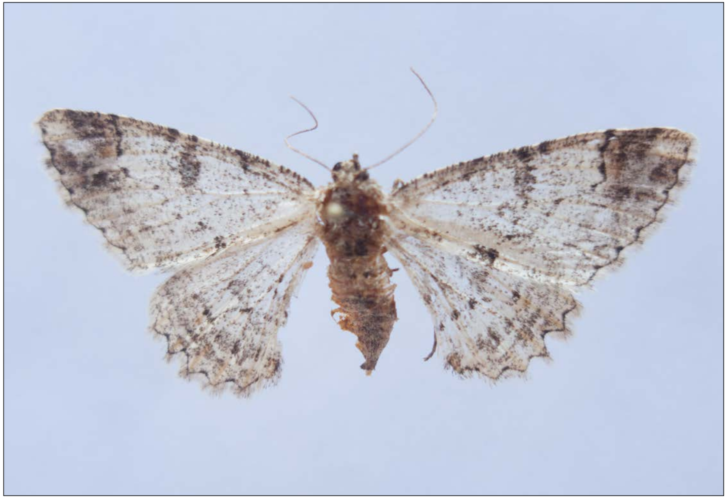

Iridopsis sp (TL:)

BC

BOLD:AEE8244
OTU-94

Additional compared specimen

LMR-Geo-

Compared specimen:

LMR-Geo-

0045

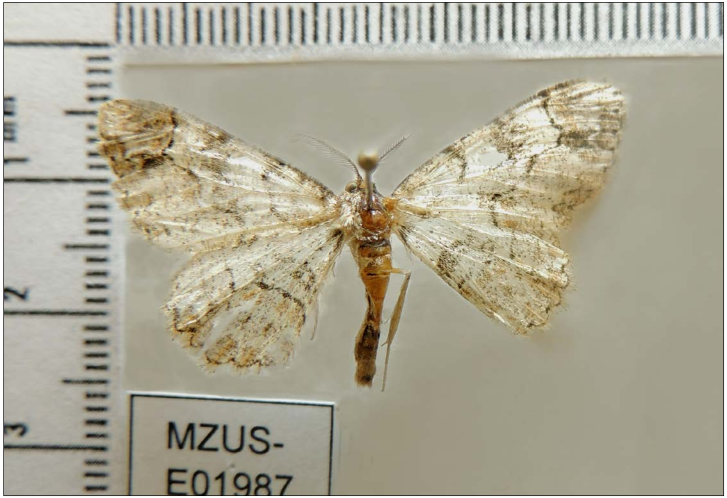

Iridopsis sp (TL:)

BC

BOLD:AEE3609

OTU-109

Additional compared specimen
near: Ec-Geo-24225|Ecuador|Zamora Chinchipe|BOLD:AAW5552

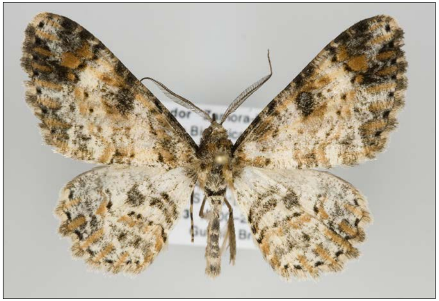

LMR-Geo-

Compared specimen:
(no photo available)

LMR-Geo-
0067

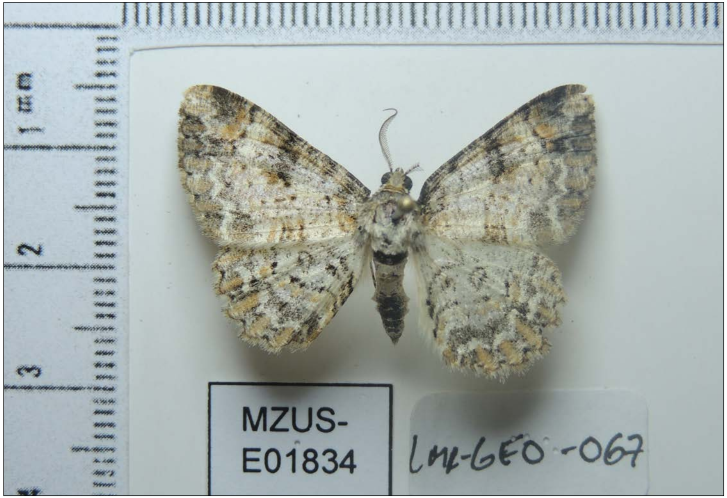

Iridopsis validaria Guenée (TL: Brazil: Rio de Janeiro])

BC

BOLD:AAA7224

OTU-178

Additional compared specimen
= Ec-Geo-16927|Ecuador|Zamora Chinchipe

LMR-Geo-

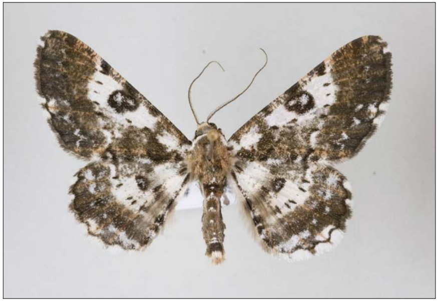

Compared specimen:

NHM type of synonym

LMR-Geo-

0226

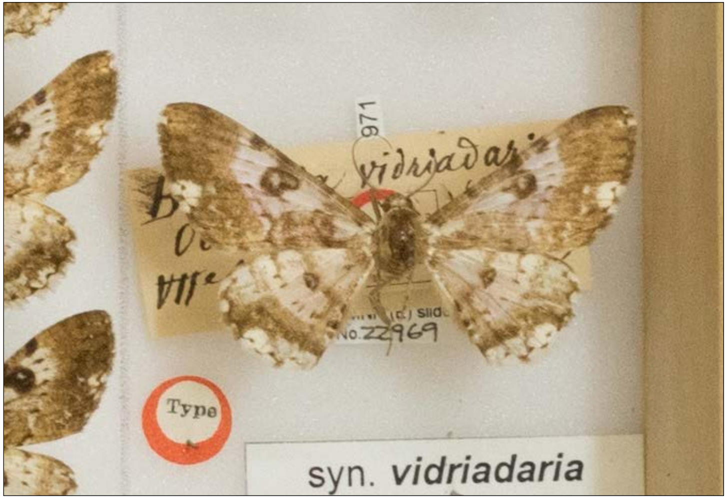

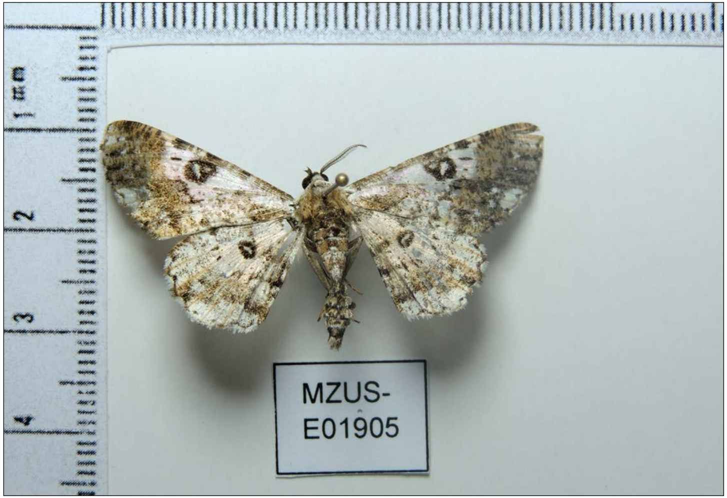

Isochromodes nr epioneata Walker (TL: [Brazil]: Rio de Janeiro)

BC

BOLD:AEE8755
OTU-189

Additional compared specimen

LMR-Geo-

Compared specimen:

NHM type of synonym: *submarginata* Warren

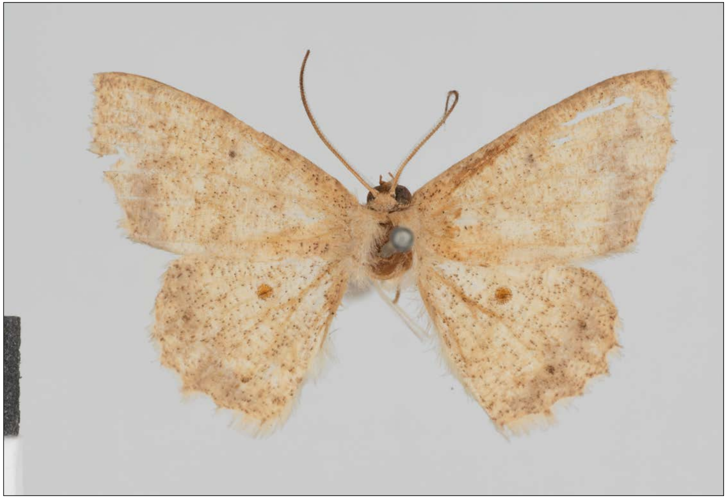

LMR-Geo-

0225

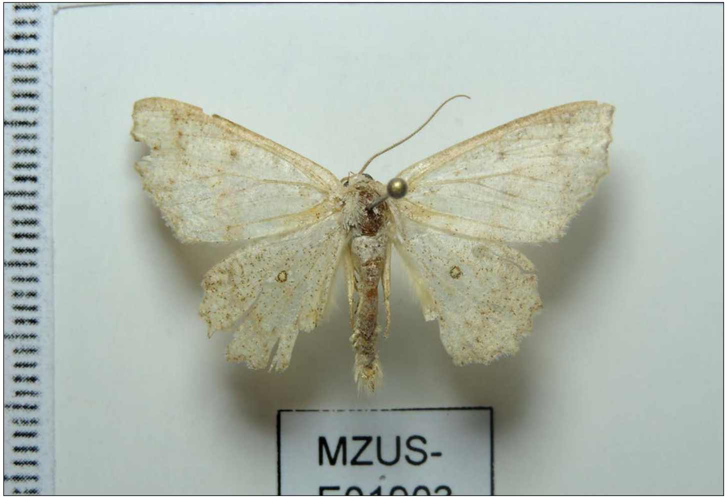

Isochromodes sabularia Dognin (TL: Ecuador, San-Francisco near Loja)

BC

BOLD:AAC2727

OTU-188

Additional compared specimen
= Ec-Geo-19097|Ecuador|Zamora Chinchipe

LMR-Geo-

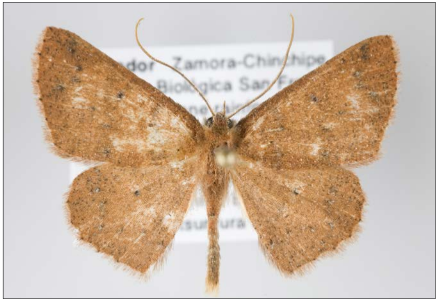

Compared specimen:
USNM photo

LMR-Geo-
0222

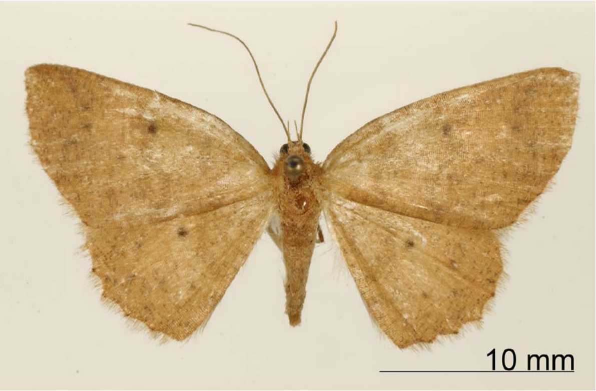

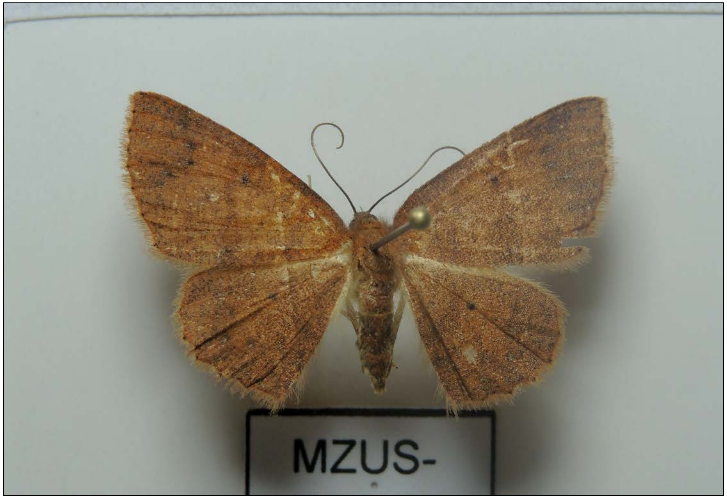

Isochromodes sheila Schaus (TL: Costa Rica, Juan Vinas; Sitio; Tuis)

BC

BOLD:AEE1230
OTU-179

Additional compared specimen

near 19209|Ecuador|Zamora Chinchipe|BOLD:ACJ8324

LMR-Geo-

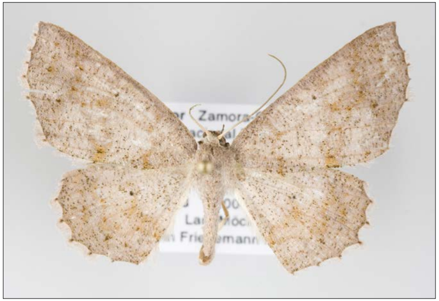

Compared specimen:

USNM type

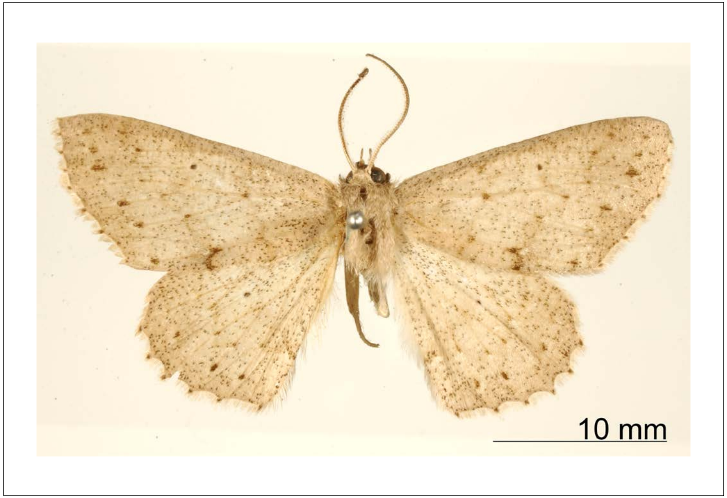

LMR-Geo-

0227

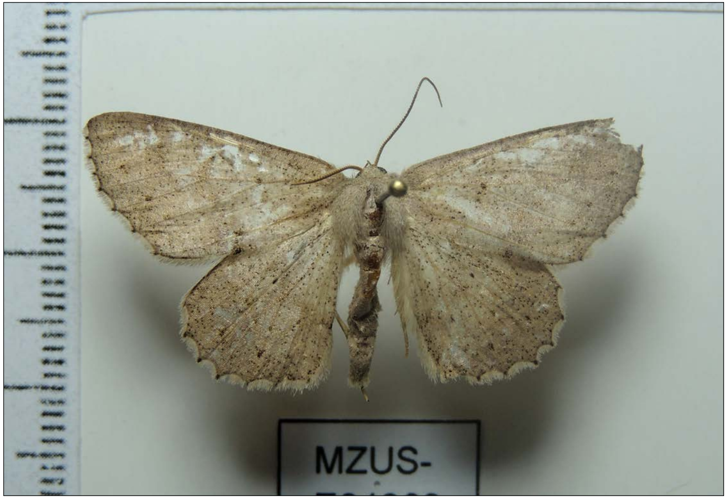

Isochromodes sp (TL:)

BC

BOLD:AEE8304
OTU-103

Additional compared specimen
near Ec-Geo-22601|Ecuador|Zamora Chinchipe|BOLD:AAP1695

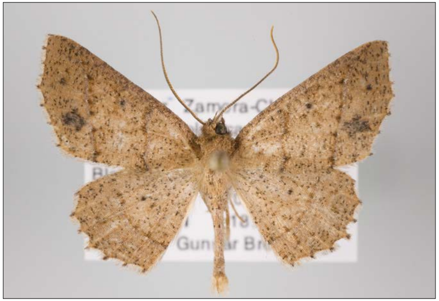

LMR-Geo-
0052

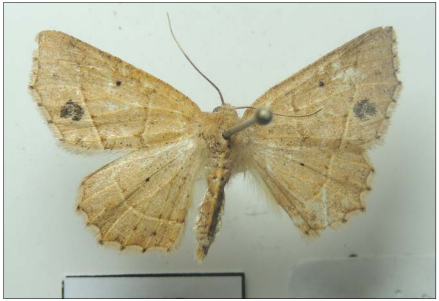

Compared specimen:

LMR-Geo-
0053

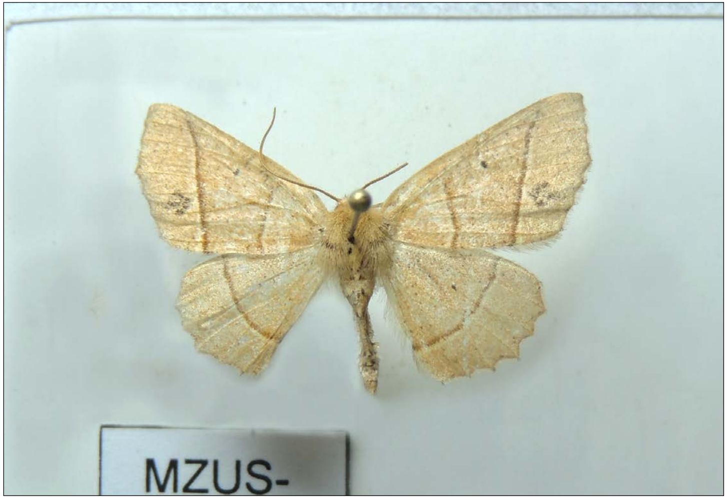

Isochromodes sp (TL:)

BC

BOLD:AEE8305
OTU-165

Additional compared specimen
near Ec-Geo-55261|Ecuador|Loja|BOLD:AAM5110

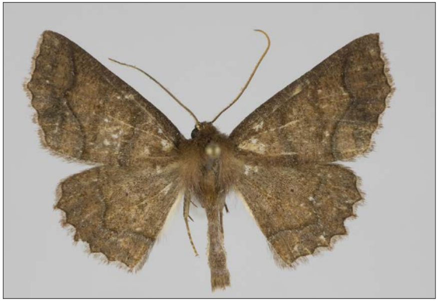

LMR-Geo-

Compared specimen:

LMR-Geo-
0213

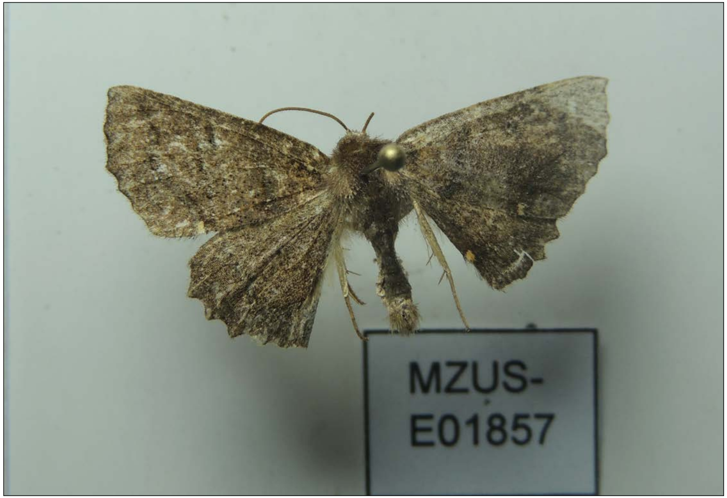

Leuciris nr mysteriotis Prout (TL: Puerto Rico)

BC

BOLD:AAO4885
OTU-136

Additional compared specimen
distant: Ec-Geo-22661|Ecuador|Zamora Chinchipe|BOLD:AAI1310

LMR-Geo-

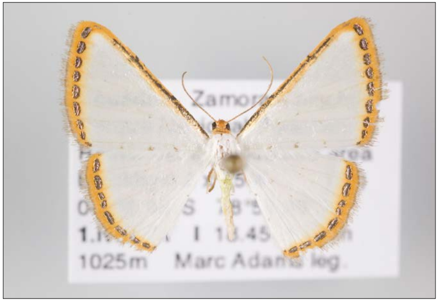

Compared specimen:

NHM type

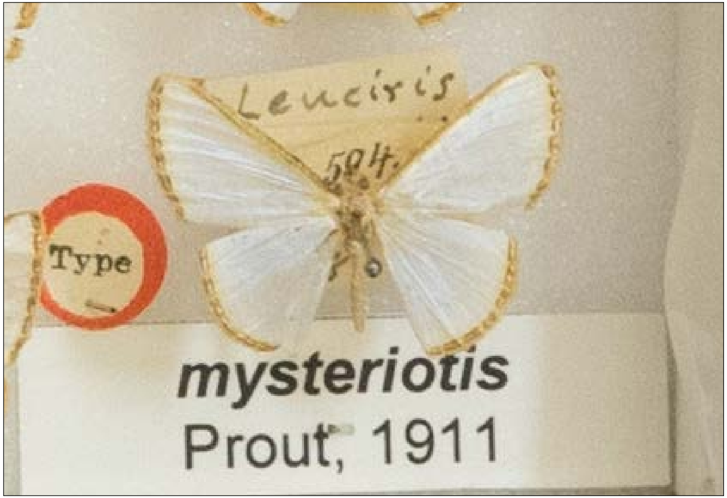

LMR-Geo-
0112

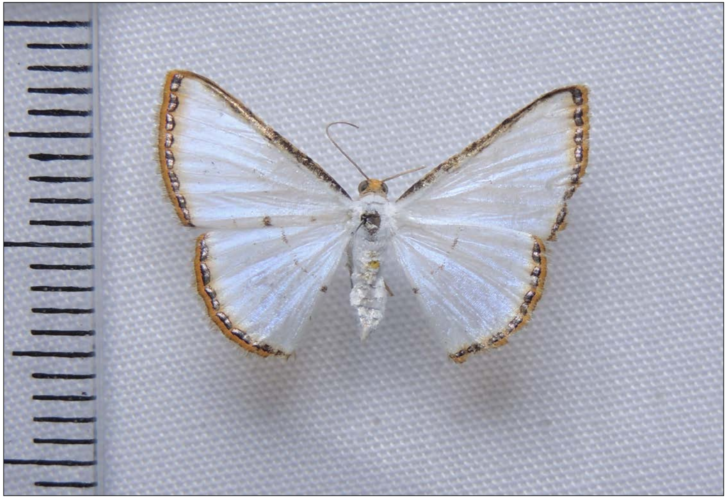

Leucula festiva Cramer (TL: Surinam)

BC

BOLD:AEC0345

OTU-47

Additional compared specimen
distant: Pe-Geo-0609|Peru|Cuzco|BOLD:AAF8123

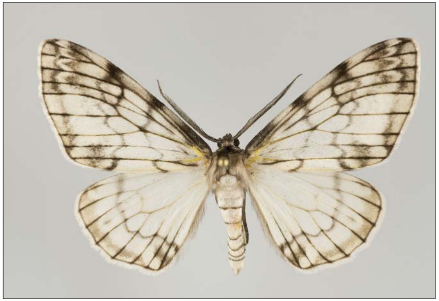

LMR-Geo-

Compared specimen:

NHM no type

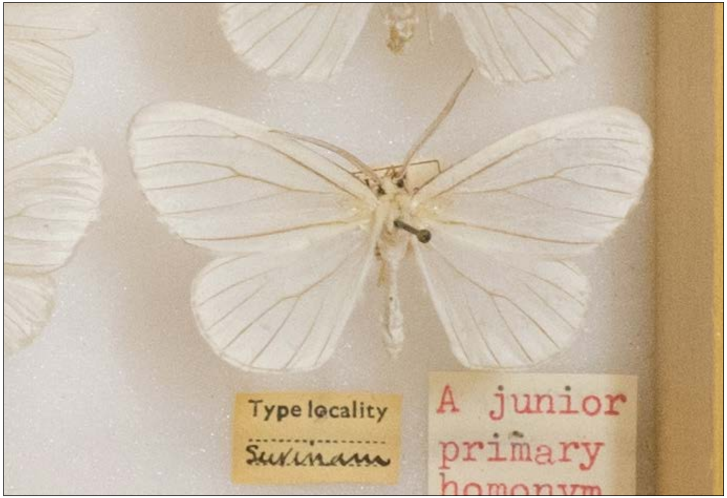

LMR-Geo-

0358

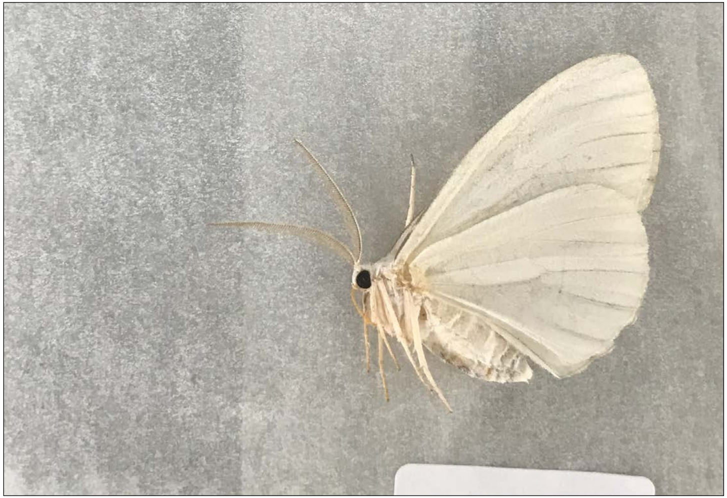

Lomographa sp (TL:)

BC

no BIN 339 bp	
OTU-52	

Additional compared specimen

LMR-Geo-

Compared specimen:

LMR-Geo-
0039

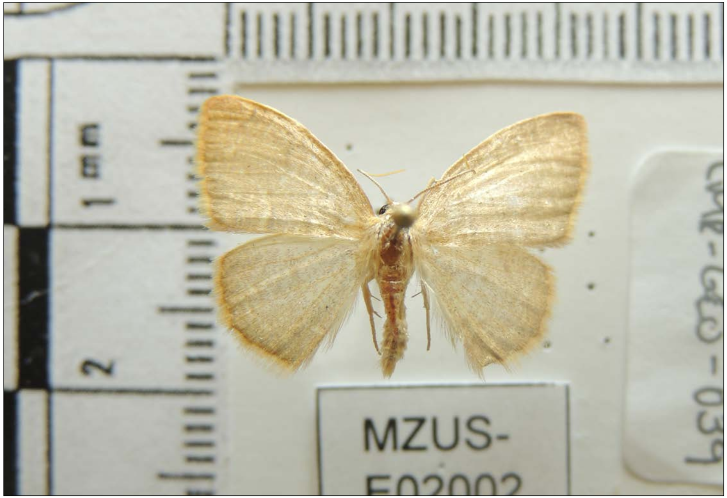

Lomographa x (TL: x)

BC

BOLD:AEE5493
OTU-107

Additional compared specimen
= Ec-Geo-18055|Ecuador|Loja|BOLD:AAI1081

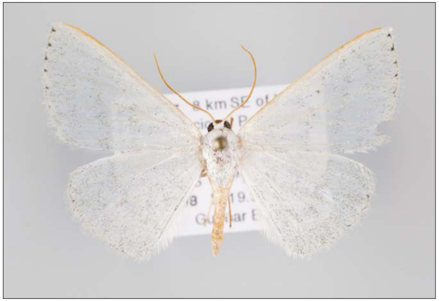

LMR-Geo-

Compared specimen:

LMR-Geo-
0069

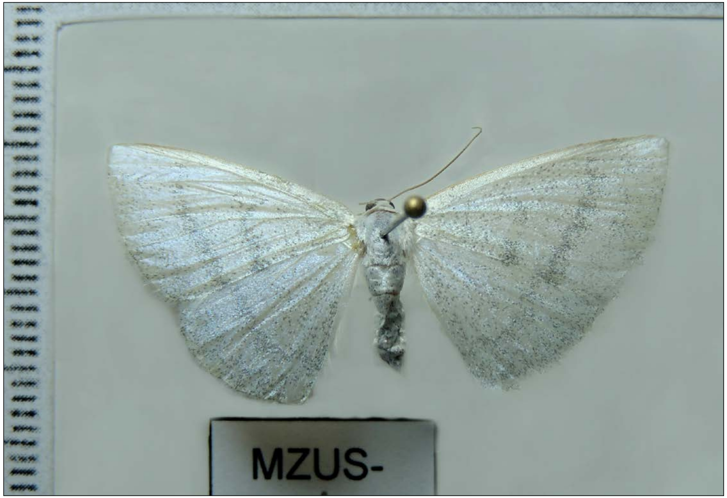

Lomographa sp (TL: x)

BC

BOLD:AEE4997
OTU-82

Additional compared specimen
near Pe-Geo-0691|Peru|Cuzco|BOLD:ADF1762

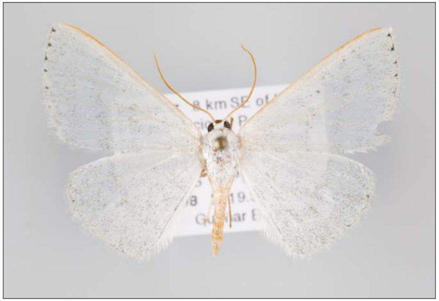

LMR-Geo-

Compared specimen:

LMR-Geo-
0248

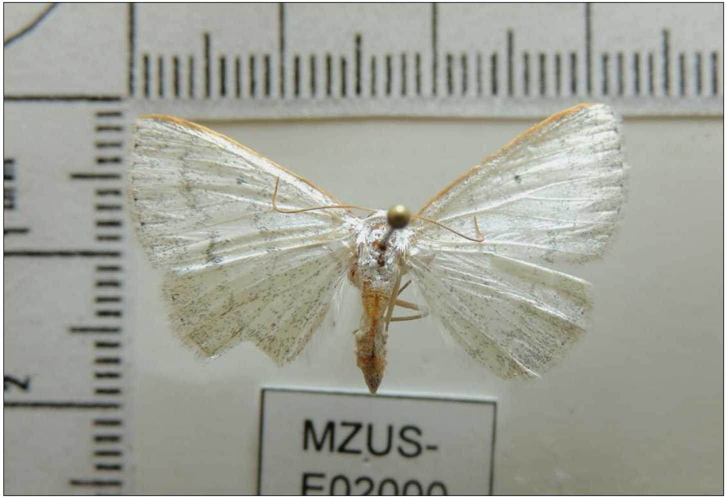

Lomographa nr tributaria Walker (TL: [Colombia]: Bogota)

BC

BOLD:AAE0992

OTU-57

Additional compared specimen
= 17155|Ecuador|Zamora Chinchipe|BOLD:AAE0992

LMR-Geo-

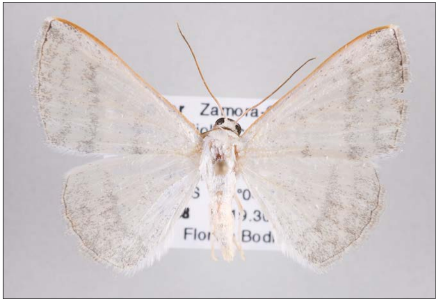

Compared specimen:

NHM type

LMR-Geo-

0380

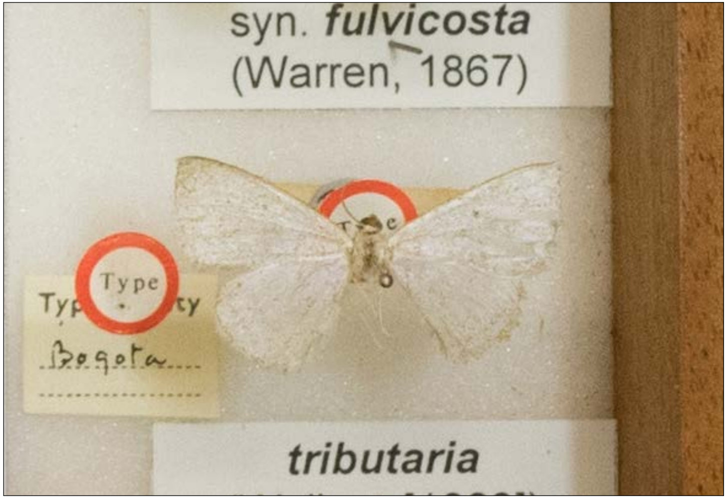

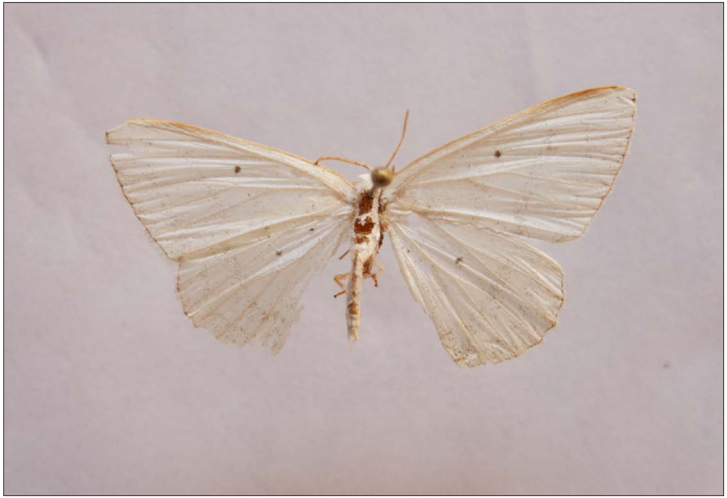

Macaria abydata Guenée (TL: Brazil)

BC

BOLD:AAA1494

OTU-139

Additional compared specimen

LMR-Geo-
0113 (no photo)

Compared specimen:
NHM type

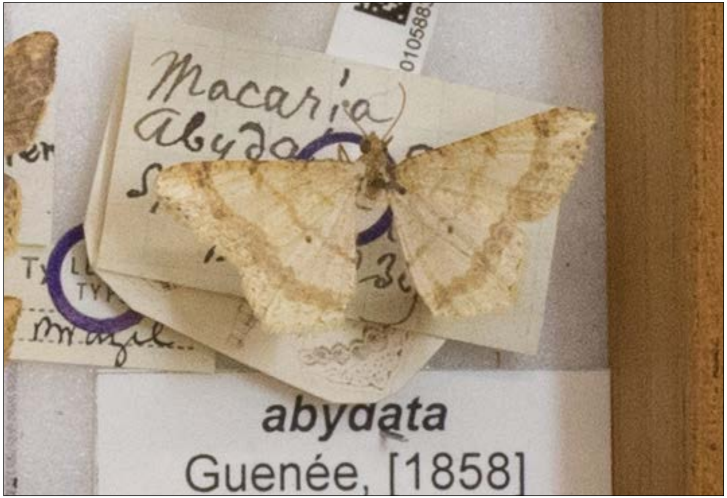

LMR-Geo-
0116

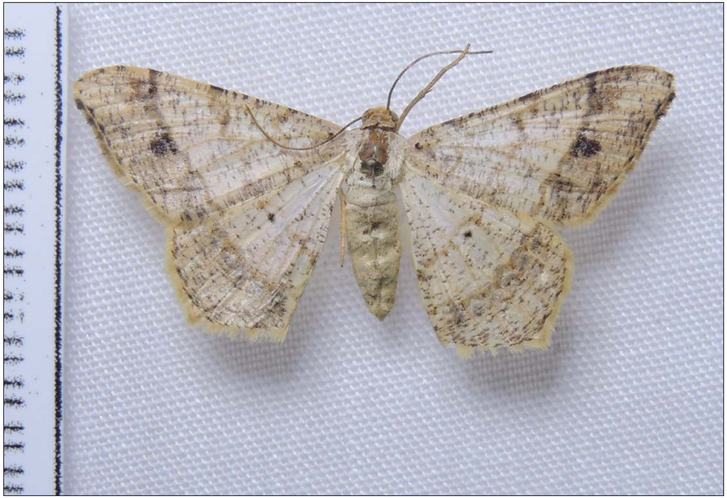

Macaria nr bejucoaria Dyar (TL: Panama: Canal Zone, Bejuco River)

BC

OLD:ACJ8353
OTU-81

Additional compared specimen
= 19854|Ecuador|Zamora Chinchipe

LMR-Geo-

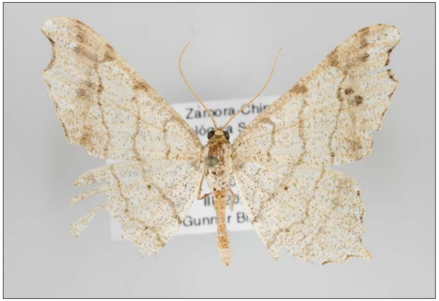

Compared specimen:

USNM type

LMR-Geo-

0036

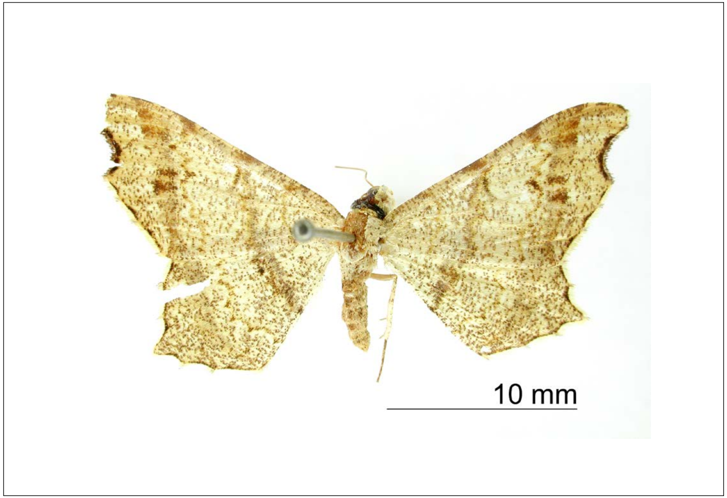

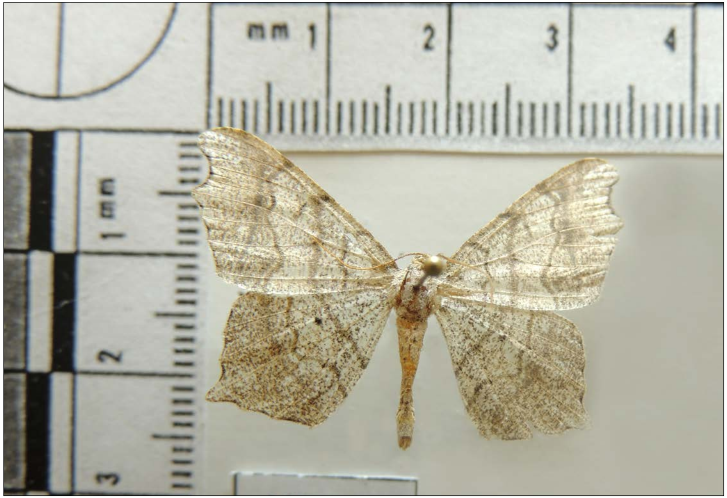

Macaria carpo Druce (TL: Mexico, Guatemala, Costa Rica...)

BC

BOLD:AAA0866
OTU-41

Additional compared specimen

LMR-Geo-
0354, (0080, 0077 no photo)

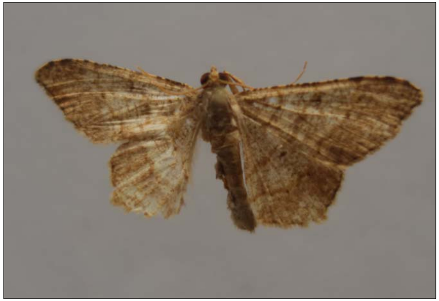

Compared specimen:
NHM type

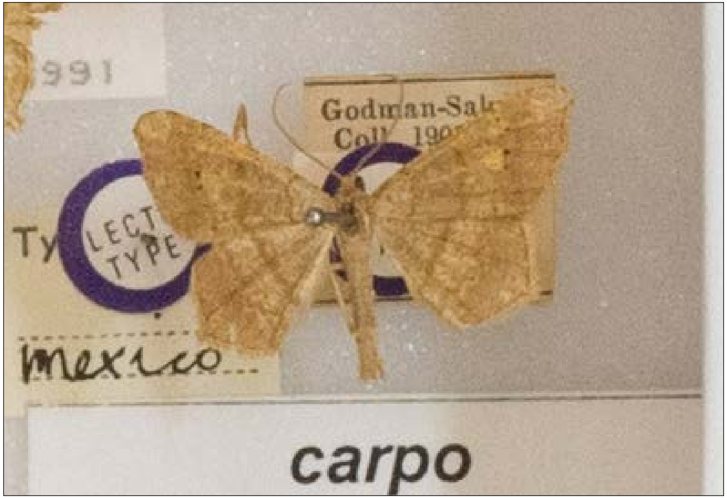

LMR-Geo-
0356

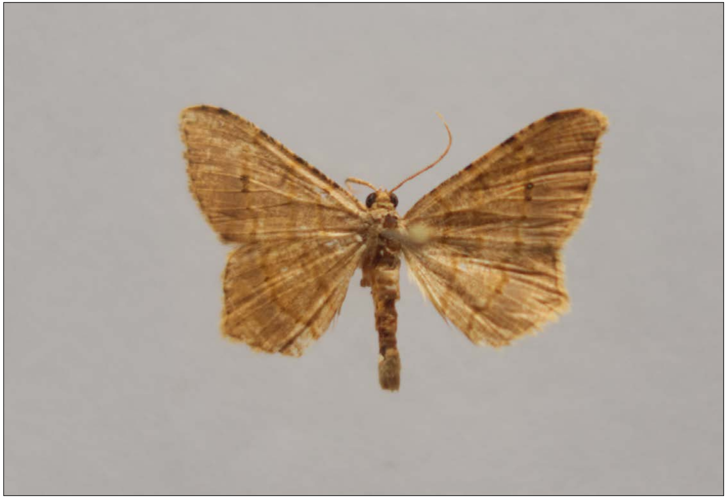

Macaria nr triplicaria Herrich-Schäffer (TL: Brazil)

BC

BOLD:AAR4948	
OTU-78	

Additional compared specimen
= 24137|Ecuador|Zamora Chinchipe

LMR-Geo-

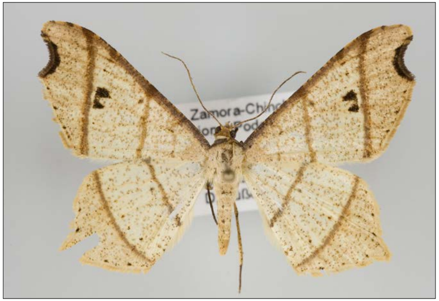

Compared specimen:
NHM no type

LMR-Geo-
0022

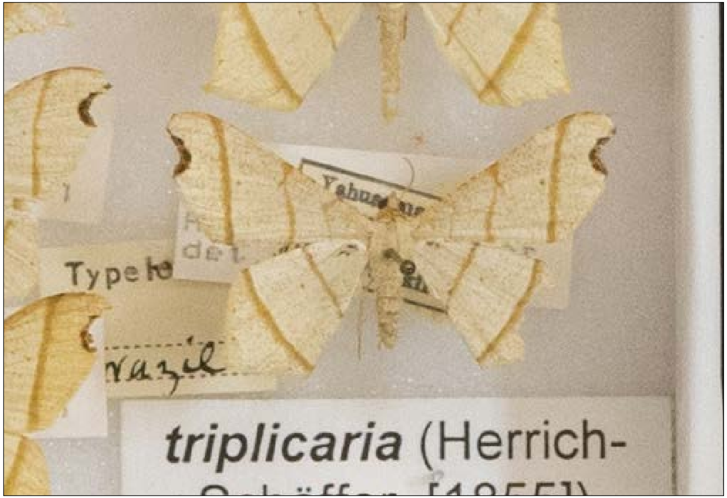

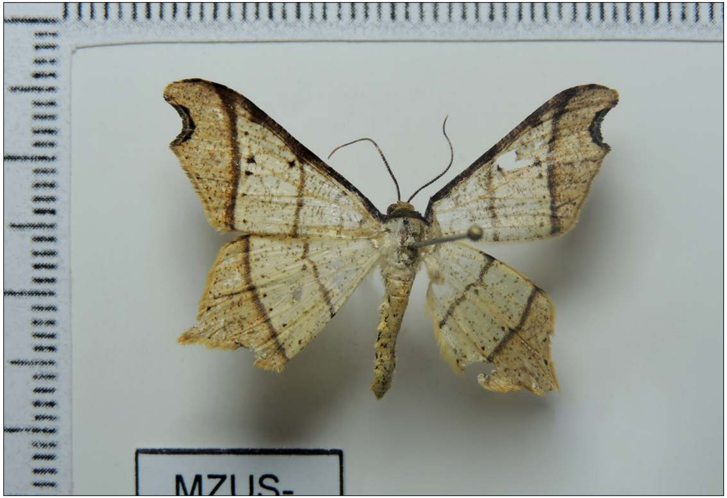

Macaria sp (TL:) closest match in *Macaria:* 94%

BC

BOLD:AEE2850

OTU-116

Additional compared specimen

LMR-Geo-

Compared specimen:

LMR-Geo-
0079

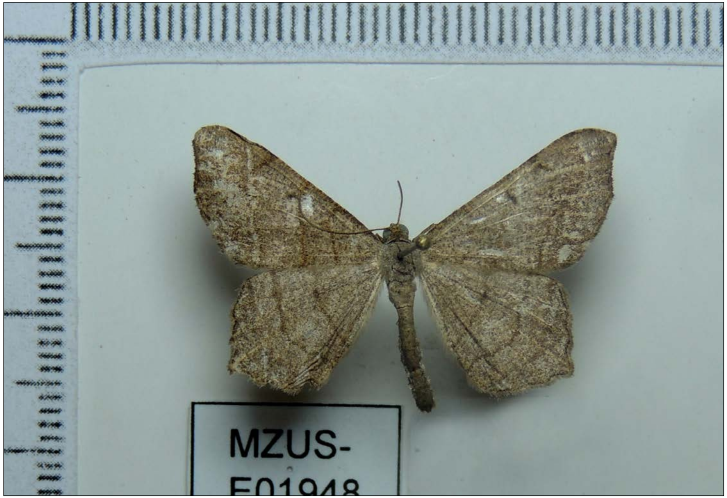

Macaria sp (TL:)

BC

BOLD:AAM8794
OTU-138

Additional compared specimen

LMR-Geo-
0114 (449 bp, no photo)

Compared specimen:

LMR-Geo-
0115

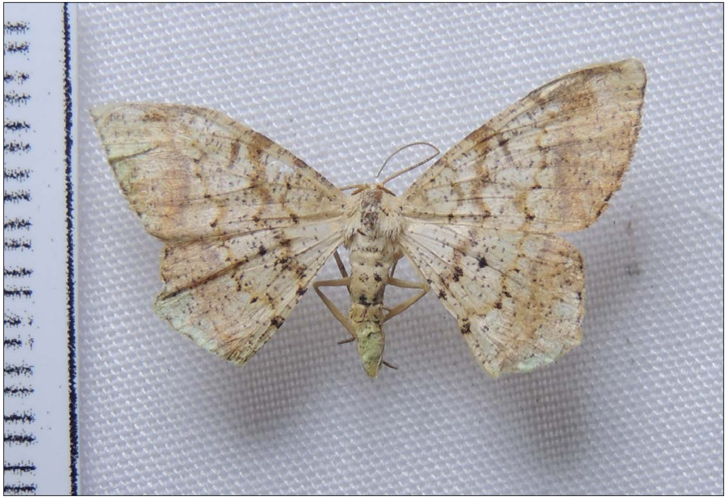

Macaria sp (TL:)

BC

BOLD:AAH5010
OTU-141

Additional compared specimen

LMR-Geo-

Compared specimen:

LMR-Geo-

0117

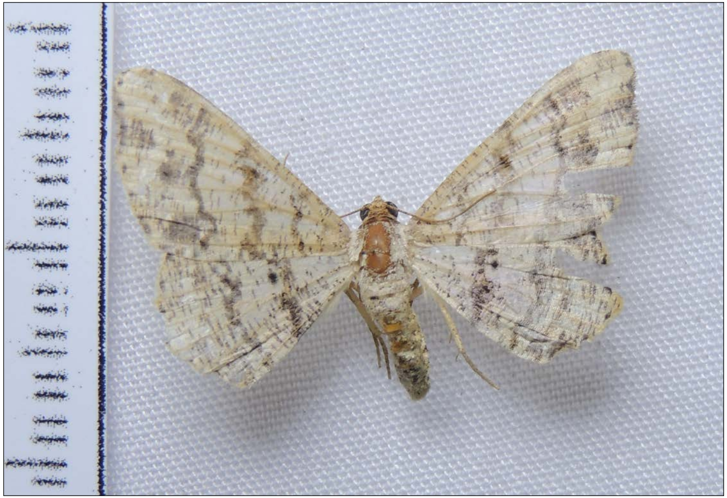

Macaria sp (TL:)

BC

BOLD:AEE4560
OTU-140

Additional compared specimen

LMR-Geo-

Compared specimen:

LMR-Geo-

0119

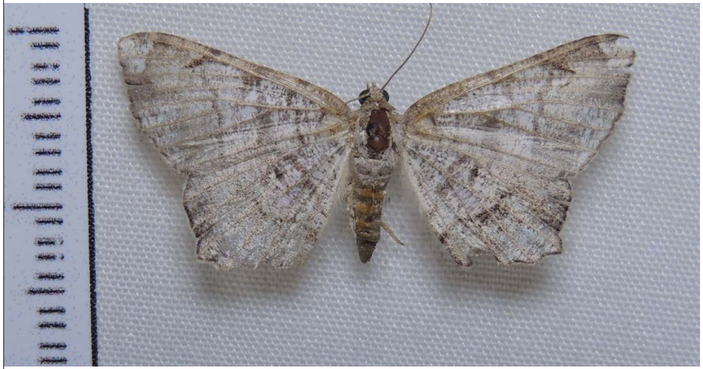

Macaria sp (TL:)

BC

BOLD:AAJ2405
OTU-16

Additional compared specimen

LMR-Geo-

Compared specimen:

LMR-Geo-

0324

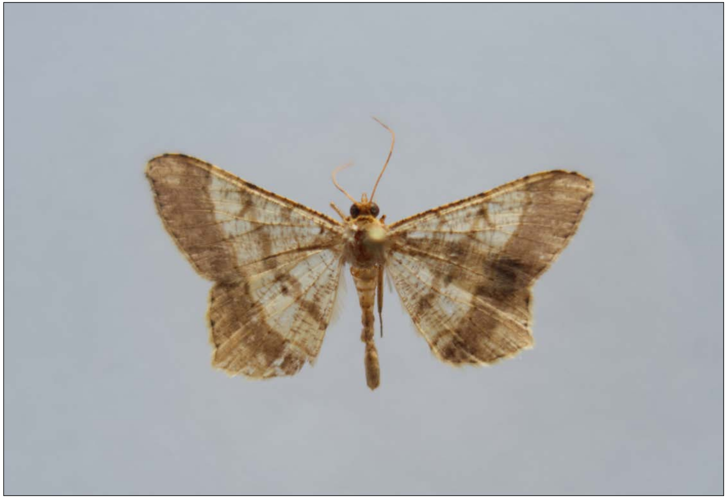

Macaria sp (TL:)

BC

BOLD:AAK6423
OTU-22

Additional compared specimen
near: Pe-Geo-0727|Peru|Huanuco|BOLD:ADF0535

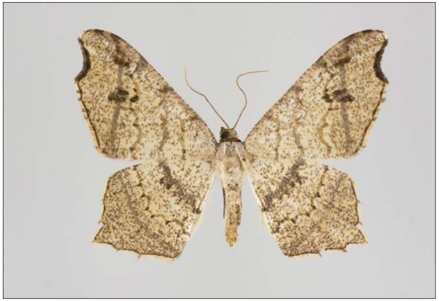

LMR-Geo-

Compared specimen:

LMR-Geo-
0330

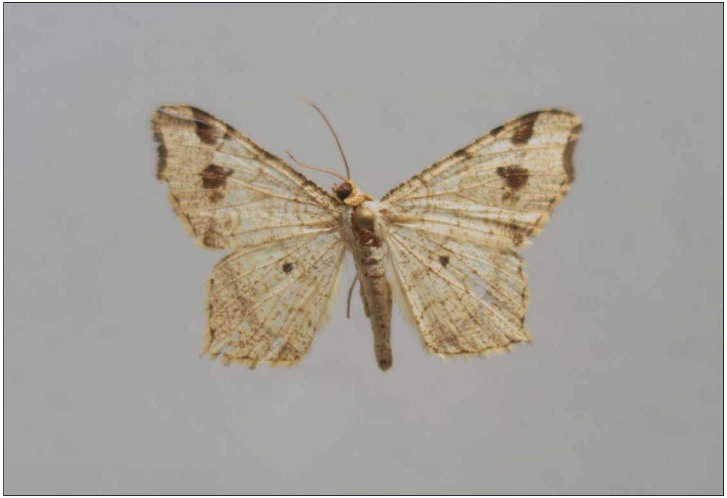

Melanolophia sp (TL:)

BC

BOLD:AEE8422
OTU-181

Additional compared specimen

LMR-Geo-

Compared specimen:

LMR-Geo-
0234

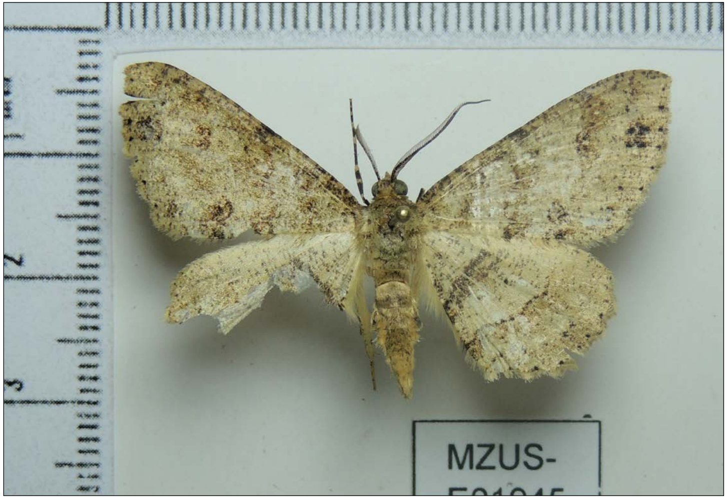

Melinodes detersaria? Herrich-Schäffer (TL: Colombia)

BC
BOLD:ACF7176
OTU-2

Additional compared specimen
= 18695|Ecuador|Zamora Chinchipe

LMR-Geo-

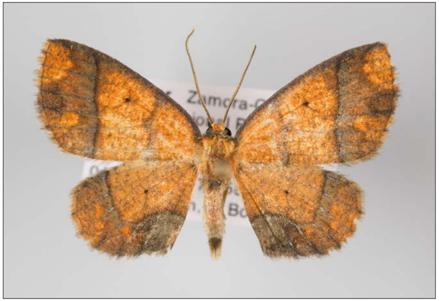

Compared specimen:
NHM no type

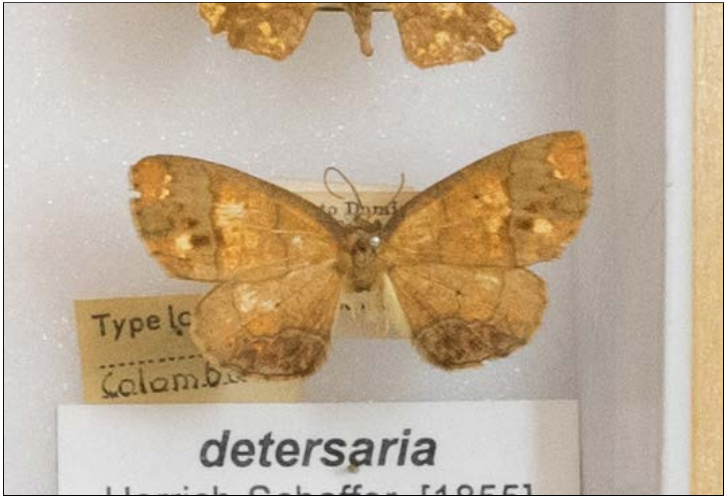

LMR-Geo-
0303

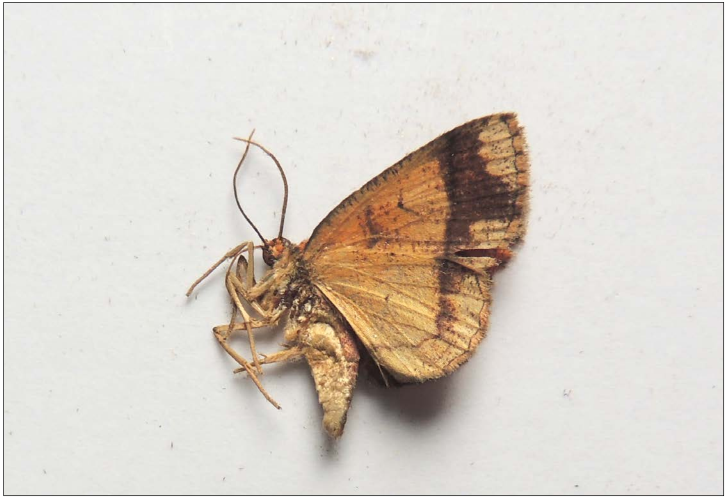

Melinodes nr saeta Dognin (TL: [Ecuador]: Loja)

BC

BOLD:AEE2908

OTU-100

Additional compared specimen
near Pe-Geo-0322|Peru|Cuzco|BOLD:ADF215

LMR-Geo-

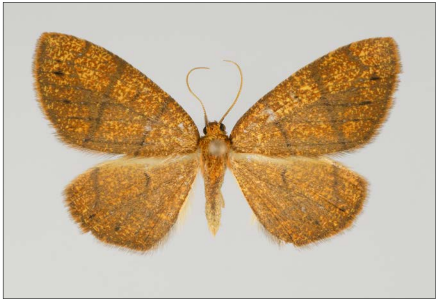

Compared specimen:
USNM type

LMR-Geo-
0054

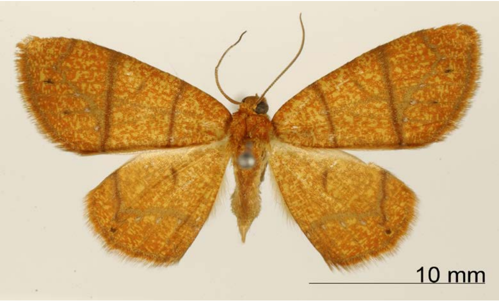

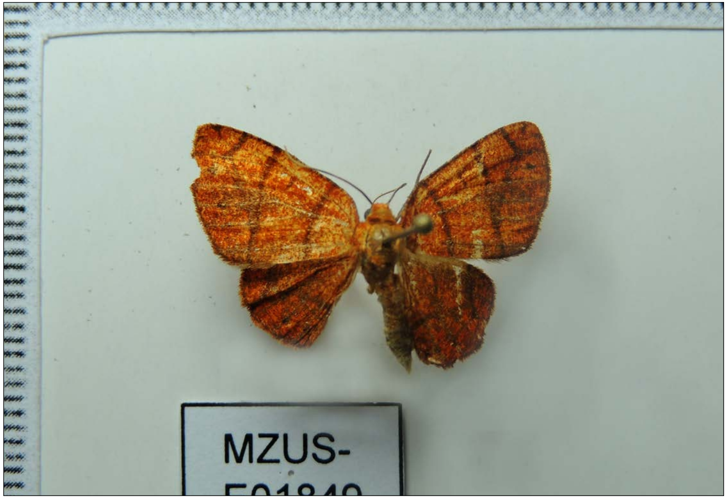

Mesedra nr confinis Warren (TL: Peru (south-east): Agualani, 10,000 ft)

BC

BOLD:AEE3917

OTU-111

Additional compared specimen
near Ec-Geo-16470|Ecuador|Zamora Chinchipe|BOLD:AAK7598

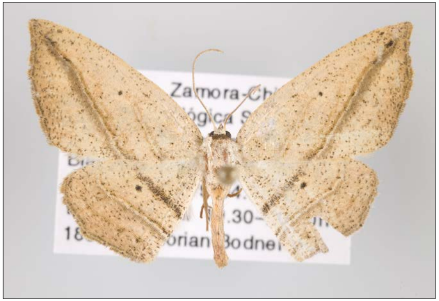

LMR-Geo-

Compared specimen:
xx

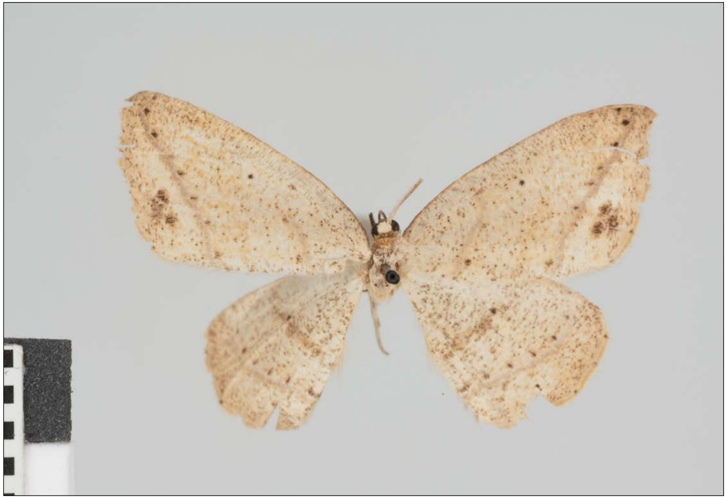

LMR-Geo-
0065

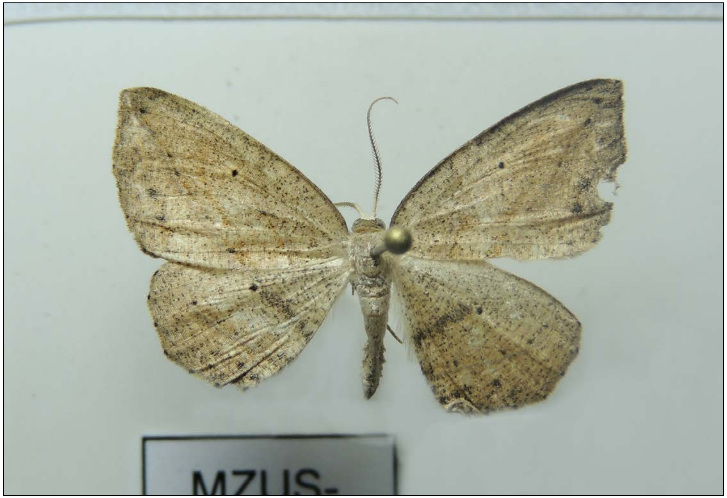

Mesedra sp (TL:)

BC

Additional compared specimen

LMR-Geo-

not in tree
OTU-209

Compared specimen:

LMR-Geo-

0020

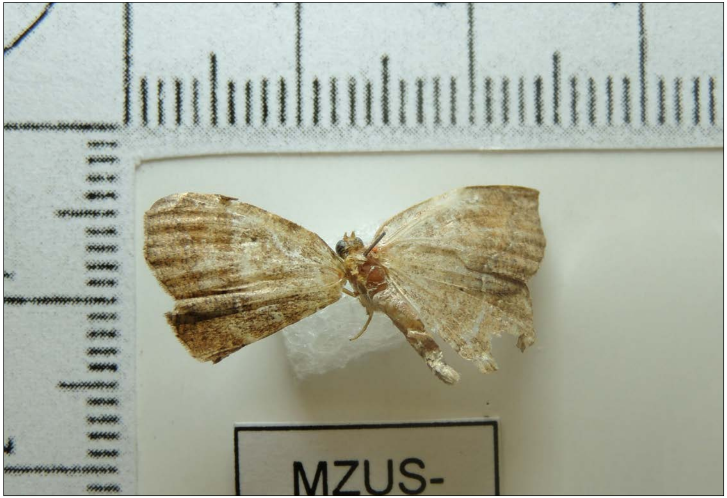

Microxydia nr colorata Warren (TL: Peru, (south-east): Carabaya, Santo Domingo, 6000 ft)

BC

BOLD:AEE2013
OTU-182

Additional compared specimen
near: Ec-Geo-25088|Ecuador|Zamora Chinchipe|BOLD:ACP7162

LMR-Geo-

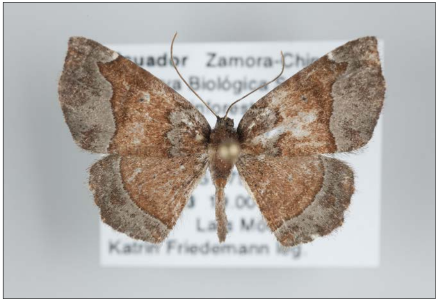

Compared specimen:
NHM type

LMR-Geo-
0231

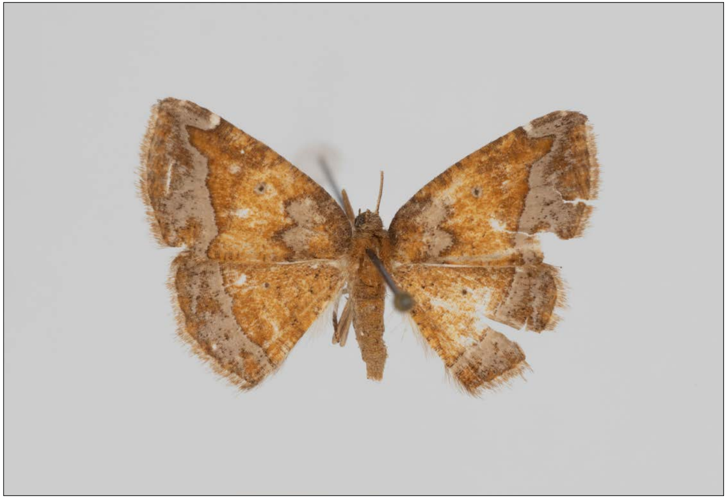

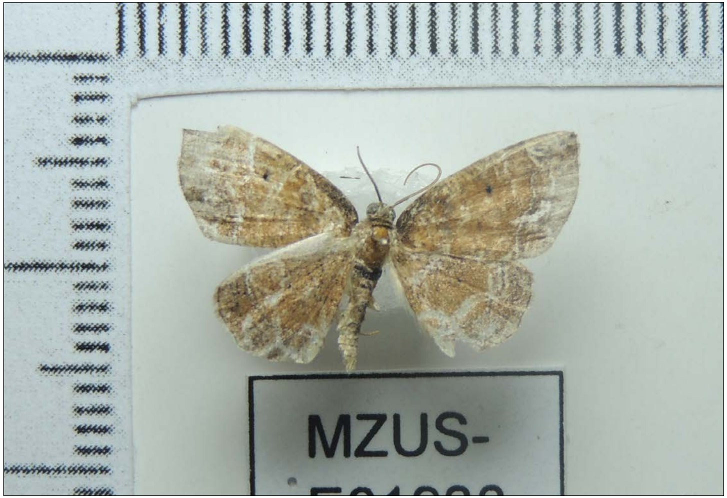

Microxydia sp (TL:)

BC

BOLD:AEE2013
OTU-091

Additional compared specimen
near Ec-Geo-17754|Ecuador|Zamora Chinchipe|BOLD:AAI1520

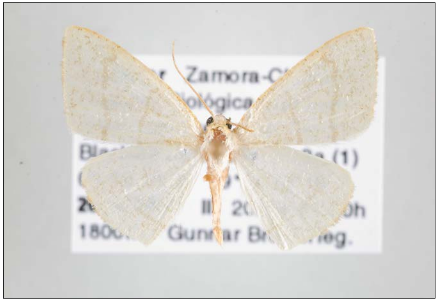

LMR-Geo-

Compared specimen:

LMR-Geo-
0048

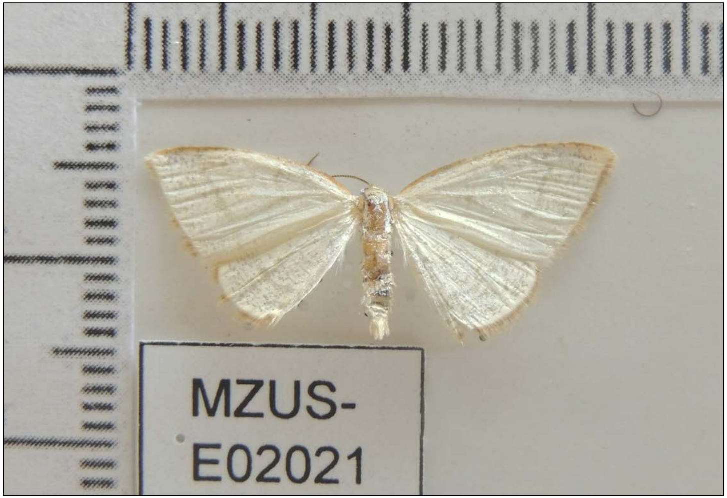

Mychonia nr corticinaria Herrich-Schäffer (TL: Brazil)

BC
BOLD:AEE0464
OTU-93

Additional compared specimen
near Pe-Geo-0300|Peru|Cuzco|BOLD:ADF1416

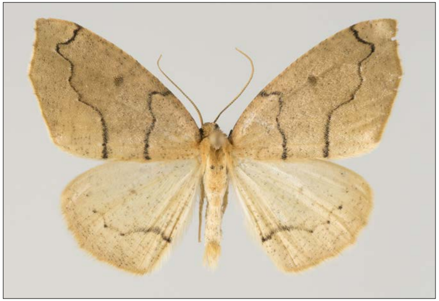

LMR-Geo-

Compared specimen:

NHM type of synonym

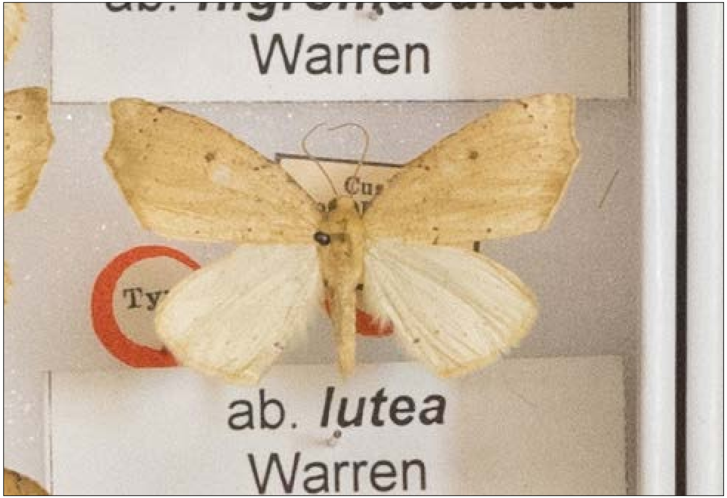

LMR-Geo-

0040

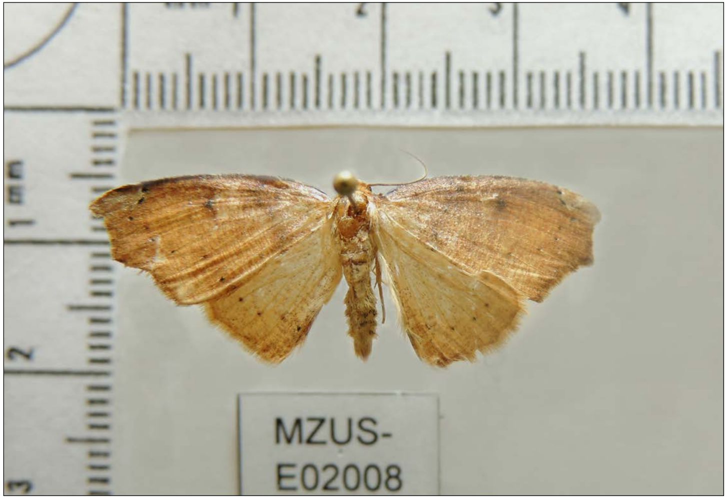

Nematocampa nr anguilifera Oberthür (TL: Peru, Huamba)

BC

BOLD:AAI3075
OTU-69

Additional compared specimen
= Ec-Geo-19010|Ecuador|Zamora Chinchipe

LMR-Geo-

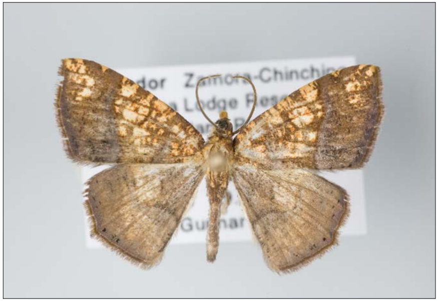

Compared specimen:

NHM type

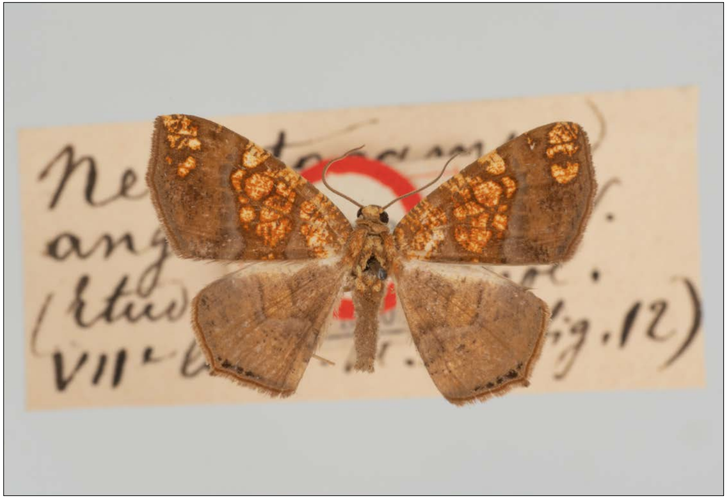

LMR-Geo-

0008

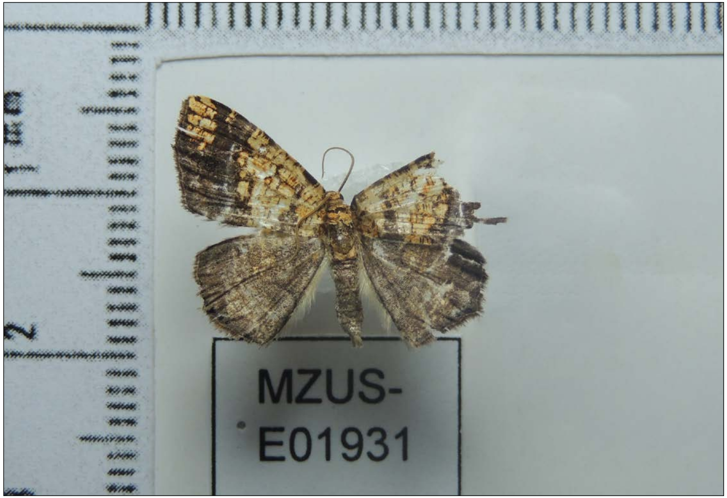

"Nematocampa" nr falsa Warren (TL: French Guiana: St Jean, Maroni River)

BC

BOLD:AEE4428	
OTU-110	

Additional compared specimen
near Pe-Geo-0152|Peru|Cuzco|BOLD:ADE9847

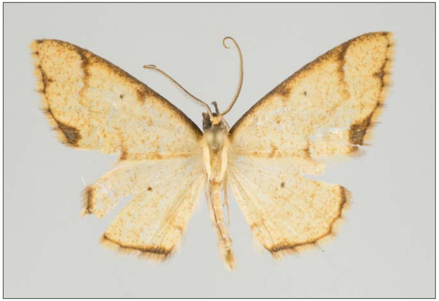

LMR-Geo-
0062

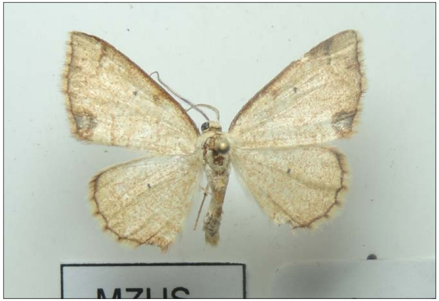

Compared specimen:
USNM type

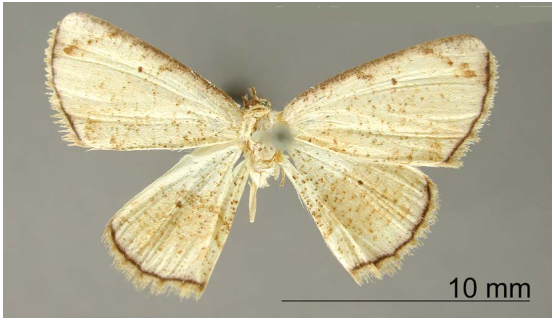

LMR-Geo-
0064

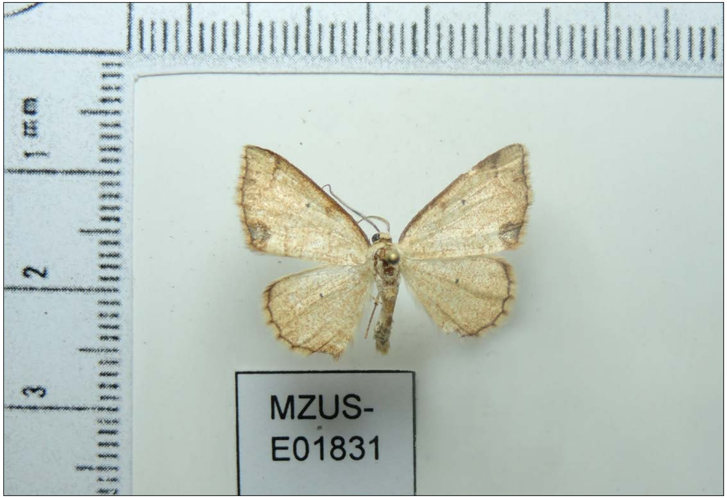

Neoselenia sp (TL:)

BC

BOLD:AEE9673
OTU-213

Additional compared specimen
no close relatives

LMR-Geo-

Compared specimen:

LMR-Geo-
0294

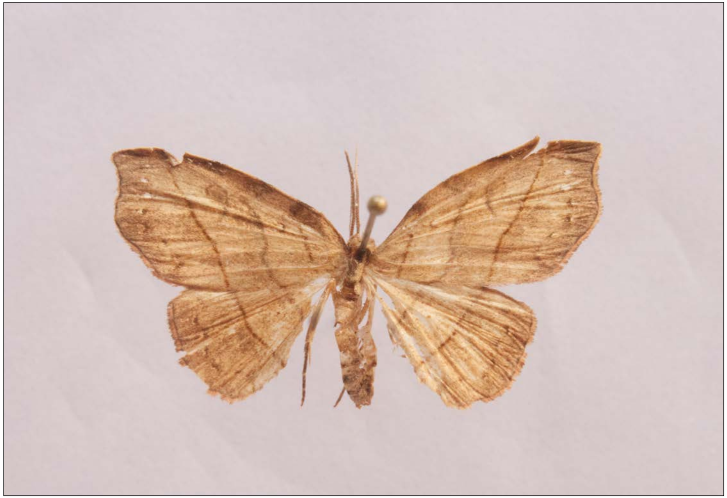

Nephodia nr pellucenta Dognin (TL: [Ecuador]: Loja surroundings)

BC

BOLD:AEC0294
OTU-7

Additional compared specimen
distant Ec-Geo-22588|Ecuador|Zamora Chinchipe|BOLD:AAF4374

LMR-Geo-

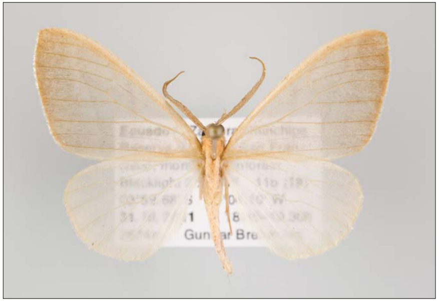

Compared specimen:

USNM type

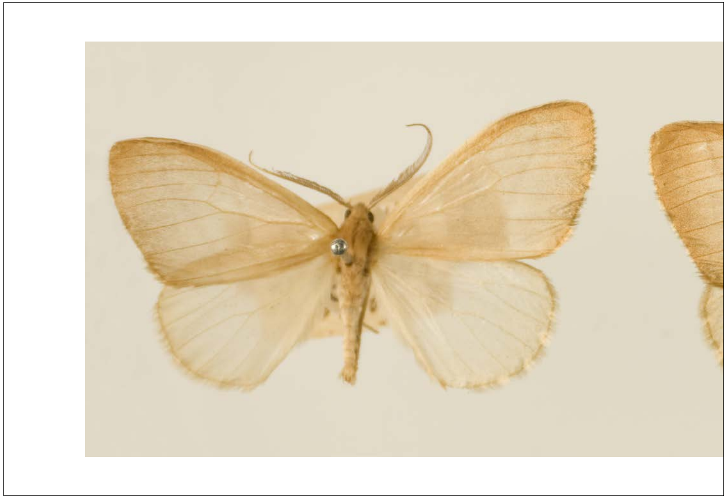

LMR-Geo-

0310

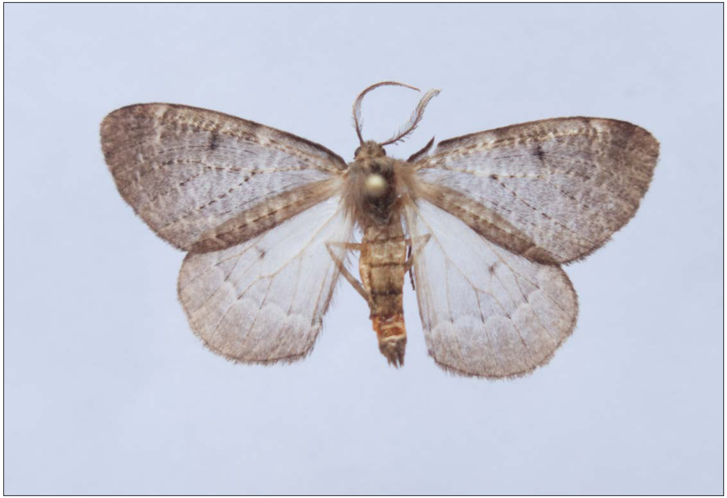

Nephodia sp (TL:)

BC

BOLD:AEB7135
OTU-51

Additional compared specimen
near Pe-Geo-1028|Peru|Cuzco|BOLD:ADF5898

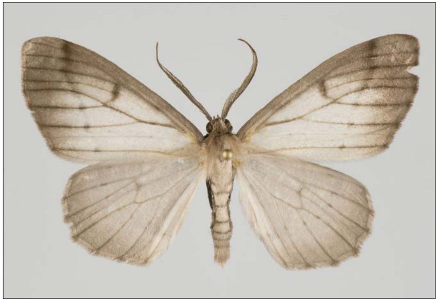

LMR-Geo-
0363

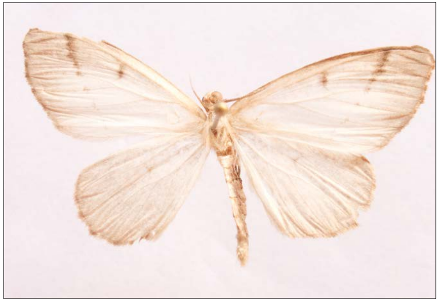

Compared specimen:

LMR-Geo-
0298

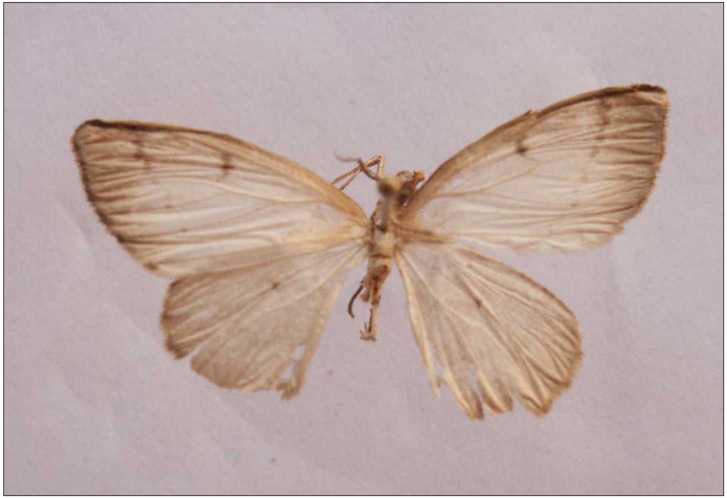

Nephodia sp (TL:)

BC

BOLD:AEB7537
OTU-6

Additional compared specimen
distant Ec-Geo-19045|Ecuador|Zamora Chinchipe|BOLD:ACF7140

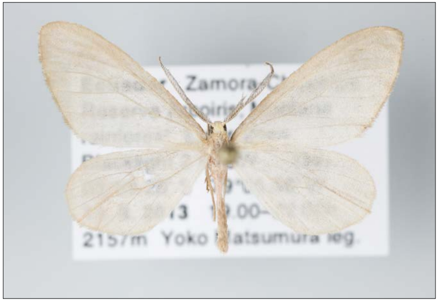

LMR-Geo-
0309

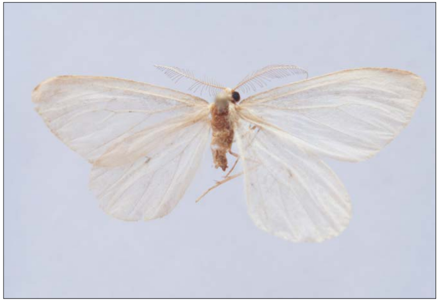

Compared specimen:

LMR-Geo-
0317

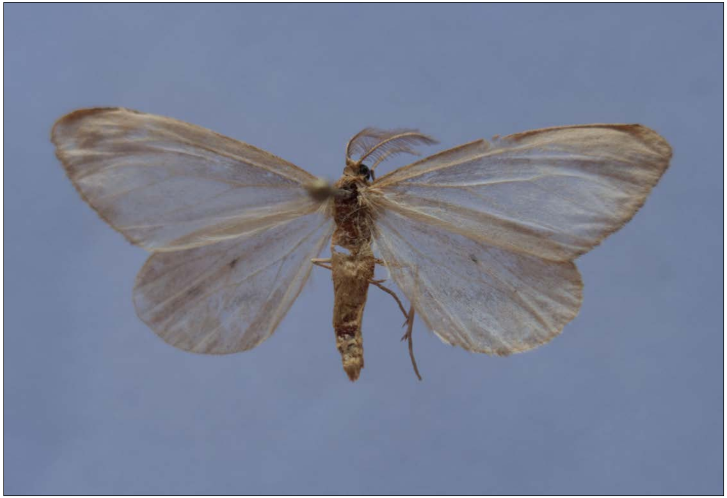

Nephodia sp (TL:)

BC

BOLD:AAE3952	
OTU-45	

Additional compared specimen
near Pe-Geo-0377|Peru|Cuzco|BOLD:AAP8432

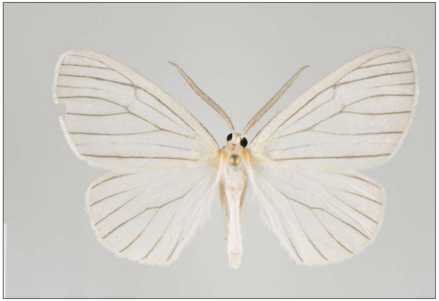

LMR-Geo-
0365, 0374

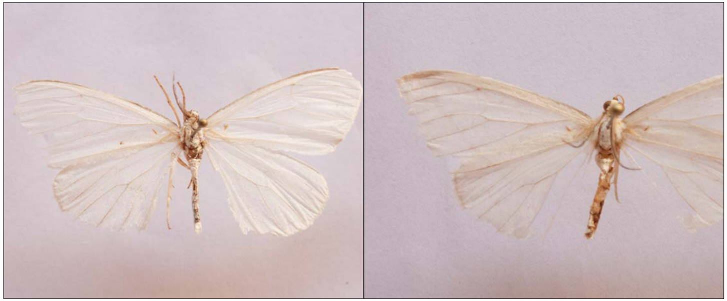

Compared specimen:

LMR-Geo-
0362

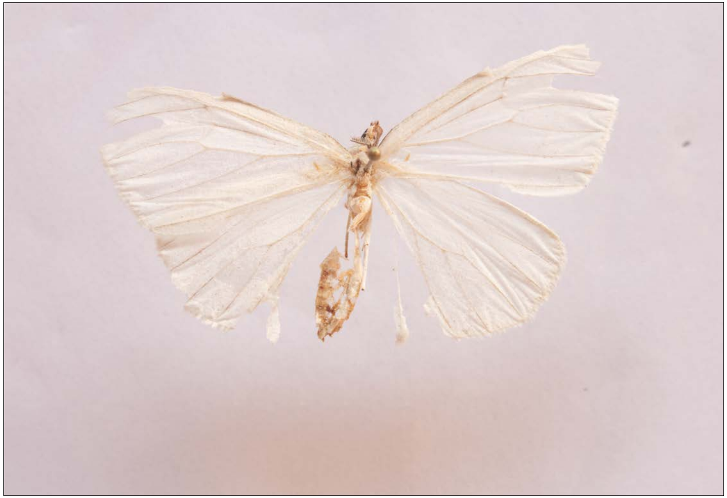

Nephodia sp (TL:)

BC

BOLD:AEC0697
OTU-11

Additional compared specimen

LMR-Geo-

Compared specimen:

LMR-Geo-

0314

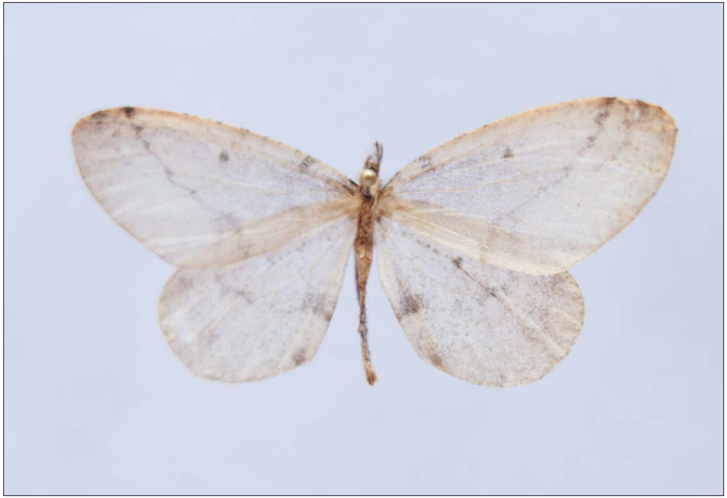

Nephodia sp (TL:)

BC

BOLD:AAM2716	
OTU-58	

Additional compared specimen
= Ec-Geo-22057|Ecuador|Zamora Chinchipe

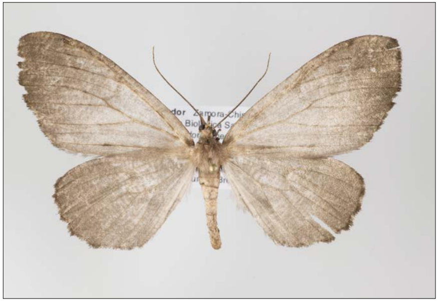

LMR-Geo-

Compared specimen:

LMR-Geo-
0379 (no BIN)

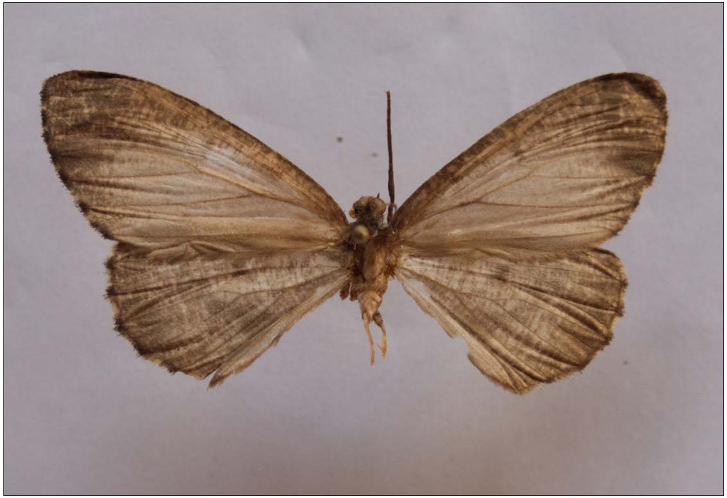

Opisthoxia nr corinnoides Thierry-Mieg (TL: Peru)

BC

BOLD:AEC1261
OTU-4

Additional compared specimen
near Pe-Geo-1261|Peru|Huanuco|BOLD:AAK5714

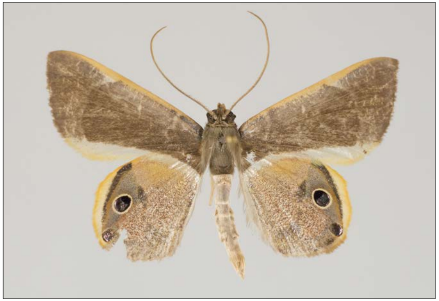

LMR-Geo-

Compared specimen:
no photo available

LMR-Geo-
0306

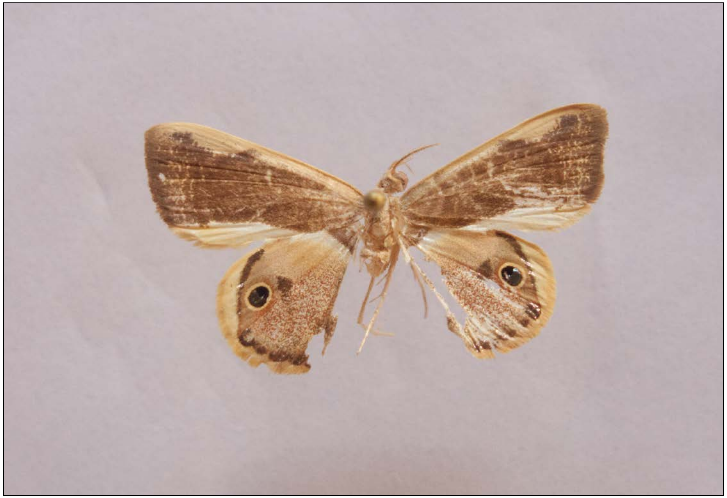

Opisthoxia metargyria Walker (TL: [Colombia]: Bogota)

BC

BOLD:AAW1086
OTU-119

Additional compared specimen
= Ec-Geo-22085|Ecuador|Zamora Chinchipe

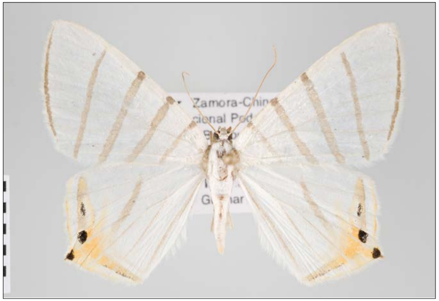

LMR-Geo-

Compared specimen:

NHM type

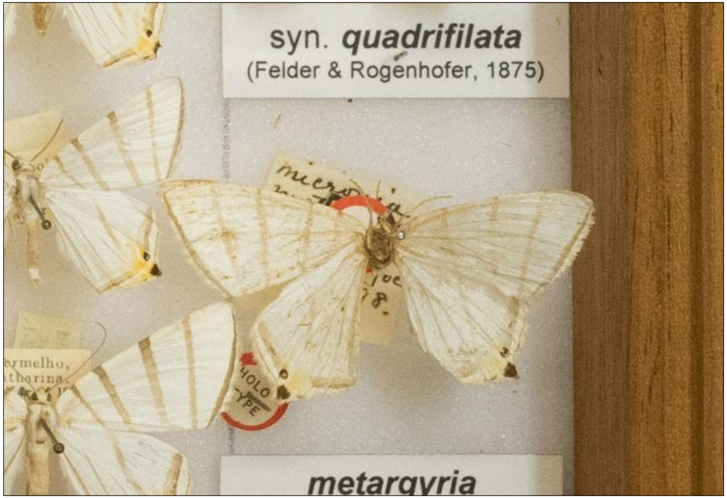

LMR-Geo-
0072

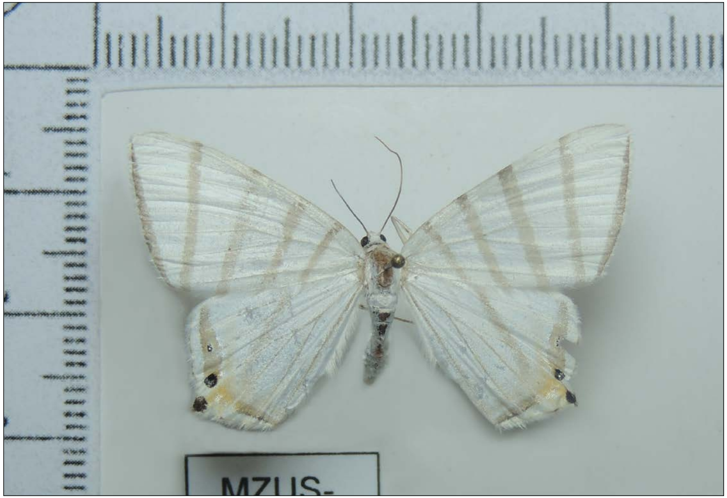

Oxydia augusta group Druce (TL: Panama: Volcan, de Chiriqui, 2000-3000 ft; Ecuador)

BC
BOLD:AAC8450
OTU-212

Additional compared specimen
near: Ec-Geo-22435|Ecuador|Zamora Chinchipe|BOLD:AEE5057

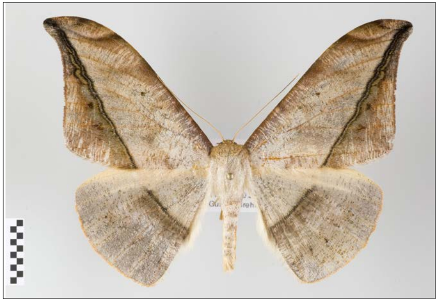

LMR-Geo-

Compared specimen:
NHM type

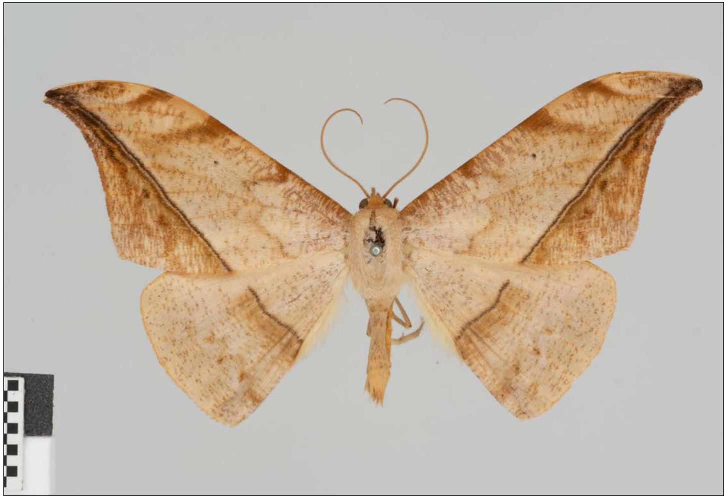

LMR-Geo-
0293

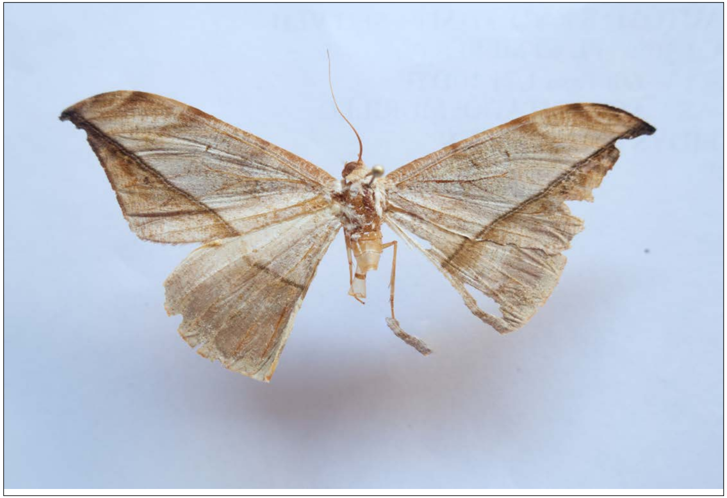

Oxydia nr distichata Guenée (TL: Brazil)

BC

BOLD:AEE9550

OTU-72

Additional compared specimen
distant Ec-Geo-19350|Ecuador|Zamora ChinchipeIBOLD:AAC0627

LMR-Geo-

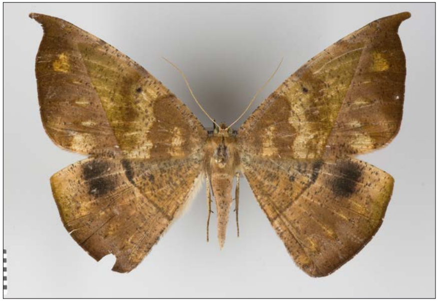

Compared specimen:

NHM type

LMR-Geo-

0024

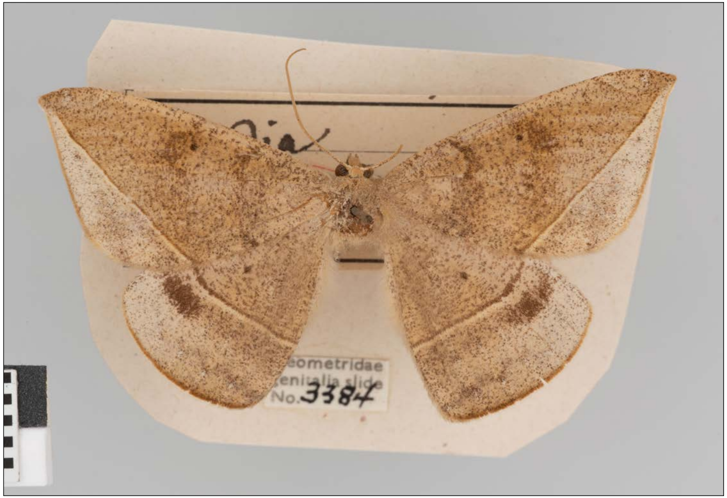

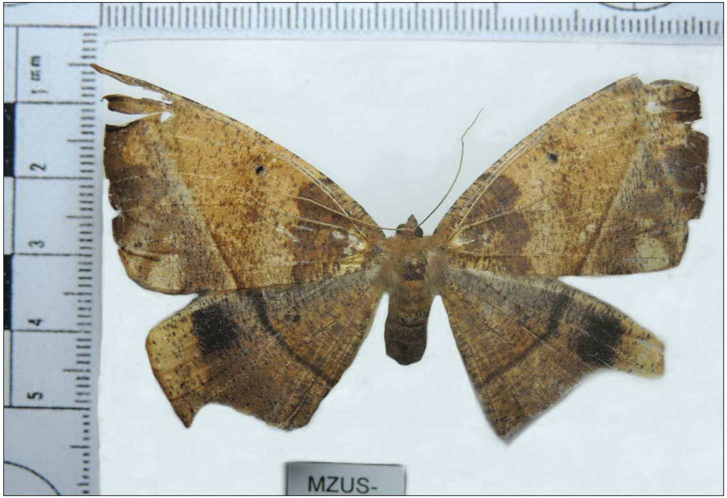

Oxydia nr herbertina Dognin (TL: [Ecuador]: near Loja, Sarajacu)

BC

BOLD:AEB7977
OTU-54

Additional compared specimen
near: Pe-Geo-0066|Peru|Cuzco|BOLD:AAZ8876

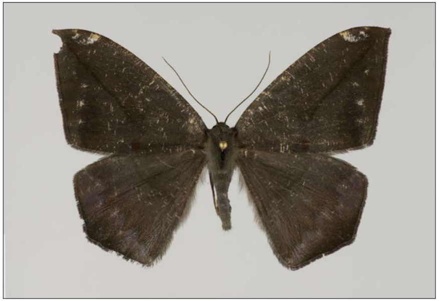

LMR-Geo-

Compared specimen:
USNM type

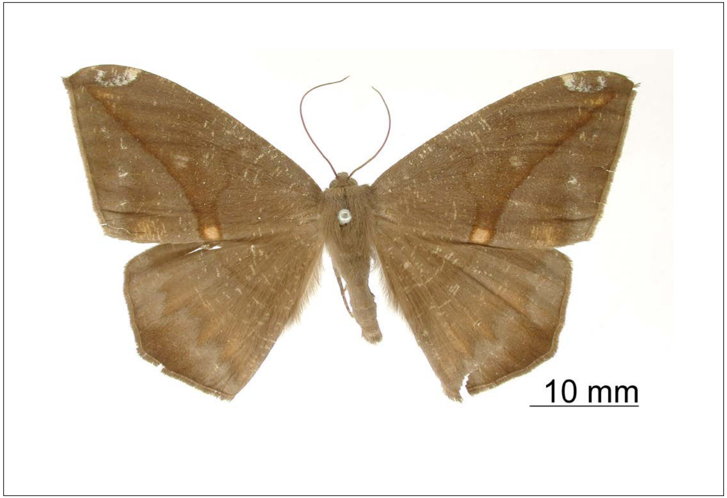

LMR-Geo-
0372

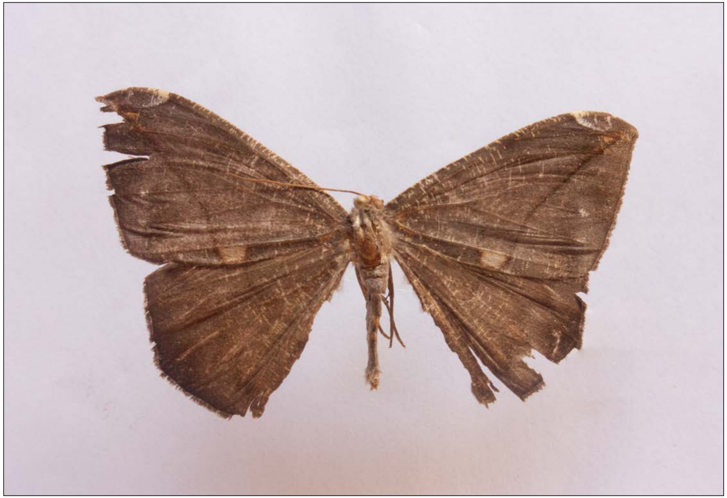

Oxydia olivata Dognin (TL: Bolivia)

BC

|BOLD:AAF2393

OTU-108

Additional compared specimen
= Ec-Geo-19270|Ecuador|Zamora Chinchipe

LMR-Geo-

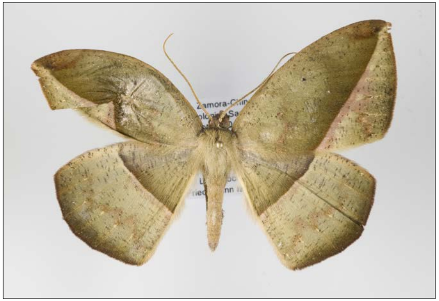

Compared specimen:

NHM type of synonym

LMR-Geo-

0066

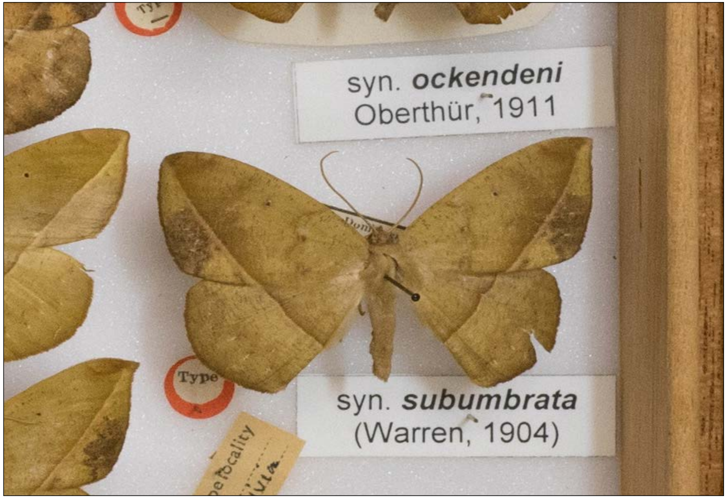

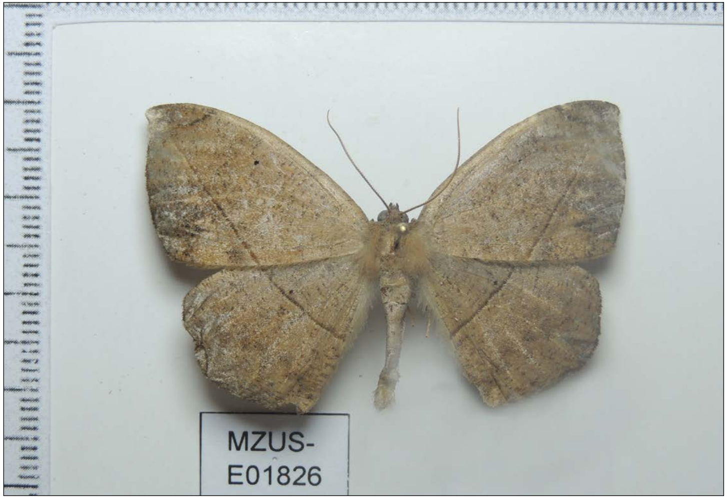

Oxydia nimbata Guenée [Ecuador]: [TL: Brazil]: Rio-Janeiro [Rio de Janeiro]) could also be *sociata* Warren

BC

BOLD:ABY8527

OTU-53

Additional compared specimen
= Ec-Geo-43295|Ecuador|Zamora Chinchipe

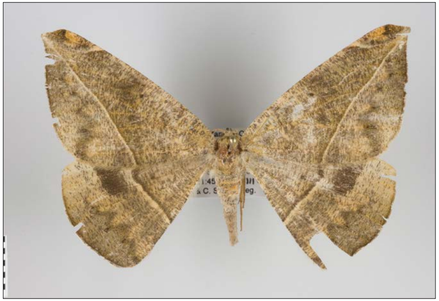

LMR-Geo-
0378

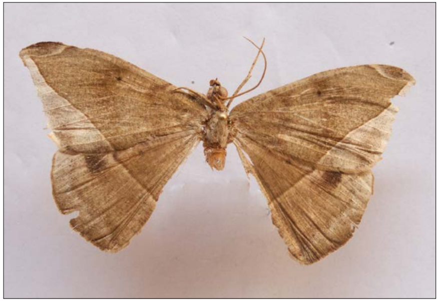

Compared specimen:
NHM type of *nimbata*

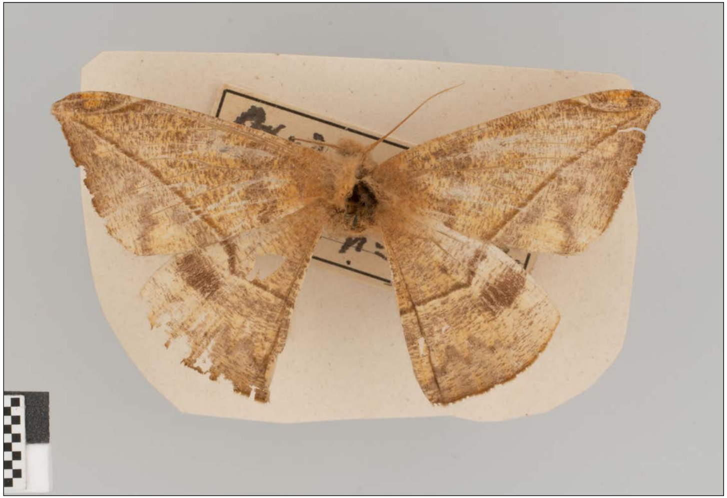

LMR-Geo-
0373

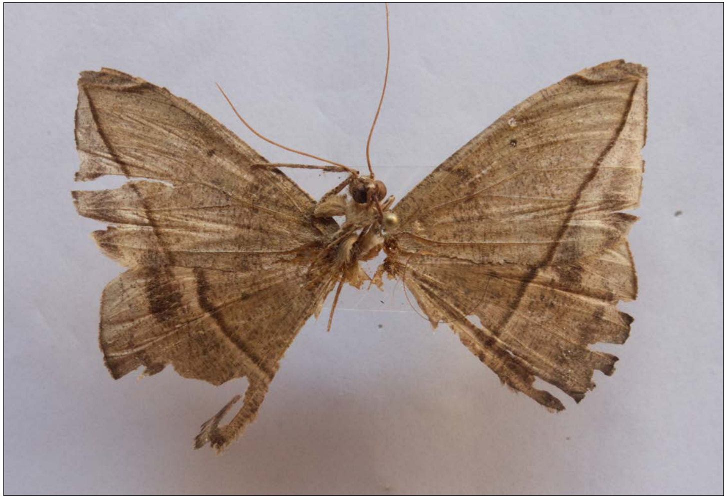

Oxydia scriptipennaria group Walker (TL: Venezuela)

BC

|BOLD:AEE1511

OTU-214

Additional compared specimen
near: Ec-Geo-22243|Ecuador|Zamora Chinchipe|BOLD:AAE3861

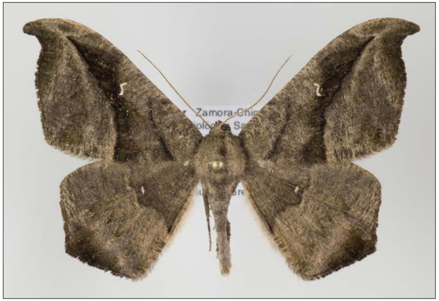

LMR-Geo-

Compared specimen:

NHM type

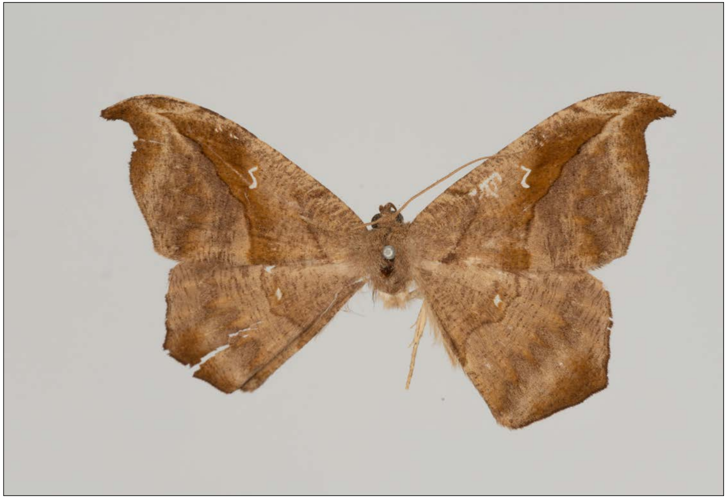

LMR-Geo-

0296

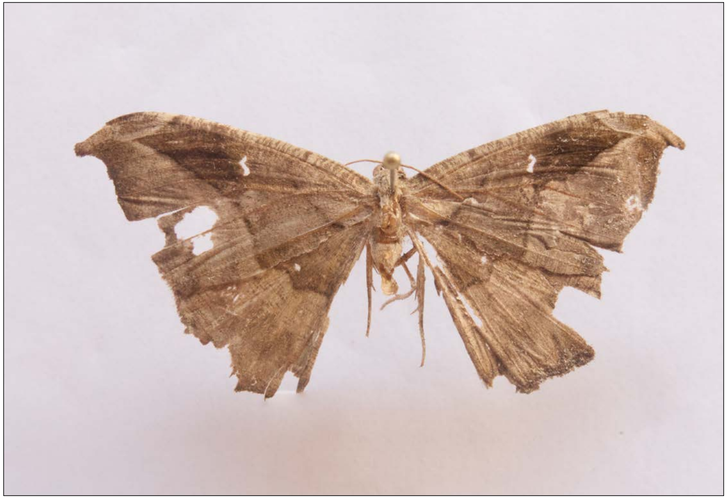

Oxydia trychiata group Guenée (TL: Brazil)

BC

BOLD:AAC5864
OTU-73

Additional compared specimen
= Ec-Geo-22099|Ecuador|Zamora Chinchipe

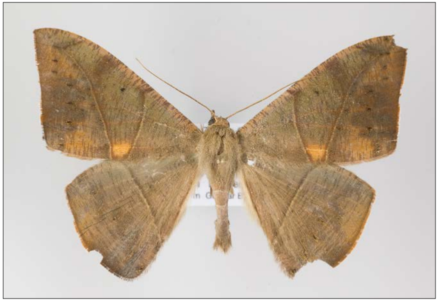

LMR-Geo-

Compared specimen:

NHM type

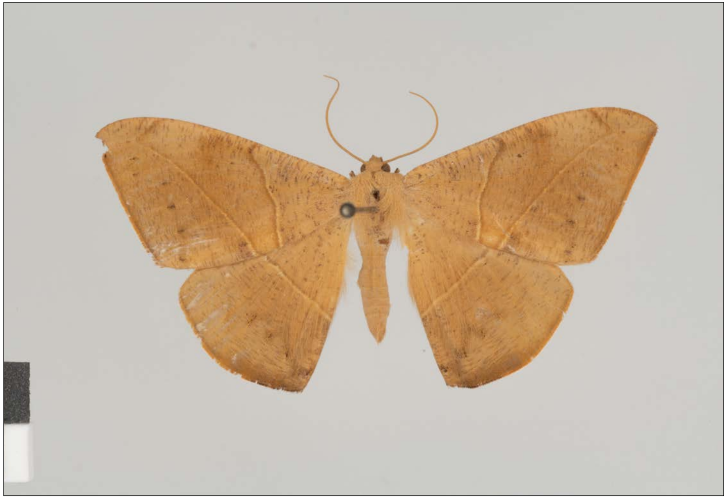

LMR-Geo-

0025

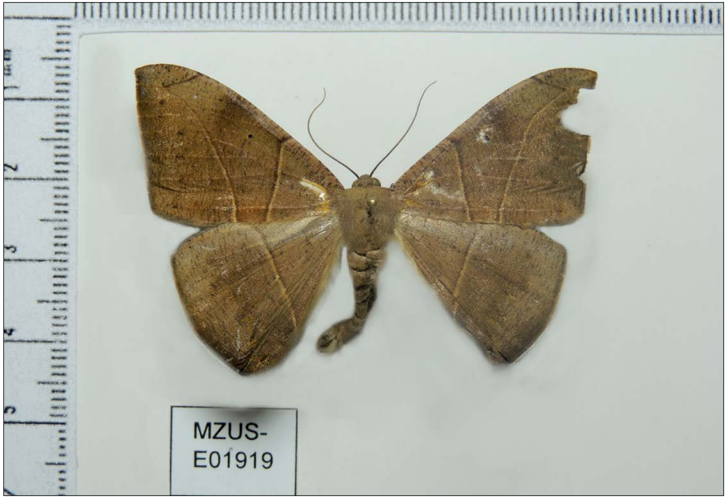

Oxydia trychiata group Guenée (TL: Brazil)

BC

BOLD:AEF0255

OTU-161

Additional compared specimen

near Ec-Geo-18366|Ecuador|Zamora Chinchipe|BOLD:AAA9917

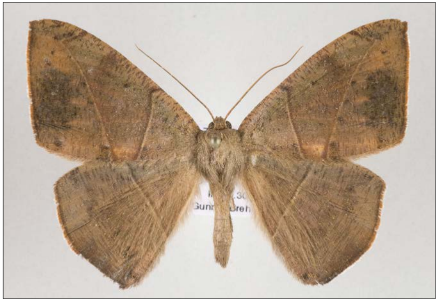

LMR-Geo-

Compared specimen:

NHM type

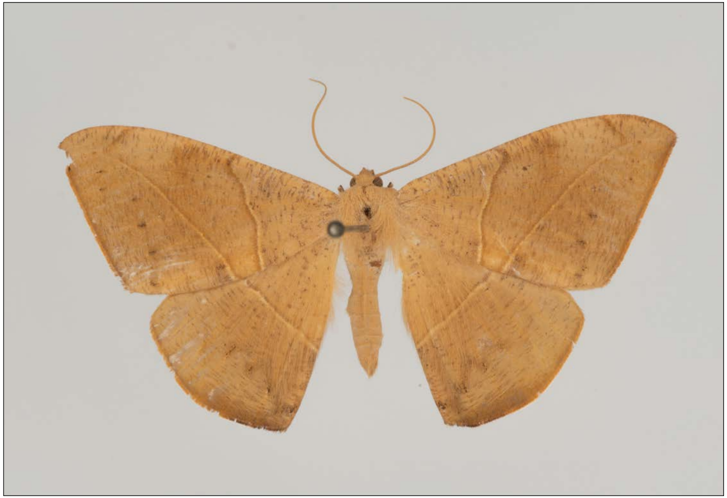

LMR-Geo-

0209

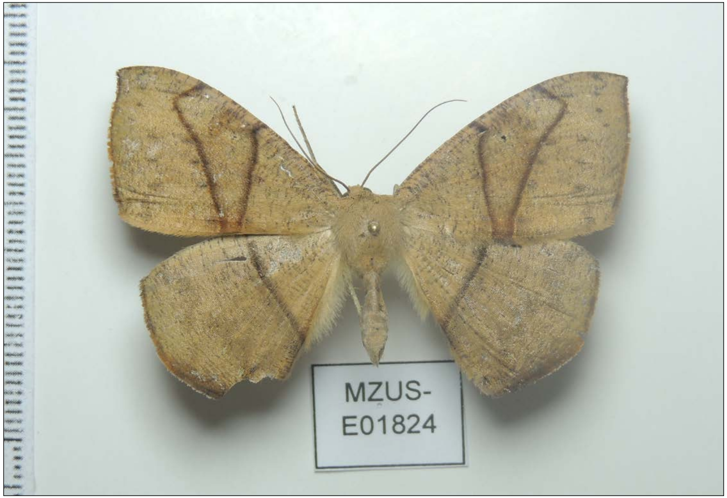

Paragonia cruraria group Herrich-Schäffer (TL: Surinam)

BC

BOLD:AEE1530
OTU-128

Additional compared specimen

LMR-Geo-

Compared specimen:

NHM type of synonym

LMR-Geo-

0138

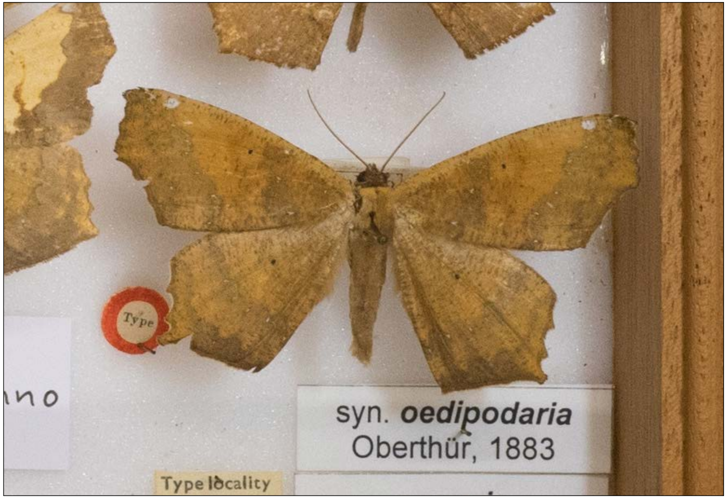

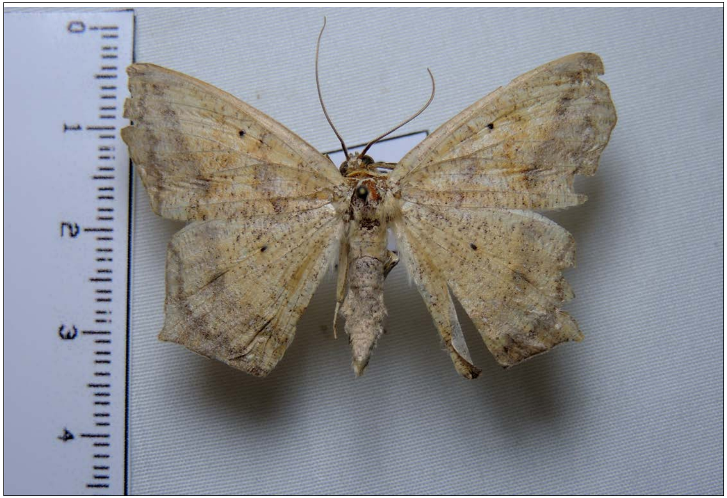

Paragonia cruraria group Herrich-Schäffer (TL: Surinam)

BC

BOLD:ACE9929

OTU-36

Additional compared specimen

LMR-Geo-

Compared specimen:

NHM type of synonym

LMR-Geo-

0348

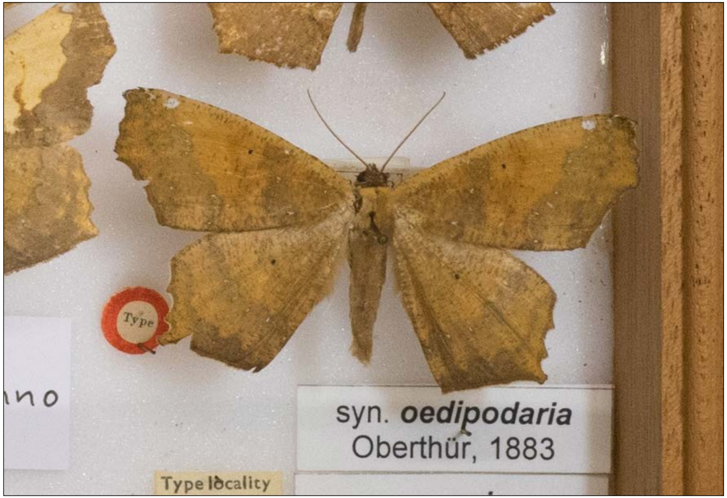

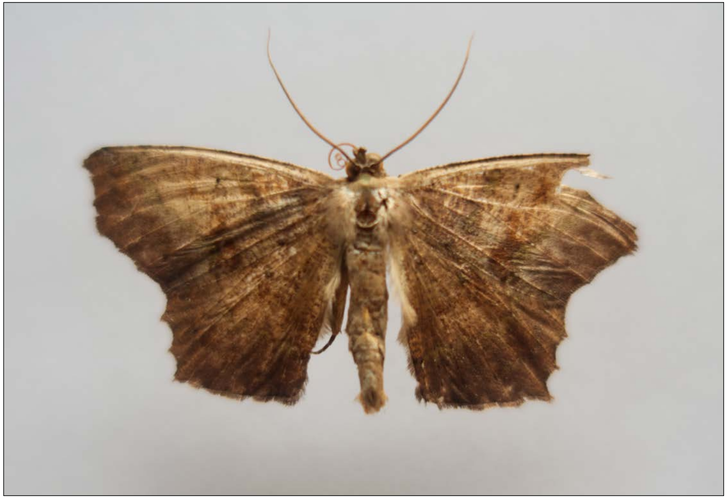

Parilexia nicetaria group Guenée (TL: Haiti)

BC

BOLD:AAA7651
OTU-21

Additional compared specimen
= Pe-Geo-0852|Peru|Huanuco|BOLD:AAA7651

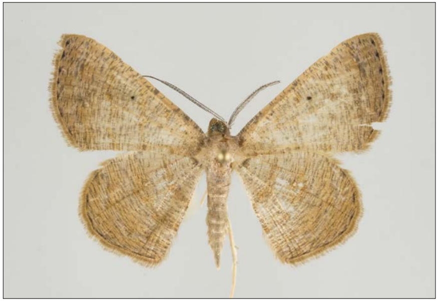

LMR-Geo-
0143

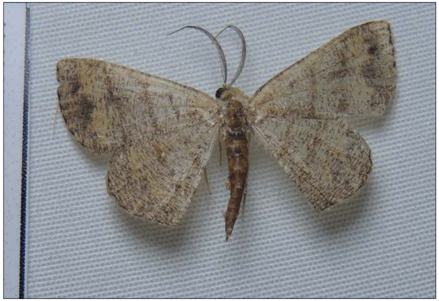

Compared specimen:
NHM type

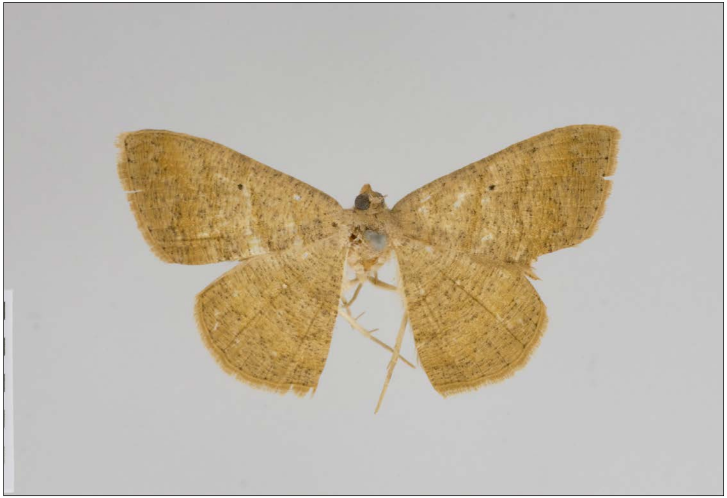

LMR-Geo-
0327

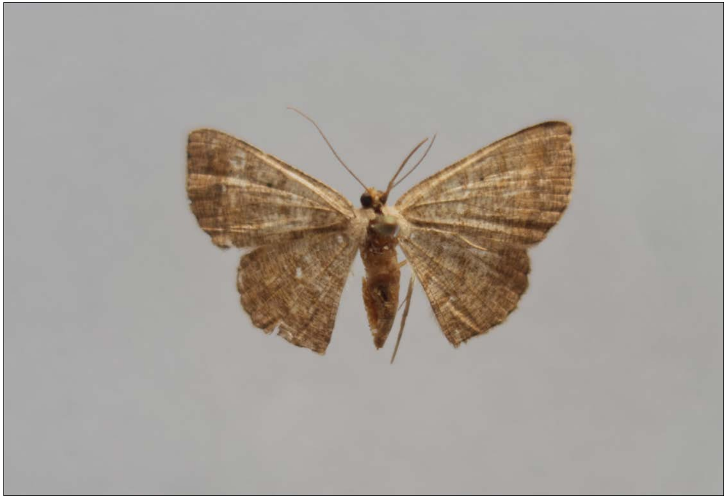

Parilexia nr cermala Druce (TL: Mexico, Guatemala, Costa Rica)

BC

BOLD:ABZ1395
OTU-25

Additional compared specimen
near: Pe-Geo-0404|Peru|Cuzco|BOLD:ADF2333

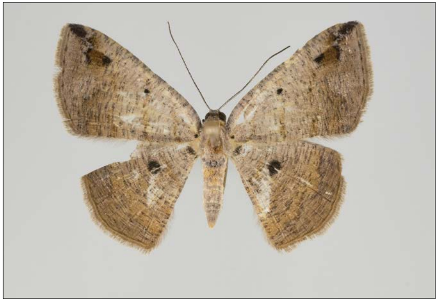

LMR-Geo-

Compared specimen:
NHM type

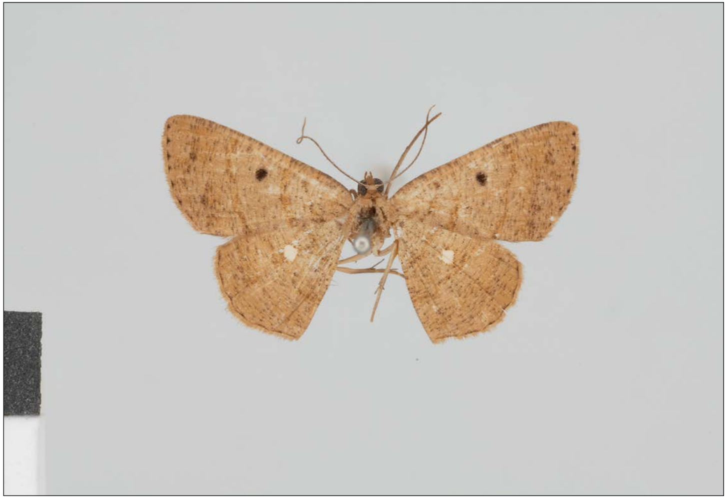

LMR-Geo-
0331

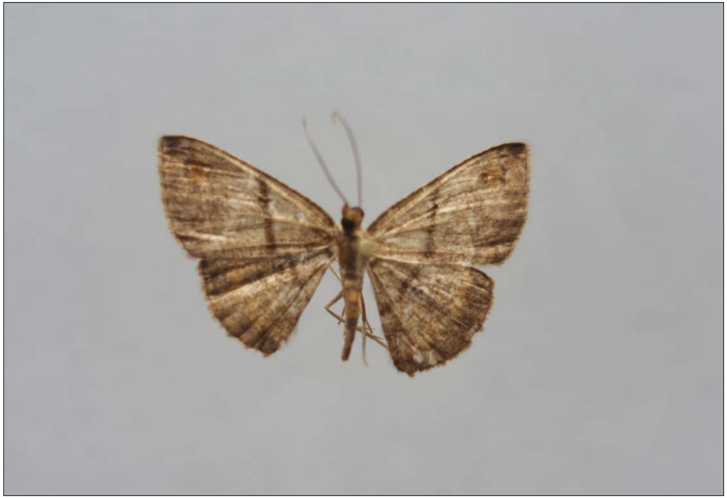

Perissopteryx sp (TL:)

BC
BOLD:AAC9368
OTU-48

Additional compared specimen
near: Pe-Geo-3035|Peru|Cuzco|BOLD:ADK4144

LMR-Geo-

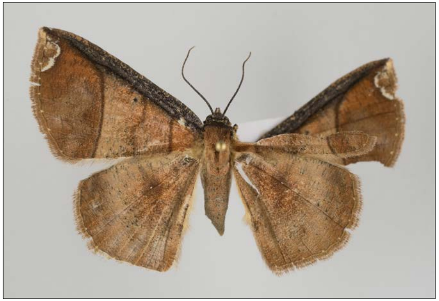

Compared specimen:

LMR-Geo-
0369

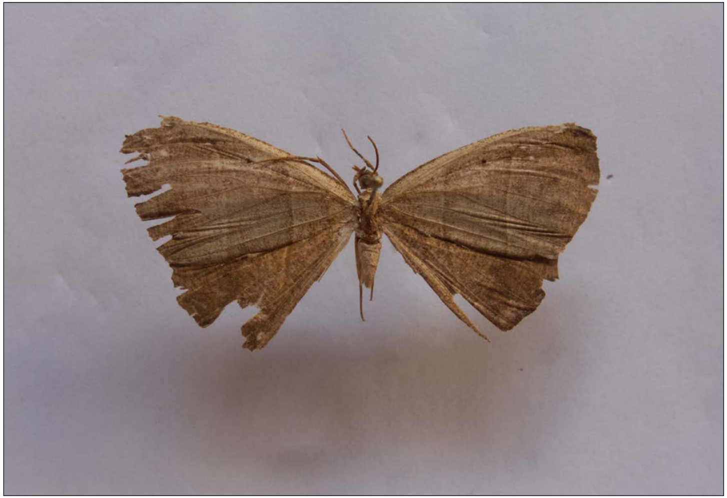

Patalene asychisaria Walker (TL: Brazil)

BC

BOLD:AAA8106	
OTU-18	

Additional compared specimen
= Pe-Geo-3732|Peru|Cuzco|BOLD:AAA8106

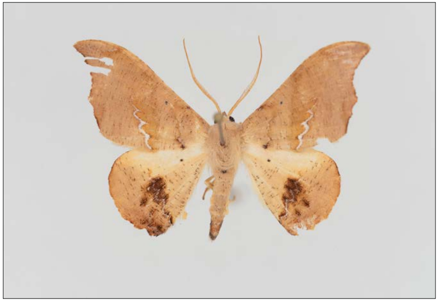

LMR-Geo-

Compared specimen:
NHM type

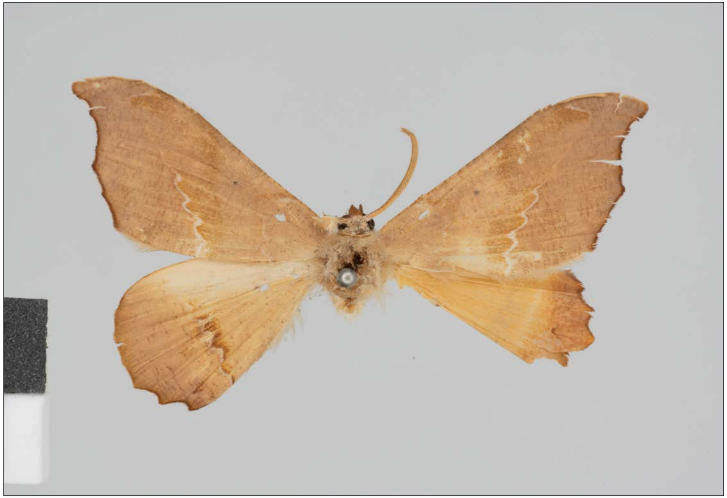

LMR-Geo-
0326

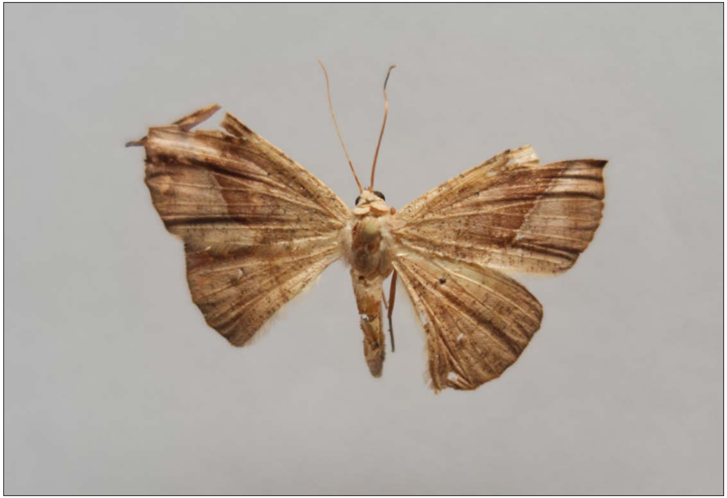

Periclina apricaria Herrich -Schäffer (TL: Venezuela)

BC

BOLD:AAU0219
OTU-95

Additional compared specimen

LMR-Geo-
0051, 0044

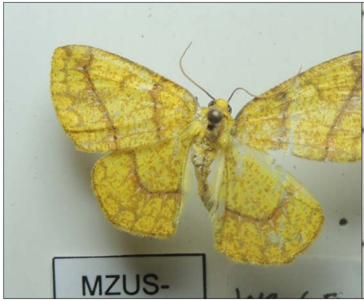

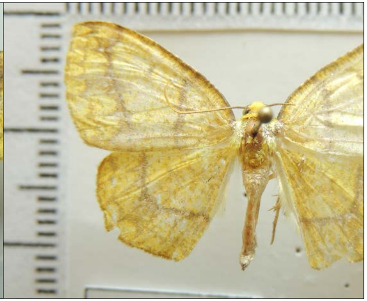

Compared specimen:
NHM type

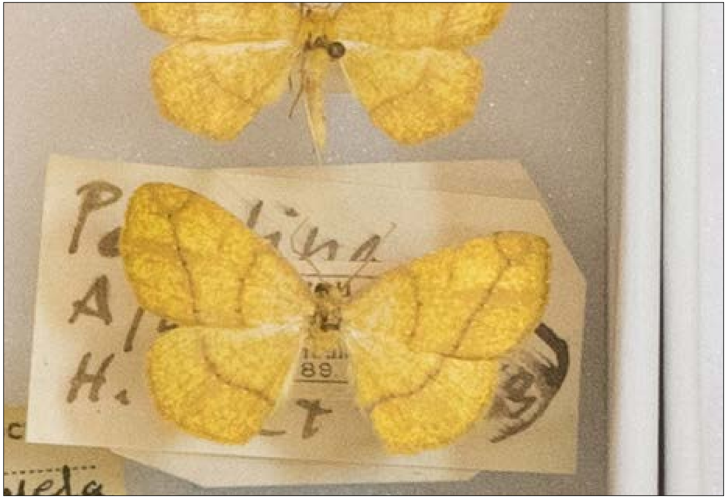

LMR-Geo-
0056

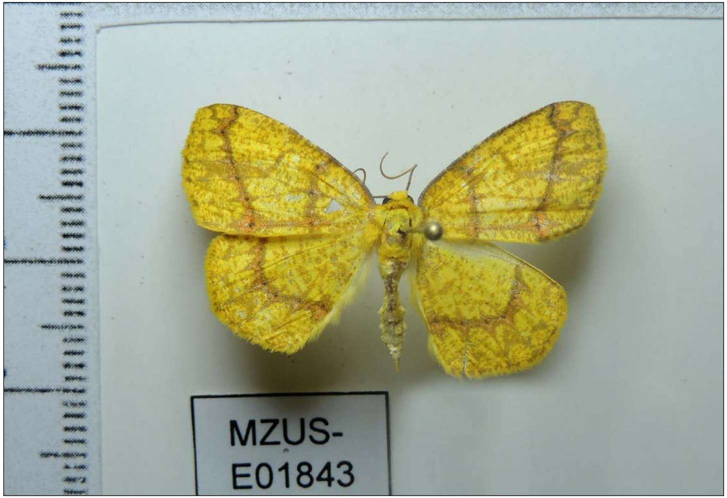

Pero scitaria Oberthür (TL: [Peru]: Tambillo)

BC

BOLD:ABY9748
OTU-201

Additional compared specimen
= Ec-Geo-14801|Ecuador|Zamora Chinchipe

LMR-Geo-

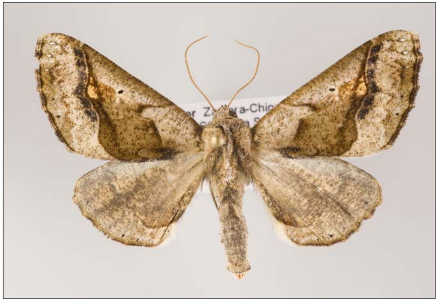

Compared specimen:

NHM type

LMR-Geo-

0246

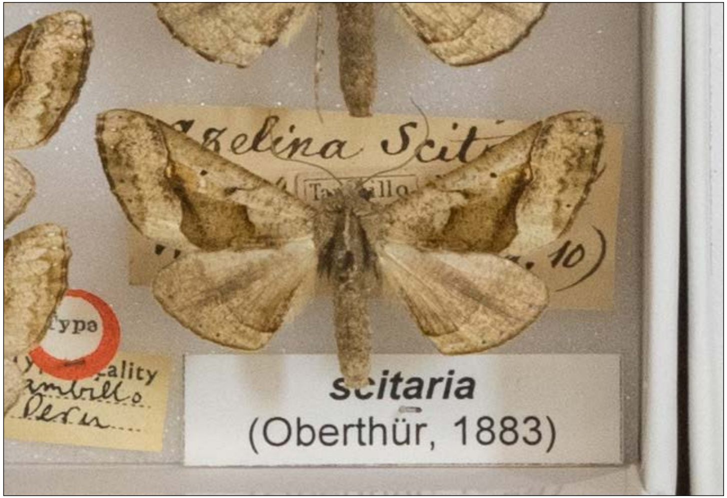

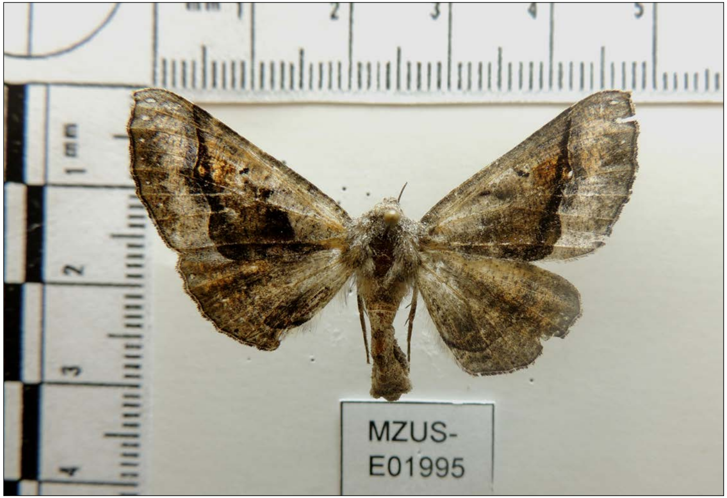

Pero buckleyi Butler (TL: Ecuador)

BC

BOLD:AAD1741

OTU-218

Additional compared specimen
= Ec-Geo-22193|Ecuador|Zamora Chinchipe

LMR-Geo-

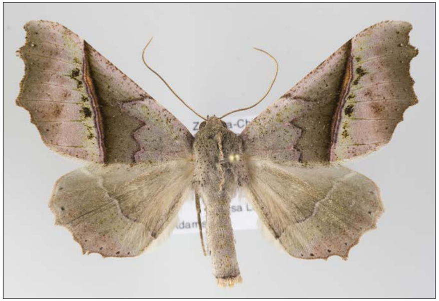

Compared specimen:

NHM type

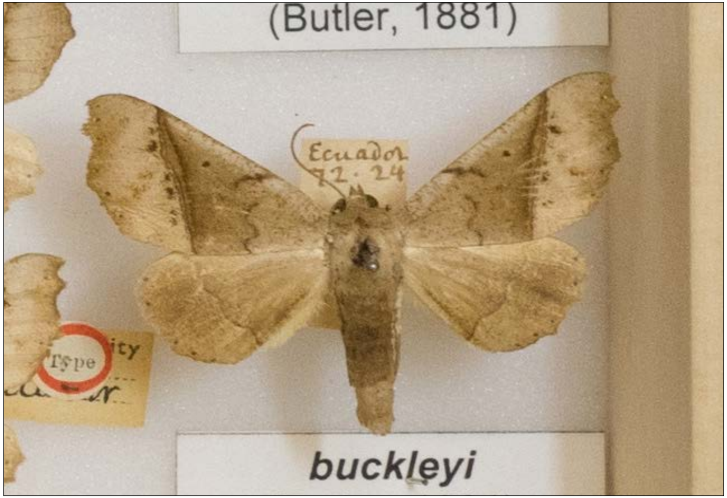

LMR-Geo-

0290

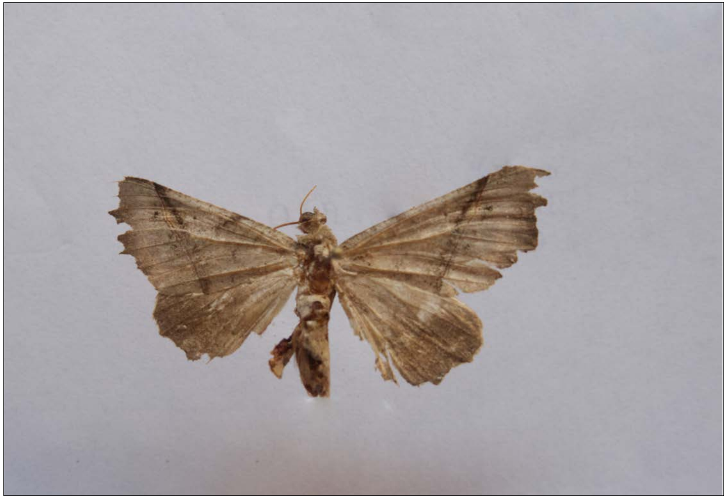

Perusia nr verticata Warren (TL: Peru: Cerro de Pasco, Huancabamba, 6400 ft)

BC

BOLD:AEE6694
OTU-89

Additional compared specimen
near: Pe-Geo-0362|Peru|Cuzco|BOLD:ADF1637

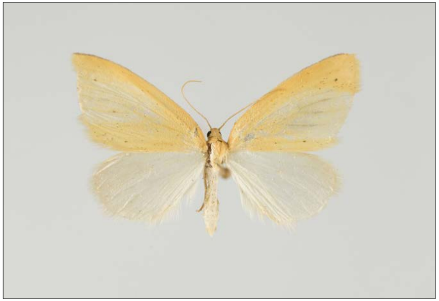

LMR-Geo-

Compared specimen:
NHM type

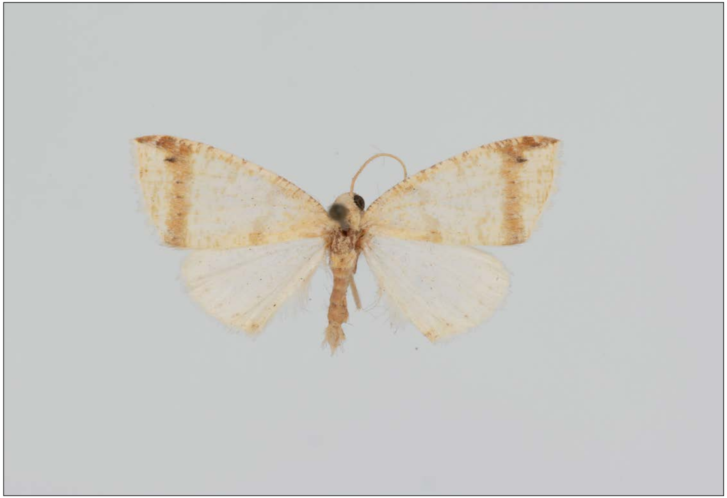

LMR-Geo-
0046

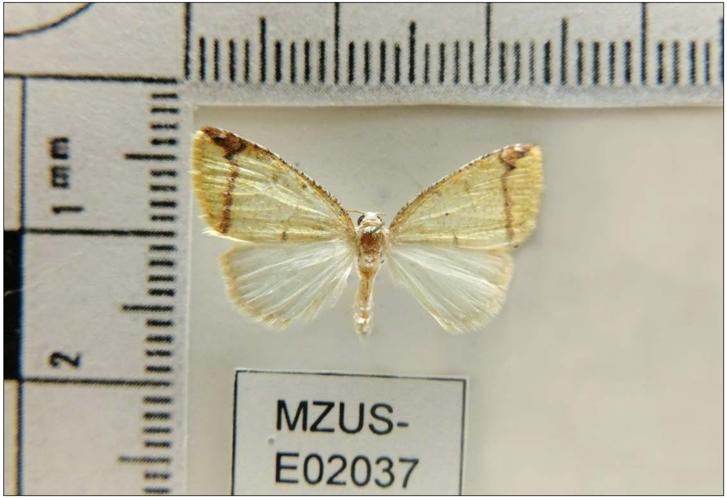

Perusia nr verticata Warren (TL: Peru: Cerro de Pasco, Huancabamba, 6400 ft)

BC
BOLD:AEE3906
OTU-88

Additional compared specimen
near: Ec-Geo-15452|Ecuador|Zamora Chinchipe|BOLD:AAI2456

LMR-Geo-

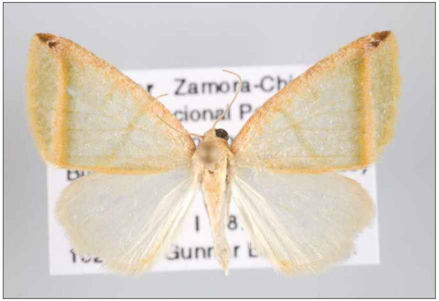

Compared specimen:
NHM type

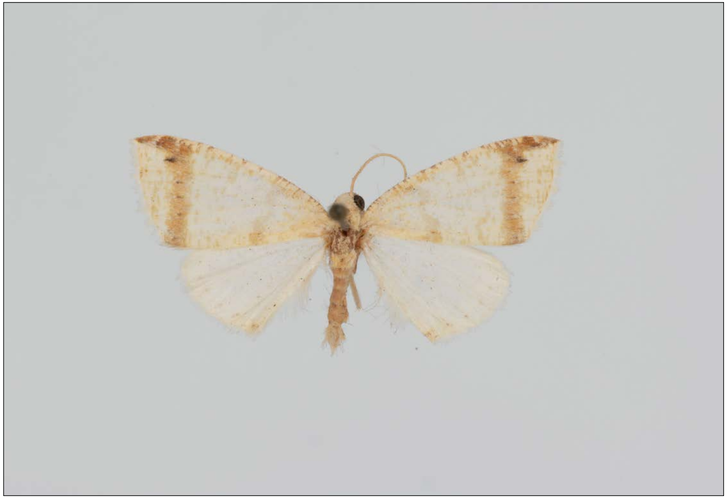

LMR-Geo-
0047

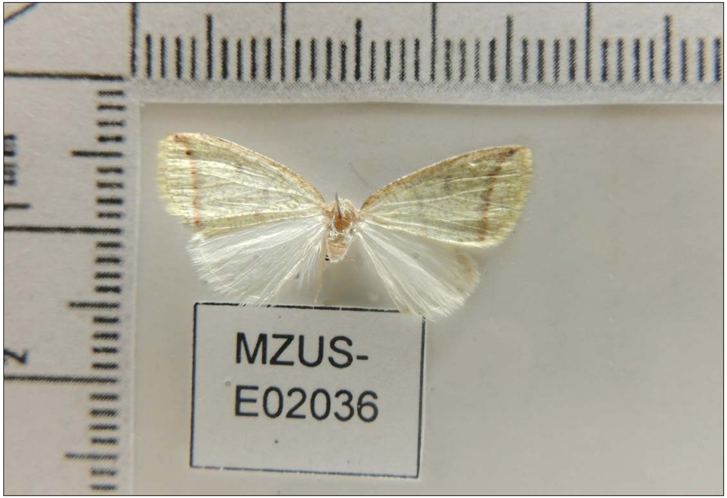

Perusia nr zoma Dognin (TL: [Ecuador]: Loja)

BC

no BIN
OTU-90

Additional compared specimen

near Ec-Geo-18158|Ecuador|Loja|BOLD:AAM6916

LMR-Geo-

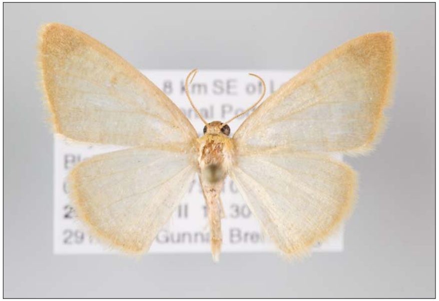

Compared specimen:

USNM type

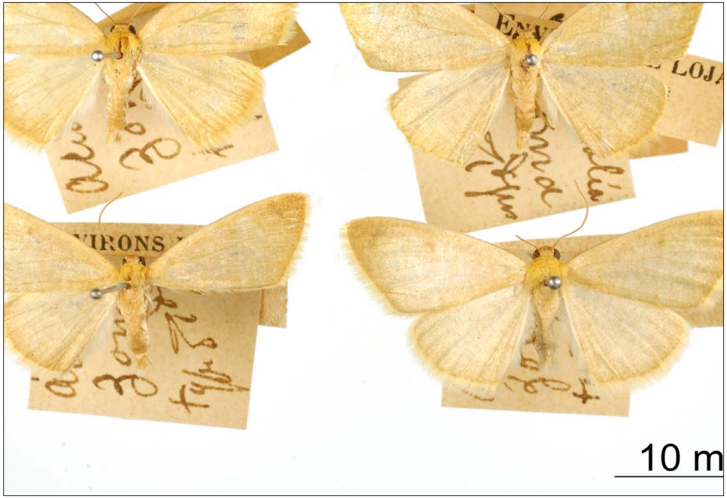

LMR-Geo-

0049

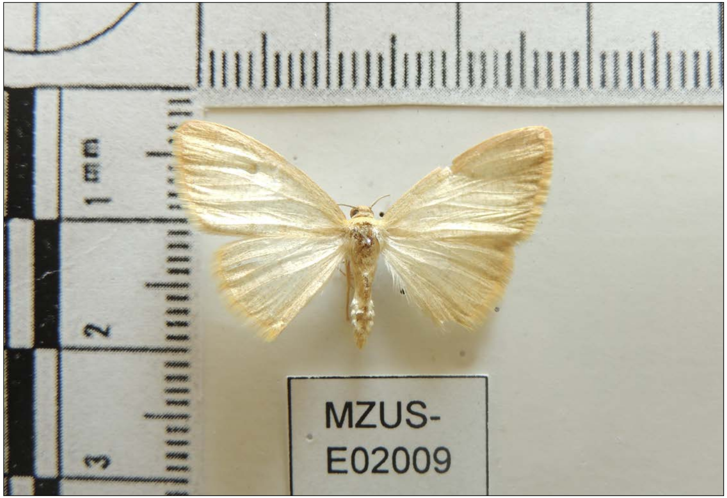

Phyllodonta succedens group Walker (TL: [Colombia], Bogota; [Ecuador], Quito)

BC
BOLD:AAE0170
OTU-208

Additional compared specimen
= Ec-Geo-22064|Ecuador|Zamora Chinchipe

LMR-Geo-

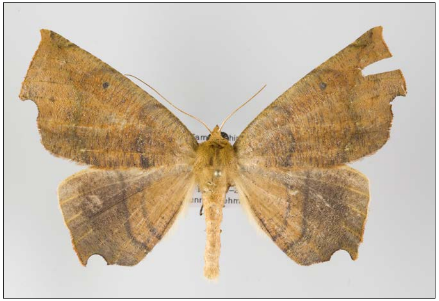

Compared specimen:

NHM type

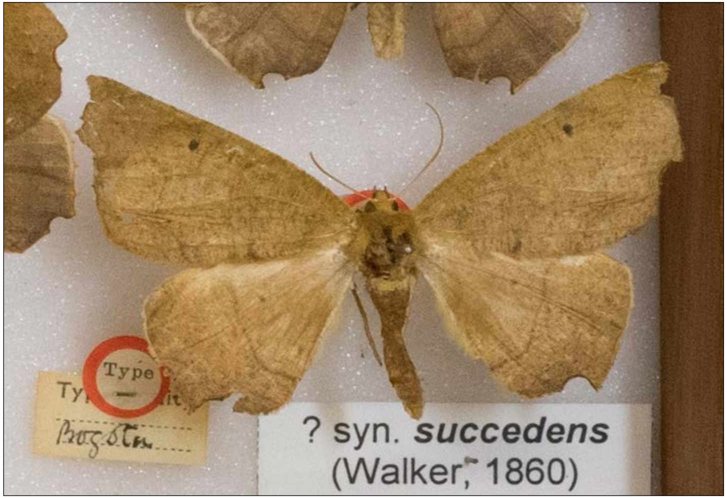

LMR-Geo-

0301

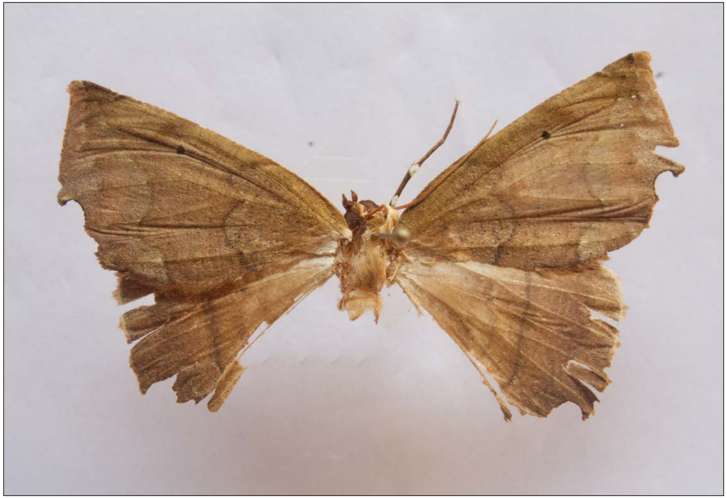

Physocleora sp (TL:)

BC

BOLD:ADU0597
OTU-86

Additional compared specimen
distant: Ec-Geo-19105|Ecuador|Zamora Chinchipe|BOLD:AAY6214

LMR-Geo-

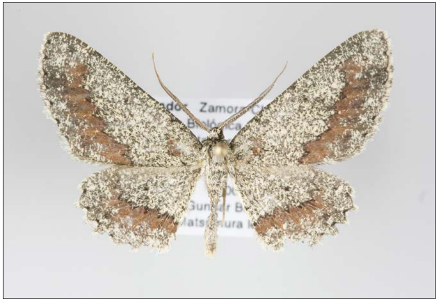

Compared specimen:

LMR-Geo-
0035

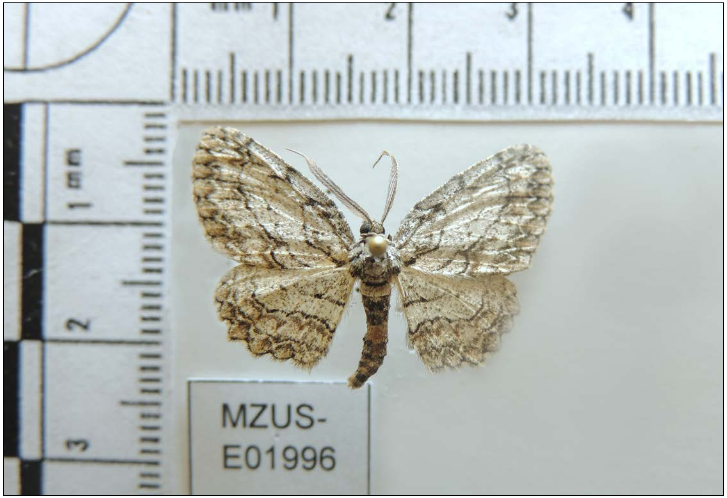

Physocleora sp (TL:)

BC

BOLD:AEE3065

OTU-92

Additional compared specimen
distant: Ec-Geo-19105|Ecuador|Zamora Chinchipe|BOLD:AAY6214

LMR-Geo-

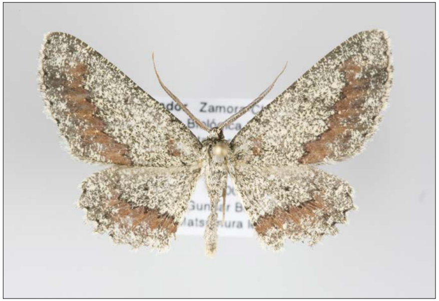

Compared specimen:

LMR-Geo-
0042

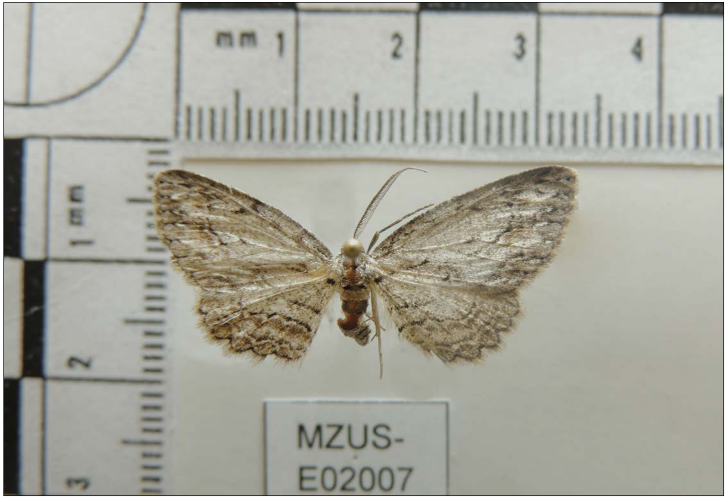

Physocleora sp (TL:)

BC

no BIN
OTU-129

Additional compared specimen

LMR-Geo-
0110 (333bp)

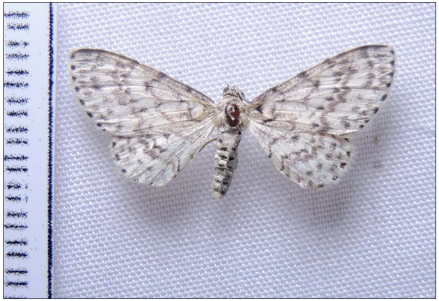

Compared specimen:

LMR-Geo-
0135 (304bp)

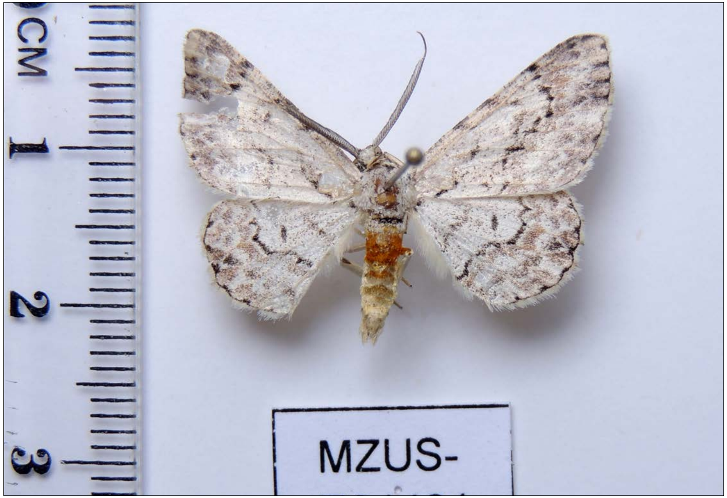

Physocleora sp (TL:)

BC

BOLD:AEE9876
OTU-84

Additional compared specimen

LMR-Geo-
0031

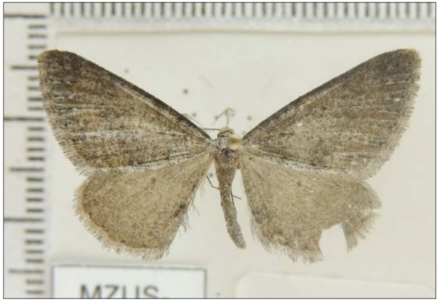

Compared specimen:

LMR-Geo-
0032

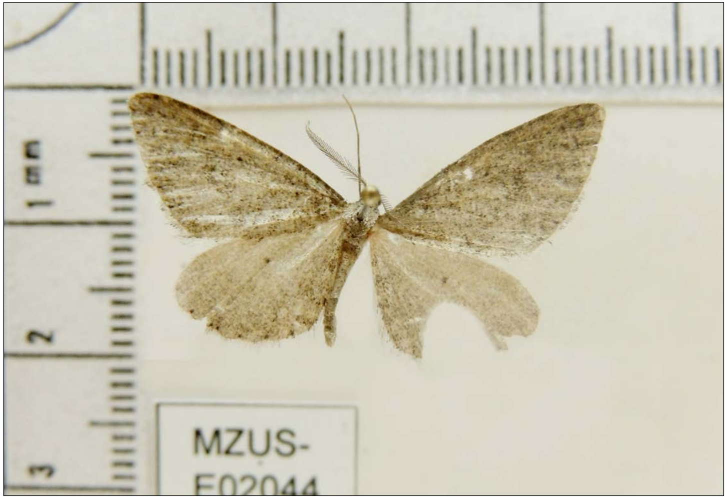

Physocleora sp (TL:)

		BC
		BOLD:AEE9875
		OTU-85

Additional compared specimen

LMR-Geo-0029

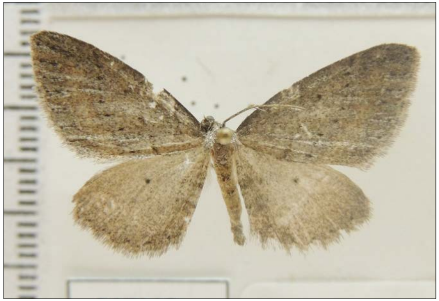

Compared specimen:

LMR-Geo-0030

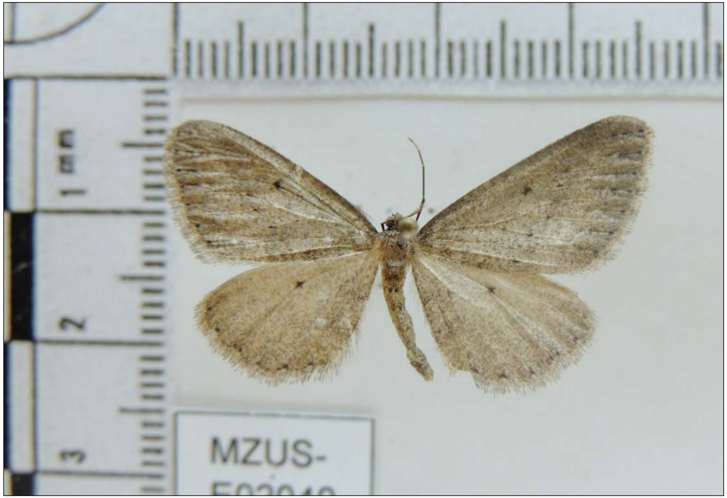

Physocleora sp (TL:)

BC

BOLD:AEE7026
OTU-101

Additional compared specimen
no close relative

LMR-Geo-

Compared specimen:

LMR-Geo-
0055

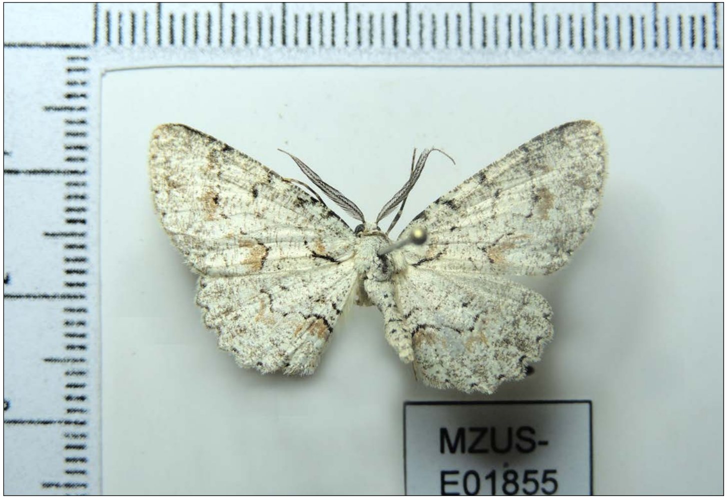

Physocleora sp (TL:)

BC

BOLD:ADT0023
OTU-104

Additional compared specimen
no close relative

LMR-Geo-

Compared specimen:

LMR-Geo-
0070

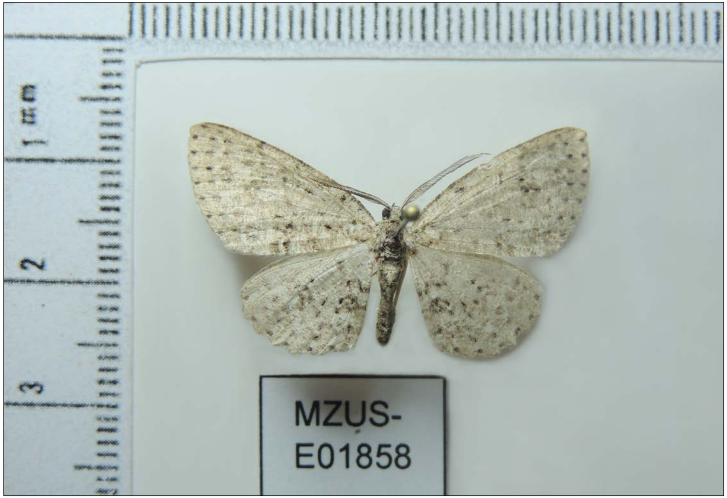

Physocleora sp (TL:)

BC

BOLD:AEE0627
OTU-118

Additional compared specimen
no close relative

LMR-Geo-
0074

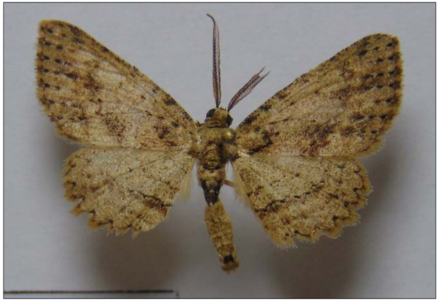

Compared specimen:

LMR-Geo-
0078

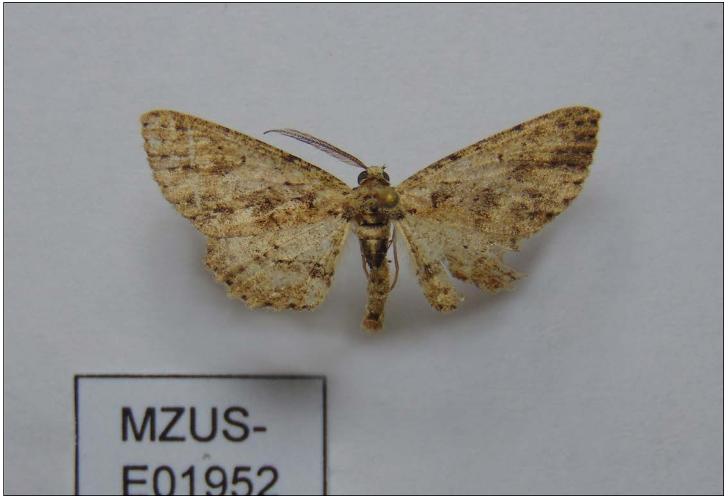

Physocleora sp (TL:)

BC

no BIN
OTU-137

Additional compared specimen
no close relative

LMR-Geo-

Compared specimen:

LMR-Geo-
0110

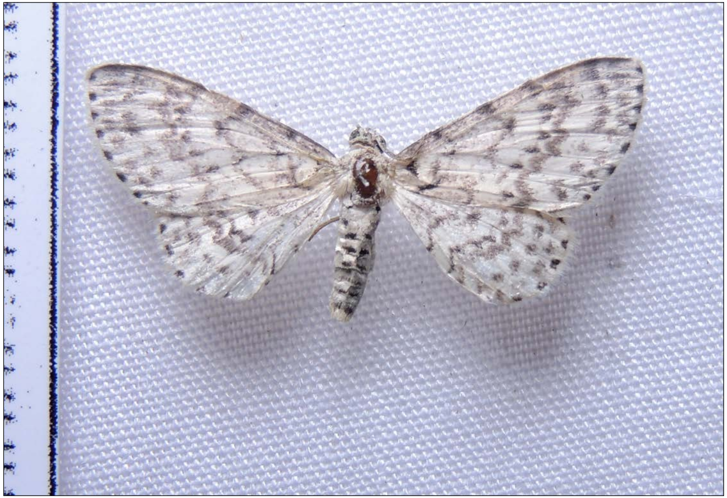

Physocleora sp (TL:)

BC

BOLD:AEE0626
OTU-180

Additional compared specimen
near Ec-Geo-13349|Ecuador|Zamora Chinchipe|BOLD:AAI2939

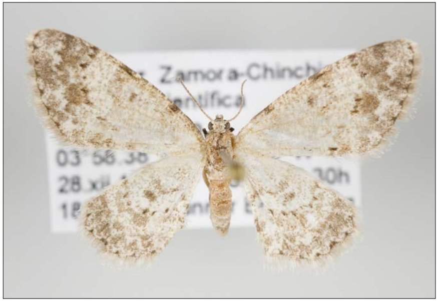

LMR-Geo-

Compared specimen:

LMR-Geo-
0233

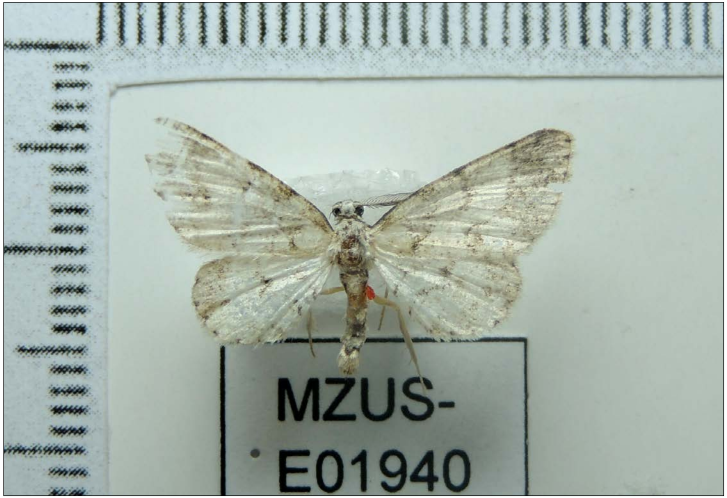

Physocleora sp (TL:)

BC

BOLD:AEE8794

OTU-194

Additional compared specimen
no close relative

LMR-Geo-

Compared specimen:

LMR-Geo-
0262

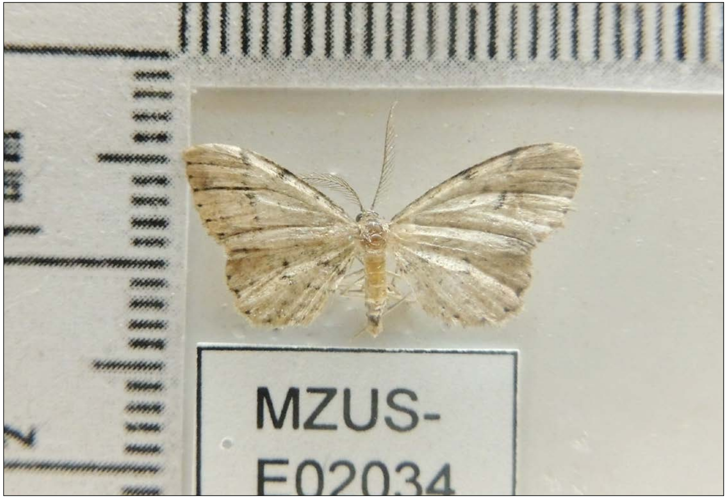

Physocleora (TL:)

BC

BOLD:AEE4831

OTU-217

Additional compared specimen
distant: Ec-Geo-25108|Ecuador|Zamora Chinchipe|BOLD:ACF7088

LMR-Geo-

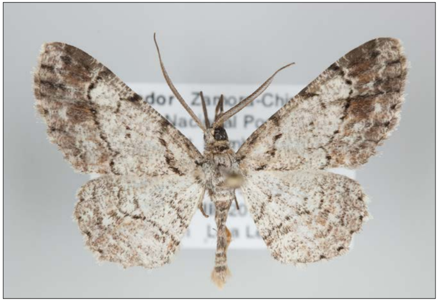

Compared specimen:

LMR-Geo-
0289

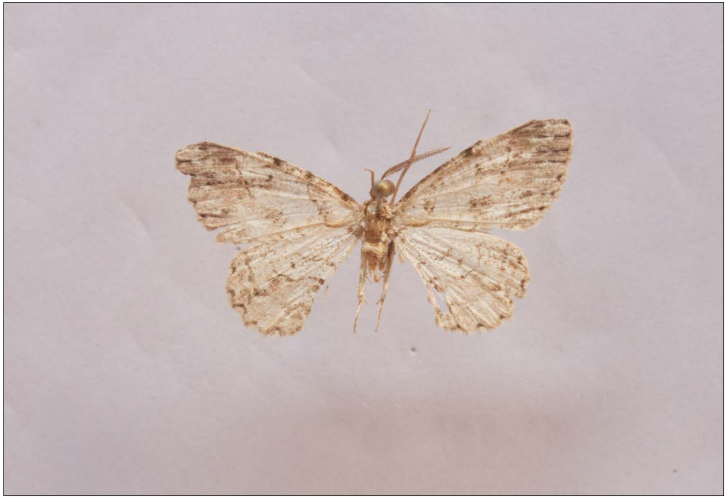

Pityeja histrionaria group Herrich-Schäffer (TL: Venezuela)

BC

BOLD:AAD3605	
OTU-80	

Additional compared specimen
= Ec-Geo-19836|Ecuador|Zamora Chinchipe

LMR-Geo-

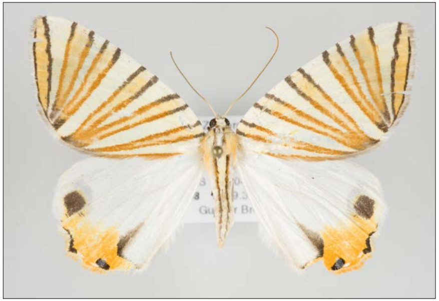

Compared specimen:
NHM type of synonym

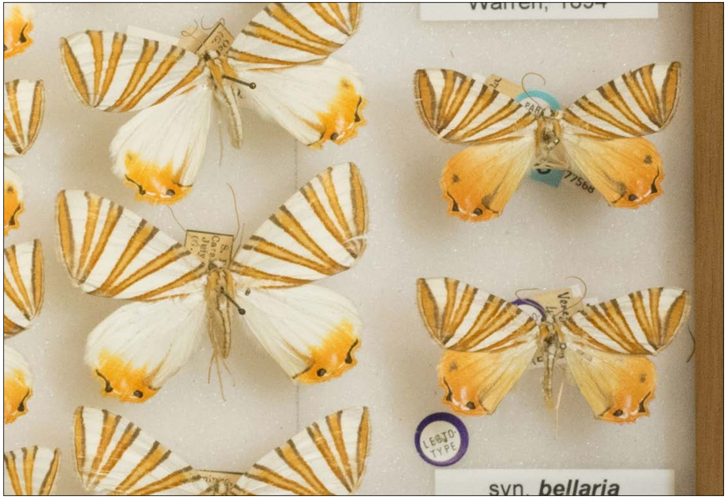

LMR-Geo-
0037

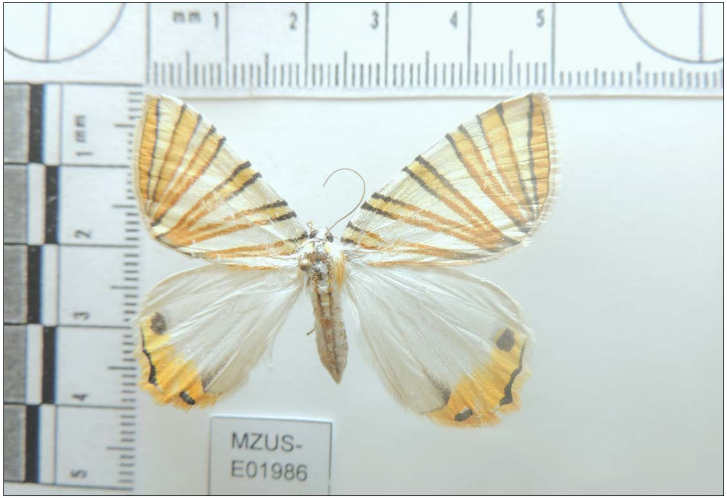

Prochoerodes marciana Druce (TL: Mexico, Guatemala)

BC

BOLD:AAK9639
OTU-31

Additional compared specimen

LMR-Geo-

Compared specimen:

NHM type

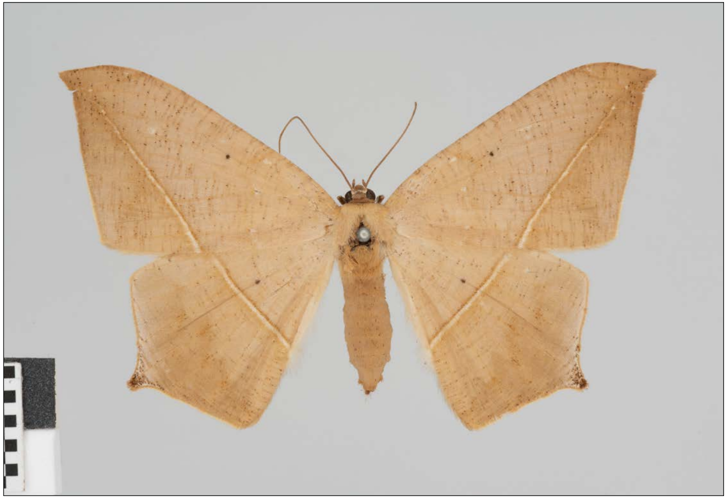

LMR-Geo-

0338

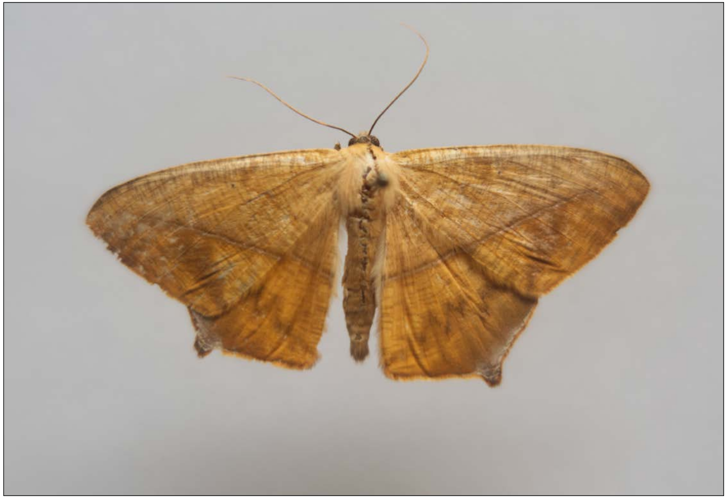

Prochoerodes striata group Stoll (TL: Surinam)

BC

BOLD:AAC9373
OTU-30

Additional compared specimen

LMR-Geo-

Compared specimen:

NHM type of synonym

LMR-Geo-

0339

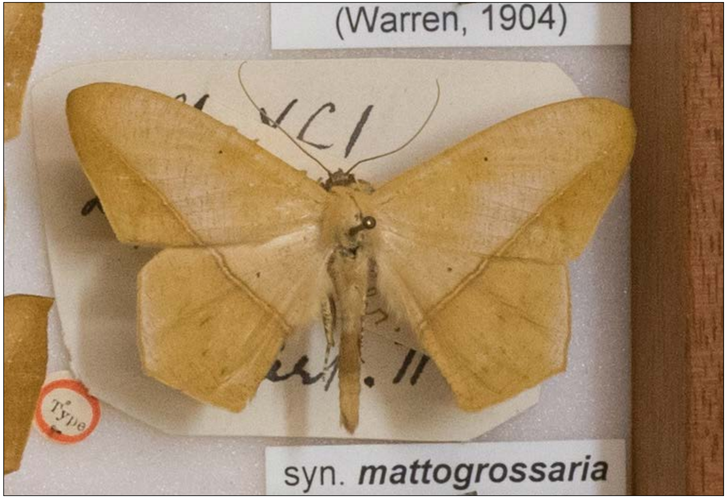

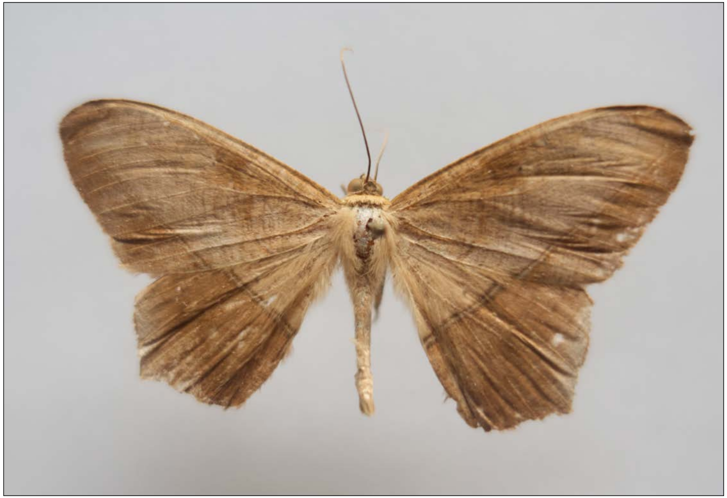

Rucana sp (TL:)

BC

BOLD:AEC0740

OTU-13

Additional compared specimen
distant: Ec-Geo-22841|Ecuador|Zamora Chinchipe|BOLD:AAM6917

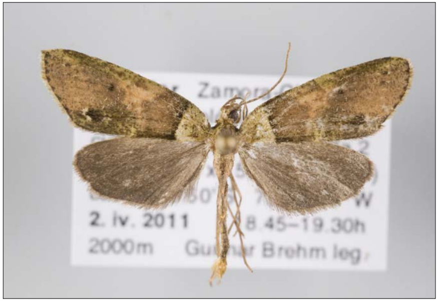

LMR-Geo-

Compared specimen:

LMR-Geo-
0318

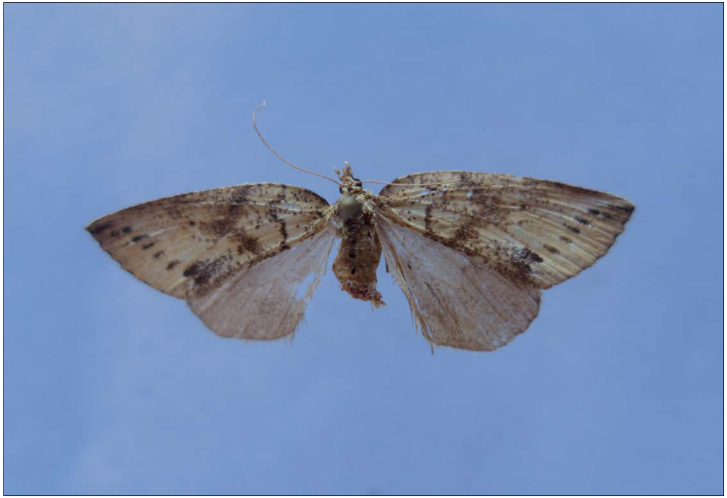

Sabulodes caberata oberthuri Rindge (TL: Ecuador: Pichincha)

BC
BOLD:AAE1766
OTU-117

Additional compared specimen
= Ec-Geo-22016|Ecuador|Zamora Chinchipe|BOLD:AAE1766

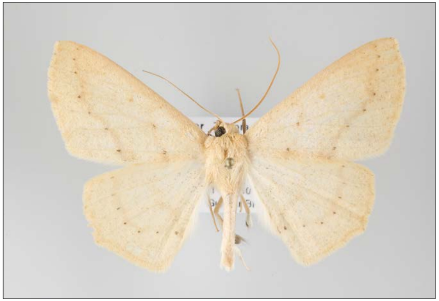

LMR-Geo-

Compared specimen:

NHM syntype

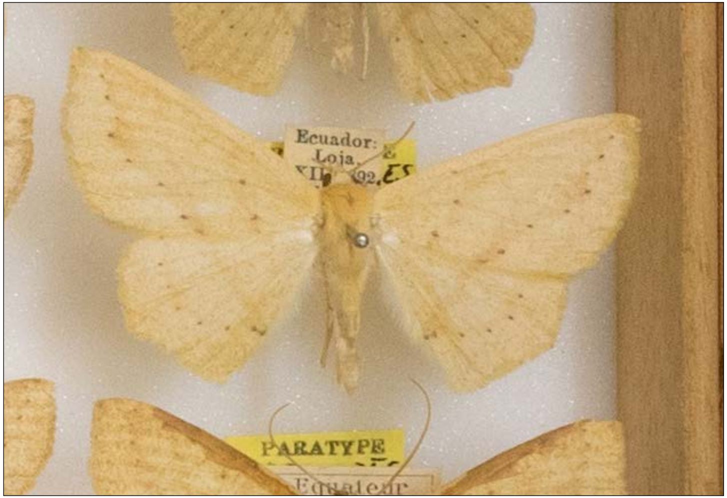

LMR-Geo-

0075

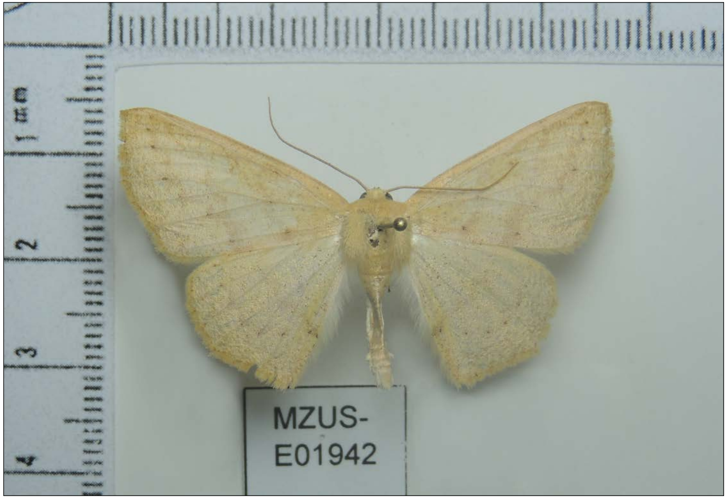

Sericoptera mahometaria group Herrich-Schäffer (TL: Venezuela)

BC

|BOLD:AEE1197

OTU-105

Additional compared specimen

very near: Ec-Geo-22020|Ecuador|Zamora Chinchipe|BOLD:ABY7947

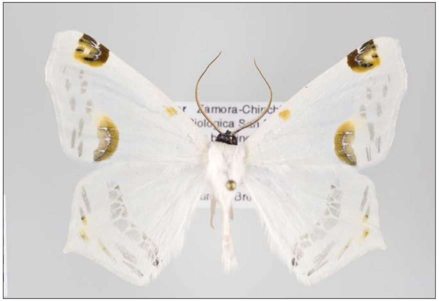

LMR-Geo-

0071

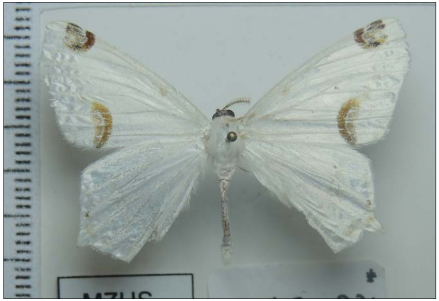

Compared specimen:

NHM type of synonym

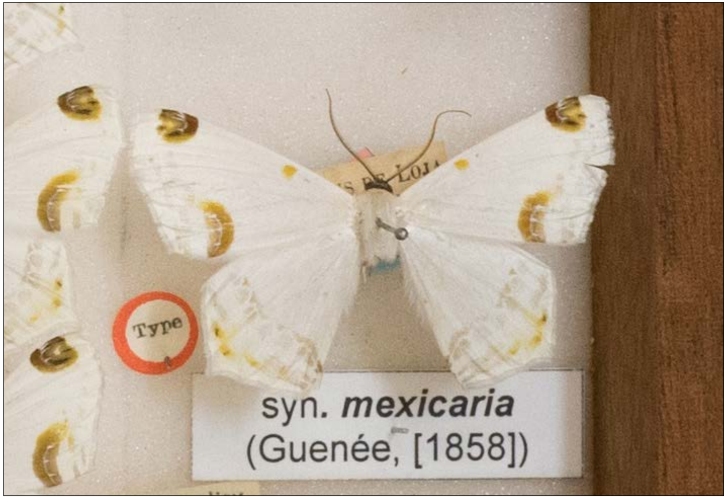

LMR-Geo-

0073

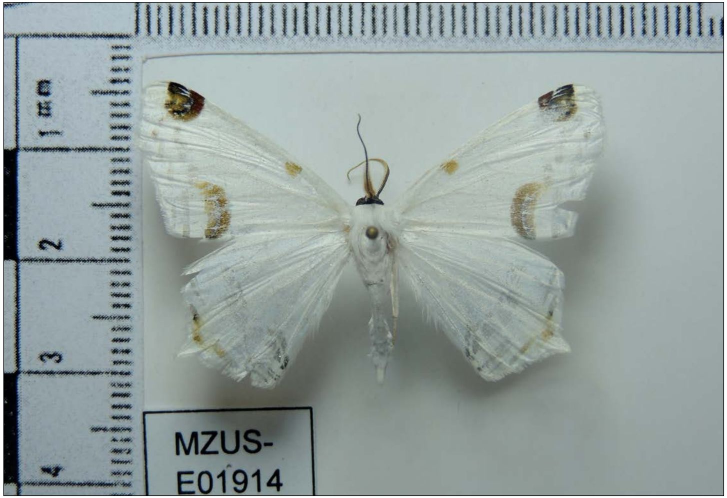

Sicya nr dognini Thierry-Mieg (TL: Bolivia)

BC

BOLD:AEE2297
OTU-216

Additional compared specimen
near: Pe-Geo-3781|Peru|Cuzco|BOLD:AEF7573

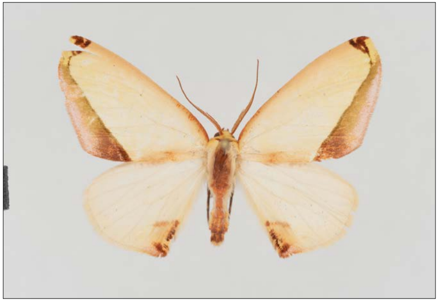

LMR-Geo-

Compared specimen:
USNM type

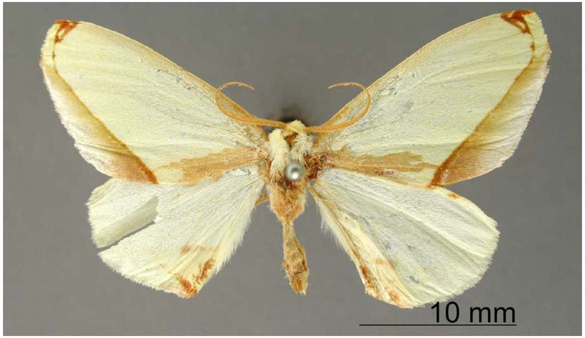

LMR-Geo-
0288

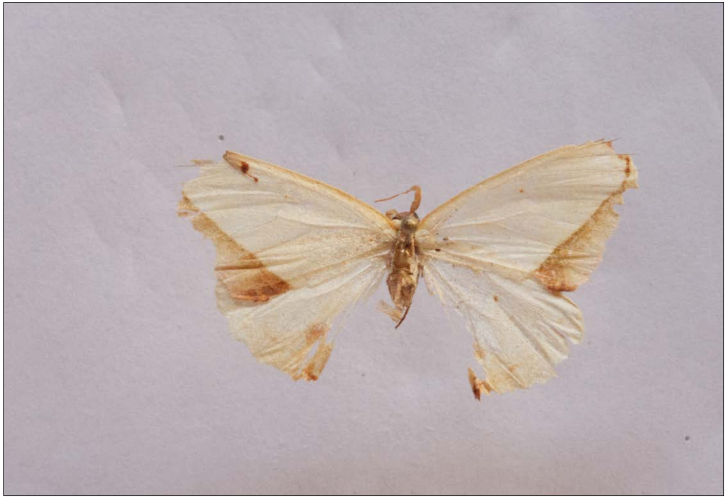

Sicyia nr pomona Oberthür (TL: [Peru]: Tambillo; Huambo)

BC

BOLD:AEB7289
OTU-55

Additional compared specimen
near: Ec-Geo-22190|Ecuador|Zamora Chinchipe|BOLD:AAH9580

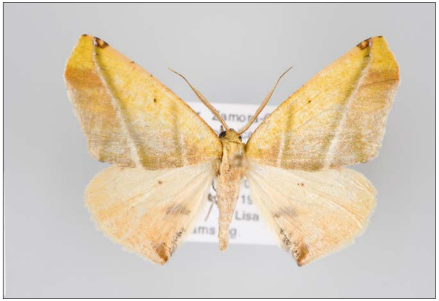

LMR-Geo-

Compared specimen:
NHM type

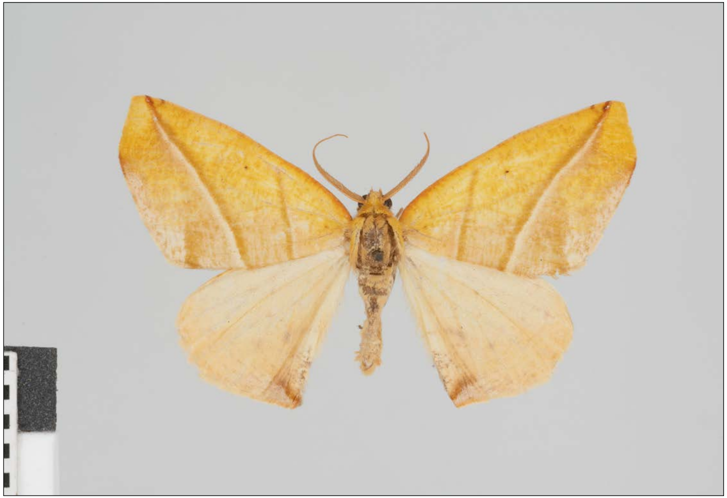

LMR-Geo-
0370

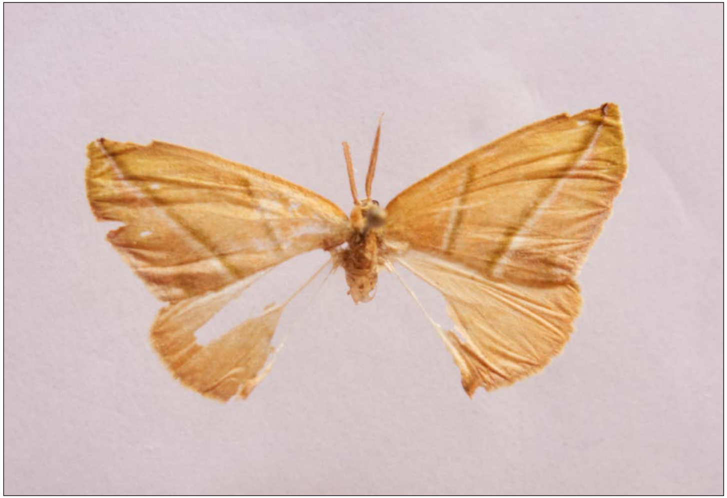

Siosta bifasciata group Latreille (TL: Equatorial America)

BC

BOLD:ACT1305

OTU-1

Additional compared specimen

LMR-Geo-

Compared specimen:

NHM type of synonym

LMR-Geo-

0302

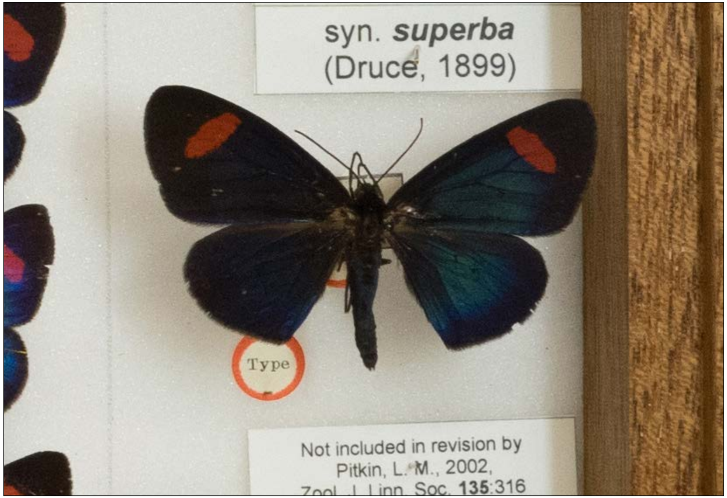

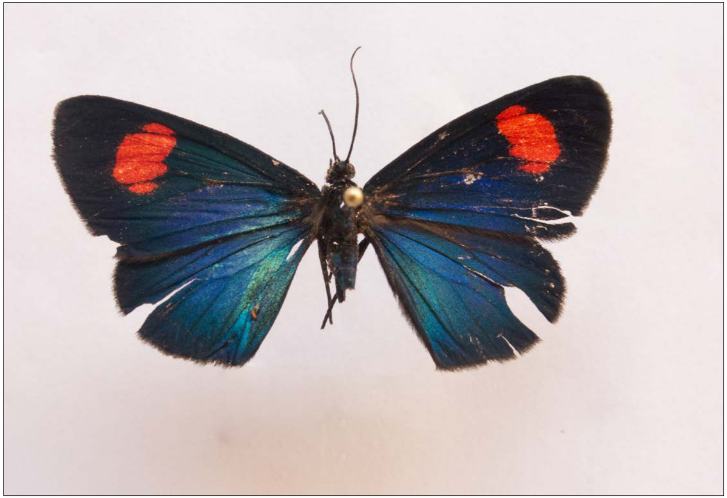

Thyrinteina arnobia Stoll (TL: Surinam)

BC

BOLD:AAA0688	
OTU-131	

Additional compared specimen

LMR-Geo-
0140

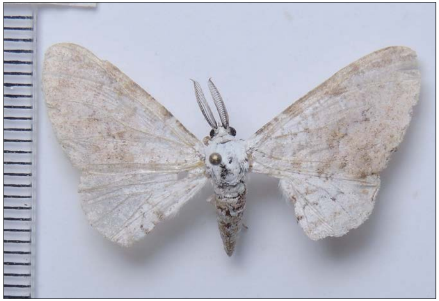

Compared specimen:
NHM no type

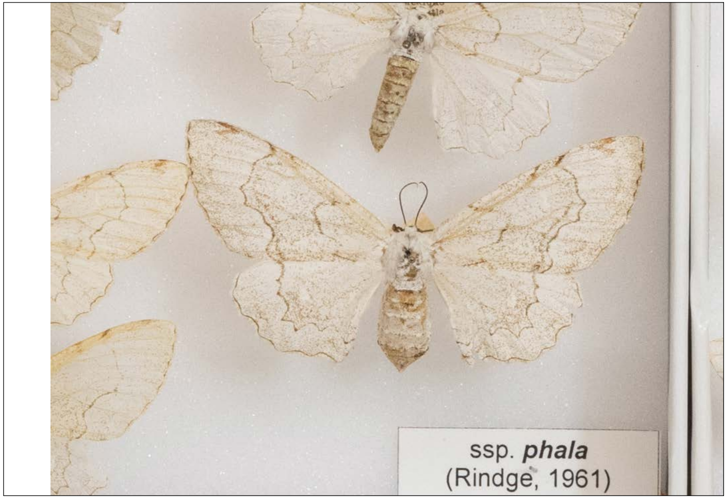

LMR-Geo-
0141

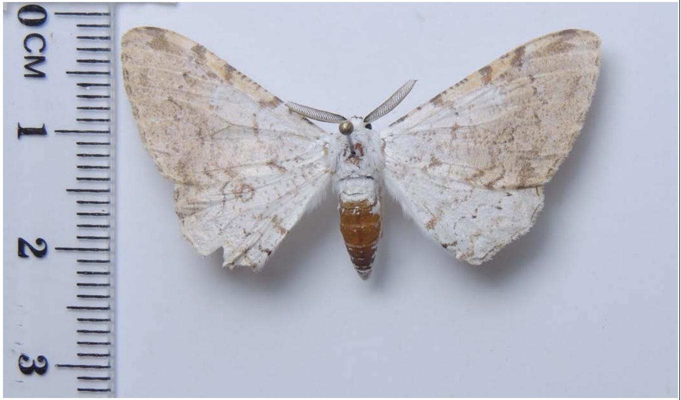

Undescribed genus

BC

BOLD:AAD4562
OTU-142

Additional compared specimen

LMR-Geo-

Compared specimen:

LMR-Geo-

0128

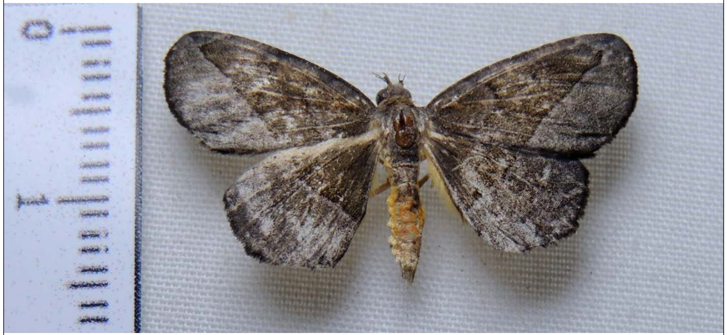

Undescribed genus

BC
BOLD:AEE1508
OTU-120

Additional compared specimen
near Pe-Geo-1204|Peru|Huanuco|BOLD:AAN5610

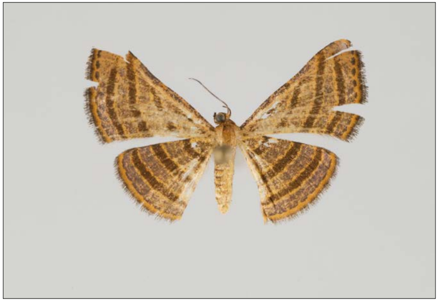

LMR-Geo-

Compared specimen:
NHM type

LMR-Geo-
0109

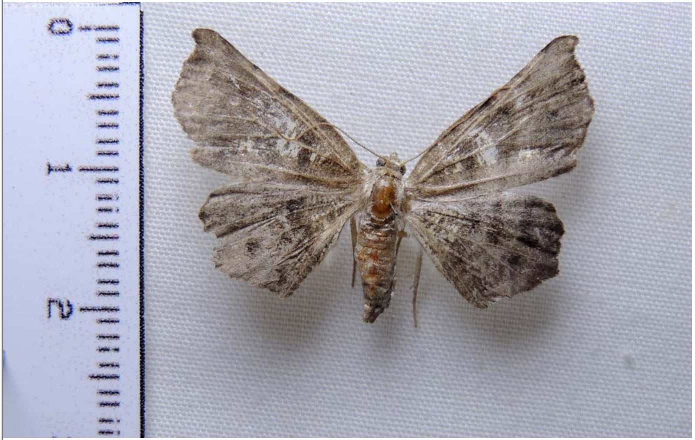

BC

BOLD:AAH0688
OTU-40

Additional compared specimen

LMR-Geo-

Compared specimen:

LMR-Geo-

0353

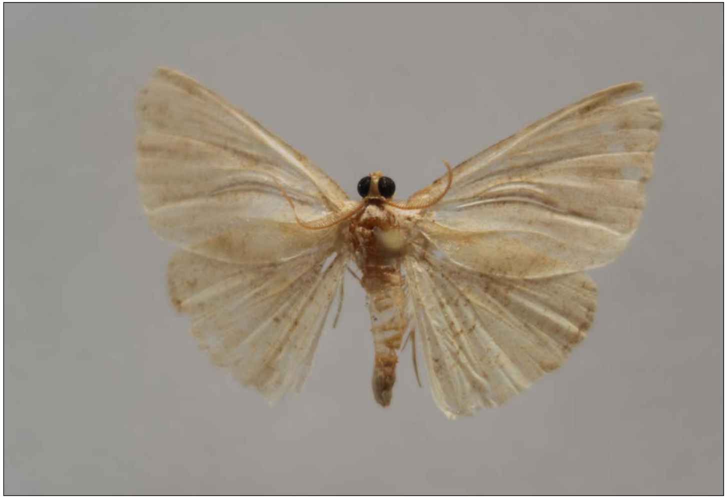

NN sp (TL:)

BC

BOLD:ADV3890
OTU-173

Additional compared specimen

LMR-Geo-

Compared specimen:

NHM type

LMR-Geo-

0205

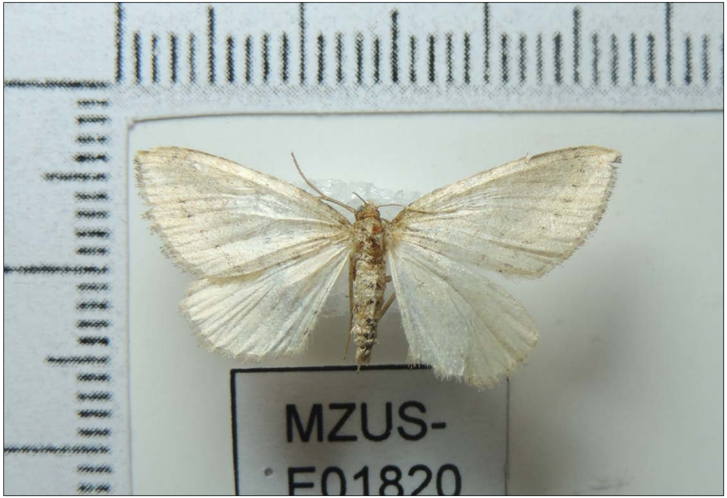

Undescribed genus (Boarmiini)

BC

BOLD:AEE9877
OTU-211

Additional compared specimen

LMR-Geo-
0132

Compared specimen:
NHM type

LMR-Geo-
0133

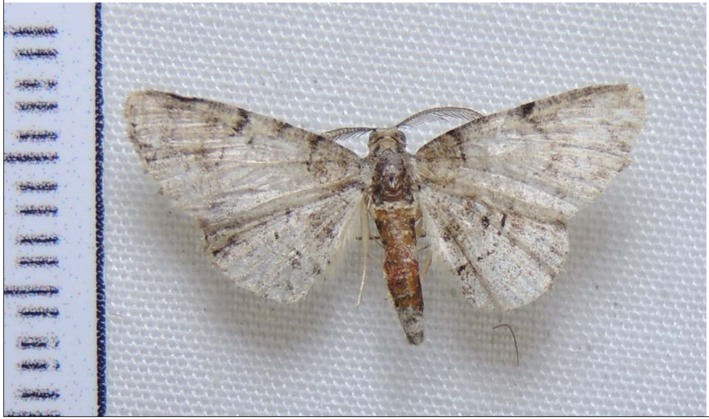

Supplement: Supplementary material 3 — Illustrated catalogue of all new records of Colombian Geometridae, part 1 [file bdj-09-e68693-s003.pdf]
