# Supplementary material for: A database and checklist of geometrid moths (Lepidoptera) from Colombia
Source: Biodivers Data J. 2021 Sep 3;9:e68693. doi: 10.3897/BDJ.9.e68693 (PMC8433126; doi:10.3897/BDJ.9.e68693)

Colombia Geometridae Other subfamilies than Ennominae identification catalogue

State 1 April 2021

Identified using the NHM collection and USNM images

alphabetical order

Geometrinae

Chloropteryx nr nordicaria Schaus (TL: Mexico, [Veracruz], Orizaba)

BC

no BIN 420 bp
OTU-130

Additional compared specimen
near: Pe-Geo-1097|Peru|Huanuco|BOLD:ACZ0516

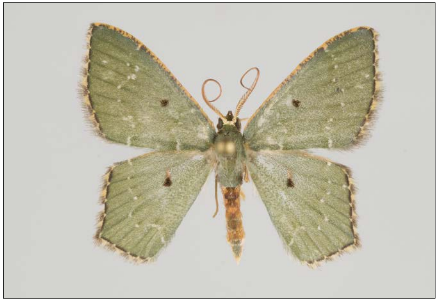

LMR-Geo-
0150

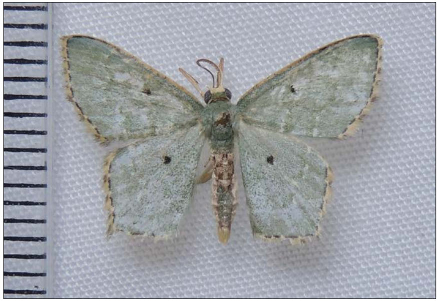

Compared specimen:
USNM type

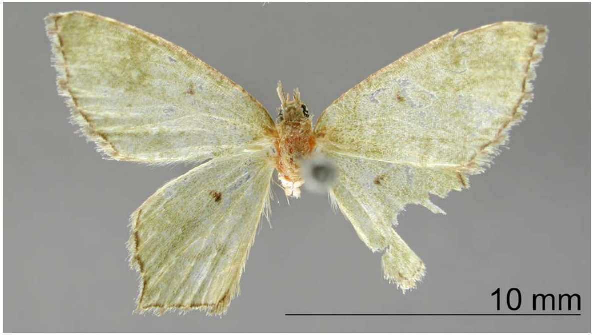

LMR-Geo-
0146

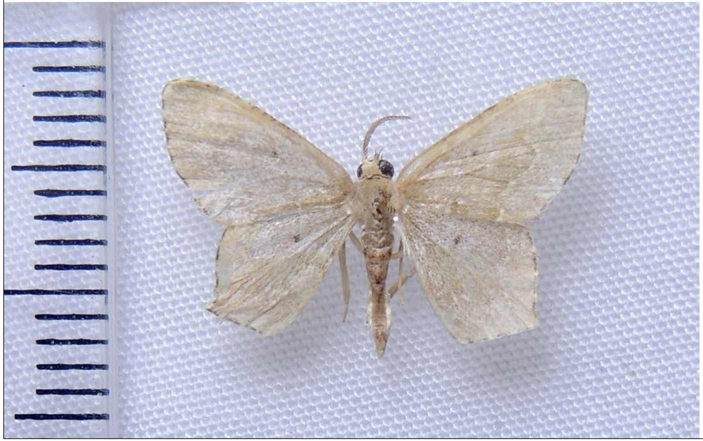

Chloropteryx nr opalaria Guenée (TL: Brazil)

BC

BOLD:AAT9814
OTU-26

Additional compared specimen
= Pe-Geo-0758|Peru|Cuzco

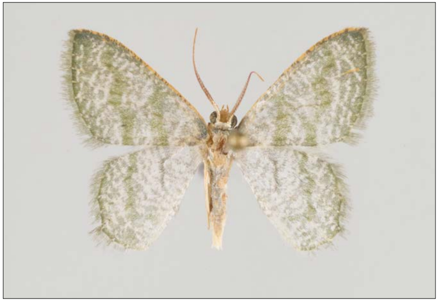

LMR-Geo-

Compared specimen:
USNM type

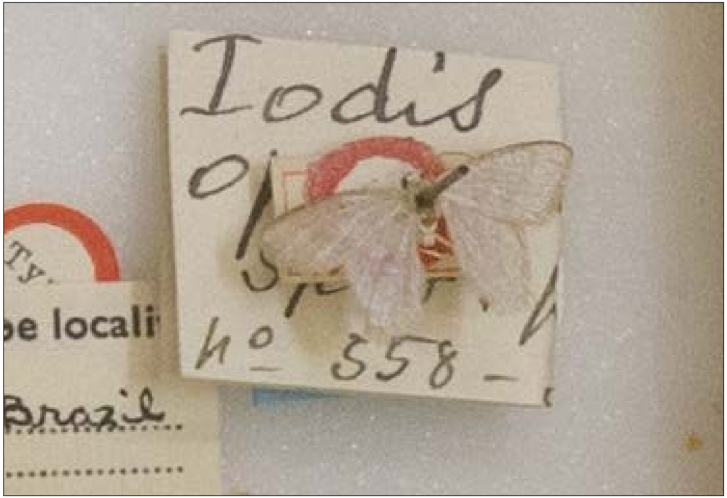

LMR-Geo-
0334

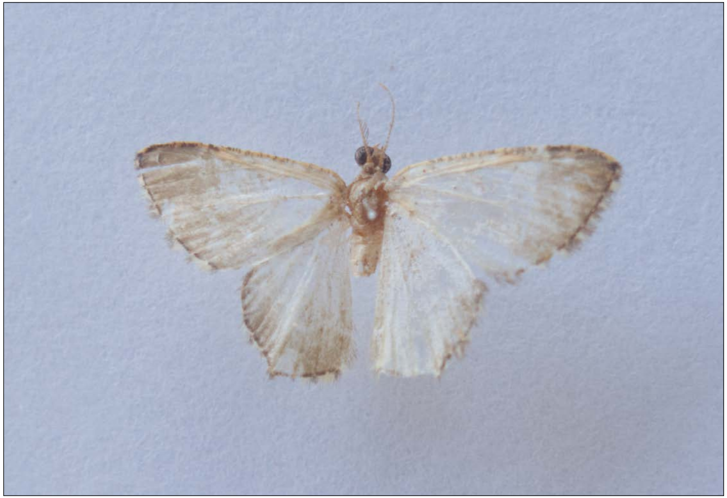

Chloropteryx punctilinea Dognin (TL: French Guiana: St Laurent du Maroni)

BC

BOLD:AAD8208
OTU-27

Additional compared specimen
= GeoCR 25211|Costa Rica|Heredia|BOLD:AAD8208

LMR-Geo-

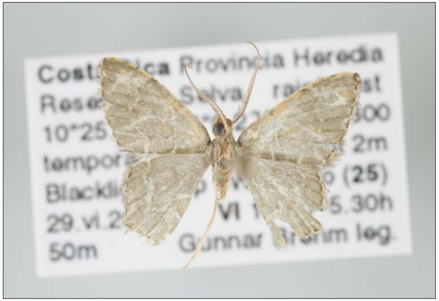

Compared specimen:
USNM type

LMR-Geo-
0333

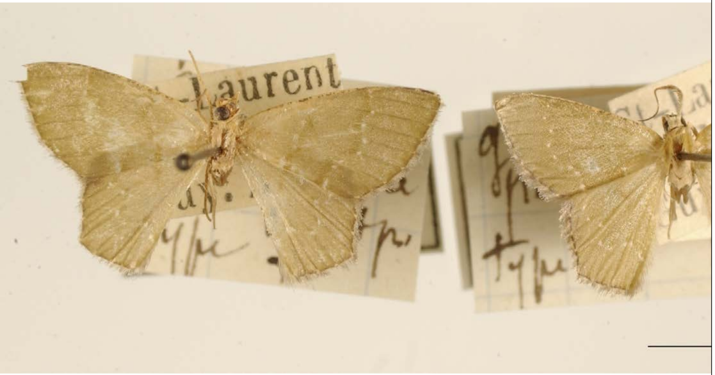

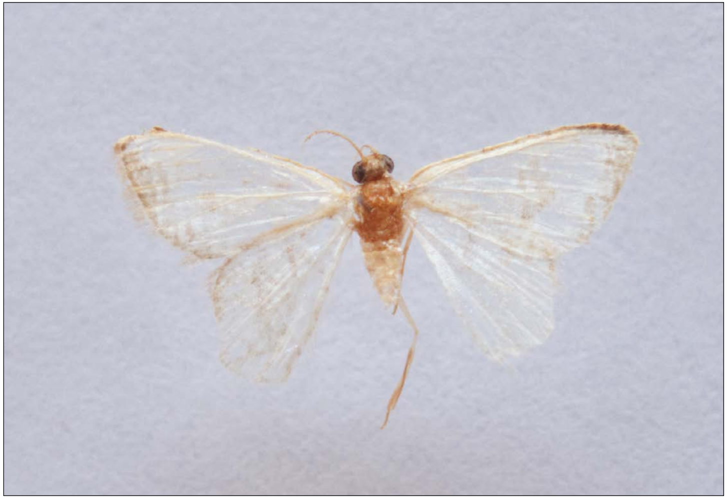

Chloropteryx spumosaria Dognin (TL: Ecuador: Loja)

BC

BOLD:AAM5958
OTU-203

Additional compared specimen
= Ec-Geo-22396|Ecuador|Loja|

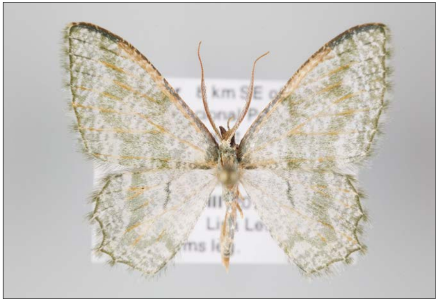

LMR-Geo-

Compared specimen:

USNM type

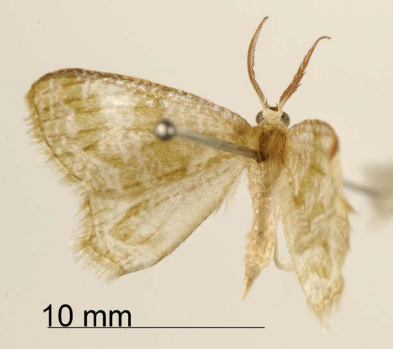

LMR-Geo-

0249

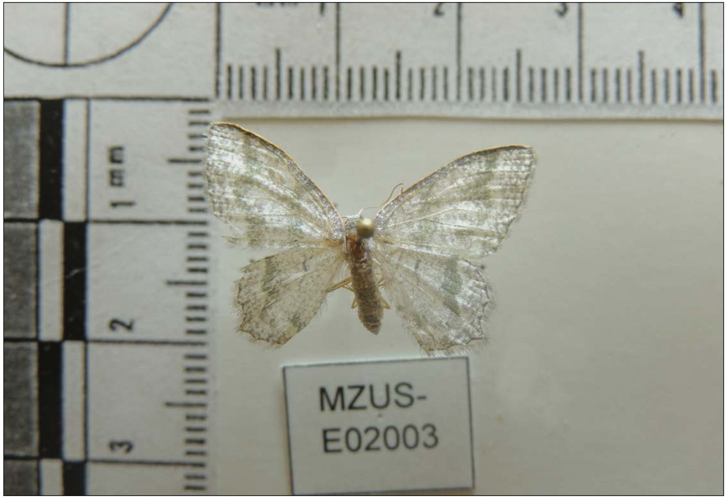

Chloropteryx sp (TL:)

BC

no BIN / 340 bp	
OTU-133	

Additional compared specimen

LMR-Geo-

Compared specimen:

LMR-Geo-
0147

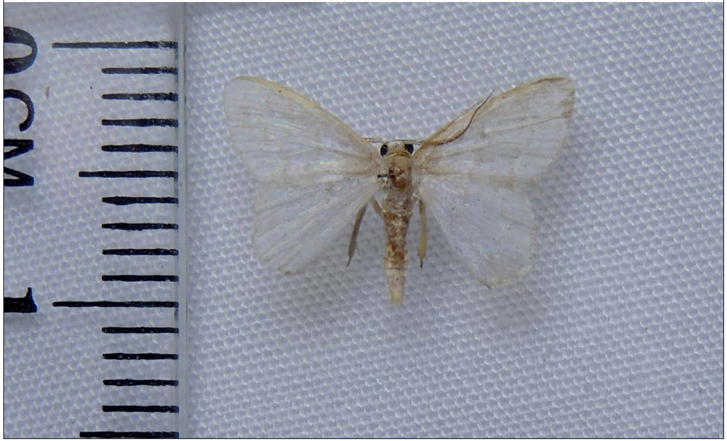

Chloropteryx sp (TL:)

BC

BOLD:AAD0693
OTU-33

Additional compared specimen
near: Ec-Geo-22695|Ecuador|Zamora Chinchipe|BOLD:ACF6052

LMR-Geo-

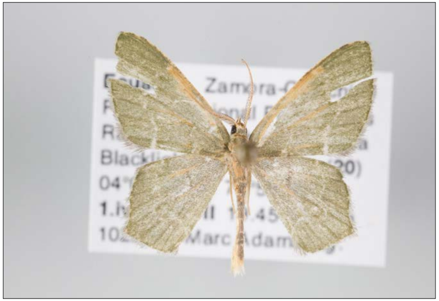

Compared specimen:
xx

LMR-Geo-
0344

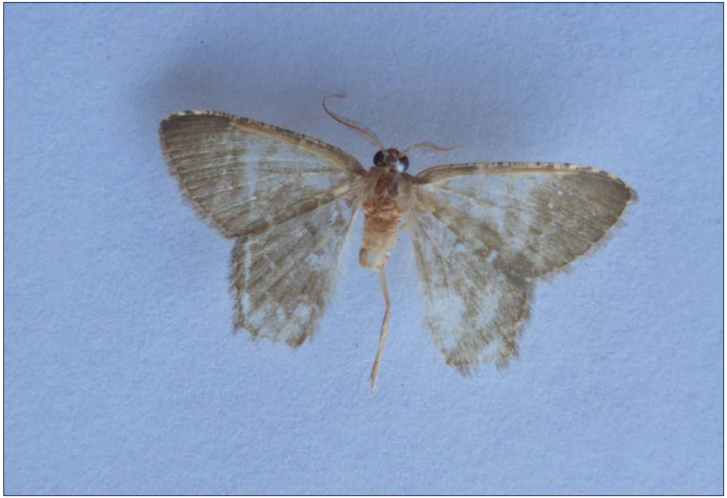

Lissochlora nigripes Dognin (TL: Colombia, Paramo del Quindin, 3800 m)

BC

BOLD:AEE3986

OTU-187

Additional compared specimen
distant: Ec-Geo-25121|Ecuador|Loja|BOLD:AAP2901

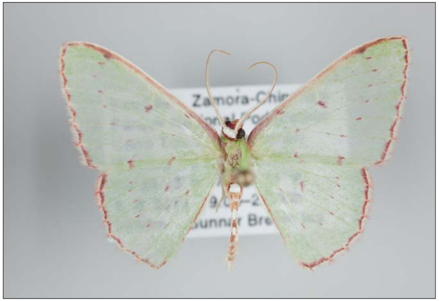

LMR-Geo-

Compared specimen:

USNM type

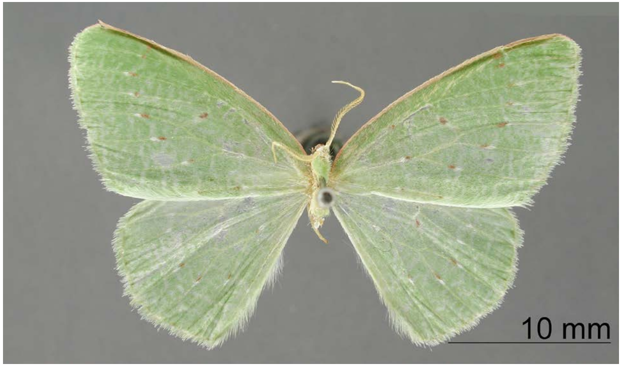

LMR-Geo-

0216

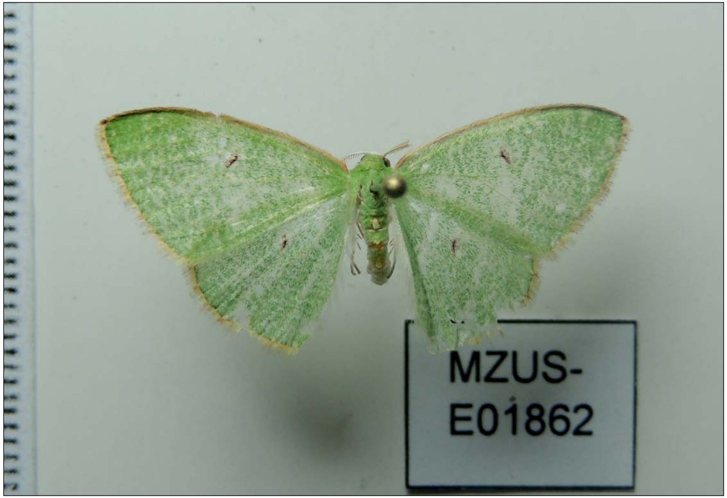

Lissochlora nr pasama Dognin (TL: Ecuador: near Loja, El Monje)

BC

BOLD:AEC0922

OTU-12

Additional compared specimen
distant: *pasama* Ec-Geo-18191|Ecuador|Zamora Chinchipe|BOLD:AAK8807

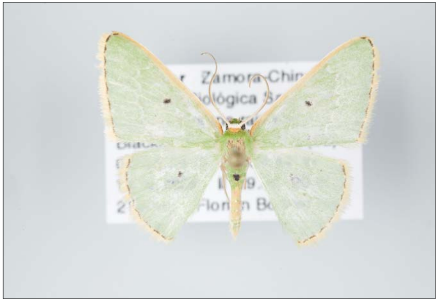

LMR-Geo-

Compared specimen:

USNM type

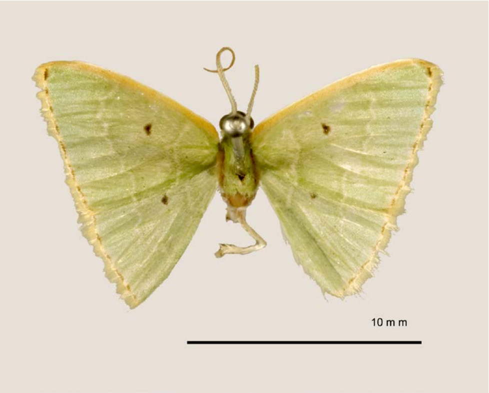

LMR-Geo-

0316

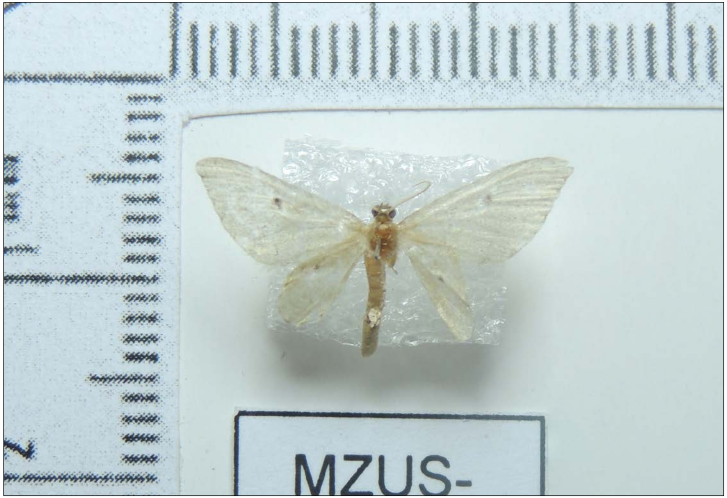

Lissochlora nr pectinifera Prout (TL: Peru: Carabaya, Santo Domingo, 6500 ft)

BC

BOLD:AEE6635

OTU-191

Additional compared specimen
distant: Ec-Geo-22033|Ecuador|Zamora Chinchipe|BOLD:AAI1120

LMR-Geo-

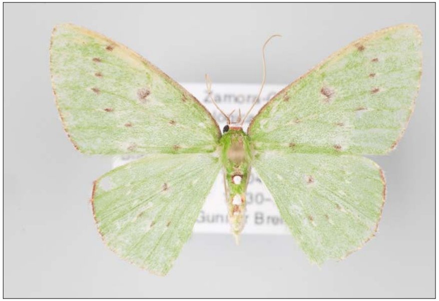

Compared specimen:

NHM type

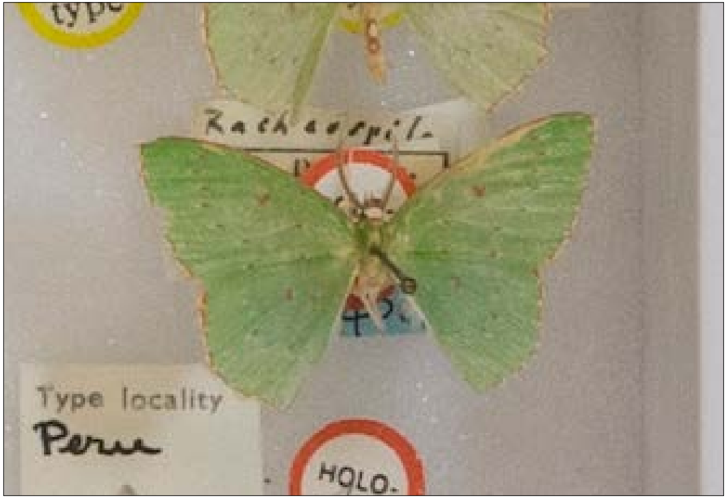

LMR-Geo-

0221

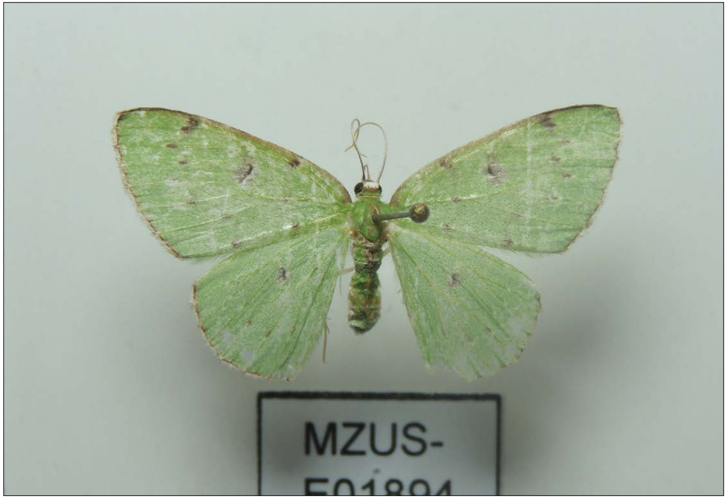

Lissochlora sp (TL:)

BC

BOLD:AEE3024
OTU-76

Additional compared specimen
near: Ec-Geo-17718|Ecuador|Zamora Chinchipe|BOLD:AAI1118

LMR-Geo-

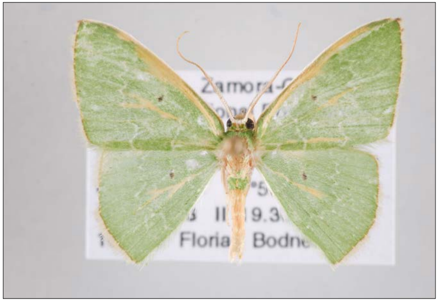

Compared specimen:

LMR-Geo-
0018

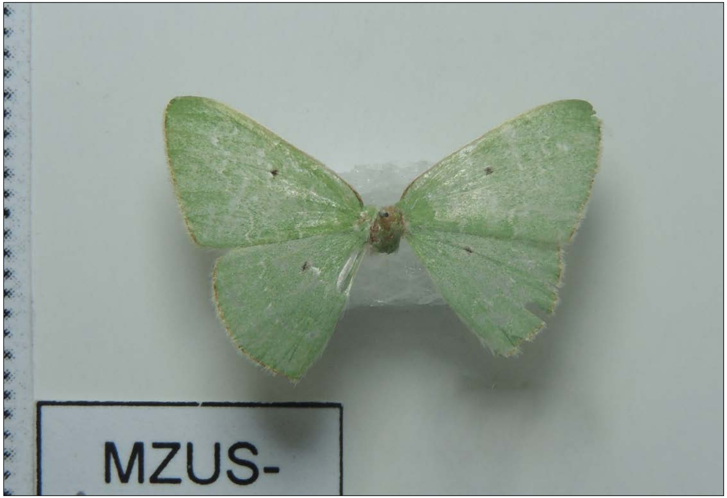

Neagathia corruptata group Felder & Rogenhofer (TL: Amazon)

BC

BOLD:AAF7692

OTU-135

Additional compared specimen
distant: Pe-Geo-0485|Peru|Cuzco|658[0n]|BOLD:AAI3231

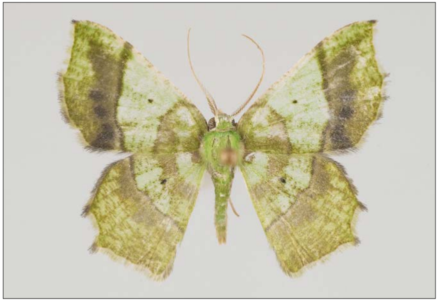

LMR-Geo-

Compared specimen:
USNM type

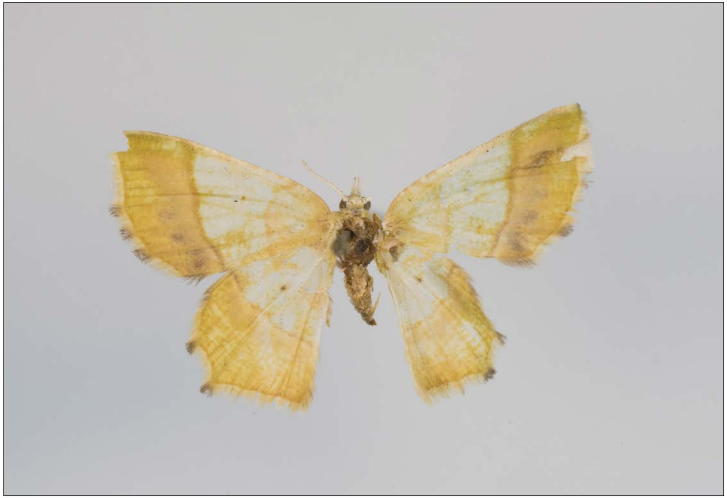

LMR-Geo-
0152

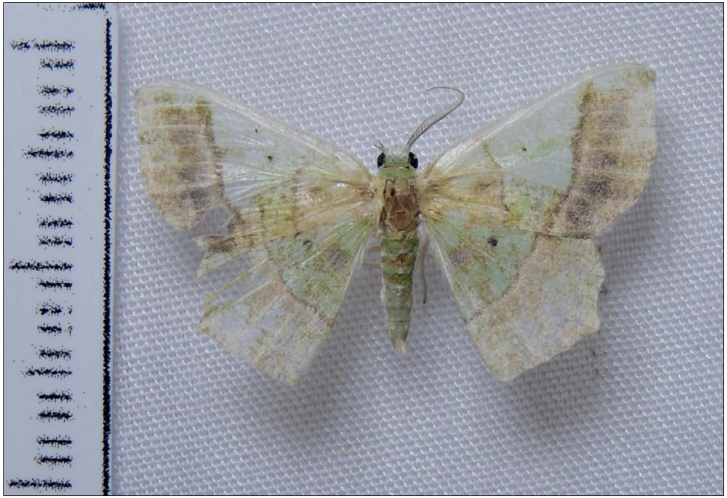

Nemoria punctilinea Dognin (TL: Venezuela, Mérida)

BC

BOLD:AAW0122

OTU-42

Additional compared specimen

LMR-Geo-

Compared specimen:

USNM type

LMR-Geo-

0351

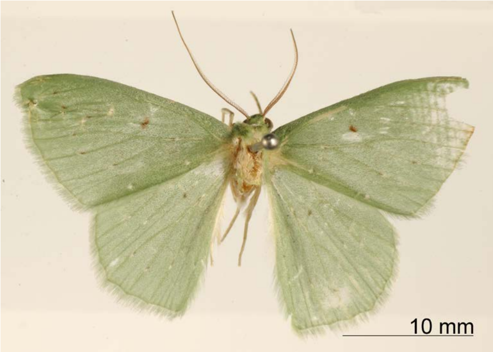

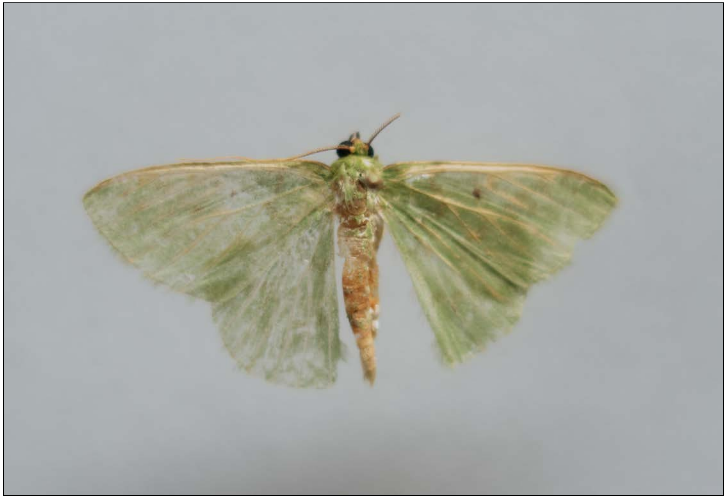

Nemoria vermiculata Dognin (TL: Colombia, Pacho, 2200 m)

BC

BOLD:AEE3299

OTU-198

Additional compared specimen

LMR-Geo-

Compared specimen:

USNM type

LMR-Geo-

0258

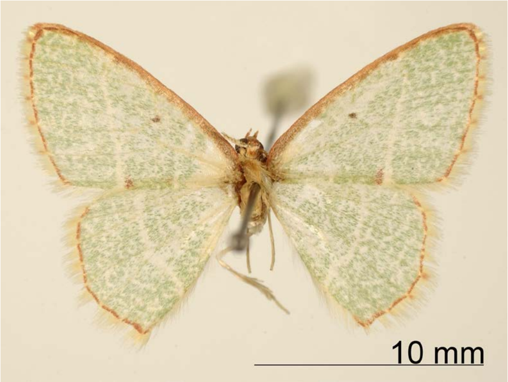

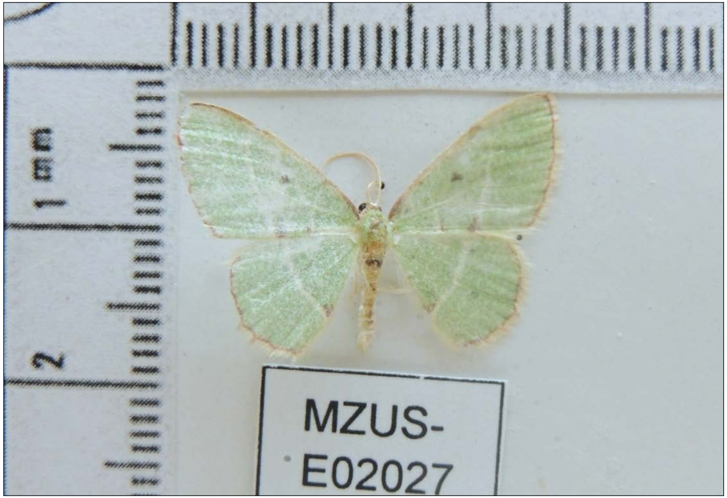

Nemoria sp (TL:)

BC

BOLD:AEE3298

OTU-190

Additional compared specimen

LMR-Geo-

Compared specimen:

x

LMR-Geo-

0220

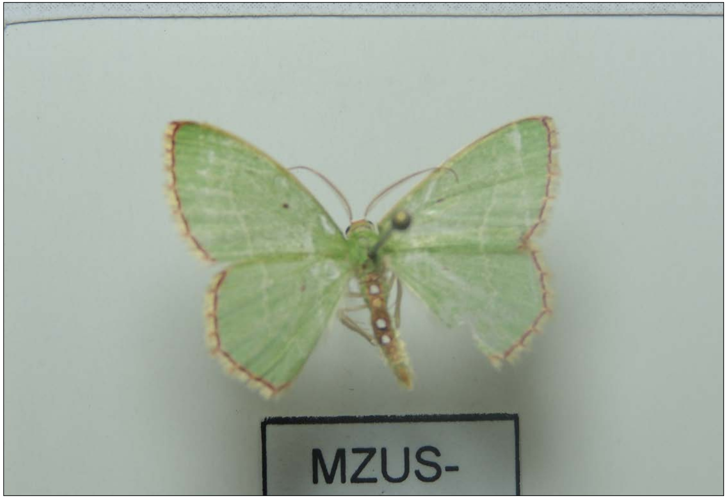

Nemoria sp (TL:)

BC

BOLD:AEB9830
OTU-15

Additional compared specimen

LMR-Geo-
0223, 0273

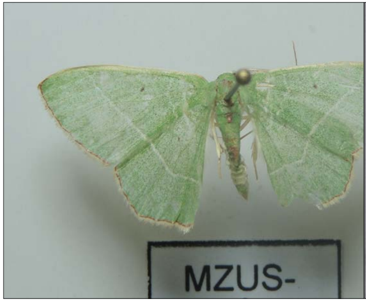

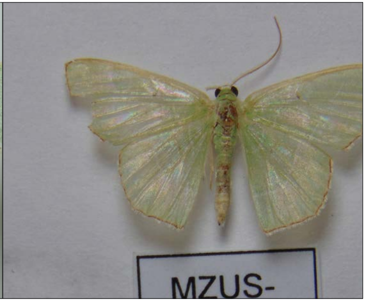

Compared specimen:
x

LMR-Geo-
0322

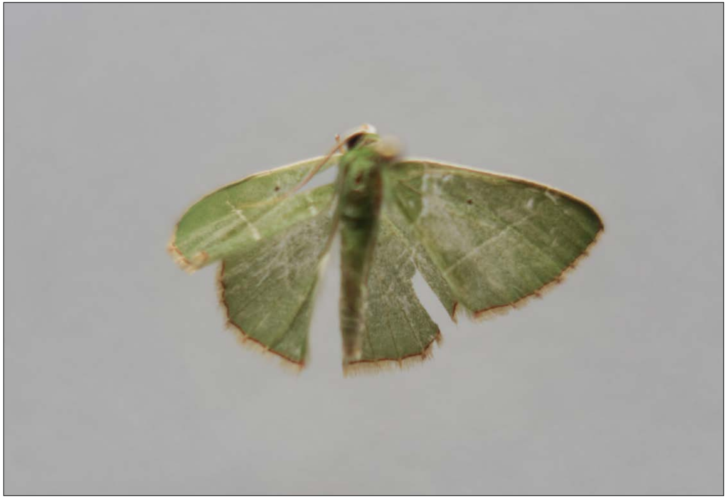

Oospila nr venezuelata Walker (TL: Venezuela)

BC
BOLD:AAK8812
OTU-152

Additional compared specimen

LMR-Geo-

Compared specimen:

USNM type

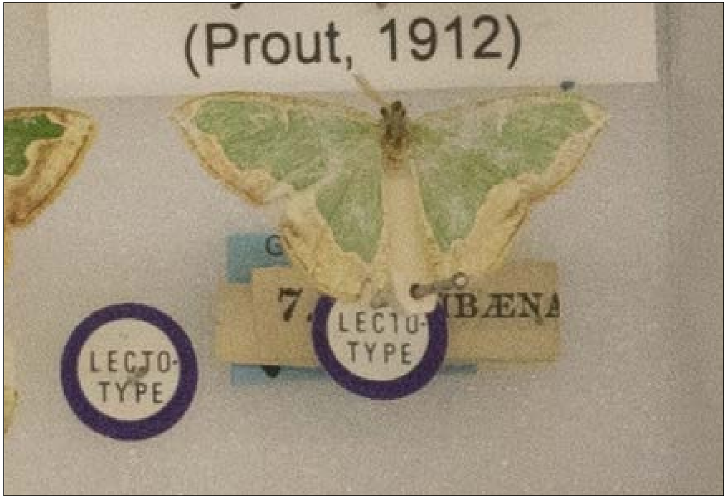

LMR-Geo-

0156

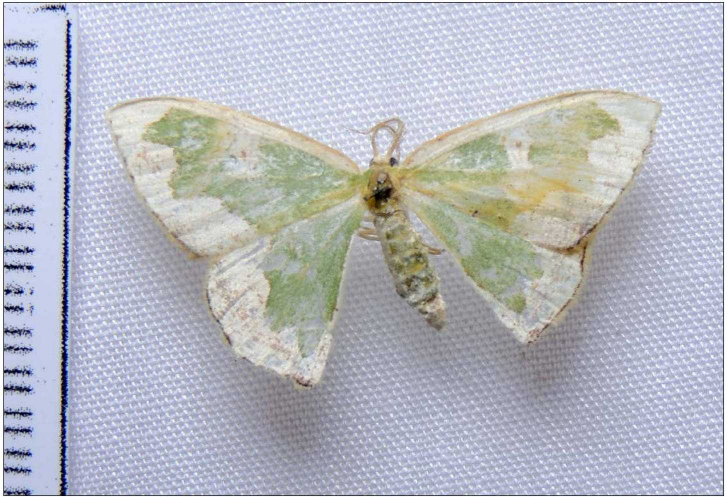

Phrudocentra pupillata Warren (TL: British Guiana [Guyana]: Rio Demerara)

BC

BOLD:AAB0438
OTU-24

Additional compared specimen

LMR-Geo-

Compared specimen:

NHM type

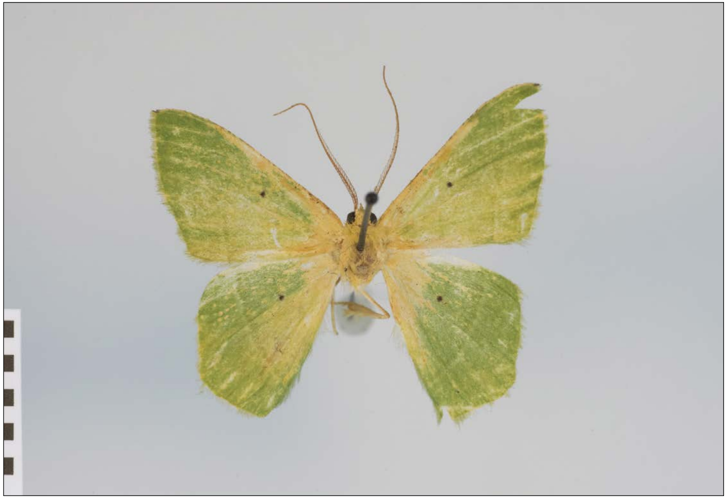

LMR-Geo-

0332

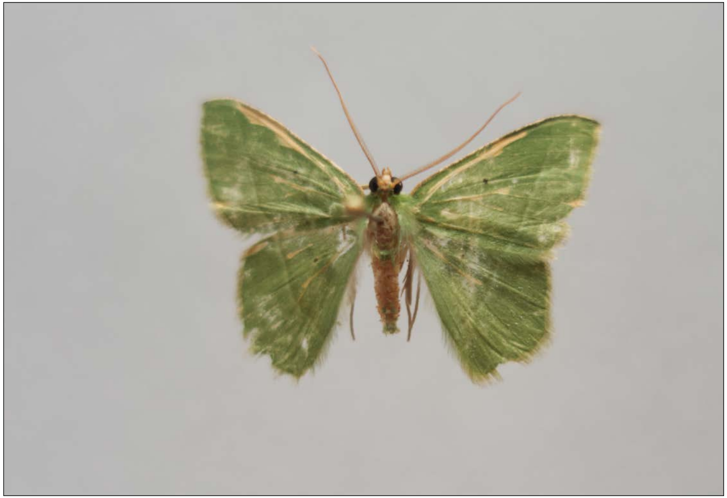

Synchlora frondaria group Guenée (TL: [French Guiana]: Cayenne)

BC

BOLD:AEE7742
OTU-132

Additional compared specimen
distant: Pe-Geo-1166|Peru|Huanuco|BOLD:AAF4445

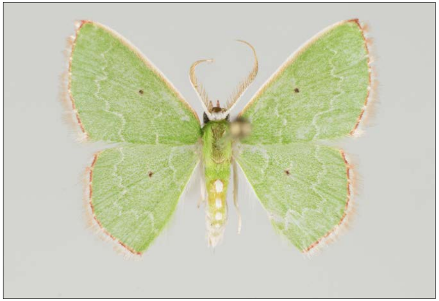

LMR-Geo-
0160

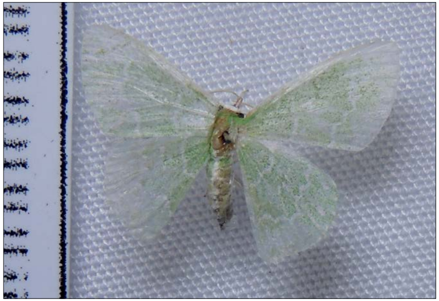

Compared specimen:
NHM type

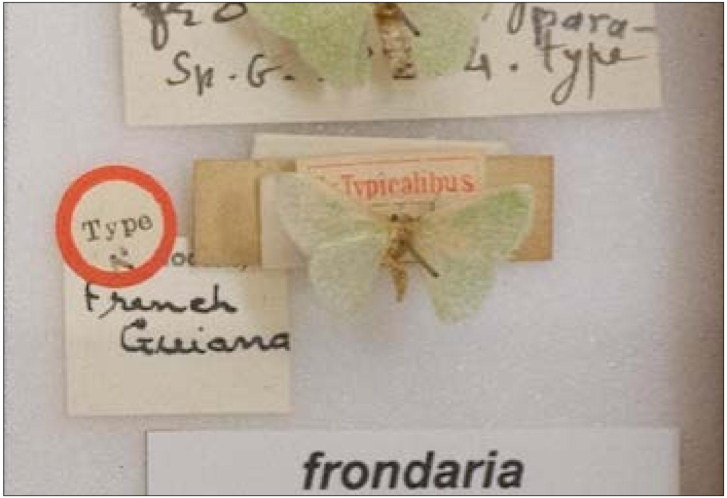

LMR-Geo-
0151

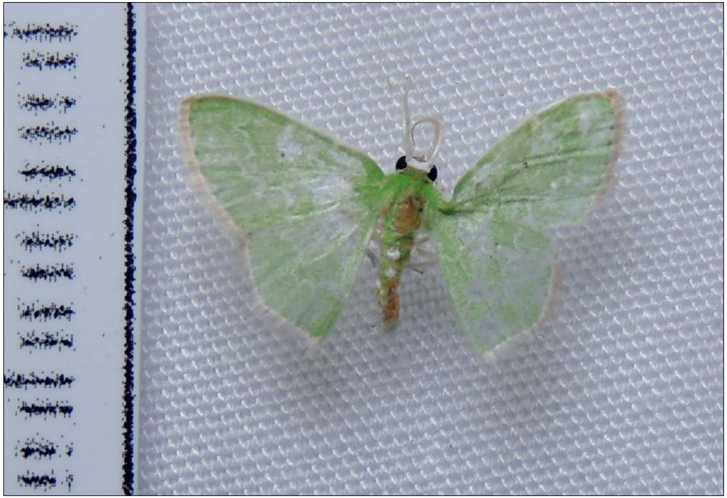

Synchlora gerularia Hübner (TL: ?)

BC

BOLD:AAA0783
OTU-153

Additional compared specimen
= Pe-Geo-0725|Peru|Huanuco

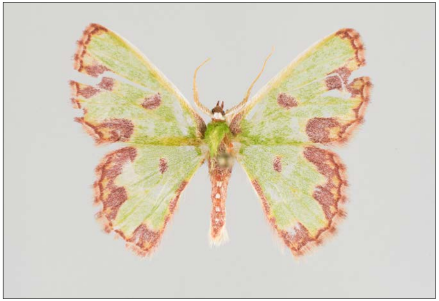

LMR-Geo-

Compared specimen:
NHM type of synonym

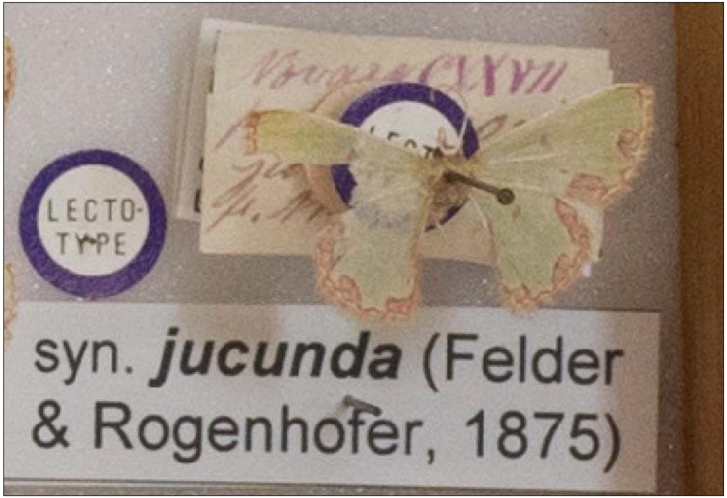

LMR-Geo-
0161

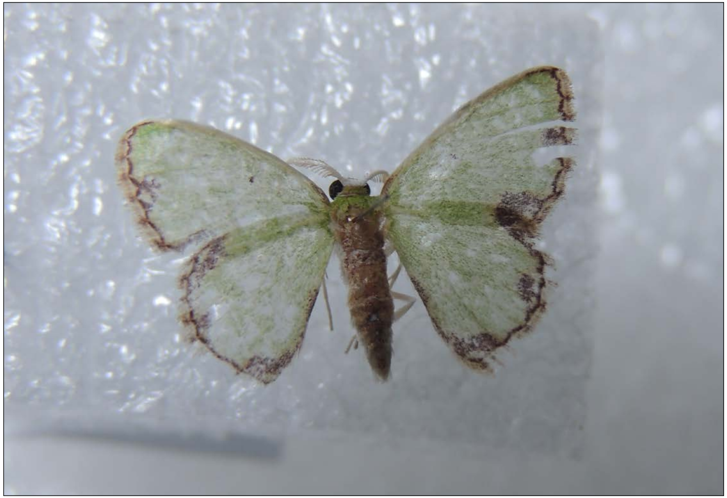

Synchlora leucoceraria Snellen (TL: [Colombia]: Bogota)

BC

BOLD:AEE8225
OTU-160

Additional compared specimen
distant: Ec-Geo-19183|Ecuador|Zamora Chinchipe|BOLD:ACF8770

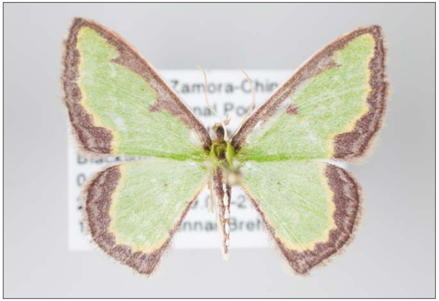

LMR-Geo-

Compared specimen:
NHM no type

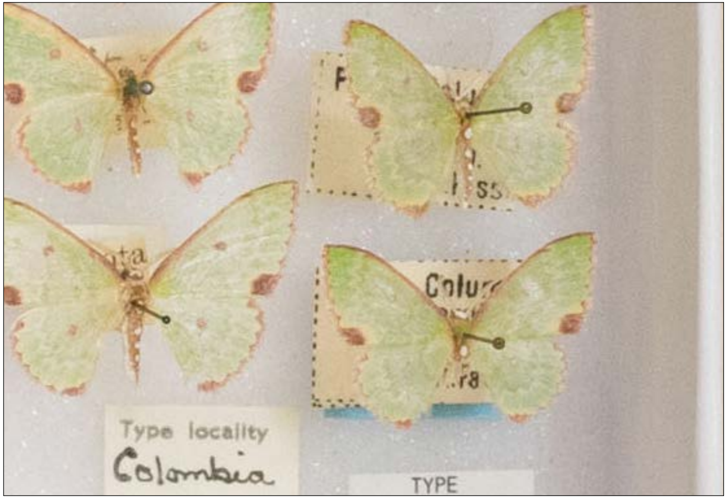

LMR-Geo-
0210

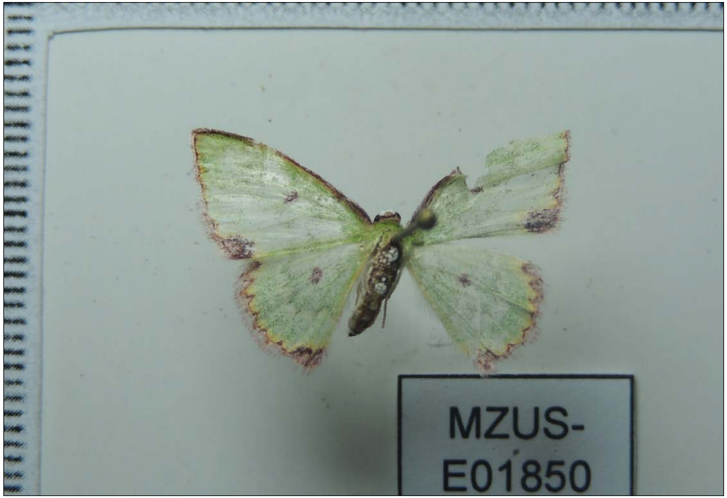

Synchlora pulchrifimbria Warren (TL: Surinam, Maroewyn Valley, Aroewarwa Creek)

BC

BOLD:AAE2416
OTU-46

Additional compared specimen
= Pe-Geo-0827|Peru|Huanuco

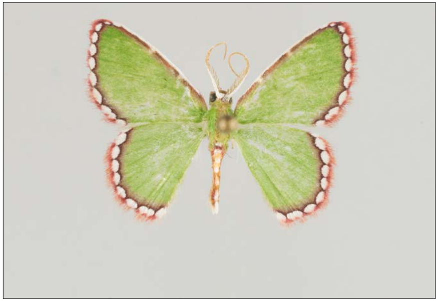

LMR-Geo-

Compared specimen:

NHM type

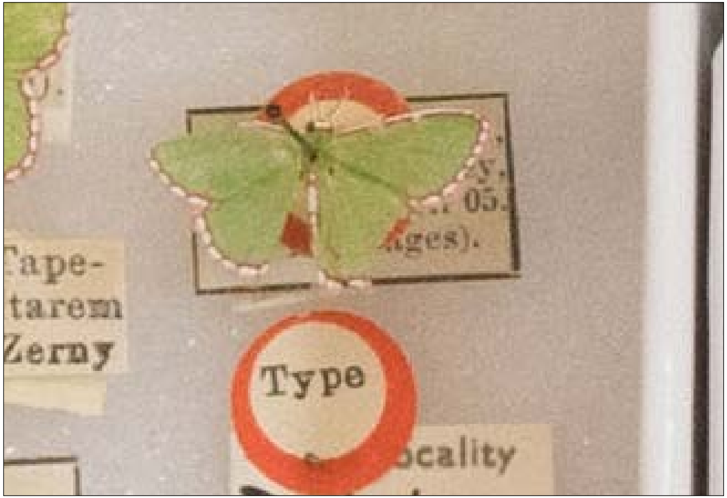

LMR-Geo-

0355

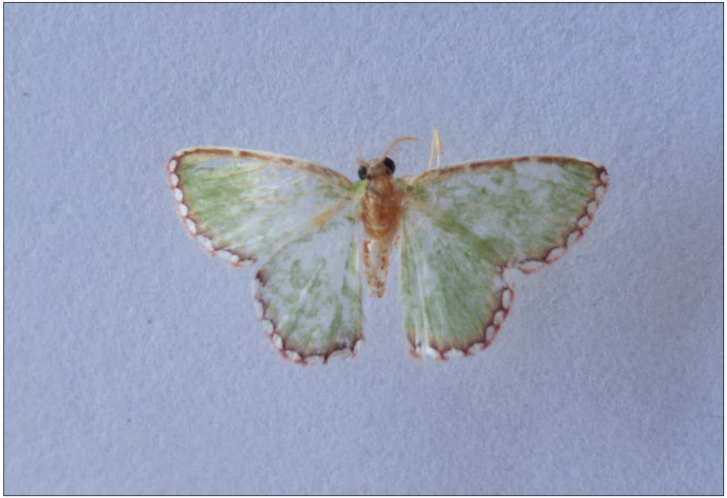

Tachyphyle sp (TL:)

BC

BOLD:AEF0021
OTU-154

Additional compared specimen
near: Pe-Geo-0874|Peru|Huanuco|BOLD:ACJ6938

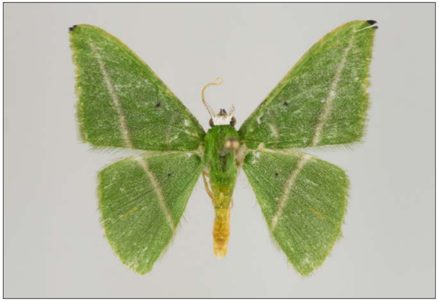

LMR-Geo-

Compared specimen:

LMR-Geo-
0163

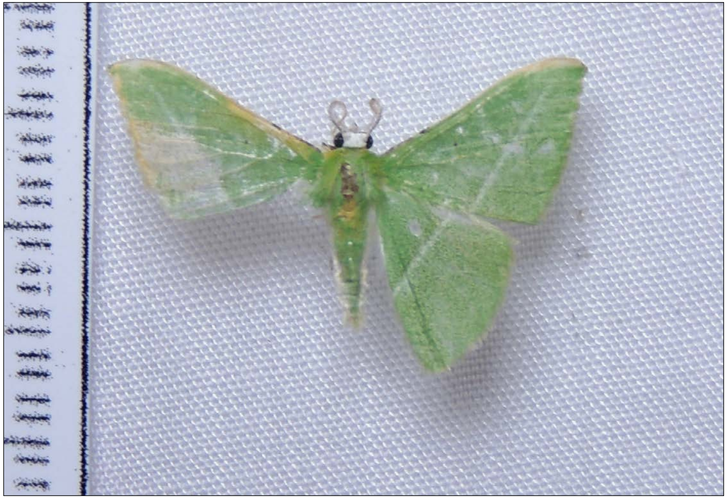

Larentiinae

Cirrolygris cecilia Dognin (TL: Colombia: Monte Socorro, 3500 and 3800 m)

BC

BOLD:AEE1101

OTU-083

Additional compared specimen

distant Ec-Geo-19312|Ecuador|Loja|BOLD:ACF7897

LMR-Geo-

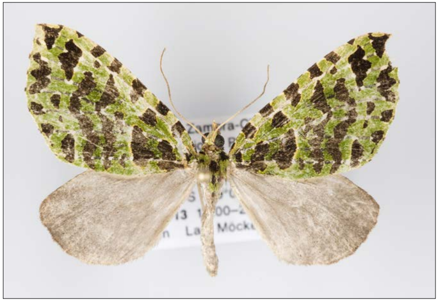

Compared specimen:

LMR-Geo-

0038

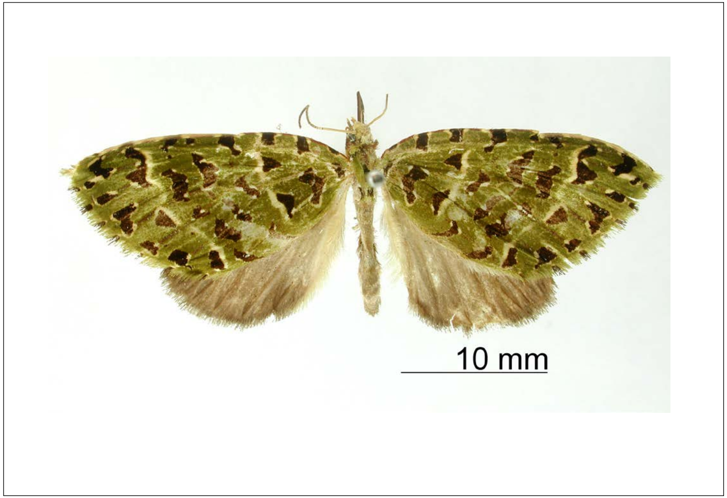

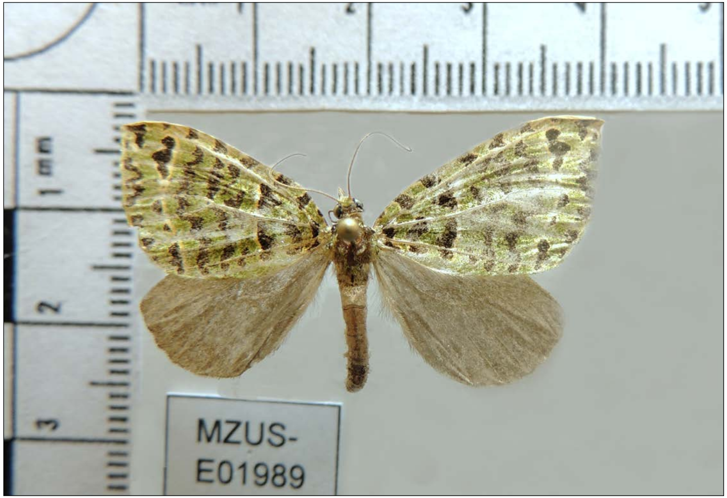

Eois adimaria group Snellen (TL: [Colombia]: Barro Blanco)

BC

BOLD:AAM5917
OTU-099

Additional compared specimen
near Pe-Geo-3388|Peru|Cuzco|BOLD:AAM5917

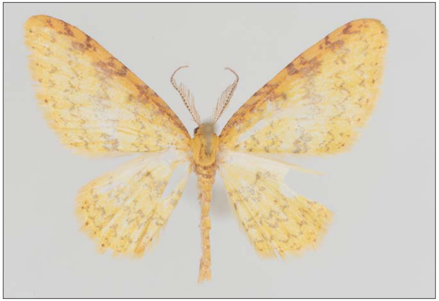

LMR-Geo-

Compared specimen:
RMNH type

LMR-Geo-
0058

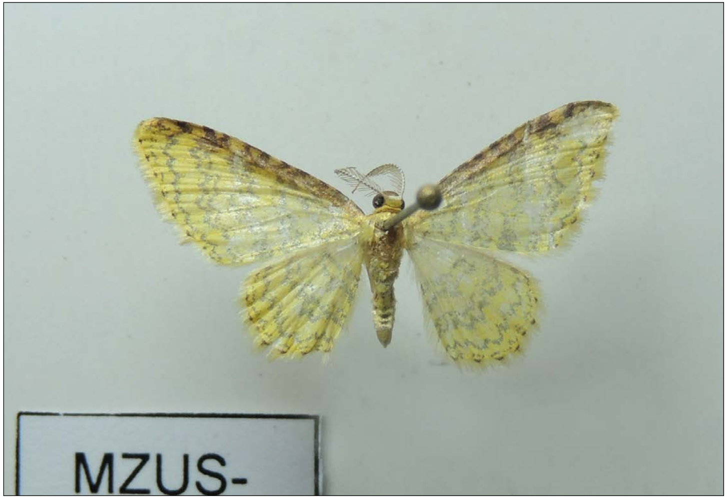

Eois adimaria group Snellen (TL: [Colombia]: Barro Blanco)

BC

BOLD:AEE7826
OTU-097

Additional compared specimen
near Pe-Geo-0204|Peru|Cuzco|BOLD:ABW9724

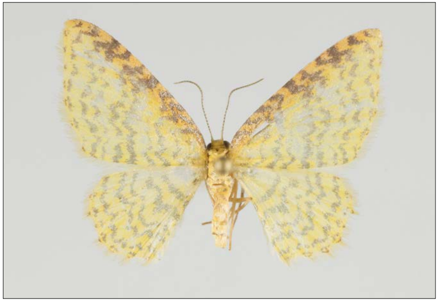

LMR-Geo-

Compared specimen:

RMNH type

LMR-Geo-

0061

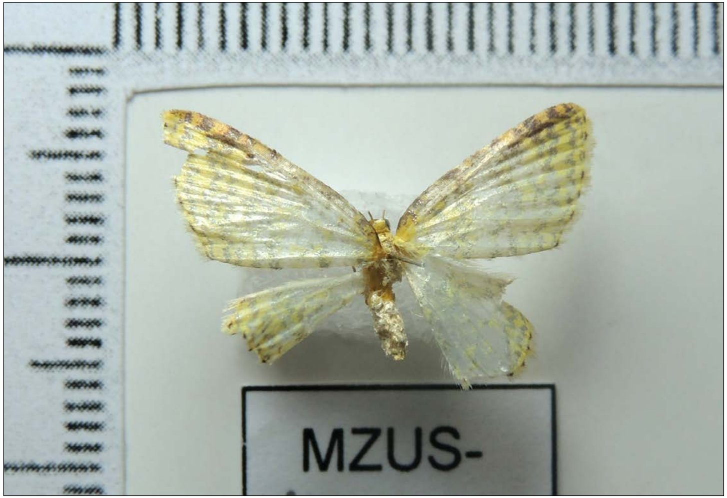

Eois nr ambarilla Dognin (TL: [Ecuador]: Loja environments)

BC
BOLD:ABA7110
OTU-172

Additional compared specimen
= Pe-Geo-0227|Peru|Cuzco

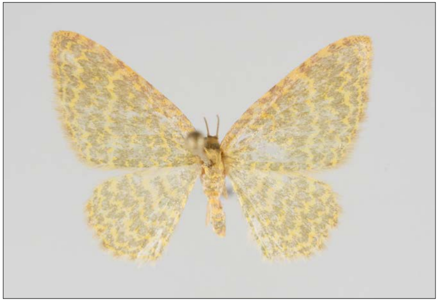

LMR-Geo-

Compared specimen:
USNM type

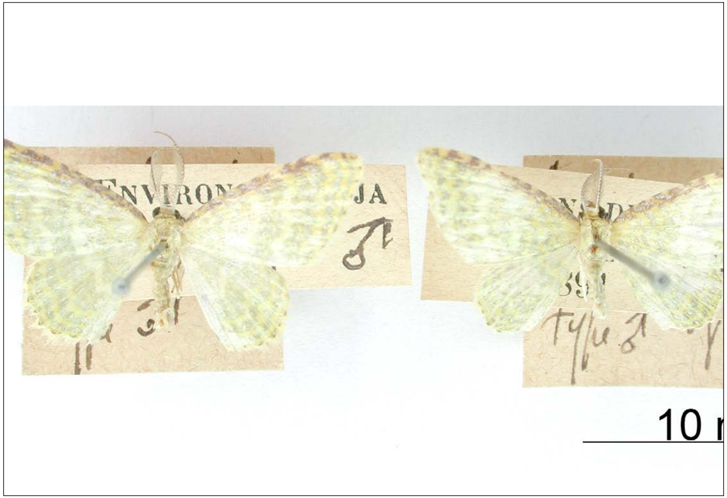

LMR-Geo-
0206

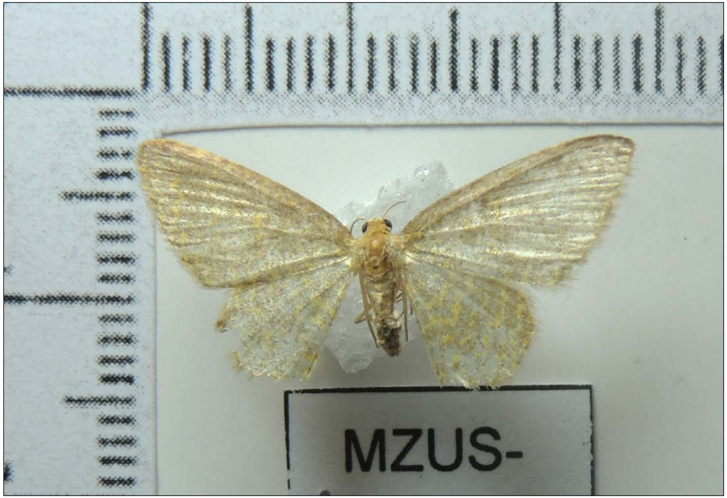

Eois azafranata group Dognin (TL: [Ecuador]: Loja)

BC

BOLD:AEE1124
OTU-184

Additional compared specimen
distant: Ec-Geo-18865|Ecuador|Loja|BOLD:AAW5495

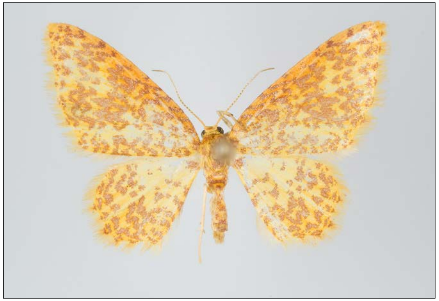

LMR-Geo-

Compared specimen:

USNM type

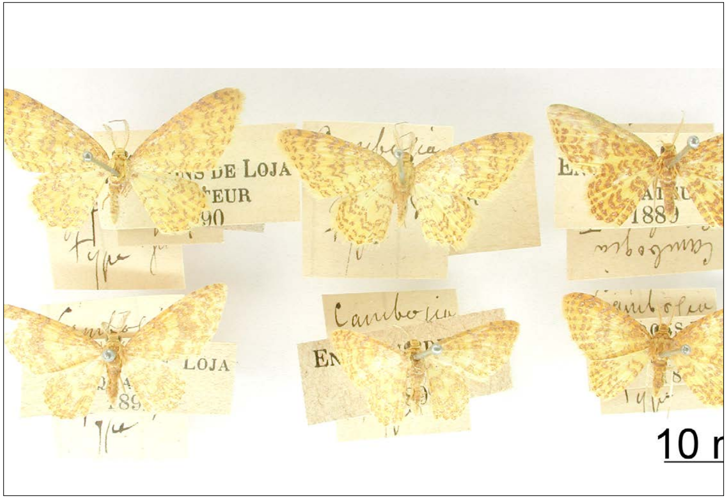

LMR-Geo-
0218

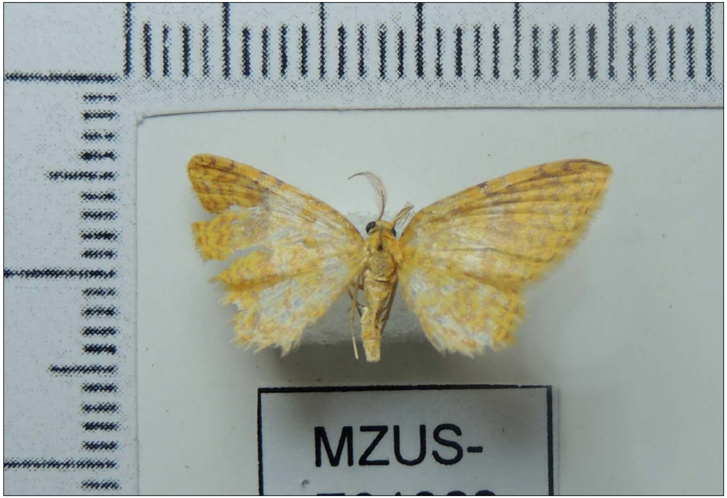

Eois azafranata group Dognin (TL: [Ecuador]: Loja)

BC

BOLD:AEE7400
OTU-068

Additional compared specimen

near GeoCR 35492|Costa Rica|Heredia|BOLD:AAI5234

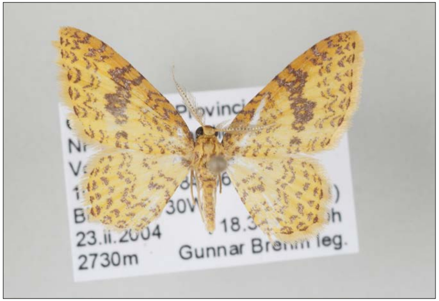

LMR-Geo-

Compared specimen:

USNM types

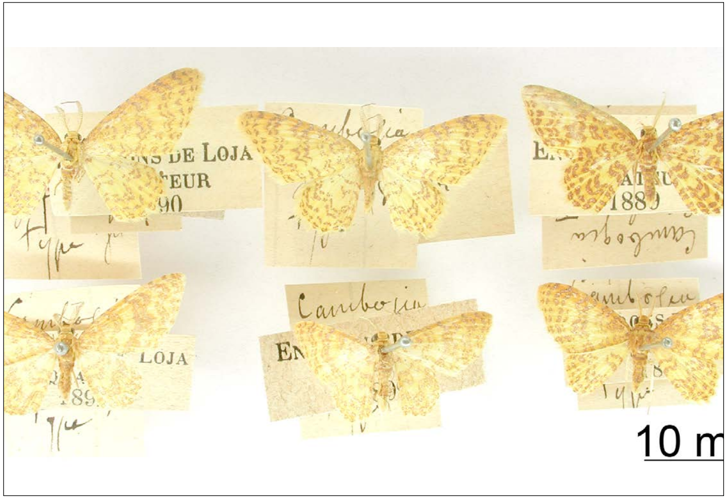

LMR-Geo-

0007

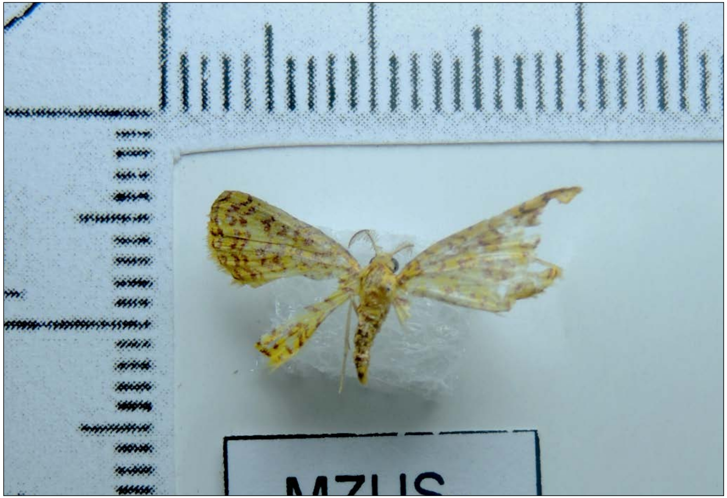

Eois nr burla Dognin (TL: [Ecuador]: Loja)

BC

BOLD:AEE2410

OTU-099

Additional compared specimen
distant: Pe-Geo-3051|Peru|Cuzco|BOLD:ADK0777

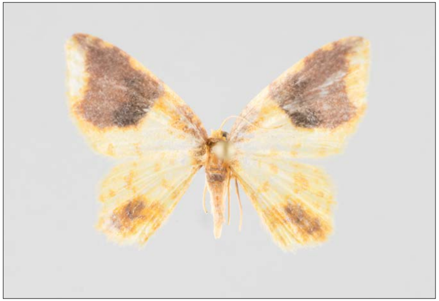

LMR-Geo-

Compared specimen:

USNM type

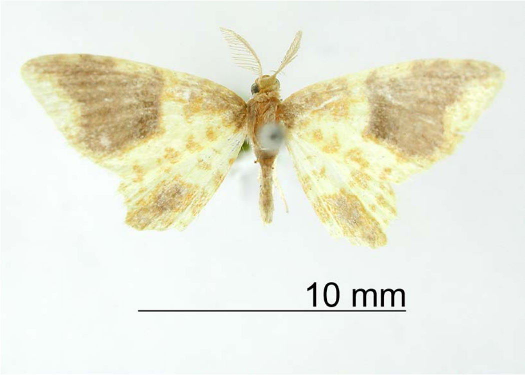

LMR-Geo-

0060

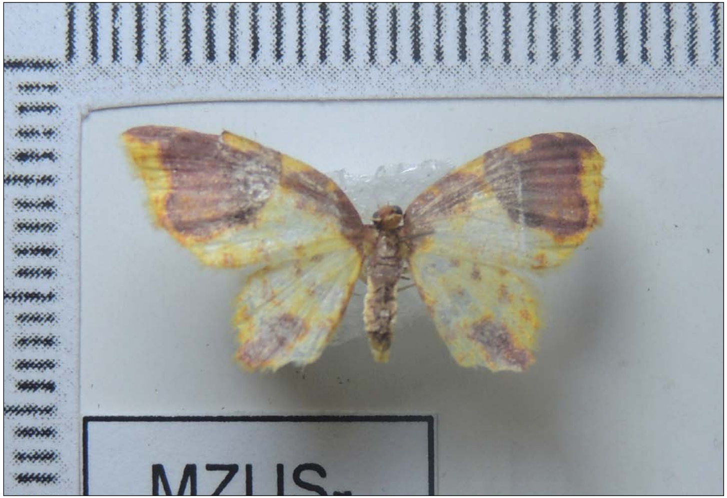

Eois deleta Schaus (TL: Costa Rica: Juan Vinas)

BC
BOLD:AAL0698
OTU-177

Additional compared specimen
= Ec-Geo-45684|Ecuador|Zamora Chinchipe|BOLD:AAL0698

LMR-Geo-

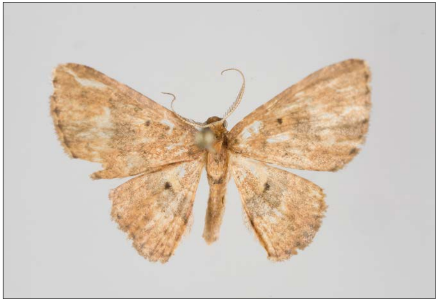

Compared specimen:
USNM type

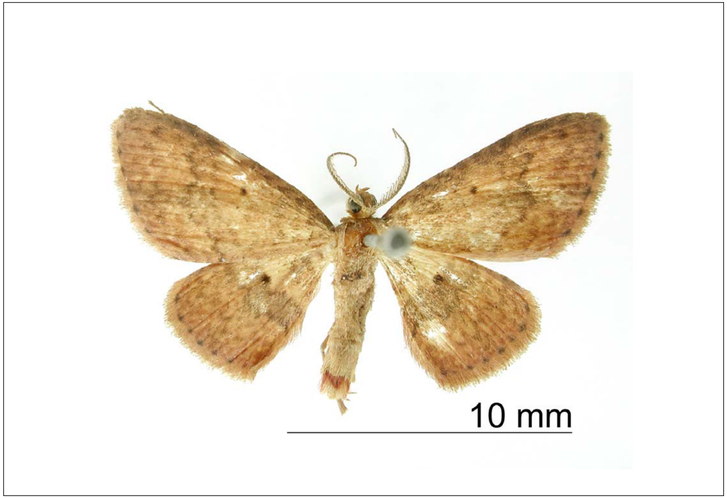

LMR-Geo-
0229

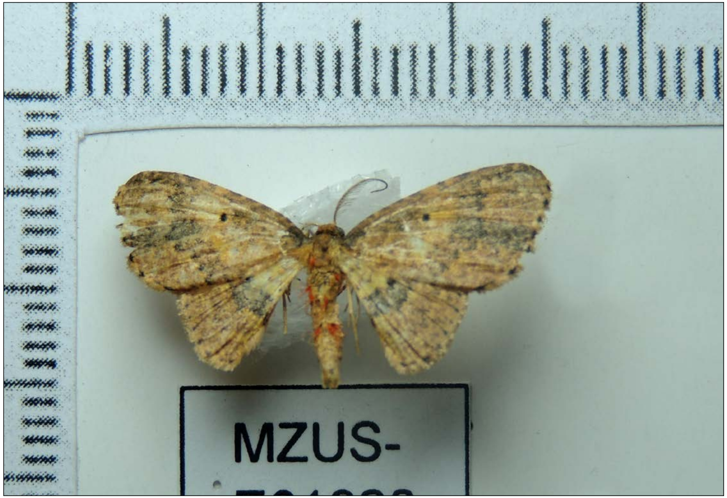

Eois golosata group Dognin (TL: [Ecuador]: Loja)

BC

BOLD:AAM4573
OTU-060

Additional compared specimen
= Ec-Geo-22735|Ecuador|Zamora Chinchipe

LMR-Geo-

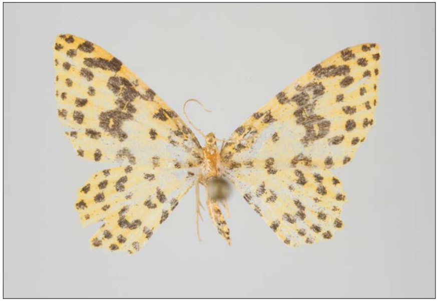

Compared specimen:

USNM types

LMR-Geo-
0004

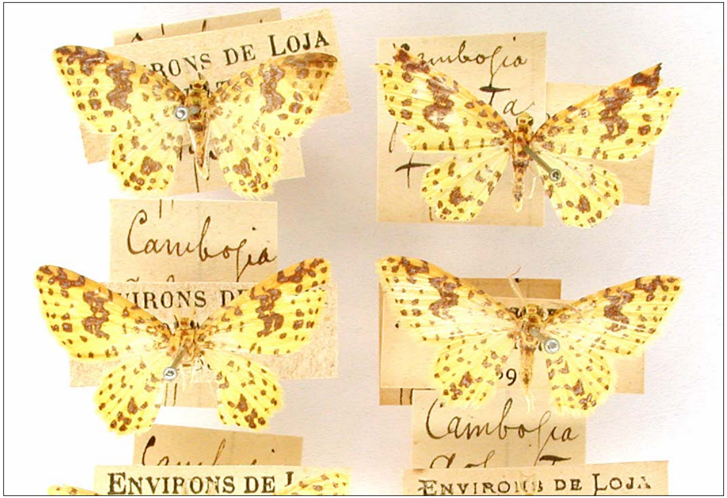

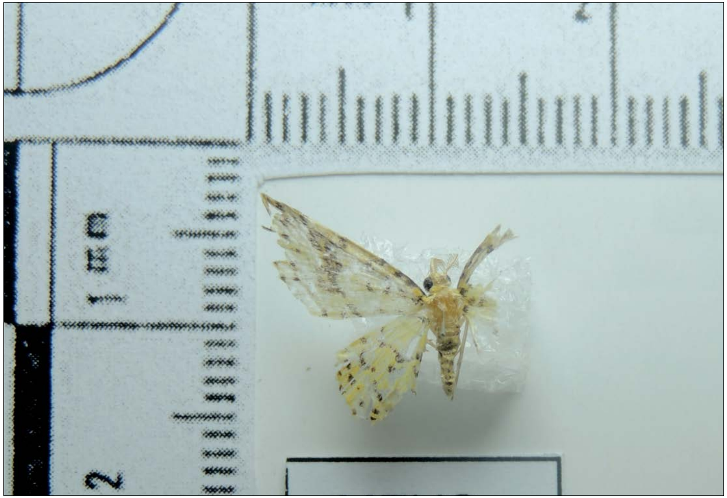

Eois olivacea group Felder & Rogenhofer (TL: [Colombia]: Bogota)

BC

BOLD:AEE7827
OTU-174

Additional compared specimen
distant: Ec-Geo-22224|Ecuador|Zamora Chinchipe|BOLD:AAW5496

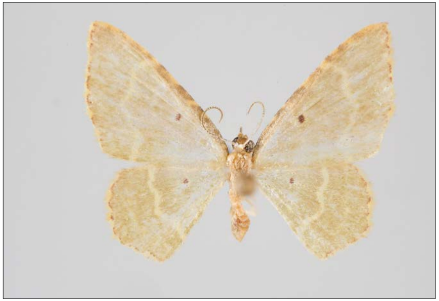

LMR-Geo-
0203, 0204, 0217

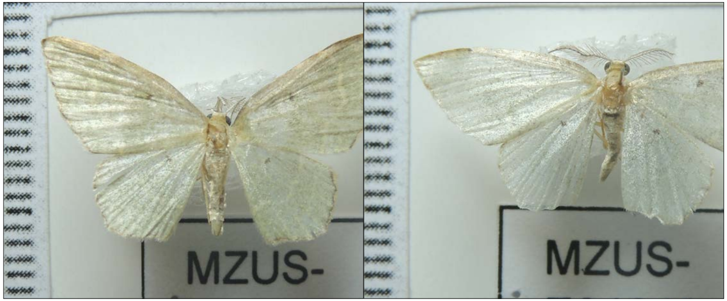

Compared specimen:
NHM type

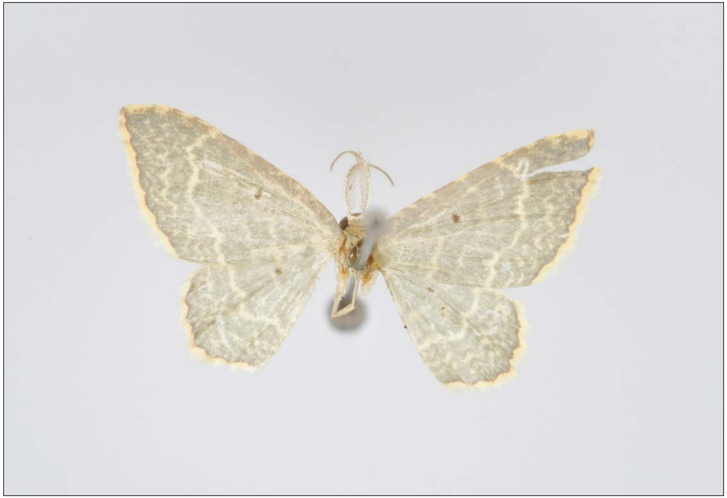

LMR-Geo-
0255

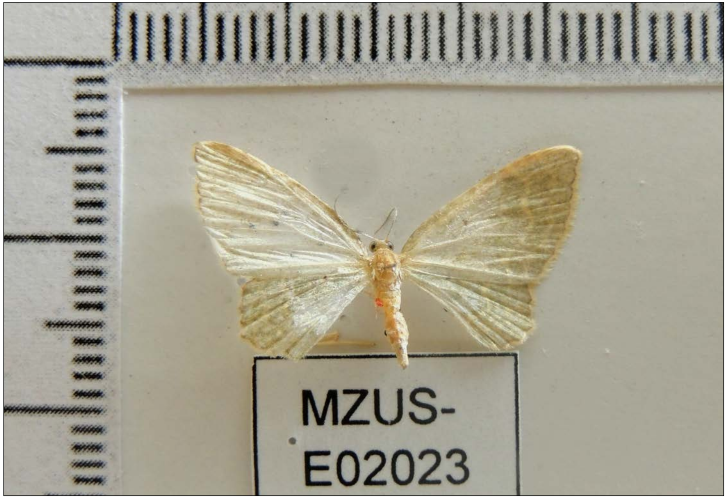

Eois paraviolascens Dognin (TL: [Ecuador]: Loja)

BC

BOLD:AAI5239
OTU-114

Additional compared specimen
= Ec-Geo-22012|Ecuador|Zamora Chinchipe|BOLD:AAI5239

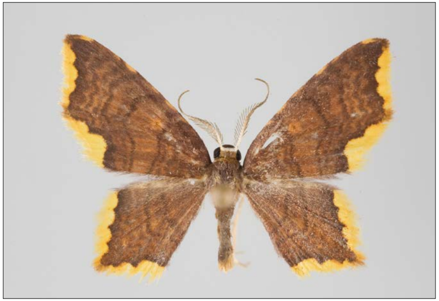

LMR-Geo-

Compared specimen:
USNM type

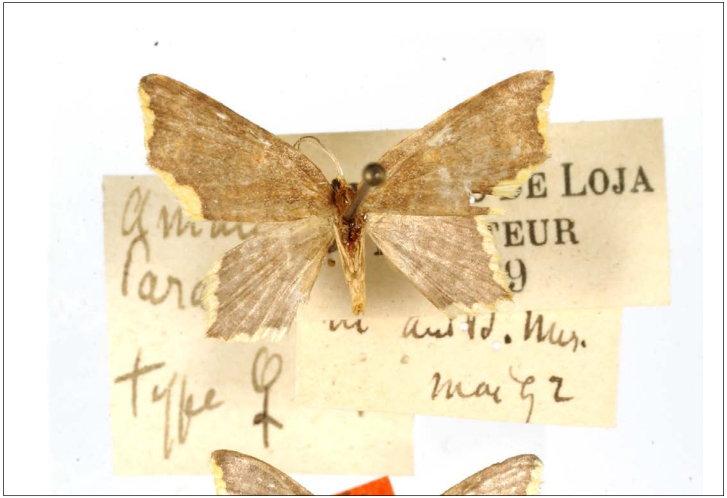

LMR-Geo-
0082

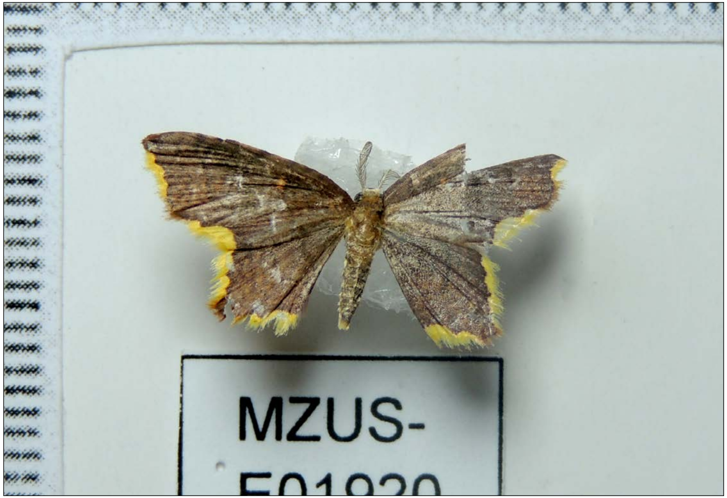

Eois undulosata Warren (TL: Colombia)

BC

BOLD:AAJ0826
OTU-19

Additional compared specimen
= GeoCR 27109|Costa Rica|Heredia|BOLD:AAJ0826

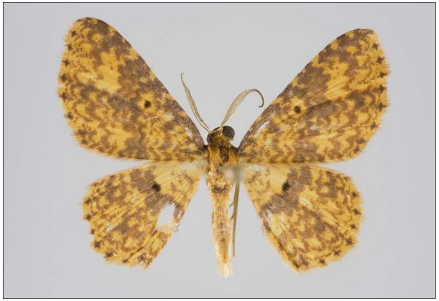

LMR-Geo-

Compared specimen:
NHM type

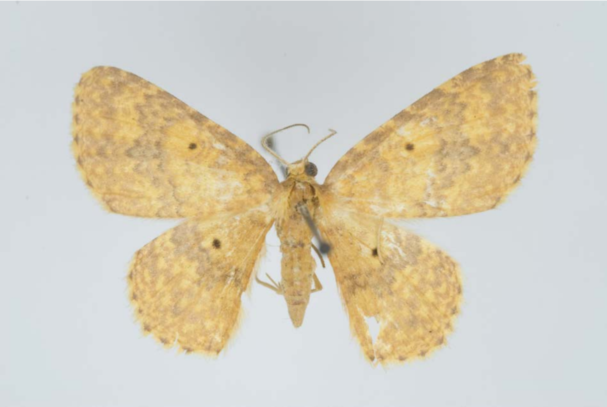

LMR-Geo-
0325

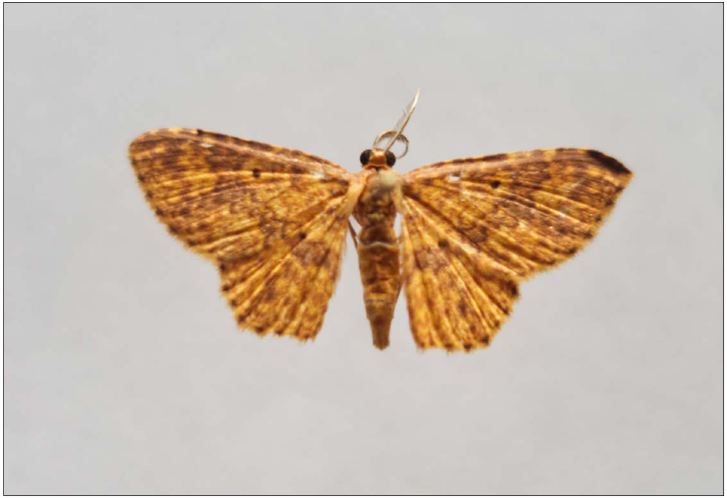

Eois sp (TL:) (not *basaliata*)

BC

BOLD:AAB8409
OTU-155

Additional compared specimen

LMR-Geo-

0166

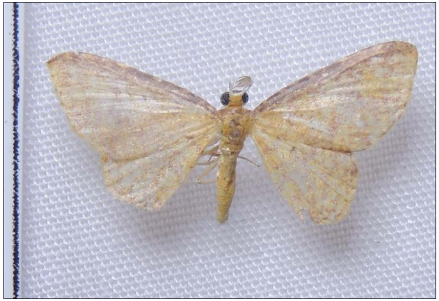

Compared specimen:

LMR-Geo-

0168

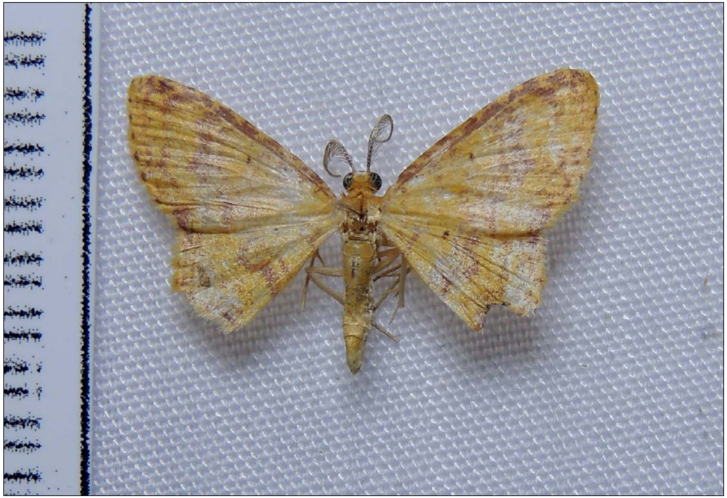

Eois sp (TL:)

BC

BOLD:AEE1125
OTU-156

Additional compared specimen

LMR-Geo-

Compared specimen:

LMR-Geo-
0167

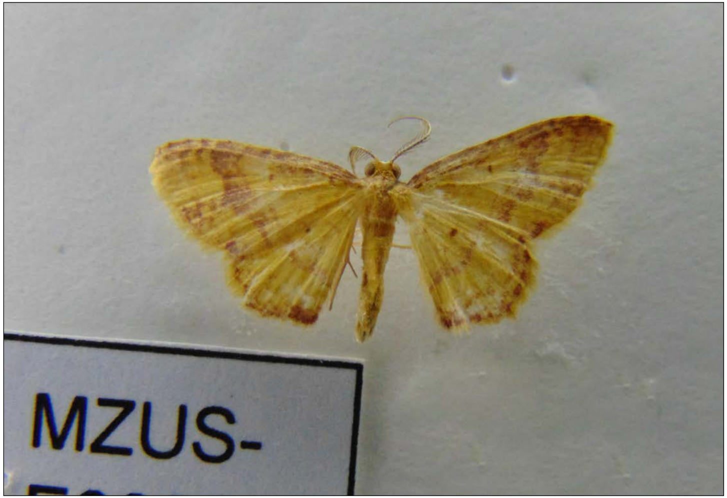

Eudule sp (TL:)

BC

BOLD:AEE2278

OTU-215

Additional compared specimen distant: *ficulnea* Ec-Geo-18630|Ecuador|Zamora Chinchipe|BOLD:AAH6727

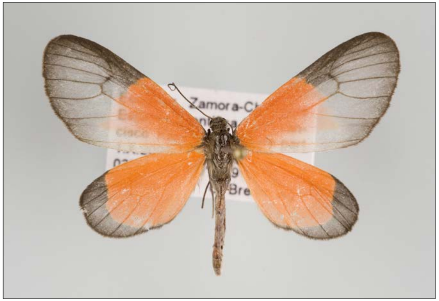

LMR-Geo-

Compared specimen:

LMR-Geo-
0297

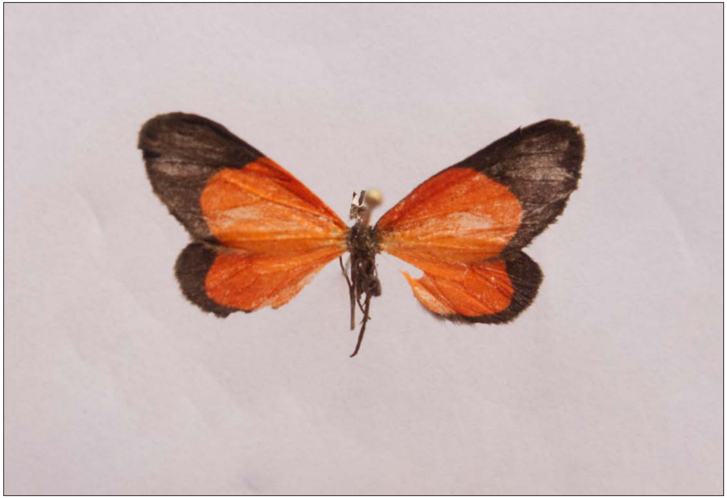

Eupithecia descimoni descimoni Herbulot (TL: Ecuador: 52 km along the Pifo-Baeza road, 2600 m)

BC

BOLD:ABZ5191
OTU-162

Additional compared specimen
= Ec-Geo-22791|Ecuador|Zamora Chinchipe

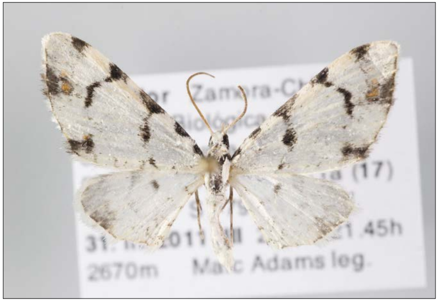

LMR-Geo-

Compared specimen:

ZSM type

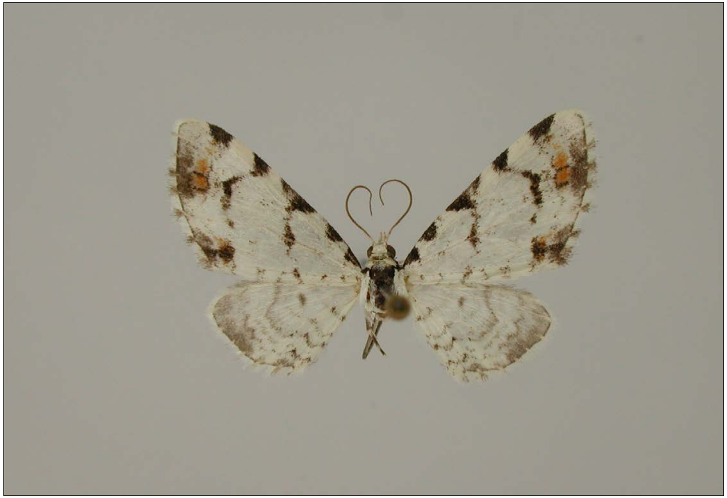

LMR-Geo-

0208

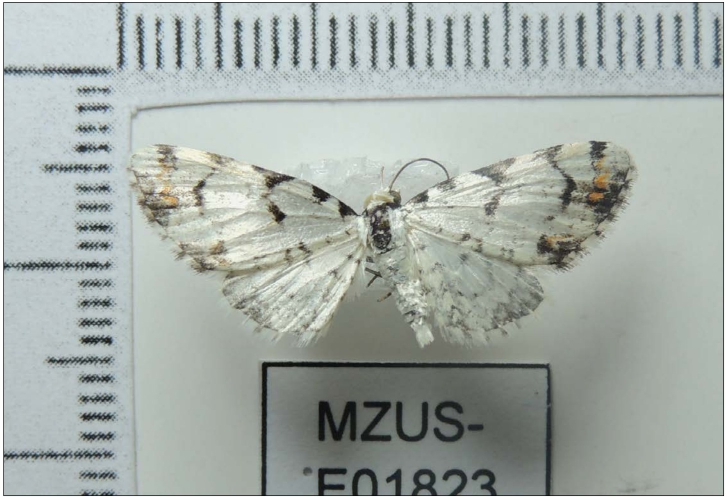

Eupithecia galepsa Herbulot (TL: Bolivia: La Paz, La Paz-Rio Songo road, 2050 m)

BC

BOLD:ACJ9646

OTU-183

Additional compared specimen
= Pe-Geo-0905|Peru|Cuzco

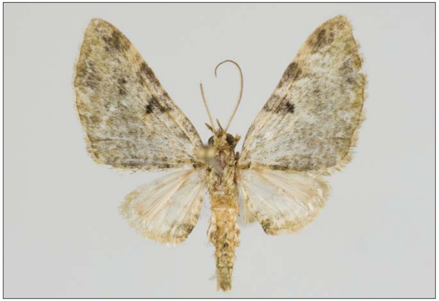

LMR-Geo-

Compared specimen:

ZSM type

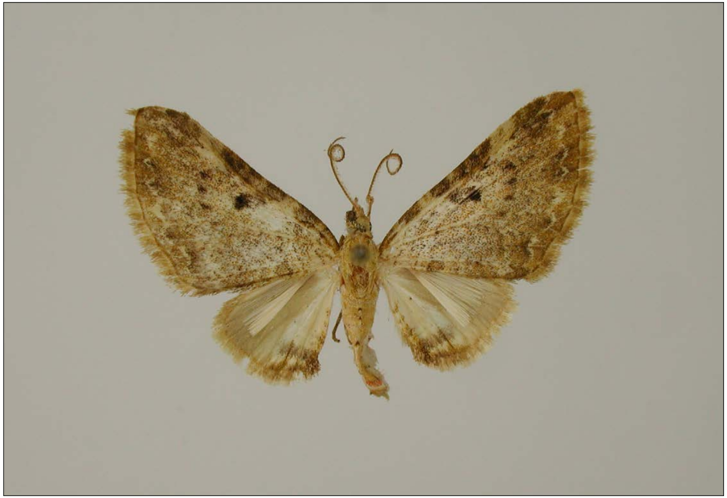

LMR-Geo-

0232

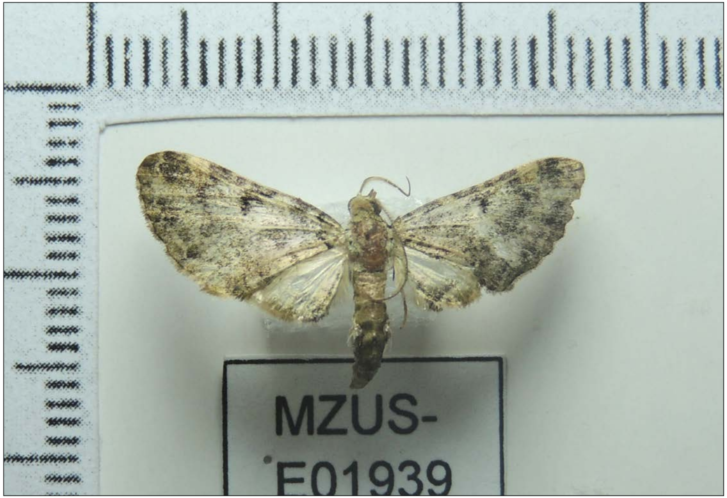

Eupithecia madura group Dognin (TL: Colombia: Micay)

BC

BOLD:AEE7495
OTU-176

Additional compared specimen
near Ec-Geo-16387|Ecuador|Zamora Chinchipe|BOLD:AAI5038

LMR-Geo-

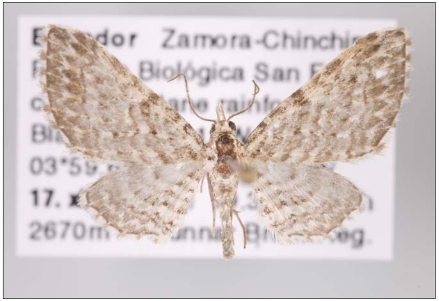

Compared specimen:
USNM type

LMR-Geo-
0228

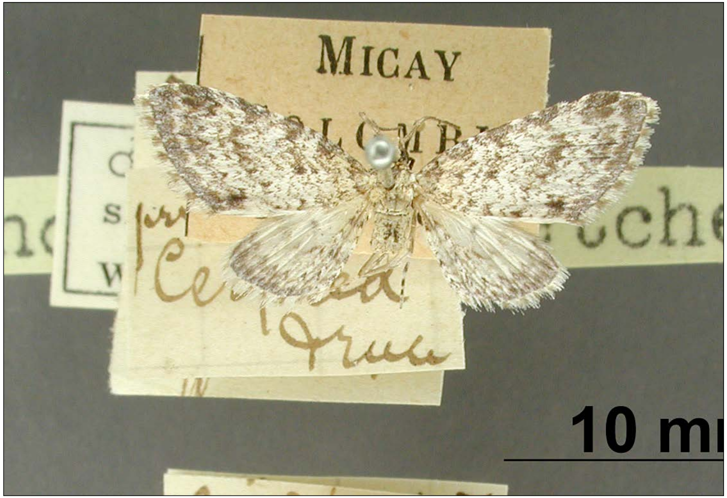

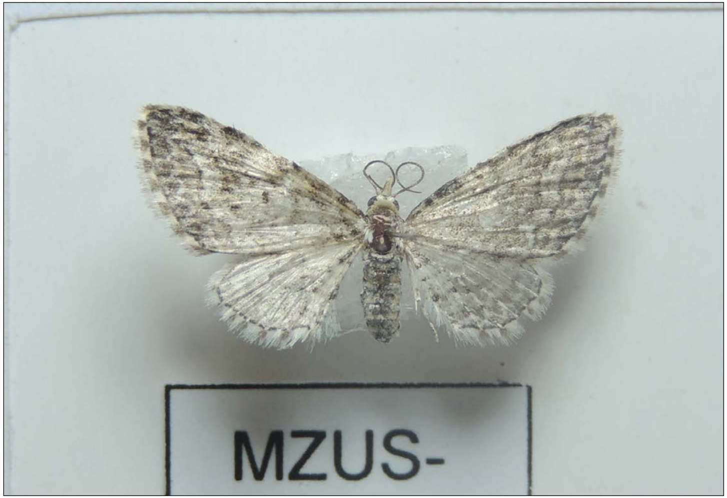

Eupithecia nr penicilla Dognin (TL: [Ecuador]: Loja)

BC

BOLD:AAI5029

OTU-193

Additional compared specimen
= Ec-Geo-15692|Ecuador|Zamora Chinchipe|BOLD:AAI5029

LMR-Geo-

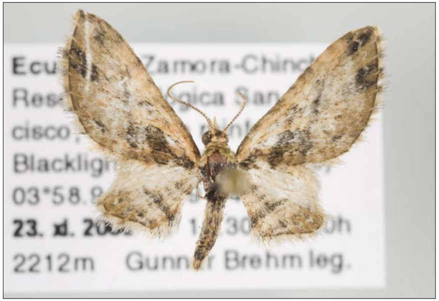

Compared specimen:

USNM type

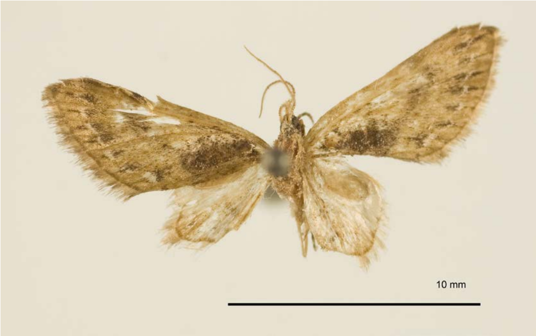

LMR-Geo-

0259

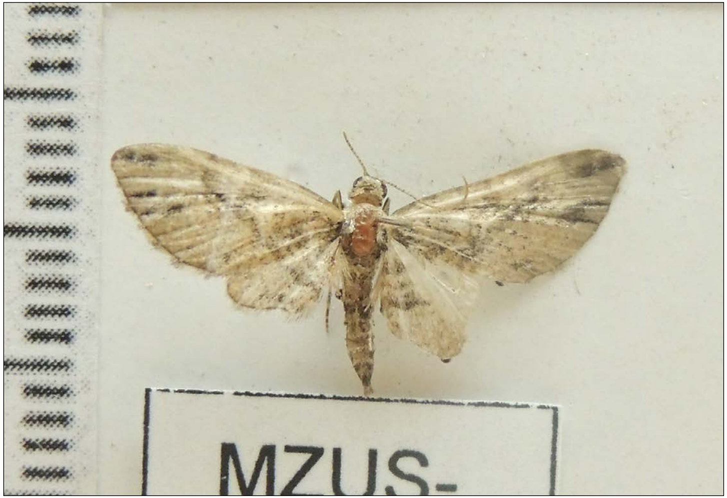

Eupithecia nr rubellicincta Warren (TL: Peru (south-east): Carabaya, Santo Domingo, 6000 ft)

BC

BOLD:ABW8857

OTU-186

Additional compared specimen
= Ec-Geo-19261|Ecuador|Zamora Chinchipe

LMR-Geo-

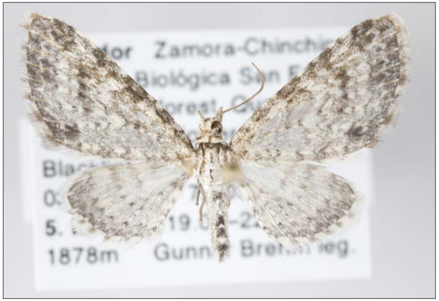

Compared specimen:

NHM type

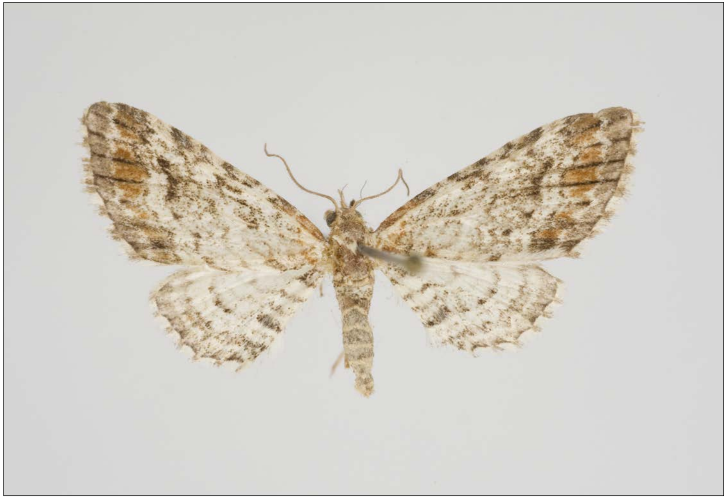

LMR-Geo-
0215

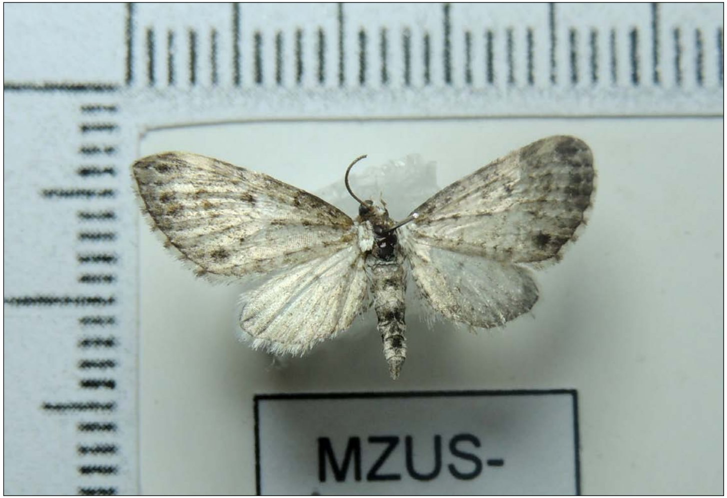

Eupithecia nr sorda Dognin (TL: [Ecuador]: Loja)

BC
BOLD:AEE9437
OTU-62

Additional compared specimen
distant: Ec-Geo-7944|Ecuador|Zamora Chinchipe|407[2n]

LMR-Geo-

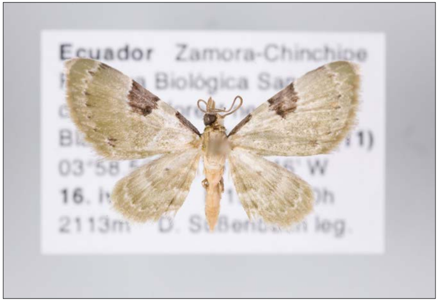

Compared specimen:
USNM type

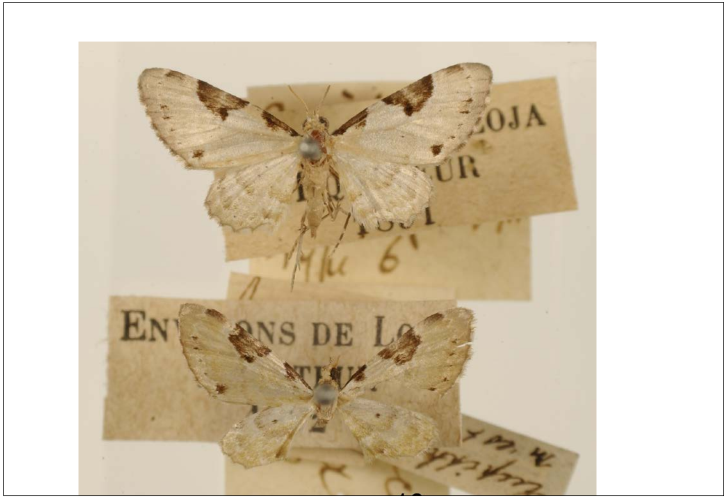

LMR-Geo-
0002

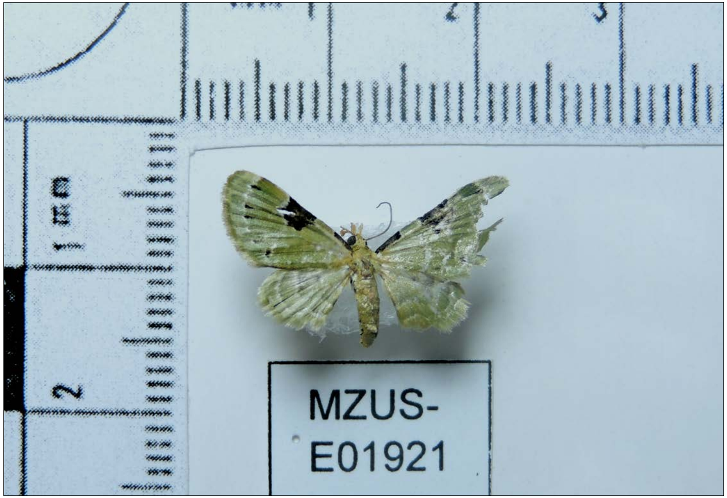

Eupithecia yangana Dognin (TL: [Ecuador]: Loja, El Monje; Yangana)

BC

BOLD:AAI5026
OTU-221

Additional compared specimen
= Ec-Geo-19902|Ecuador|Zamora Chinchipe

LMR-Geo-

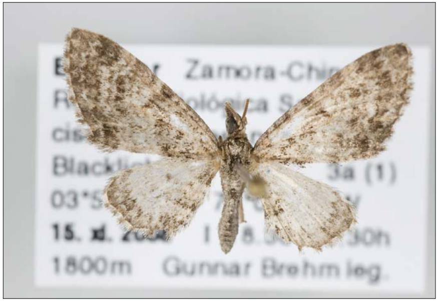

Compared specimen:
USNM types

LMR-Geo-
0264

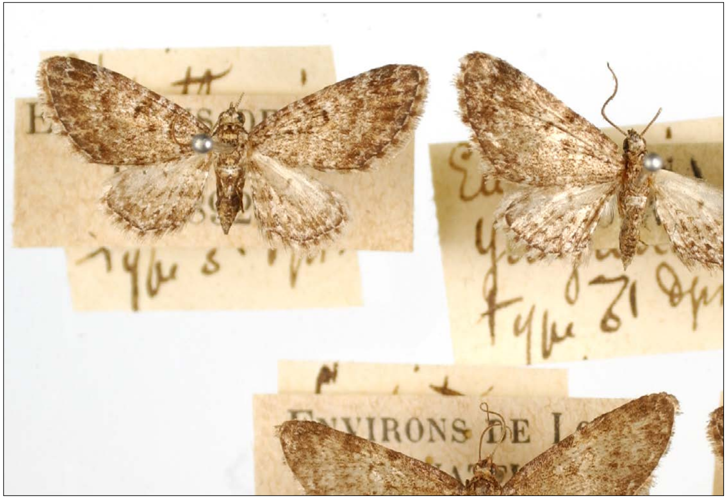

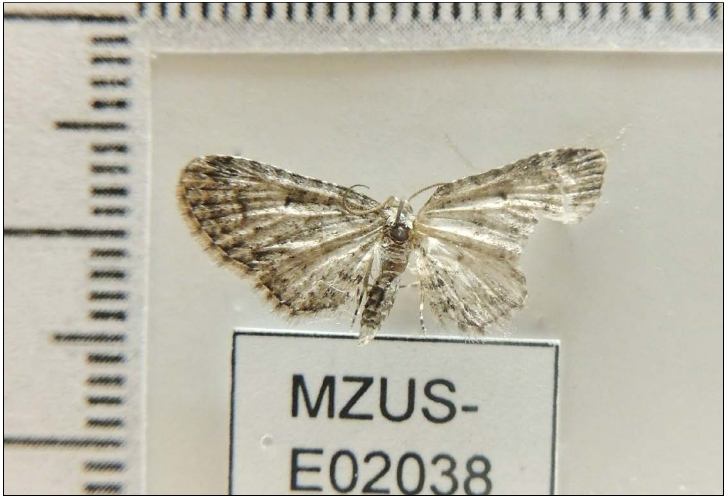

Eupithecia sp (TL:)

BC
BOLD:AEE8255
OTU-65

Additional compared specimen

no close relatives

LMR-Geo-

Compared specimen:

LMR-Geo-

0015

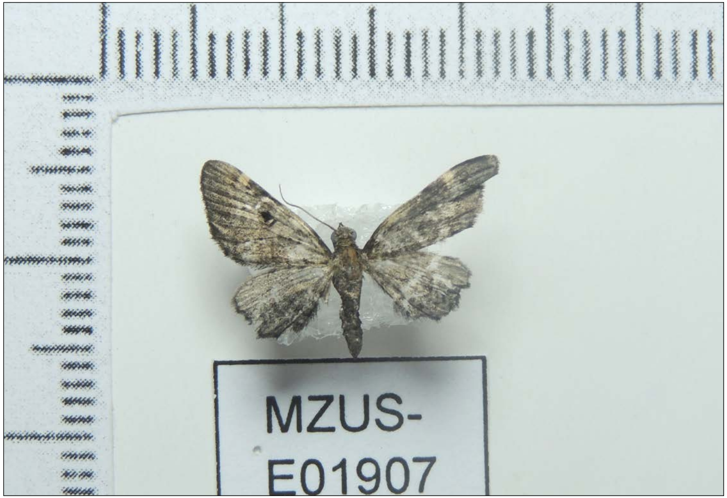

Eupithecia sp (TL:)

BC

BOLD:AAA0696
OTU-66

Additional compared specimen

LMR-Geo-

Compared specimen:

LMR-Geo-

0016

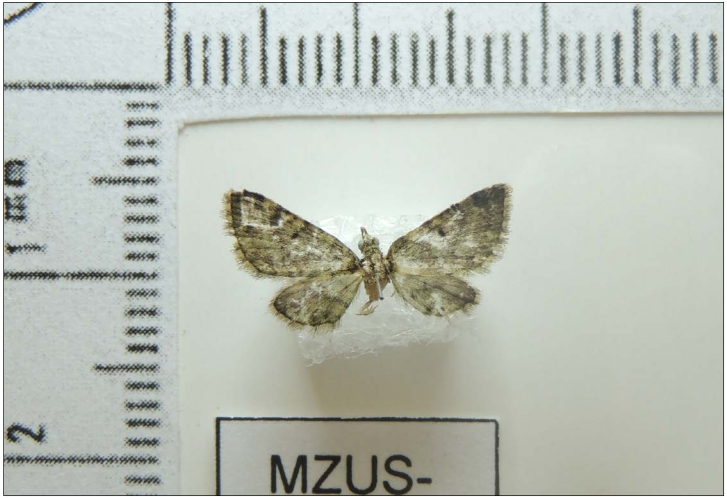

Eupithecia sp (TL:)

BC

BOLD:AAI5023

OTU-185

Additional compared specimen
= Ec-Geo-17025|Ecuador|Zamora Chinchipe

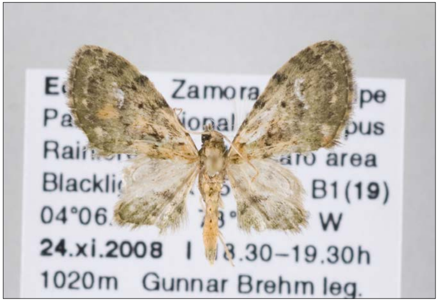

LMR-Geo-

Compared specimen:

LMR-Geo-
0219

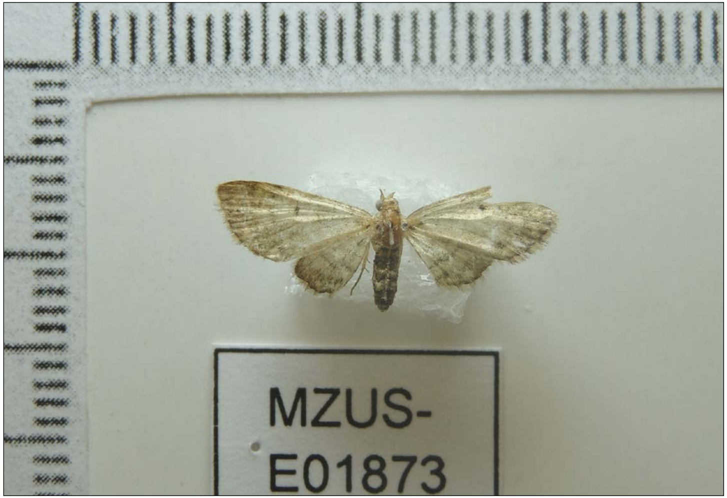

Eupithecia sp (TL:)

BC

BOLD:AEE8035
OTU-202

Additional compared specimen
distant Ec-Geo-22775|Ecuador|Zamora Chinchipe|BOLD:AAW5506

LMR-Geo-

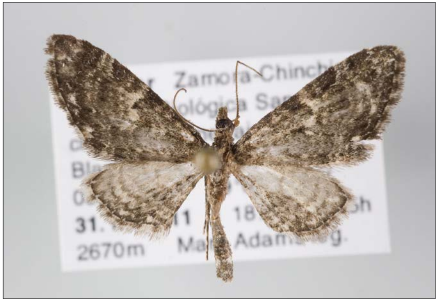

Compared specimen:

LMR-Geo-
0251

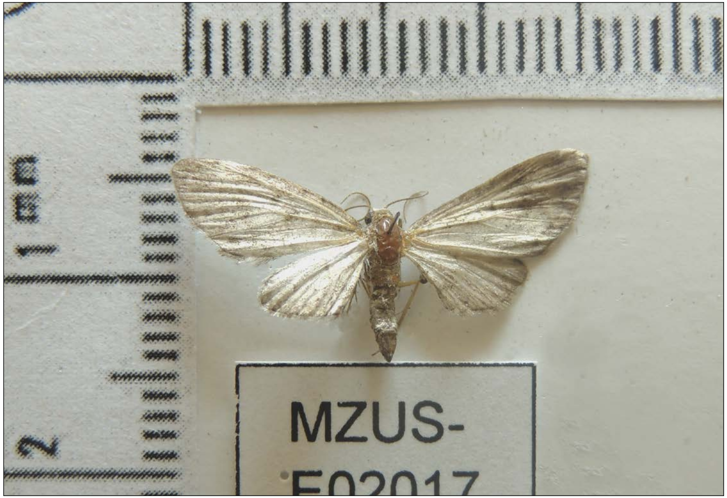

Eupithecia sp (TL:)

BC
BOLD:ADV0858
OTU-196

Additional compared specimen
distant: Ec-Geo-19274|Ecuador|Zamora Chinchipe|BOLD:AAJ0152

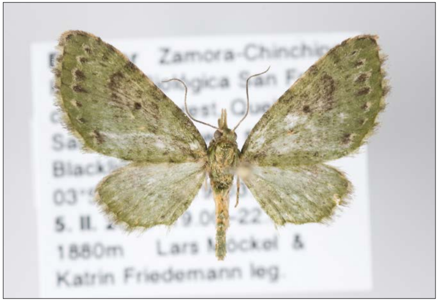

LMR-Geo-

Compared specimen:

LMR-Geo-
0254

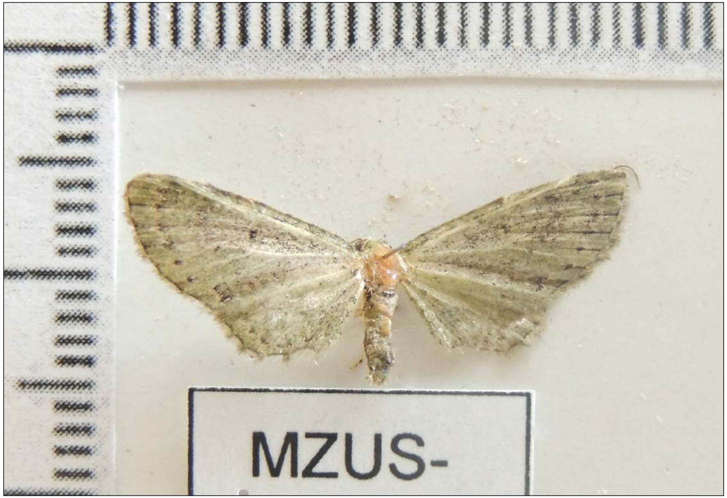

Eupithecia sp (TL:)

BC

BOLD:AAI5027
OTU-192

Additional compared specimen
= Pe-Geo-3519|Peru|Cuzco

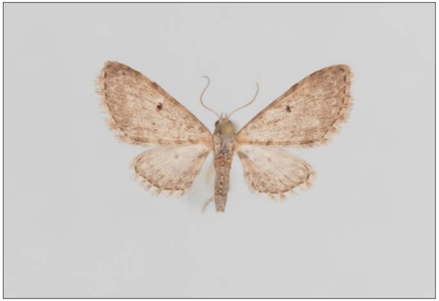

LMR-Geo-

Compared specimen:

LMR-Geo-
0260

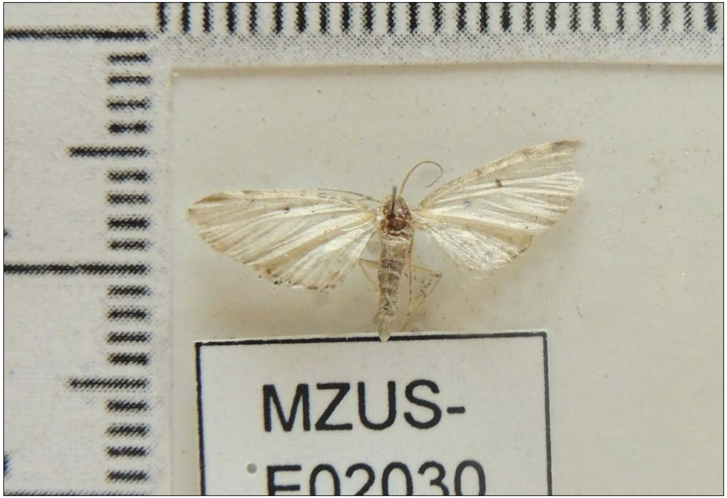

Eupithecia sp (TL:)

BC

BOLD:AEE3262
OTU-220

Additional compared specimen

LMR-Geo-

Compared specimen:

LMR-Geo-
0263

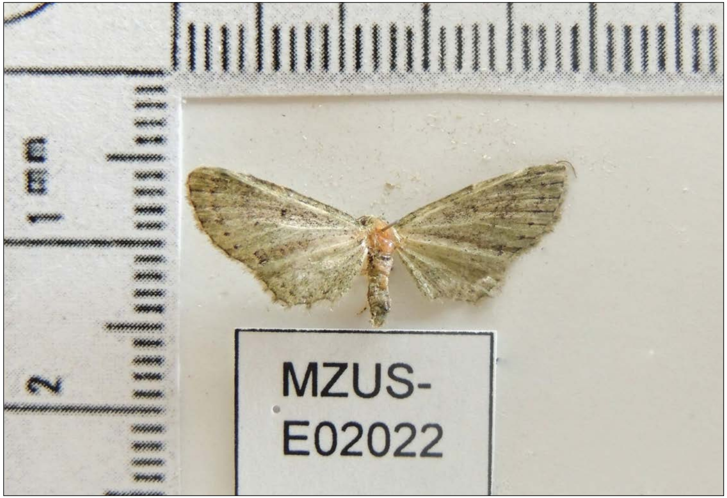

Eupithecia sp (TL:)

BC

BOLD:AEB9461

OTU-8

Additional compared specimen
near: Ec-Geo-18095|Ecuador|Loja|BOLD:AAI5069

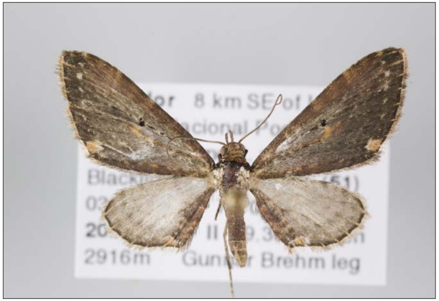

LMR-Geo-
0311, 0315

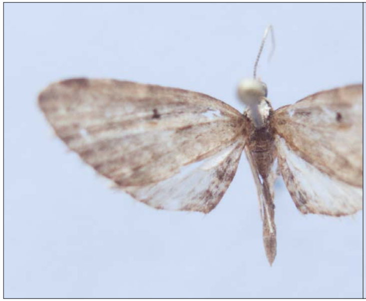

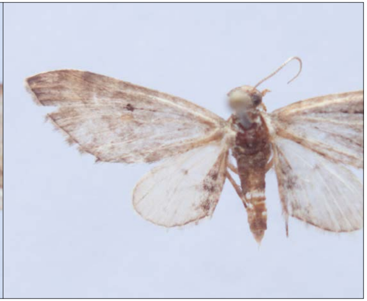

Compared specimen:

LMR-Geo-
0319

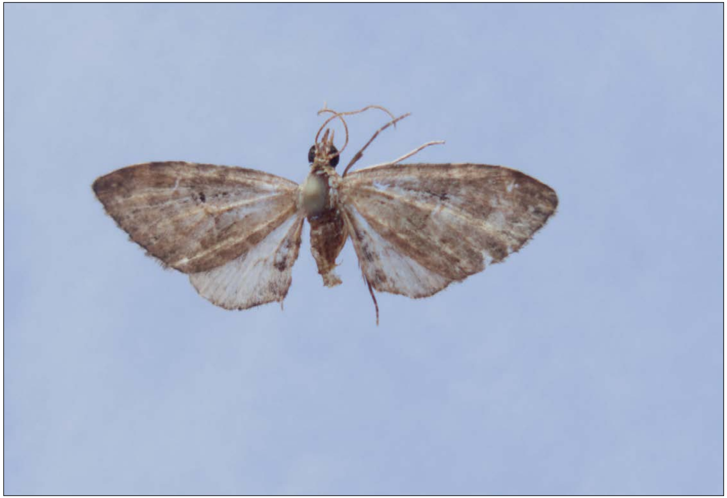

Eupithecia sp (TL:)

BC

BOLD:AEB9461
OTU-10

Additional compared specimen
near: Ec-Geo-18095|Ecuador|Loja|BOLD:AAI5069

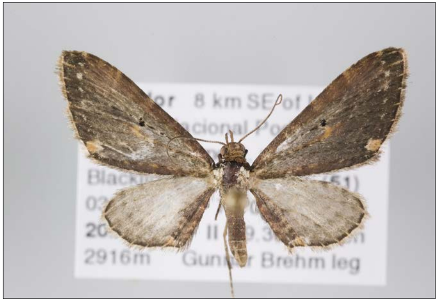

LMR-Geo-

Compared specimen:

LMR-Geo-
0313

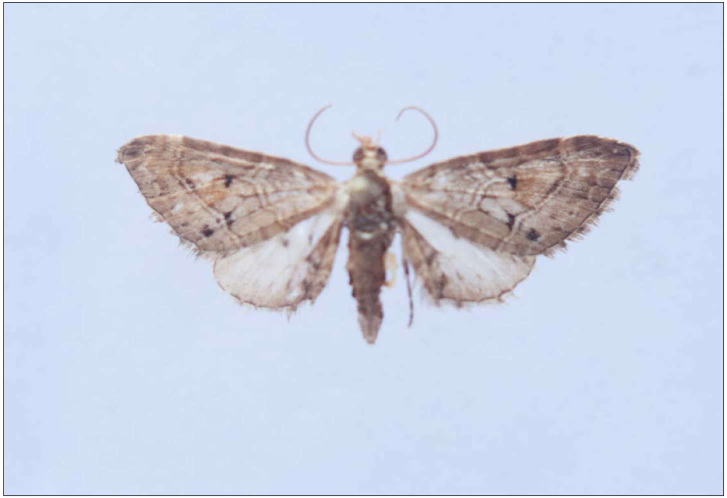

Eupithecia sp (TL:)

BC

BOLD:AEB8759
OTU-10

Additional compared specimen near Ec-Geo-22402|Ecuador|Loja|BOLD:AAW1261

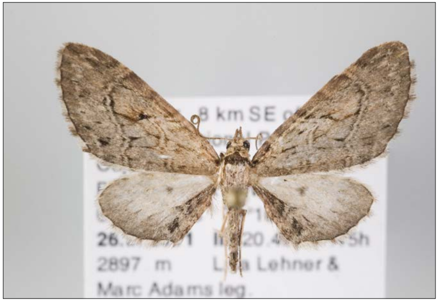

LMR-Geo-

Compared specimen:

LMR-Geo-
0320

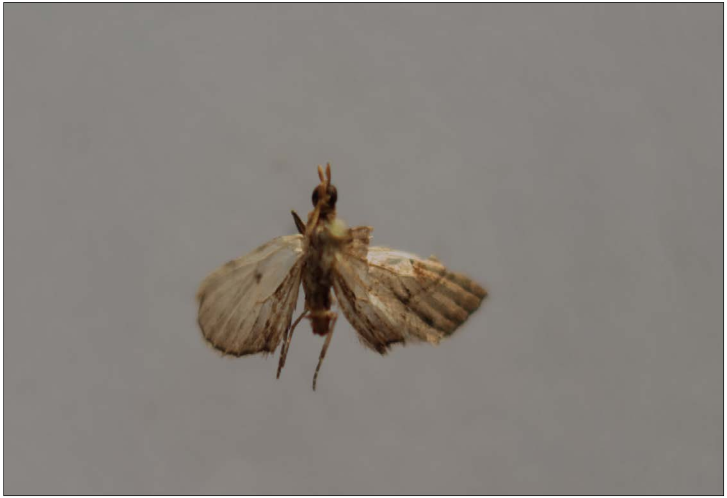

Hydriomena sp (TL:)

BC

BOLD:AEE0666
OTU-206

Additional compared specimen

LMR-Geo-

Compared specimen:

LMR-Geo-
0245

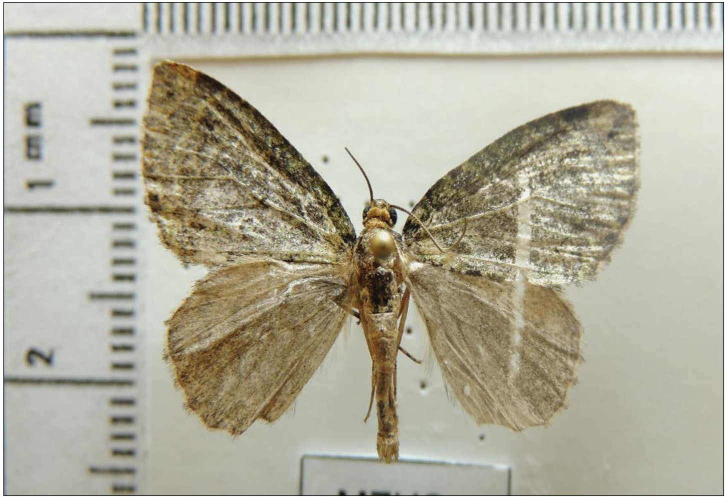

Oligopleura sp (TL:) next match 92% on BOLD

BC

BOLD:AEE1113

OTU-229

Additional compared specimen

LMR-Geo-

Compared specimen:

LMR-Geo-

0169

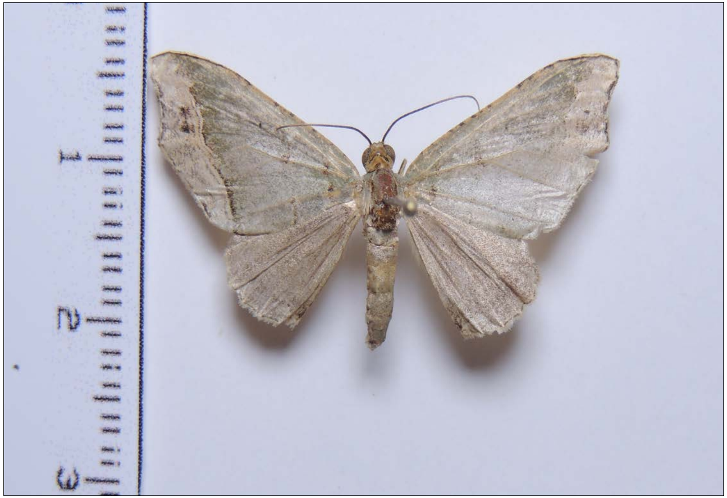

Orthonama majorata Dognin (TL: [Colombia]: near Cali; Paramo del Quindin; Rio Toche; Monte Socorro)

BC

BOLD:AEE1510
OTU-14

Additional compared specimen

LMR-Geo-

Compared specimen:

USNM type

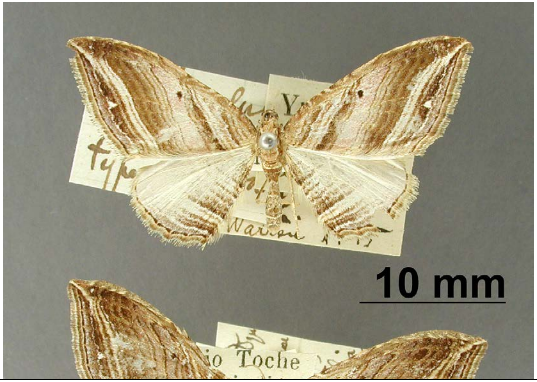

LMR-Geo-

0321

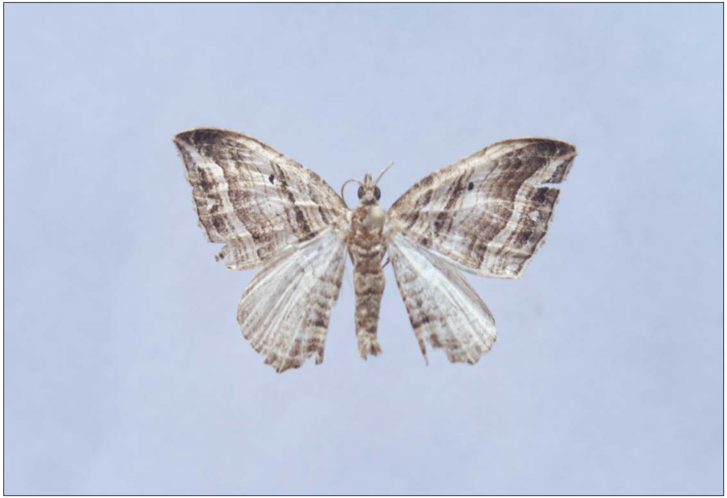

NN Larentiinae (next match *Chrysolarentia* 92%)

BC
BOLD:AEE1510
OTU-87

Additional compared specimen

LMR-Geo-

Compared specimen:

LMR-Geo-
0033

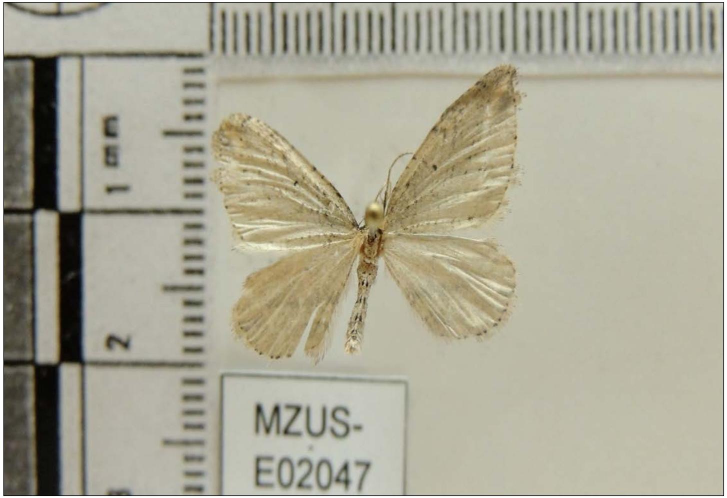

NN Larentiinae (probably undescribed genus)

BC

BOLD:AEE5590

OTU-167

Additional compared specimen

LMR-Geo-

Compared specimen:

LMR-Geo-

0211

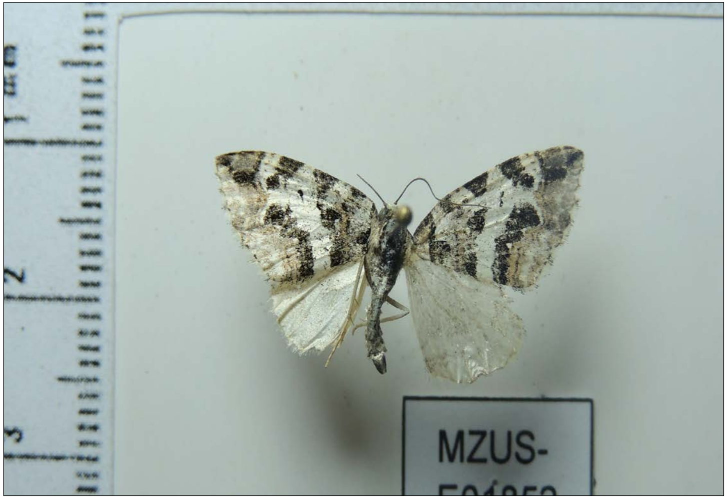

NN Larentiinae (probably undescribed genus)

BC

BOLD:AEE7431

OTU-219

Additional compared specimen

LMR-Geo-

Compared specimen:

LMR-Geo-

0292

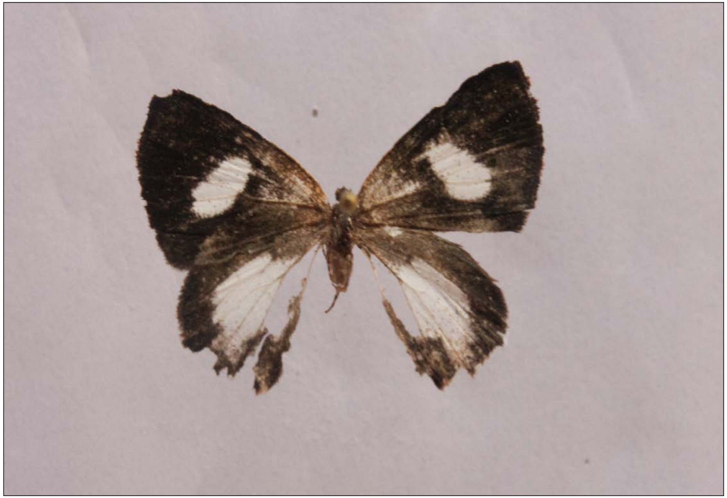

Sterrhinae

Cyclophora nodigera group Butler (TL: Brazil, ‚Amazons', Pariti Rio Purus; Rio Jurua)

BC

BOLD:AAF7873
OTU-23

Additional compared specimen
= GeoCR 31191|Costa Rica|Heredia|BOLD:AAF7873

LMR-Geo-

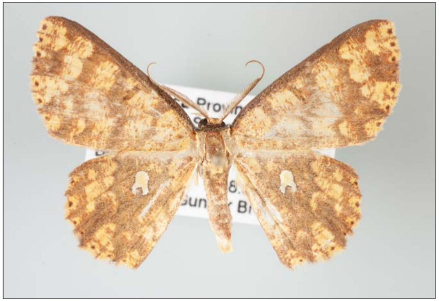

Compared specimen:
NHM type

LMR-Geo-
0329

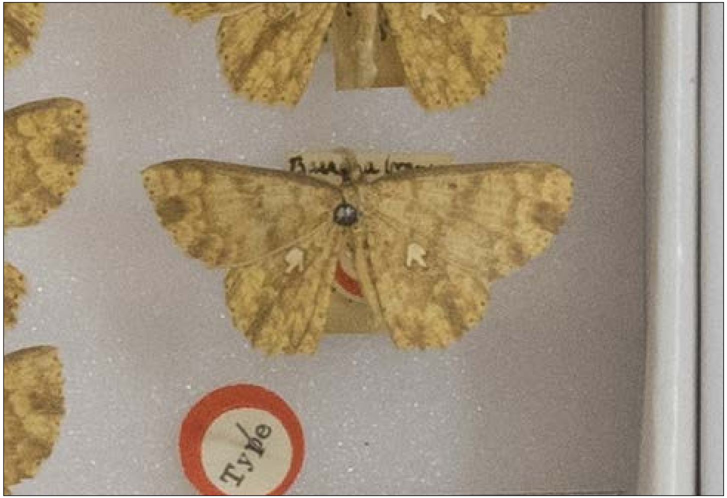

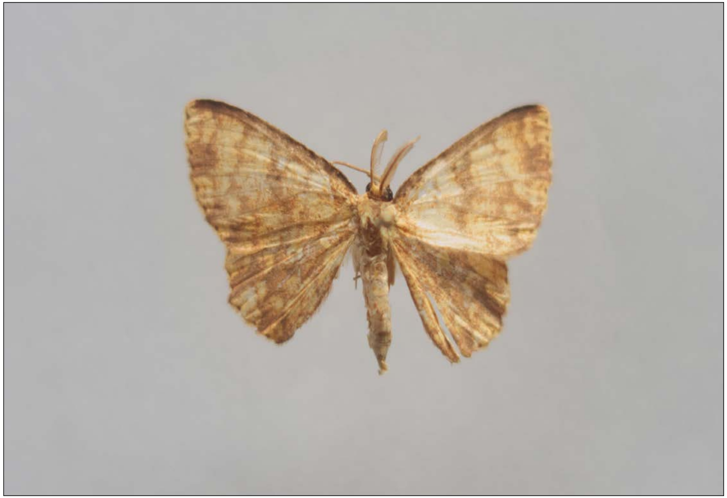

Cyclophora sp (TL:)

BC

BOLD:AEC1114
OTU-20

Additional compared specimen
no close relative

LMR-Geo-

Compared specimen:

LMR-Geo-
0328

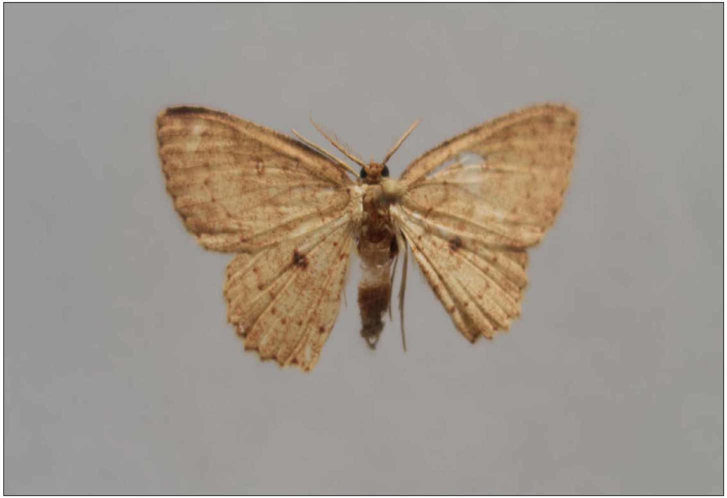

Cyclophora sp (TL:)

BC

BOLD:AEC1262
OTU-44

Additional compared specimen

LMR-Geo-

Compared specimen:

LMR-Geo-

0361

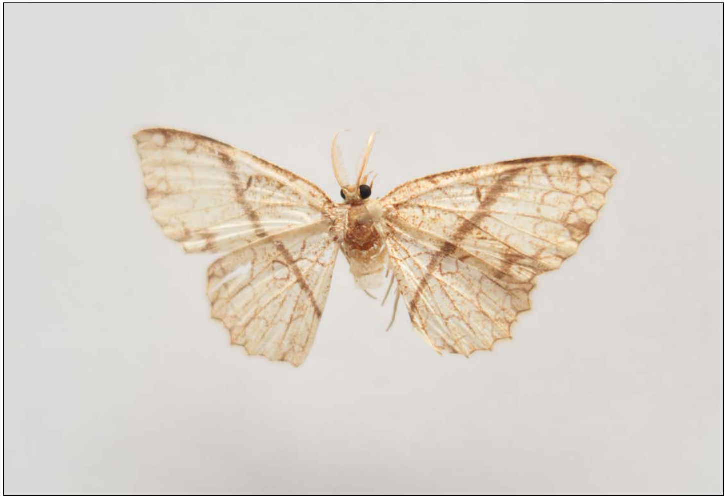

Idaea nr asceta Prout (TL: Argentina, Gran Chaco near Florenzia.)

BC

BOLD:AEE6969

OTU-147

Additional compared specimen

LMR-Geo-

Compared specimen:

NHM type

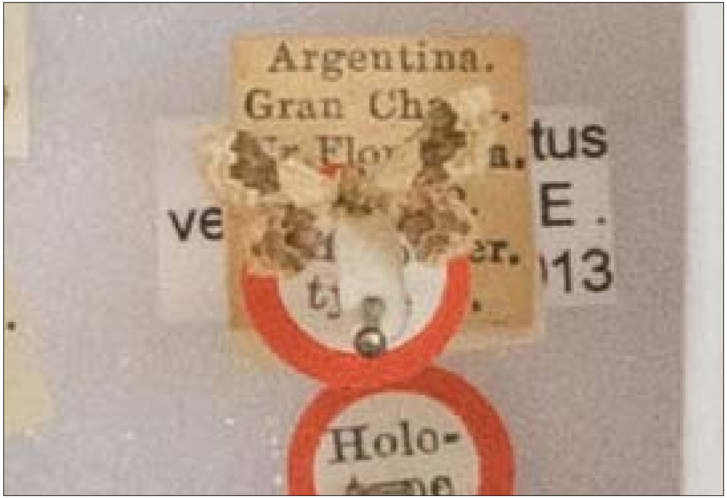

LMR-Geo-

0182

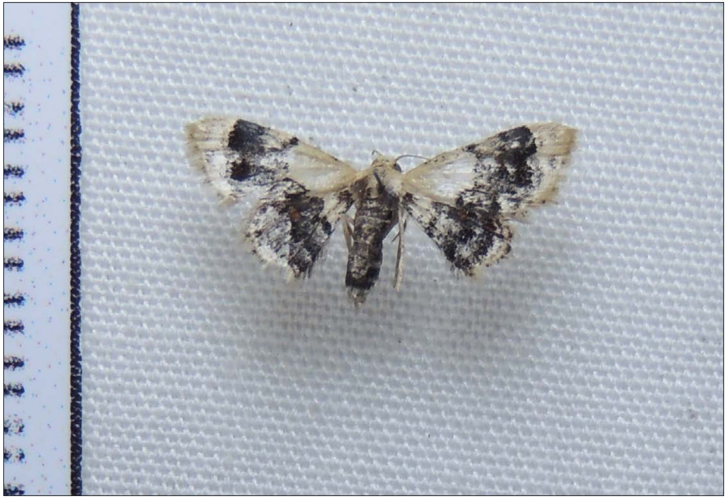

Idaea nr arhostiodes Warren (TL: Peru, Colorado, 2500 ft)

BC

BOLD:AEE2177

OTU-144

Additional compared specimen

LMR-Geo-
0173 (no photo)

Compared specimen:
NHM type

LMR-Geo-
0179

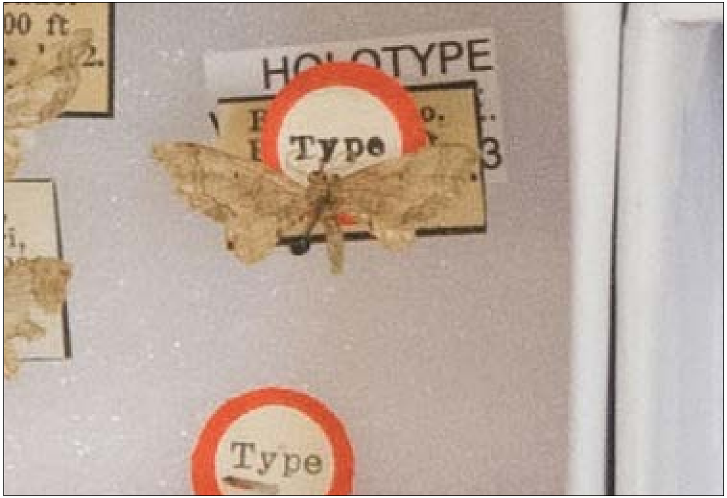

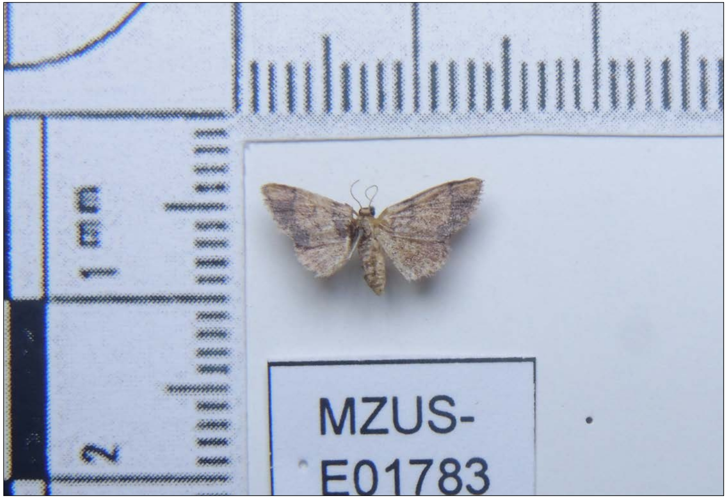

Idaea arhostiodes group Warren (TL: Peru, Colorado, 2500 ft)

BC

BOLD:AAW9693
OTU-64

Additional compared specimen
= Ec-Geo-14866|Ecuador|Zamora Chinchipe

LMR-Geo-

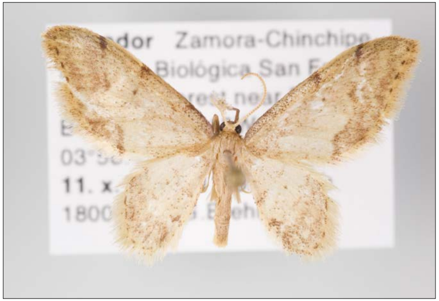

Compared specimen:
NHM type

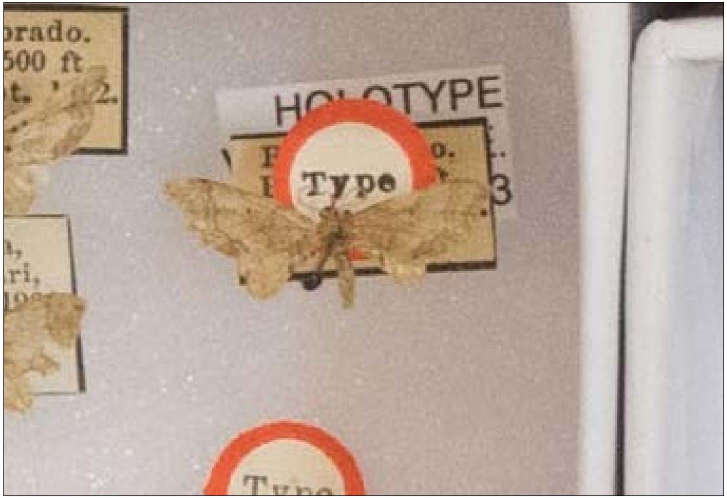

LMR-Geo-
0014

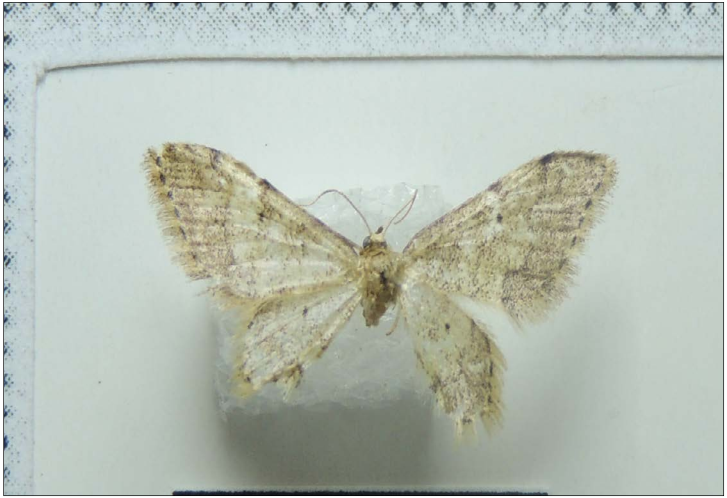

Idaea nr fervens Butler (TL: [Brazil]: Amazonas, Rio Tapajos; Rio Jurua; Rio Jutahi)

BC

BOLD:AAB6314
OTU-17

Additional compared specimen

LMR-Geo-
0269 (no photo)

Compared specimen:
NHM type

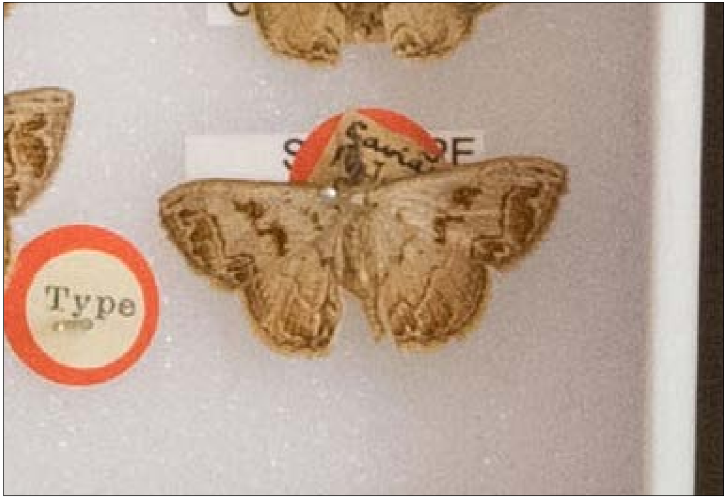

LMR-Geo-
0323

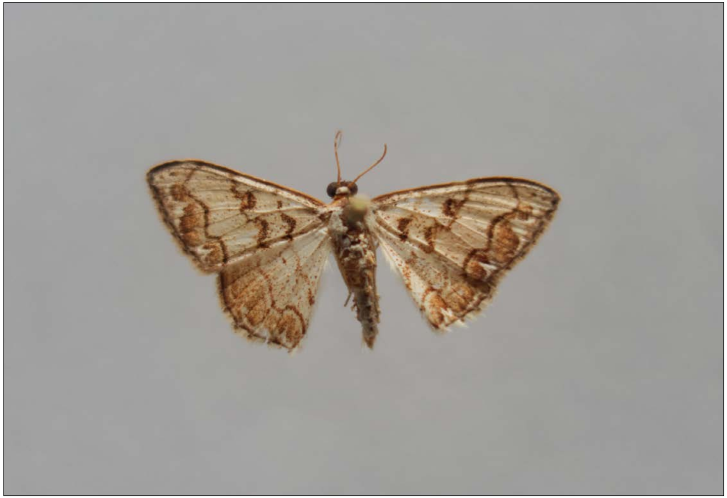

Idaea nr conifer Warren (TL: French Guiana: Cayenne)

BC

BOLD:AEE5658
OTU-158

Additional compared specimen

LMR-Geo-0243 (no photo)

Compared specimen:

USNM type

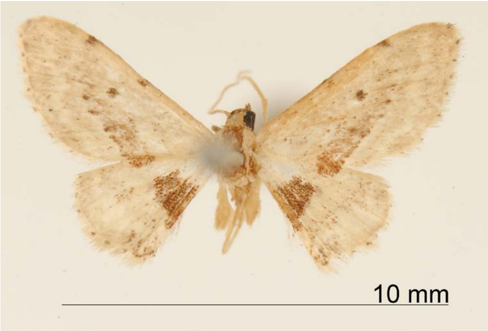

LMR-Geo-0177

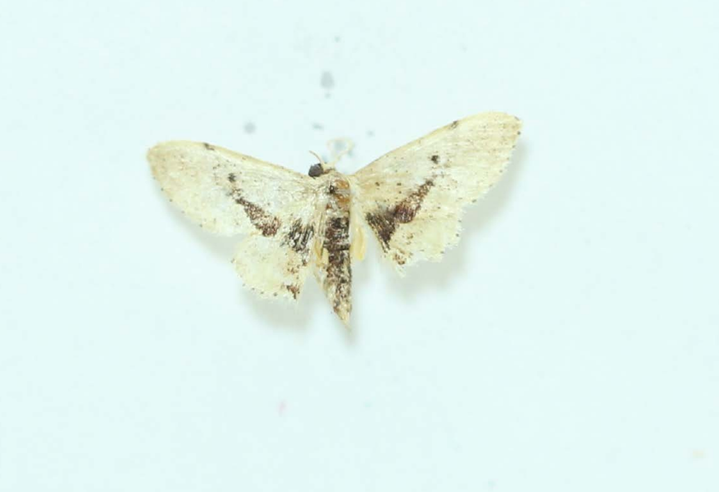

Idaea nr fimbriata Warren (TL: Ecuador: Chimbo, 1000 ft)

BC

BOLD:AEE8476	
OTU-228	

Additional compared specimen

LMR-Geo-

Compared specimen:

NHM type

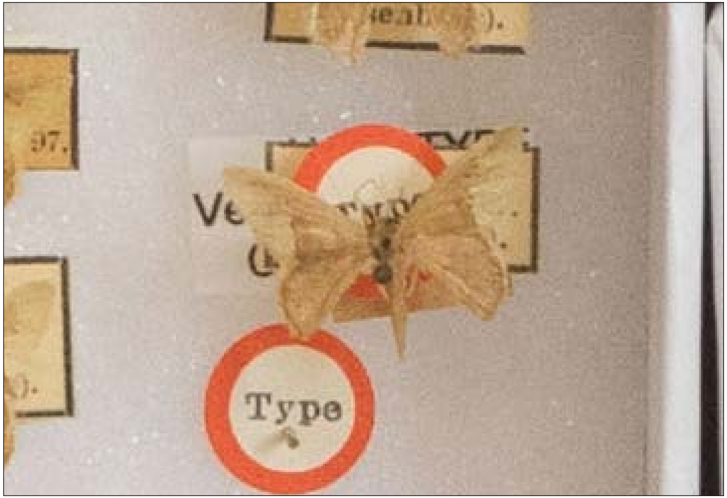

LMR-Geo-

0174

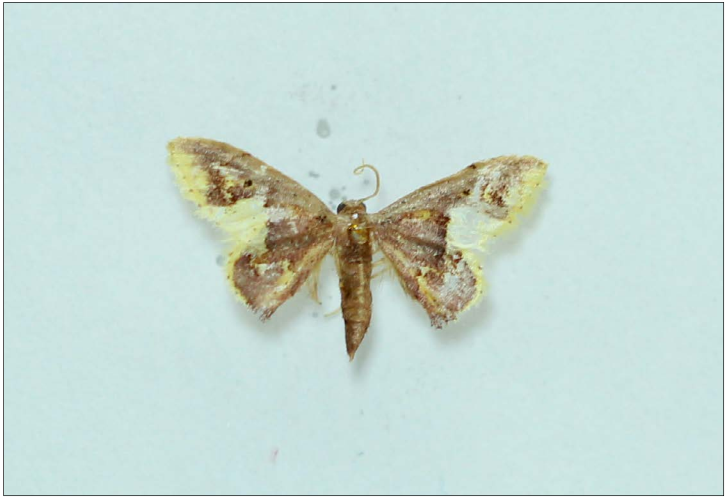

Idaea nr macouma Schaus (TL: Peru; Aroa; Venezuela)

BC

BOLD:AAK4254

OTU-175

Additional compared specimen
near: Pe-Geo-0395|Peru|Cuzco|BOLD:AAE2732

LMR-Geo-

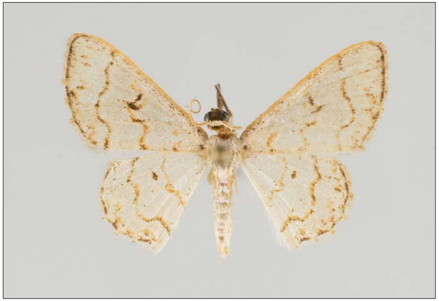

Compared specimen:

USNM type

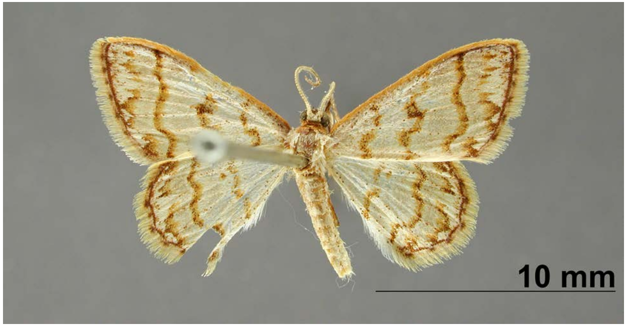

LMR-Geo-

0201

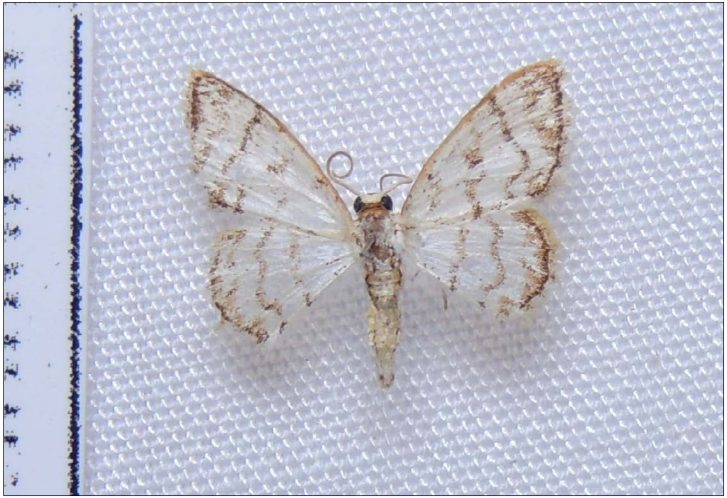

Idaea sp (TL:)

BC

BOLD:AEE7632
OTU-126

Additional compared specimen

LMR-Geo-
0102 (no photo)

Compared specimen:
NHM type

LMR-Geo-
0185

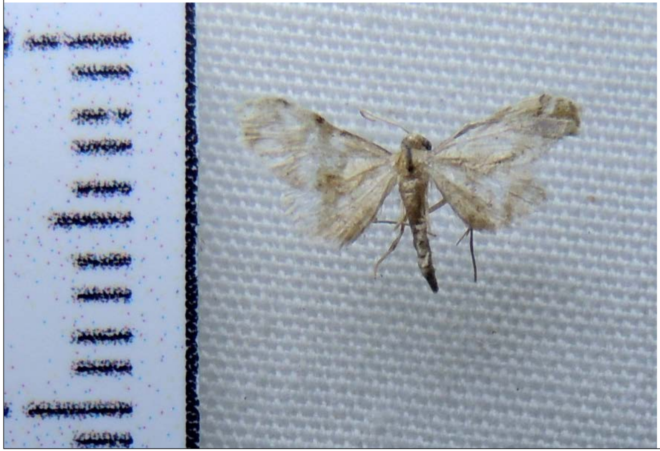

Idaea sp (TL:)

BC

BOLD:AEE2176	
OTU-70	

Additional compared specimen

LMR-Geo-0009

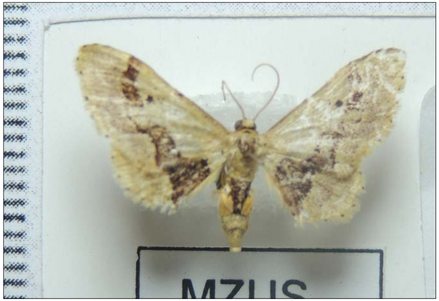

Compared specimen:

LMR-Geo-0010

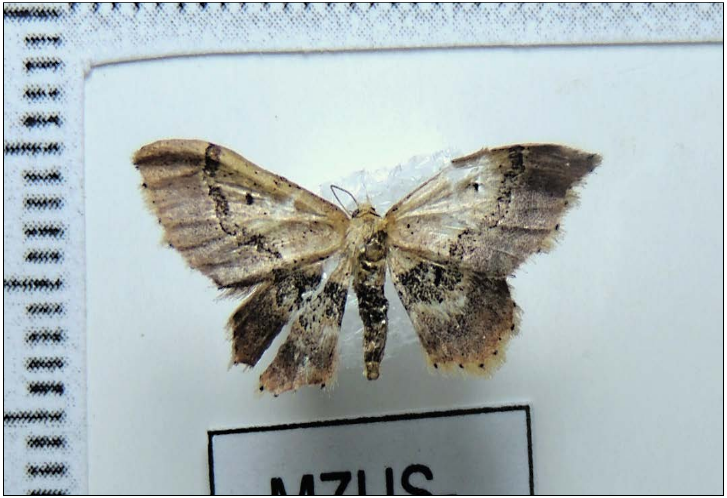

Idaea sp (TL:)

BC

BOLD:AEE2178
OTU-63

Additional compared specimen

LMR-Geo-
0013, 0006

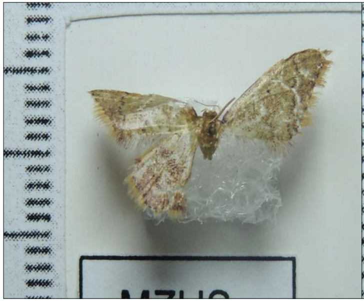

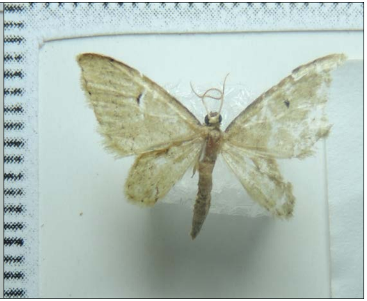

Compared specimen:

LMR-Geo-
0001

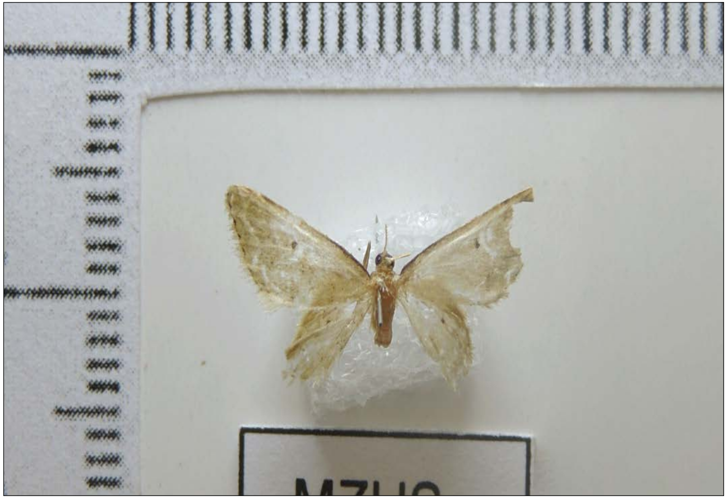

Idaea sp (TL:)

BC

BOLD:AEB7925
OTU-39

Additional compared specimen

LMR-Geo-

Compared specimen:

LMR-Geo-

0347

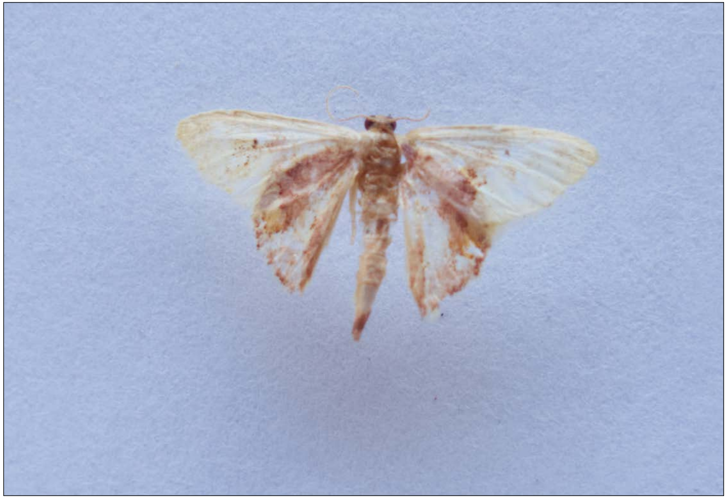

Idaea sp (TL:)

BC

BOLD:AEE2175
OTU-71

Additional compared specimen
distant: Ec-Geo-14873|Ecuador|Zamora Chinchipe|BOLD:AAI2289

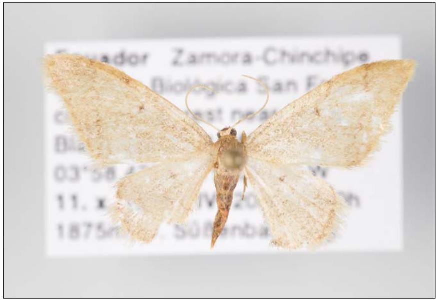

LMR-Geo-
0011

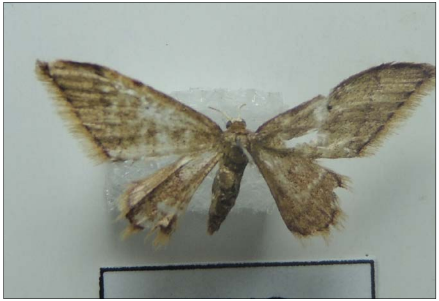

Compared specimen:

LMR-Geo-
0012

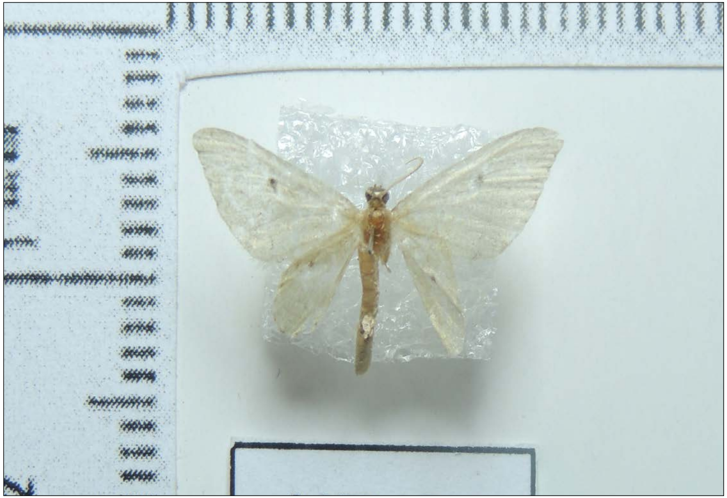

Idaea sp (TL:)

BC

BOLD:AEE9933
OTU-157

Additional compared specimen
distant: Ec-Geo-19063|Ecuador|Zamora Chinchipe|BOLD:ACJ9266

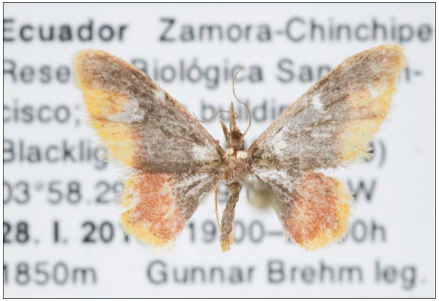

LMR-Geo-

Compared specimen:

LMR-Geo-
0175

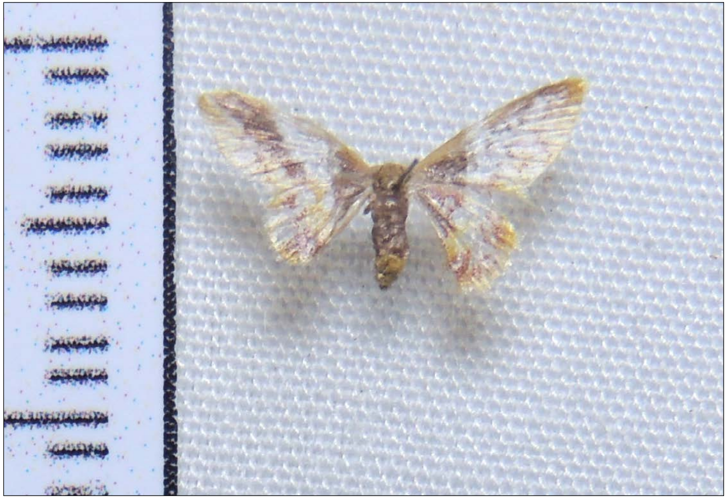

Idaea sp (TL:)

BC

BOLD:AEE2173
OTU-146

Additional compared specimen
distant: Pe-Geo-1278|Peru|Huanuco|BOLD:ADG9783

LMR-Geo-

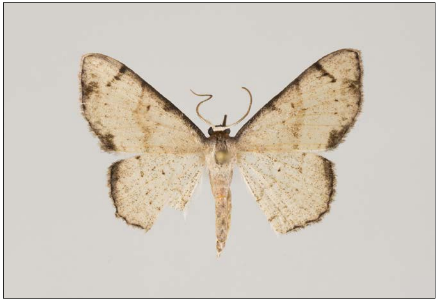

Compared specimen:

LMR-Geo-
0181

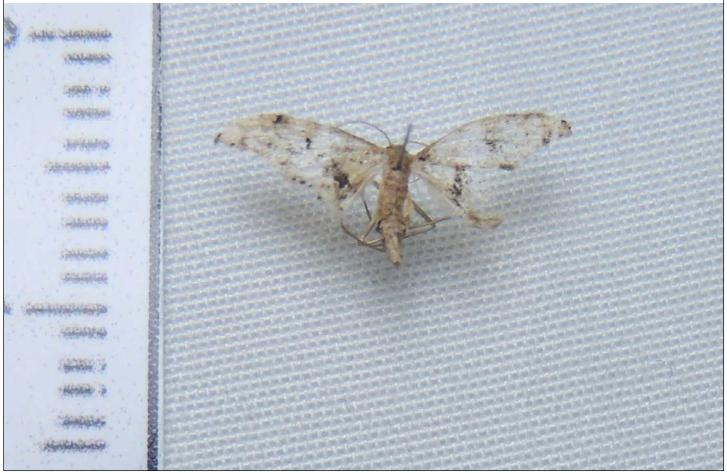

Idaea sp (TL:)

BC

BOLD:AEE2174
OTU-199

Additional compared specimen

LMR-Geo-

Compared specimen:

LMR-Geo-
0257

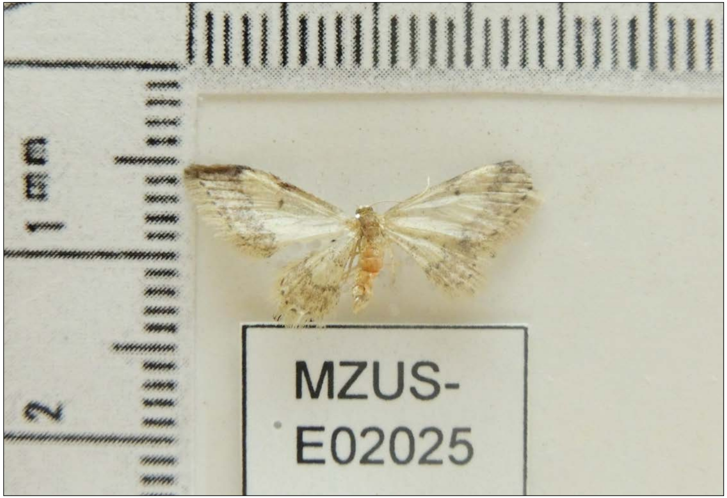

Idaea sp (TL:)

BC

BOLD:AEE4433

OTU-148

Additional compared specimen

LMR-Geo-

Compared specimen:

LMR-Geo-
0184

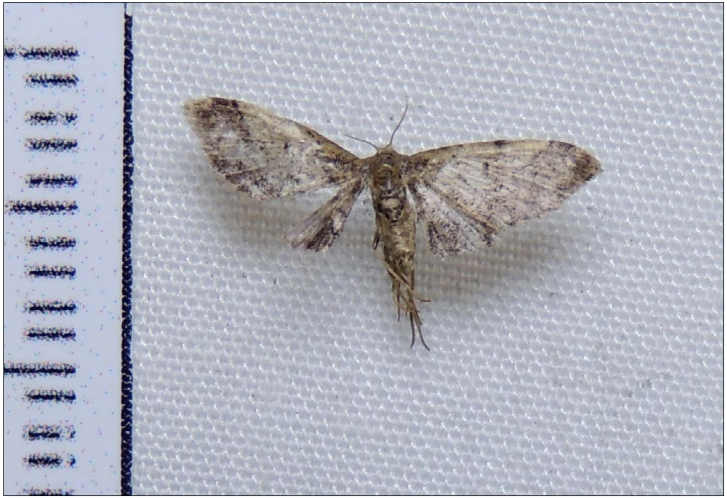

Idaea sp (TL:)

BC

BOLD:AEE4433
OTU-148

Additional compared specimen

LMR-Geo-

Compared specimen:

LMR-Geo-
0178

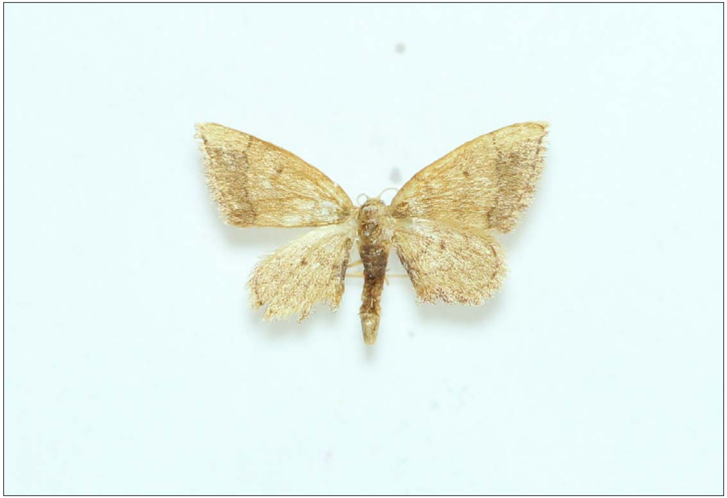

Idaea sp (TL:)

BC

BOLD:AAI7627
OTU-169

Additional compared specimen

LMR-Geo-

Compared specimen:

LMR-Geo-
0198

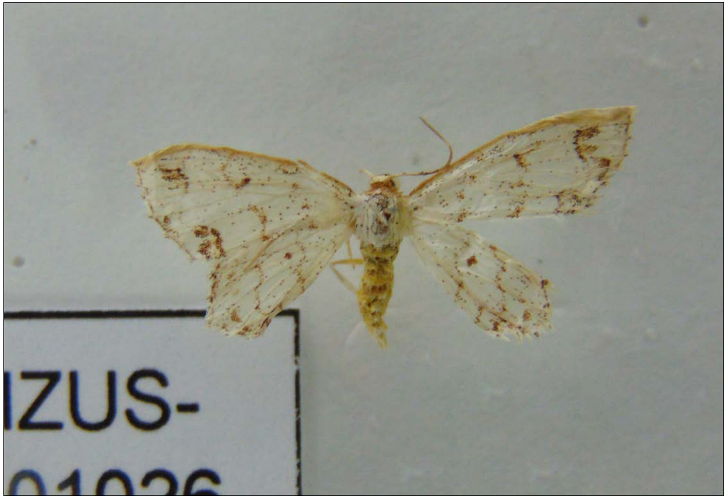

Idaea sp (TL:)

BC

BOLD:AAI7627
OTU-29

Additional compared specimen

LMR-Geo-

Compared specimen:

LMR-Geo-
0335

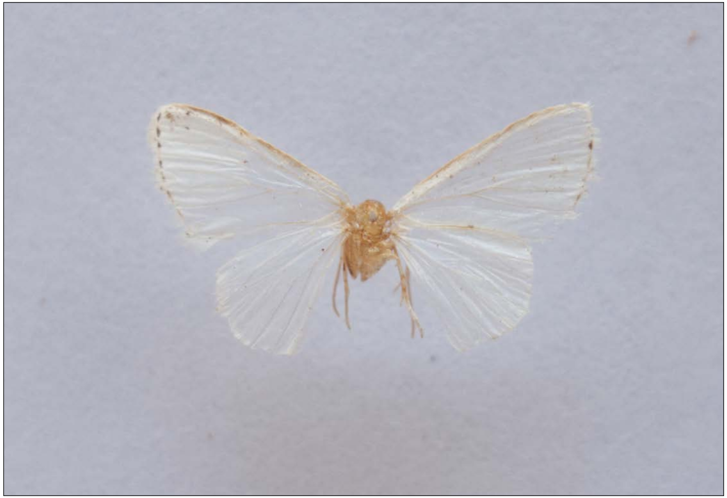

Idaea sp (TL:)

BC

BOLD:AEE5660	
OTU-197	

Additional compared specimen
distant: Ec-Geo-15663|Ecuador|Zamora Chinchipe|BOLD:AAF2870

LMR-Geo-

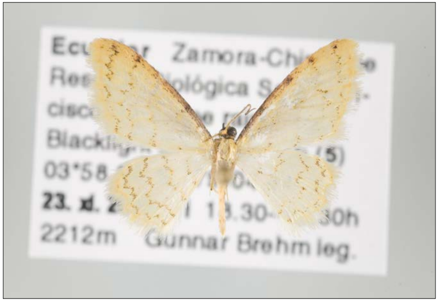

Compared specimen:

LMR-Geo-
0252 (no photo available)

Idaea sp (TL:)

BC

BOLD:AEE6278
OTU-207

Additional compared specimen

LMR-Geo-

Compared specimen:

LMR-Geo-

0242 (no photo available)

Idaea sp (TL:)

BC

BOLD:AEE6279
OTU-145

Additional compared specimen

LMR-Geo-

Compared specimen:

LMR-Geo-

0180

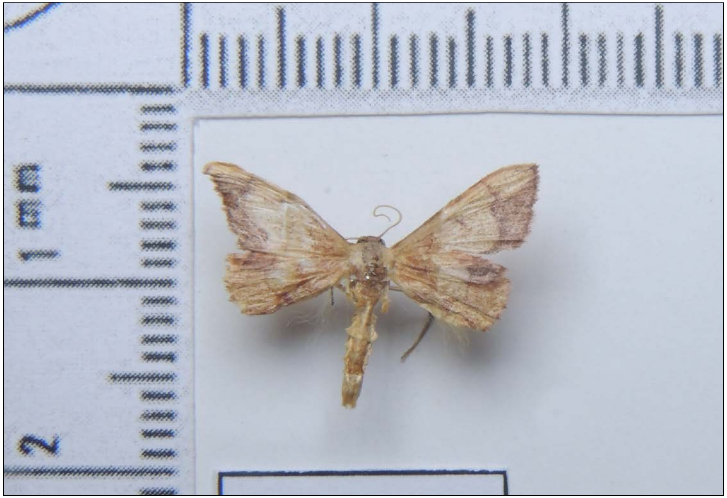

Idaea sp (TL:)

BC

BOLD:AEE6280
OTU-195

Additional compared specimen

LMR-Geo-

Compared specimen:

LMR-Geo-
0261

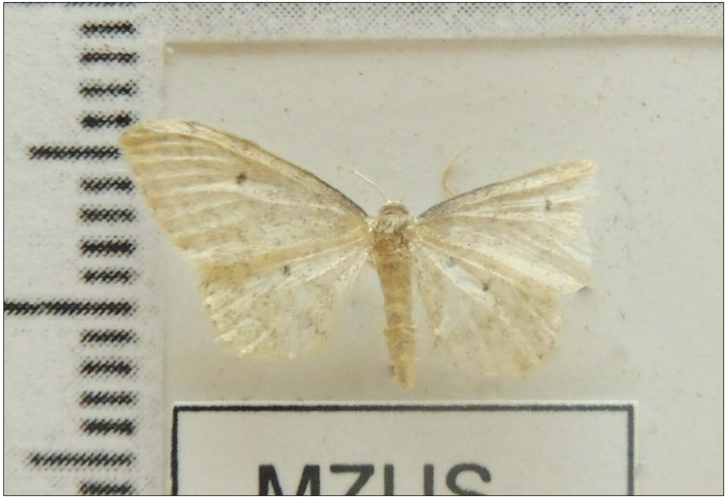

Leptostales nr terminata Guenée (TL: Colombia)

BC
BOLD:AAE2911
OTU-150

Additional compared specimen
= Ec-Geo-45546|Ecuador|Zamora Chinchipe|BOLD:AAE2911

LMR-Geo-

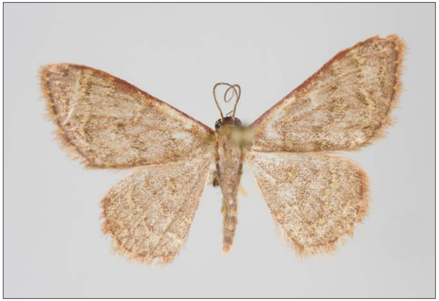

Compared specimen:
NHM type

LMR-Geo-
0188

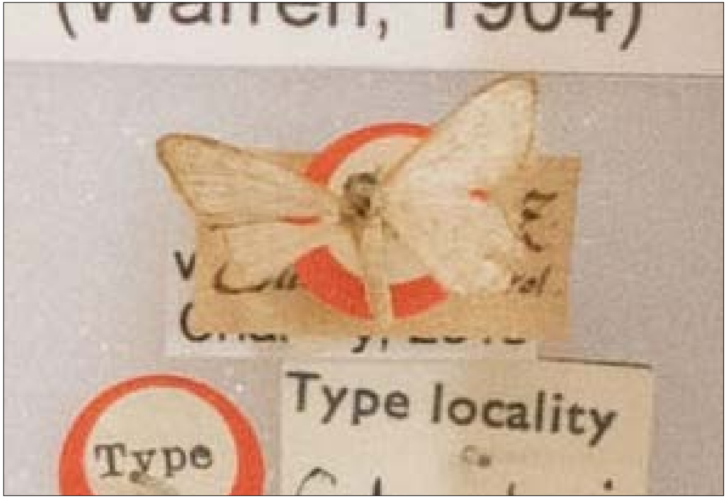

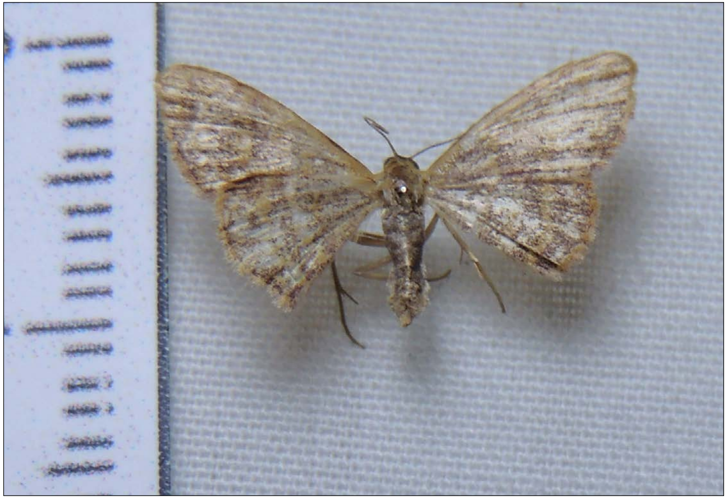

Leptostales nr terminata Guenée (TL: Colombia)

BC

BOLD:AEB9272
OTU-35

Additional compared specimen

LMR-Geo-
0342

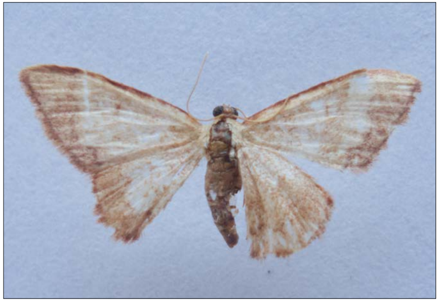

Compared specimen:

LMR-Geo-
0235

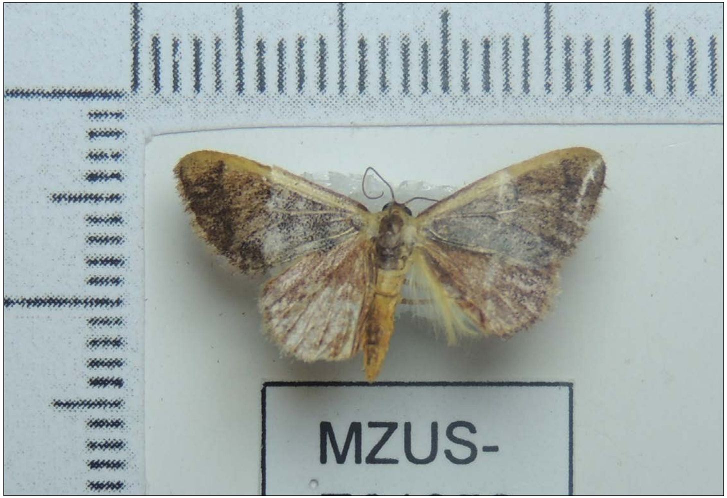

Lobocleta subcincta Dognin (TL: Colombia: Micay)

BC

BOLD:AEC0946
OTU-43

Additional compared specimen

LMR-Geo-

Compared specimen:

USNM type

LMR-Geo-

0352

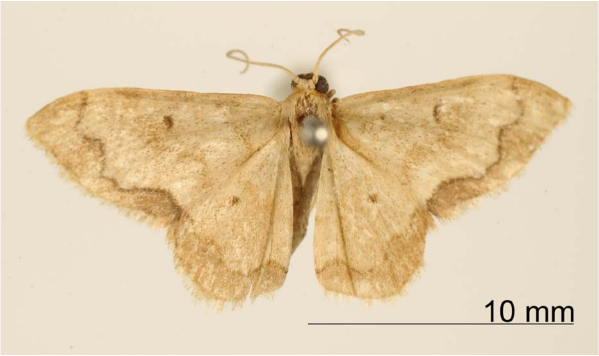

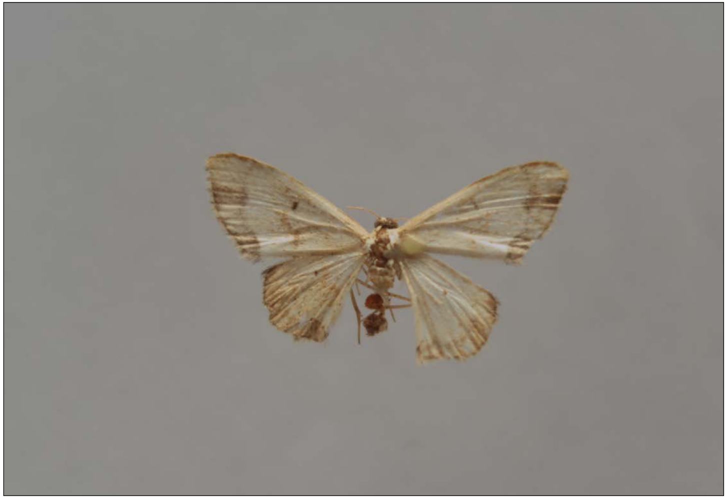

Lobocleta sp (TL:)

BC

BOLD:AAJ2315
OTU-38

Additional compared specimen

LMR-Geo-

Compared specimen:

LMR-Geo-

0346

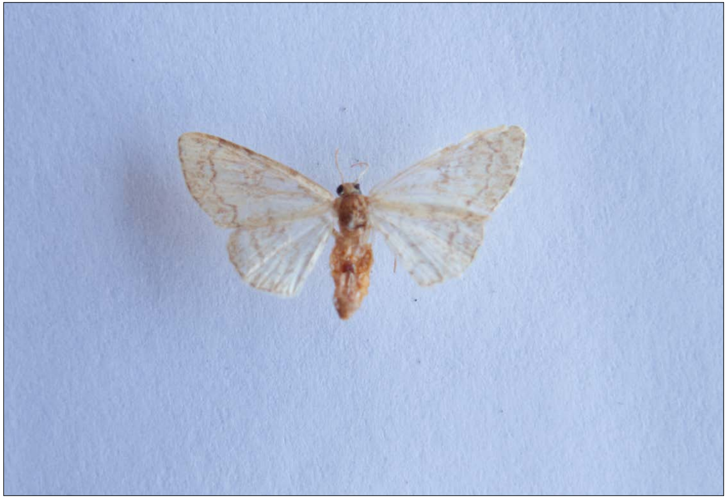

Pleuroprucha rudimentaria Guenée (TL: [Hispaniola]: Haiti)

BC

BOLD:AAB9656
OTU-204

Additional compared specimen
= Pe-Geo-1078|Peru|Huanuco

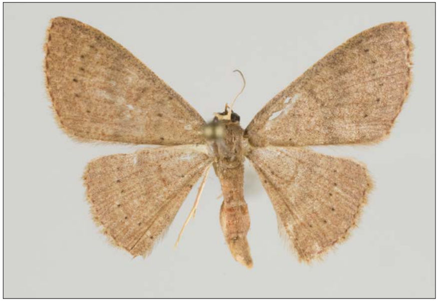

LMR-Geo-

Compared specimen:
NHM type

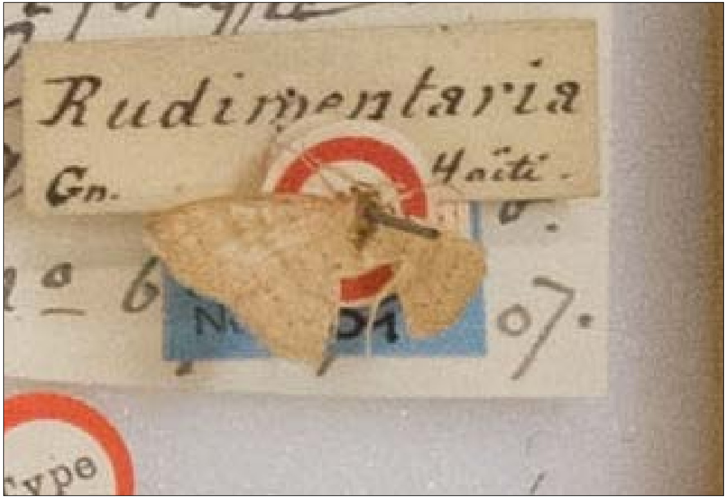

LMR-Geo-
0240

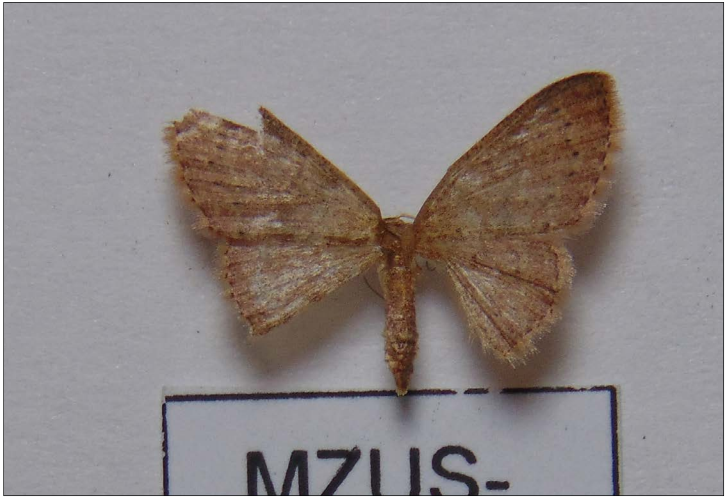

Ptychamalia cumana Schaus (TL: Mexico: Orizaba)

BC

BOLD:AAC2156
OTU-151

Additional compared specimen

LMR-Geo-

Compared specimen:

USNM type

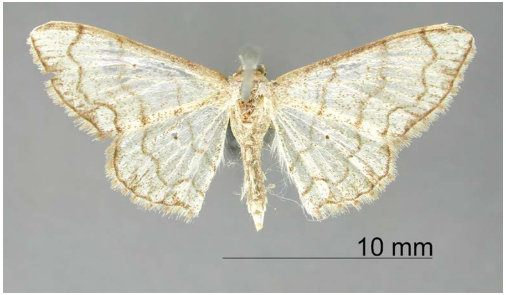

LMR-Geo-

0191

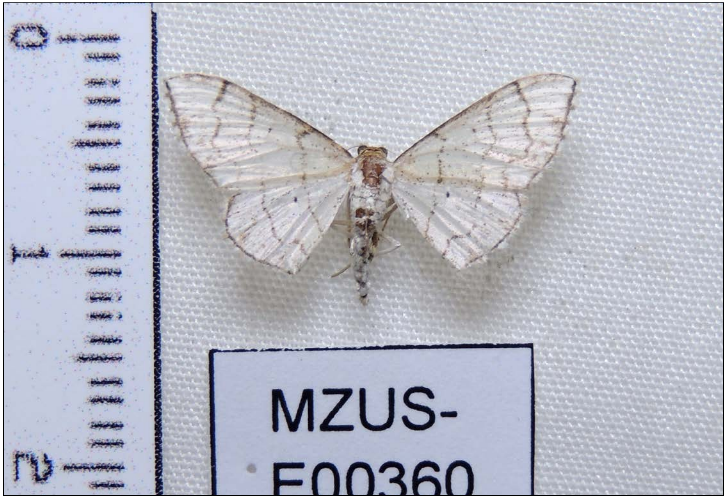

Scopula subquadrata Guenée (TL: Brazil)

BC

BOLD:AAA9026
OTU-28

Additional compared specimen

LMR-Geo-
0336, 0345

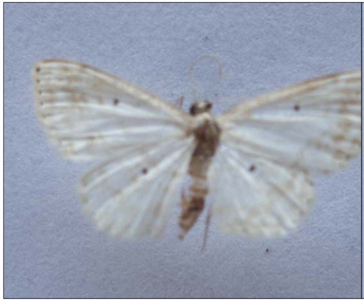

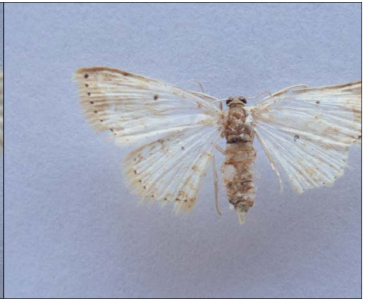

Compared specimen:
NMH paralectotype of *subquadrata*

LMR-Geo-
0239

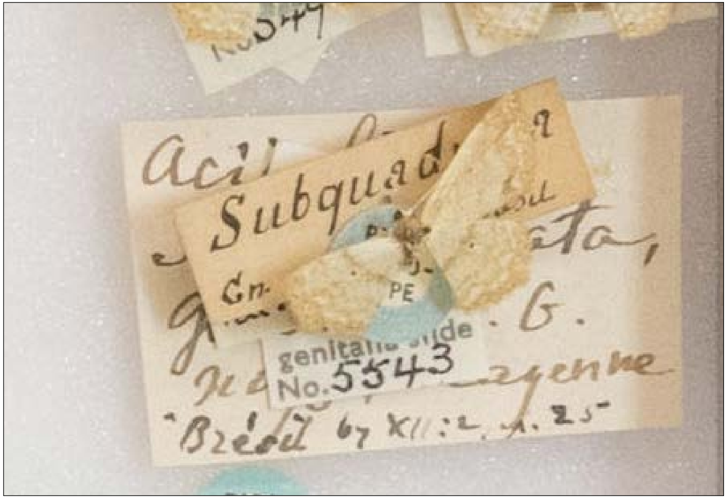

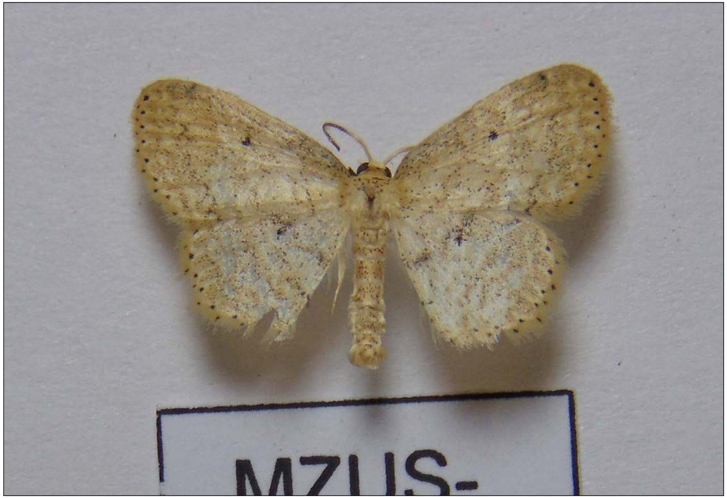

Scopula umbilicata group Fabricius (TL: Americae meridionalis Ins. [West Indies])

BC

BOLD:AEE2890
OTU-149

Additional compared specimen

LMR-Geo-
0186 (no photo), 0187

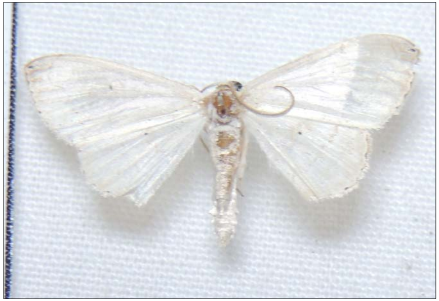

Compared specimen:
NHM no type

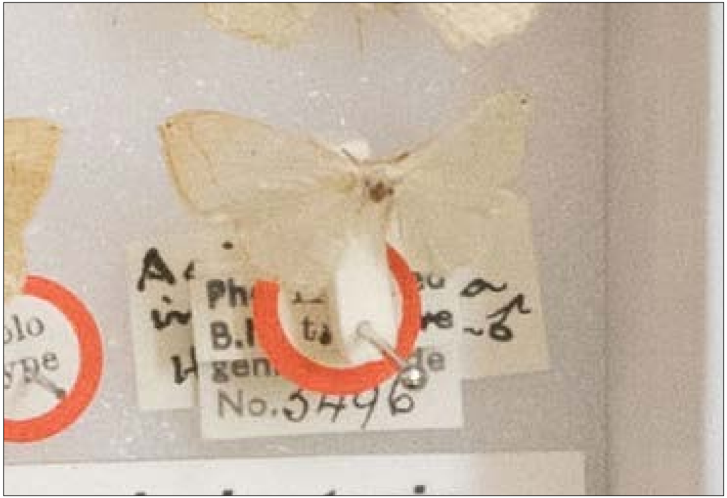

LMR-Geo-
0192

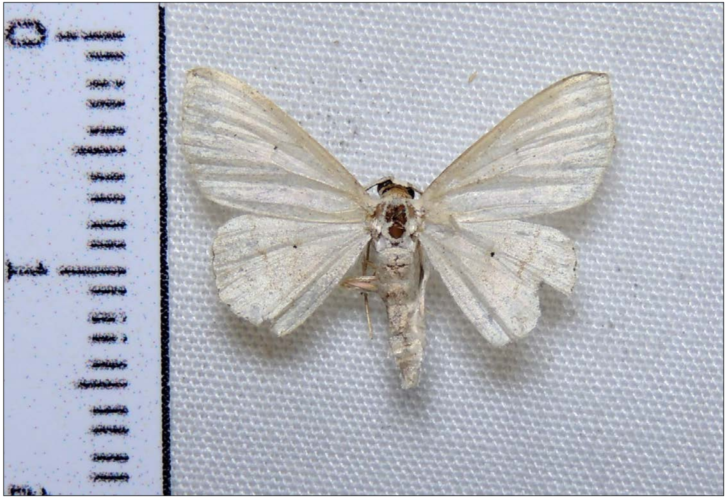

Scopula sp (TL:)

BC

BOLD:AAI7474
OTU-168

Additional compared specimen
near Pe-Geo-1089|Peru|Huanuco|BOLD:ADF7247

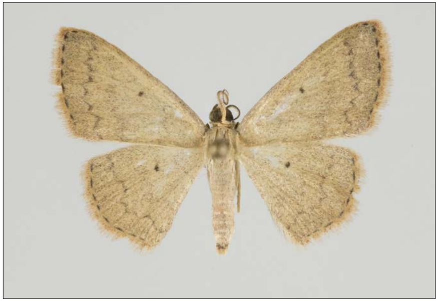

LMR-Geo-
0199

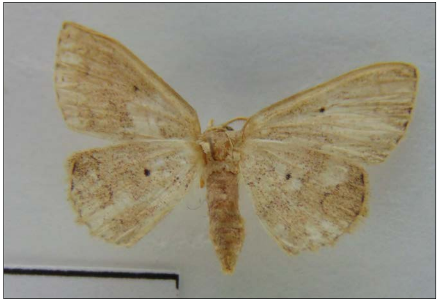

Compared specimen:

LMR-Geo-
0200

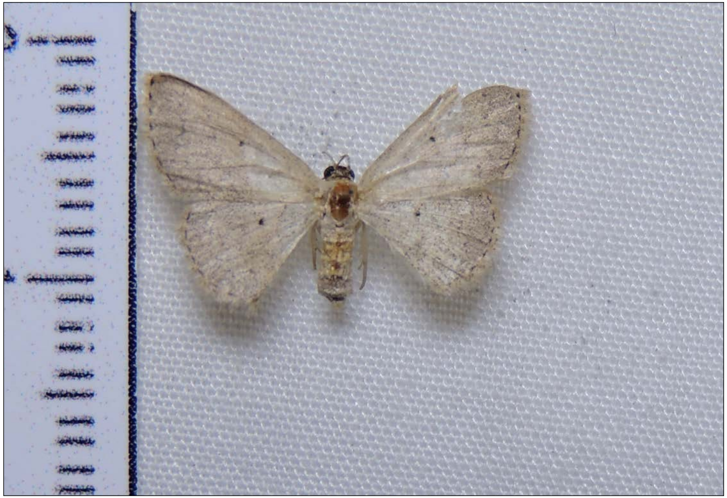

Scopula sp (TL:)

BC

BOLD:ACR5955
OTU-34

Additional compared specimen
distant Pe-Geo-1288|Peru|Huanuco|BOLD:AAT9472

LMR-Geo-

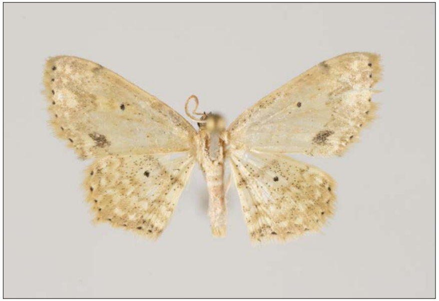

Compared specimen:

LMR-Geo-
0359

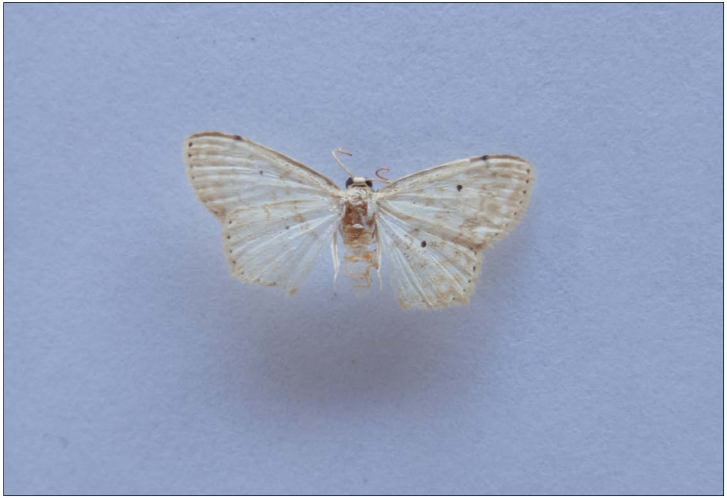

Scopula sp (TL:)

BC

no BIN / 461[2n]

OTU-205

Additional compared specimen
distant: Pe-Geo-0681|Peru|Cuzco|BOLD:ADE9632

LMR-Geo-

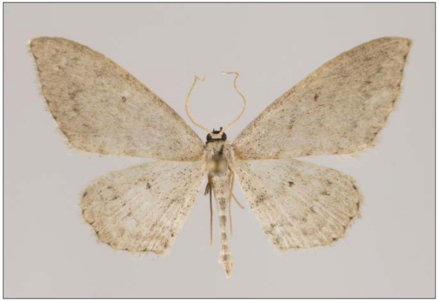

Compared specimen:

LMR-Geo-
0237

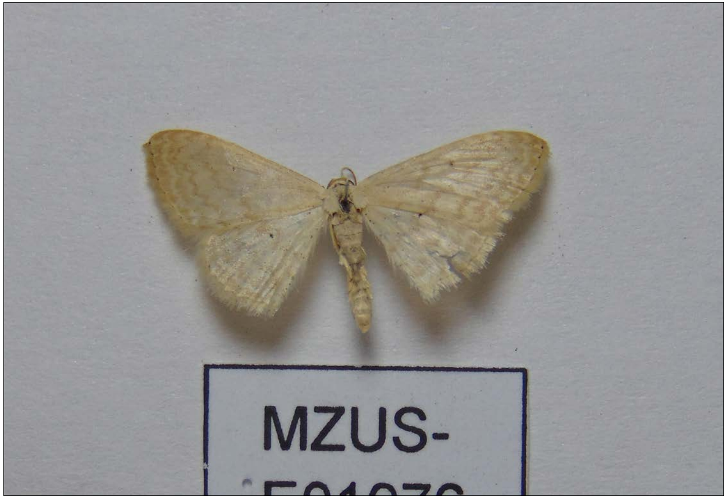

Semaeopus nr argocosma Prout (TL: Jamaica: Montego Bay)

BC

BOLD:AEE9510

OTU-171

Additional compared specimen

LMR-Geo-

Compared specimen:

NHM type

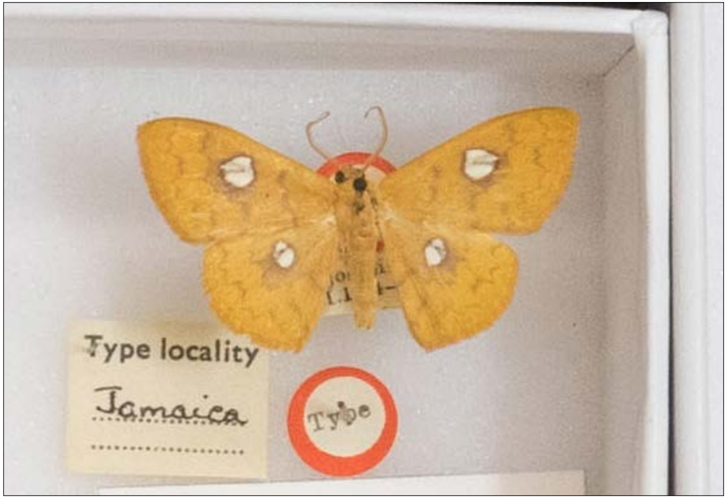

LMR-Geo-

0195

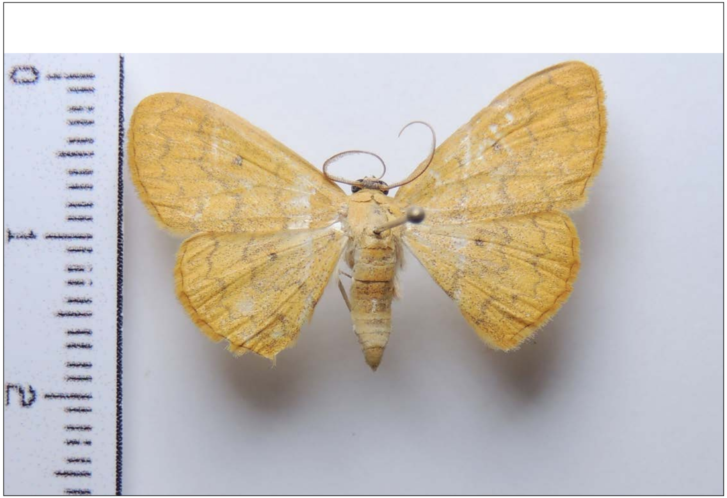

Semaeopus nr purpurea Warren (TL: French Guiana: St Jean [du Maroni], Maroni River)

BC

BOLD:AEE4607

OTU-210

Additional compared specimen

LMR-Geo-
0129

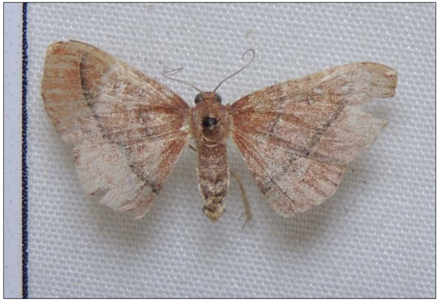

Compared specimen:
USNM type

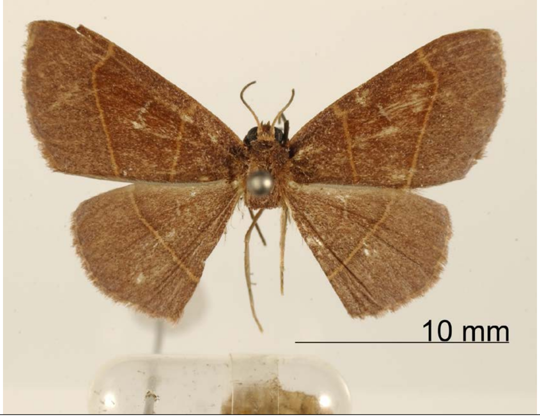

LMR-Geo-
0194

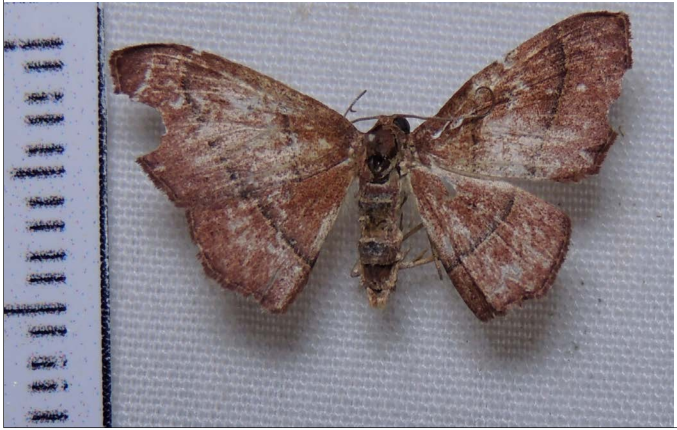

Semaeopus nr varia Warren (TL: [Guyana]: Rio Demerara)

BC

BOLD:ADW9153
OTU-226

Additional compared specimen
distant: Pe-Geo-1047|Peru|Huanuco|BOLD:AAI7476

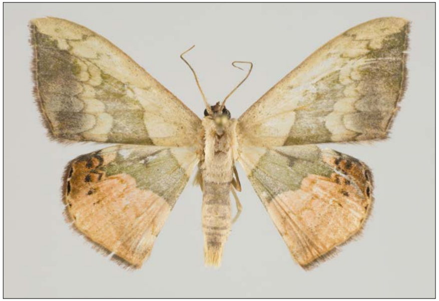

LMR-Geo-

Compared specimen:

NHM type

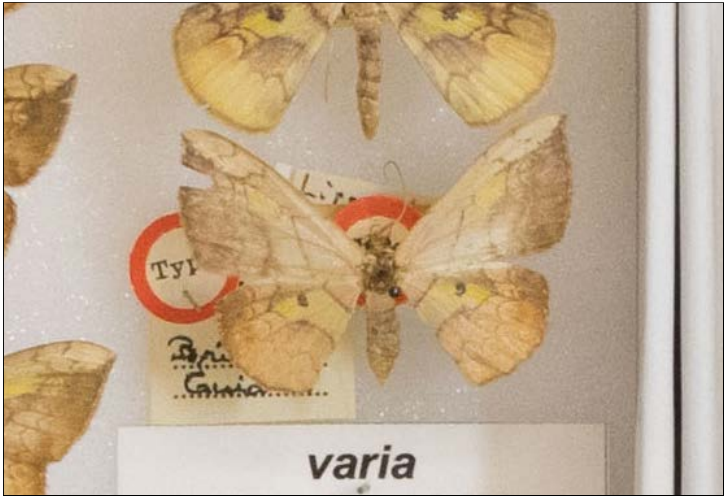

LMR-Geo-

0193

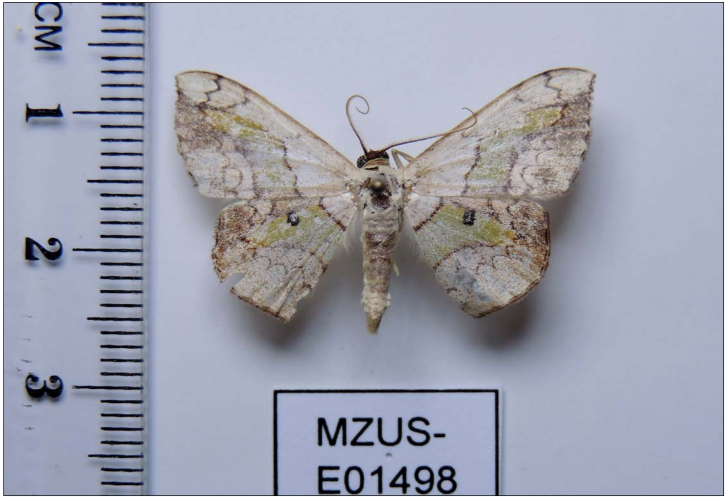

Semaeopus sp (TL:)

BC

BOLD:AEE5092
OTU-170

Additional compared specimen

LMR-Geo-

Compared specimen:

LMR-Geo-

0196

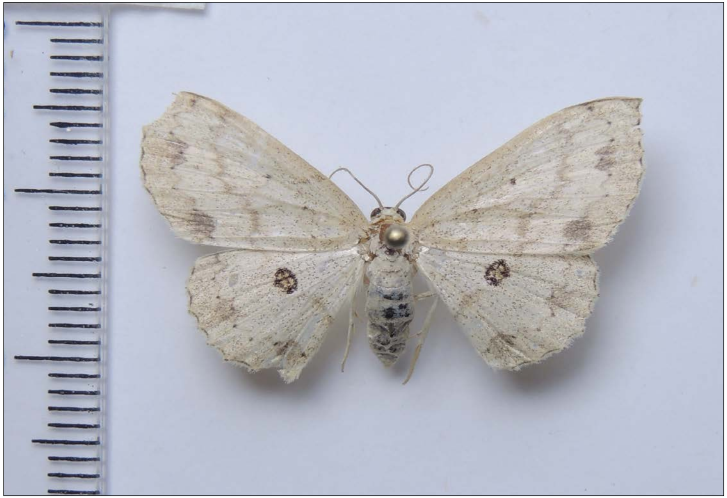

Tricentra nr navatteae Herbulot (TL: French Guiana: near Cayenne, 53 km along the Regina road)

BC

BOLD:AEE1748
OTU-61

Additional compared specimen

LMR-Geo-
0005

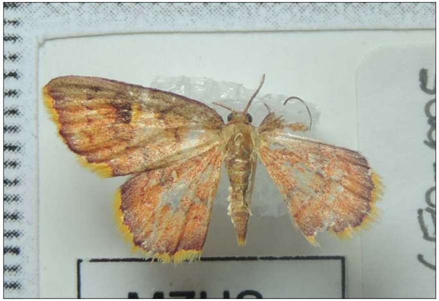

Compared specimen:
NHM type

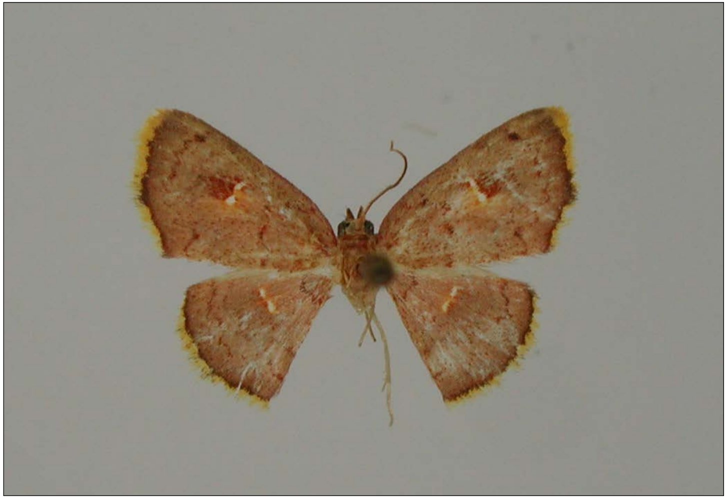

LMR-Geo-
0003

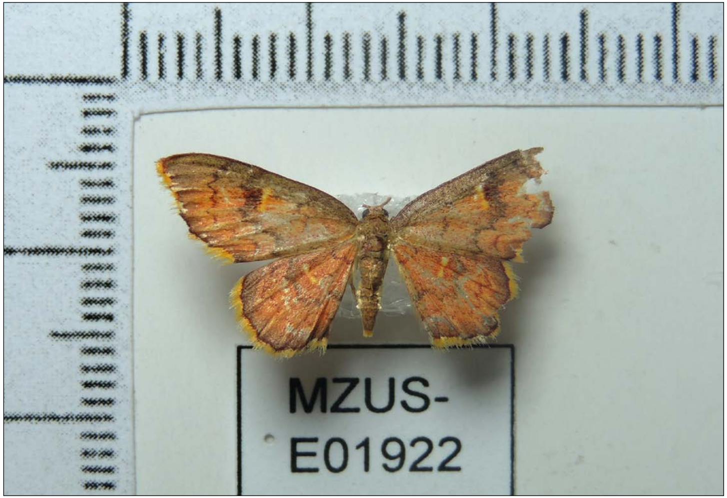

Tricentrogyna nr collustrata Snellen (TL: Colombia, Cucqueta)

BC

BOLD:AEE5660
OTU-67

Additional compared specimen
distant: Ec-Geo-22264|Ecuador|Loja|BOLD:AAM5161

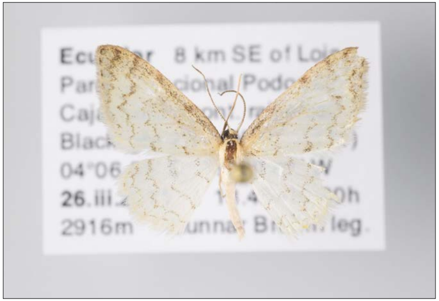

LMR-Geo-

Compared specimen:

NHM no type

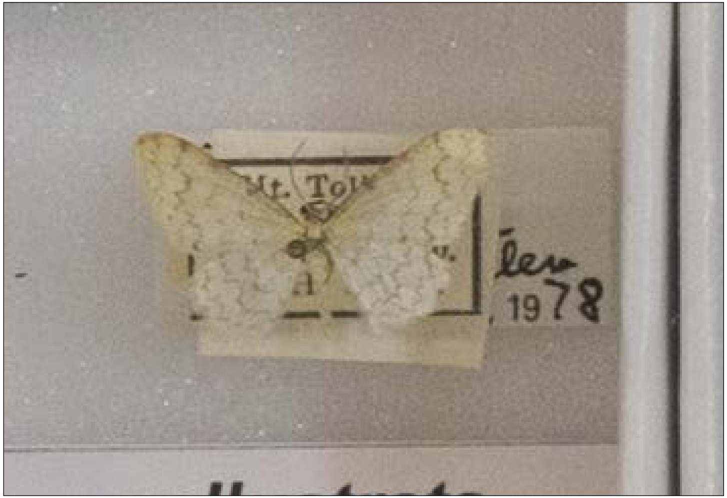

LMR-Geo-
0017

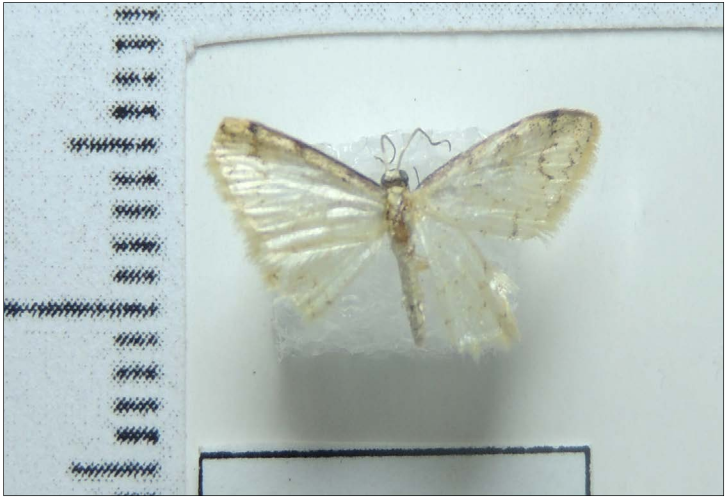

NN Sterrhinae (TL:)

BC

no BIN 348 bp

OTU-224

Additional compared specimen

LMR-Geo-

Compared specimen:

LMR-Geo-

0278

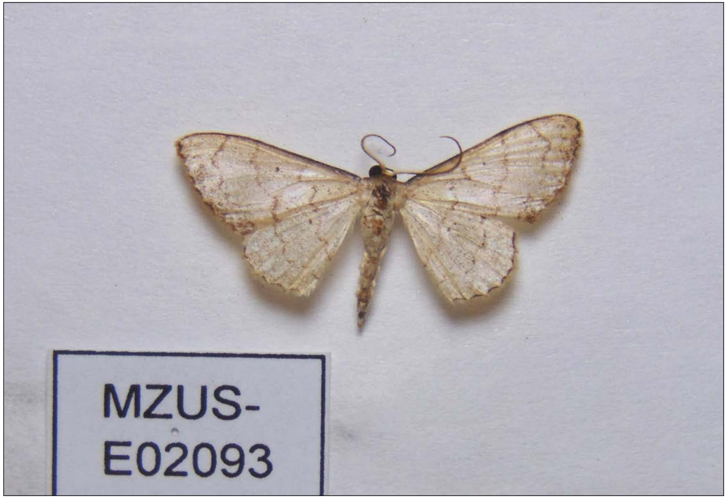

NN Sterrhinae

BC

BOLD:AEE6089
OTU-227

Additional compared specimen

LMR-Geo-

Compared specimen:

LMR-Geo-
0189

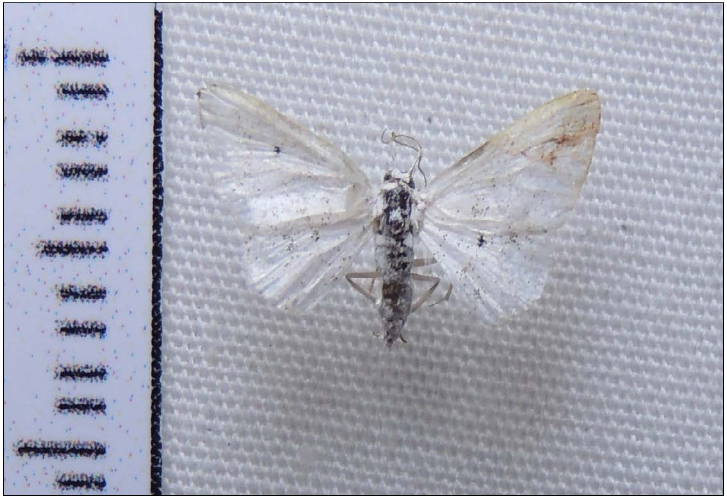

NN Sterrhinae

BC

BOLD:AEE6088
OTU-225

Additional compared specimen

LMR-Geo-

Compared specimen:

LMR-Geo-
0197

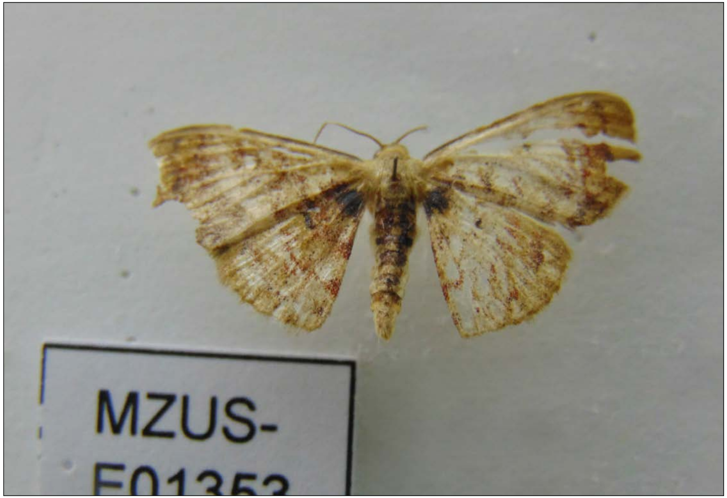

Supplement: Supplementary material 4 — Illustrated catalogue of all new records of Colombian Geometridae, part 2 [file bdj-09-e68693-s004.pdf]
