# Supplementary material for: A database and checklist of geometrid moths (Lepidoptera) from Colombia
Source: Biodivers Data J. 2021 Sep 3;9:e68693. doi: 10.3897/BDJ.9.e68693 (PMC8433126; doi:10.3897/BDJ.9.e68693)

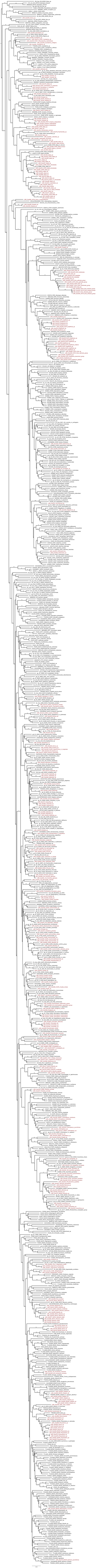

Supplement: Supplementary material 2 — Maximum Likelihood tree (ML) of Colombian geometrids including COI, Wingless and EF1a markers [file bdj-09-e68693-s002.pdf]
